# Supplementary material for: Serial high-sensitivity cardiac troponin testing for the diagnosis of myocardial infarction: a scoping review
Source: BMJ Open. 2022 Nov 22;12(11):e066429. doi: 10.1136/bmjopen-2022-066429 (PMC9685223; doi:10.1136/bmjopen-2022-066429)
Supplement: Supplementary data [file bmjopen-2022-066429supp001.pdf]

Supplementary material

Search Strategy

MEDLINE by Ovid

1 exp Chest Pain/
2 (chest adj2 pain*).ti,ab.
3 chest discomfort.ti,ab.
4 exp Acute Coronary Syndrome/
5 acute coronary syndrome*.ti,ab.
6 ACS.ti,ab.
7 exp Angina, Unstable/
8 unstable angina.ti,ab.
9 Myocardial Infarction/di
10 myocardial infarction.ti,ab.
11 heart attack.ti,ab.
12 or/1-11
13 cardiac troponin*.ti,ab.
14 high* sensitiv* troponin*.ti,ab.
15 hs-ctn*.ti,ab.
16 elecsys.ti,ab.
17 architectstat.ti,ab.
18 architect stat.ti,ab.
19 accutni*.ti,ab.
20 accutnl*.ti,ab.
21 centaur ultra.ti,ab.
22 HISCL.ti,ab.
23 vidas.ti,ab.
24 lumipulse.ti,ab.
25 serial troponin.ti,ab.
26 contemporary troponin.ti,ab.
27 heart adp.ti,ab.
28 (EDACS and troponin).ti,ab.
29 (Adapt adp and troponin).ti,ab.
30 (GRACE and troponin).ti,ab.
31 (TIMI and troponin).ti,ab
32 or/13-31

33 exp Emergency Service, Hospital/
34 (emergency adj (room* or department*)).ti,ab.
35 (ER or ED).ti,ab.
36 (presenting or presented).ti,ab.
37 presentation.ti,ab
38 (admission or admitted).ti,ab.
39 chest pain unit.ti,ab.
40 or/33-39
41 12 and 32 and 40

Embase by Elsevier

1 exp Chest Pain/
2 (chest adj2 pain*).ti,ab.
3 chest discomfort.ti,ab.
4 exp Acute Coronary Syndrome/
5 acute coronary syndrome*.ti,ab.
6 ACS.ti,ab.
7 exp Angina, Unstable/
8 unstable angina.ti,ab.
9 Myocardial Infarction/di
10 myocardial infarction.ti,ab.
11 heart attack.ti,ab.
12 or/1-11
13 cardiac troponin*.ti,ab.
14 high* sensitiv* troponin*.ti,ab.
15 hs-ctn*.ti,ab.
16 elecsys.ti,ab.
17 architectstat.ti,ab.
18 architect stat.ti,ab.
19 accutni*.ti,ab.
20 accutnl*.ti,ab.
21 centaur ultra.ti,ab.
22 HISCL.ti,ab.
23 vidas.ti,ab.
24 lumipulse.ti,ab.
25 serial troponin.ti,ab.

26 or/13-25
27 exp Emergency Service, Hospital/
28 (emergency adj (room* or department*)).ti,ab.
29 (ER or ED).ti,ab.
30 (presenting or presented).ti,ab.
31 presentation.ti,ab.
32 (admission or admitted).ti,ab.
33 chest pain unit.ti,ab.
34 or/27-33
35 12 and 26 and 34
36 limit 35 to yr="2006 -Current"

Cochrane Database of Systematic Reviews and CENTRAL database by the Cochrane Library

#1 MeSH descriptor: [Chest Pain] this term only
#2 (chest next pain):ti,ab
#3 (chest next discomfort):ti,ab
#4 MeSH descriptor: [Acute Coronary Syndrome] explode all trees
#5 ACS:ti,ab
#6 (acute next coronary next syndrome):ti,ab
#7 MeSH descriptor: [Coronary Artery Disease] explode all trees
#8 (coronary next artery next disease):ti,ab
#9 MeSH descriptor: [Angina, Unstable] explode all trees
#10 (unstable next angina):ti,ab
#11 MeSH descriptor: [Myocardial Infarction] explode all trees
#12 (myocardial next infarction):ti,ab
#13 (heart next attack):ti,ab
#14 #1 or #2 or #3 or #4 or #5 or #6 or #7 or #8 or #9 or #10 or #11 or #12 or #13
#15 MeSH descriptor: [Troponin] explode all trees
#16 troponin:ti,ab
#17 hs-ctn*:ti,ab
#18 #15 or #16 or #17
#19 MeSH descriptor: [Emergency Service, Hospital] explode all trees
#20 (emergency next (room* or department*)):ti,ab
#21 (ER or ED):ti,ab
#22 (presenting or presented):ti,ab

#23 presentation:ti,ab

#24 (admission or admitted):ti,ab

#25 "chest pain unit":ti,ab

#26 #19 or #20 or #21 or #22 or #23 or #24 or #25

#27 #14 and #18 and #26

Science Citation Index

#1 TITLE: (high* sensitiv* troponin)

#2 TITLE: ("serial troponin" or "serial cardiac troponin")

#3 TOPIC: ("myocardial infarction*" or "heart attack*" or "heart failure")

#4 TOPIC: (diagnosi* or detect* or identif*)

#5 #2 OR #1

#6 #5 AND #4 AND #3

Appendix Table 1. Included studies reporting the diagnostic accuracy of AMI and 30-day clinical outcomes.

Author/publish year[*]	Number of patients[†]	Age[‡], year	Renal dysfunction[‖]	Study name or region	Enrolment years	Need of ECG[¶]	Clinical outcomes[**]
**Troponin I assays manufactured by Abbott**							
0 and 1h protocol							
Gimenez 2015[1]	1811/2377 (76)	62 (49-62)	Excl	APACE	2006-2012		30-day
Pickering 2016a[2]	2222 (ND)	60 (±14)	Incl	ADAPT, ADAPT-ADP, EDACS-ADP, RING	ADAPT: 2007-2011; ADAPT-ADP: 2010-2012; EDACS: 2013-2014; RING: ND	Excl	Dx MI
Neumann 2016[3]	1040 (ND)	65 (52-75)	Incl	BACC	2013-2014		Dx MI
Boeddinghaus 2017[4]	2828/3642 (78)	62 (49-74)	Excl	APACE	2006-2015		Dx MI, 30-day
Neumann 2017[5]	1516 (ND)	65 (51-75)	Incl	BACC	2013-2016		Dx MI
Twerenbold 2018a[6]	2504<br>445 /4168 (71)	58 (47-70)[‡‡]<br>79 (73-84)[§§]	Excl<br>Incl	APACE<br>APACE	ND<br>ND		Dx MI, 30-day<br>Dx MI, 30-day
Twerenbold 2018b[7]	3500/5631 (62)	62[‖‖]	P-Incl	APACE, BACC	2006-2016		Dx MI, 30-day
Boeddinghaus 2018a[8]	4269/5136 (83)	62 (49-74)	P-Incl	APACE, BACC, TRAPID-AMI	2006-2015		Dx MI, 30-day
Wildi 2019[9]	2945/4169 (71)	62 (49-74)	Excl	APACE	2006-2015	P-need	Dx MI, 30-day
Nestelberger 2019a[10]	2828/3980 (71)	62 (49-74)	Excl	APACE	2006-2015		Dx MI, 30-day
Boeddinghaus 2019[11]	937/1288 (73)	60 (48-74)	Excl	APACE	2011-2015		Dx MI
Neuman 2019[12]	1534 (ND)	65[‖‖]	Incl	BACC	ND		Dx MI
Sörensen 2019[13]	1279 (ND)	65[‖‖]	Incl	BACC	2015-2016		Dx MI
Boeddinghaus 2020[14]	962/1328 (72)	60 (47-73)	Excl	APACE	2011-2014		Dx MI, 30-day
Sörensen 2021a[15]	937 (ND)	65 (52-75)	Incl	BACC	2013-2016		Dx MI
Sörensen 2021b[16]	1800 (ND)	65 (52-75)	Incl	BACC	2013-2014		Dx MI
Nestelberger 2022[17]	862/6021 (14)	61 (49-74)	Excl	APACE	2006-2018		Dx MI
0 and 2h protocol							
Cullen 2013[18]	2544 (ND)	61[‖‖]	P-Incl	APACE, ADAPT	APACE: 2006-2008; ADAPT: 2007-2011	P-need	30-day
Aldous 2014[19]	962 (ND)	66 (56-76)	Incl	ASPECT, ADAPT	2007-2010		Dx MI, 30-day
Cullen 2014[20]	1959/1571 (80)	60[‖‖]	Incl	Australia/New Zealand	2007-2011		Dx MI
Eggers 2015[21]	1624 (ND)	61 (±15)	Incl	ADAPT	2007-2011		Dx MI
Boeddinghaus 2016[22]	2629 (ND)	62[‖‖]	P-Incl	APACE, ADAPT	APACE: 2006-2012; ADAPT: 2007-2011	P-need	Dx MI, 30-day
Wildi 2017[23]	2525/4417 (57)	62[‖‖]	P-Incl	APACE, ADAPT	APACE: 2006-2013; ADAPT: 2007-2011	P-need	Dx MI, 30-day
Lindahl 2017[24]	1197 (ND)	64[‖‖]	P-Incl	APACE, FASTEST	APACE: 2008-2014, FASTEST: 2013-2014		Dx MI, 30-day
Wildi 2019[9]	2357/4169 (57)	62 (50-74)	Excl	APACE	2006-2015	P-need	Dx MI, 30-day
Nestelberger 2019b[25]	2411/2979 (81)	56[‖‖]	P-Incl	APACE, ADAPT, IMPACT	APACE: ND, ADAPT: 2007-2011, IMPACT: 2011-2014		Dx MI, 30-day
Kavsak 2021[26]	935/1366 (68)	69[‖‖]	Incl	ROMI-3	2013		30-day
Koechlin 2021[27]	1863/4587 (41)	61 (50-74)	Excl	APACE	ND		Dx MI
Nestelberger 2022[17]	660/6021 (11)	61 (49-74)	Excl	APACE	2006-2018		Dx MI
0 and 3h protocol							
Keller 2011[††28]	1818 (ND)	61 (±14)	Incl	Germany	2007-2008		Dx MI
Neumann 2016[3]	1040 (ND)	65 (52-75)	Incl	BACC	2013-2014		Dx MI
Wildi 2016[29]	2226/2922 (76)	62 (49-74)	Excl	APACE	2006-2013		Dx MI
Parsonage 2016[30]	3128/3580 (87)	61[‖‖]	P-Incl	APACE, Australia/New Zealand	Australia/New Zealand: 2008-2011; APACE: 2006-2013		30-day
Pickering 2016b[31]	1172 (ND)	60 (±14)	Incl	ADAPT, ADAPT-ADP, EDACS-ADP, RING	ADAPT: 2007-2011; ADAPT-ADP: 2010-2012; EDACS: 2013-2014; RING: ND	Excl	Dx MI
Neumann 2017[5]	1516 (ND)	65 (51-75)	Incl	BACC	2013-2016		Dx MI
Chapman 2017[32]	1218/1292 (94)	62 (±14)	Incl	High-STEACS	2013-2015		Dx MI, 30-day
Sandoval 2017[33]	1068 (ND)	ND	Incl	UTROPIA	2014		Dx MI
Chapman 2018[34]	1935/1951 (99)	62 (±14)	Incl	High-STEACS	2013-2017	P-need	Dx MI, 30-day
Gunsolus 2018[35]	1555 (ND)	58 (±15)	Incl	UTROPIA	2014		Dx MI
Wildi 2019[9]	1742/4169 (42)	63 (50-76)	Excl	APACE	2006-2015	P-need	Dx MI, 30-day
Kim 2020[36]	281/362 (78)	59[‖‖]	ND	Korea, Seoul	2015-2016		Dx MI
Sandoval 2020[37]	582 (ND)	ND	Incl	UTROPIA	2014		30-day
Dupuy 2021[38]	ND	73 (±17)	Incl	Montpellier, France	ND		Dx MI
Miscellaneous							
Gimenez 2014[39]	2226/2956 (75)	62 (50-75)	Excl	APACE	2006-2012		Dx MI
Pickering 2015[40]	368 (ND)	61 (±13)	Incl	Australia/New Zealand	2010-2012	P-need	Dx MI
Chapman 2017[32]	1218/1292 (94)	62 (±14)	Incl	High-STEACS	2013-2015		Dx MI, 30-day

Author/publish year[*]	Number of patients[†]	Age[‡], year	Renal dysfunction[‖]	Study name or region	Enrolment years	Need of ECG[¶]	Clinical outcomes[**]
Sandoval 2017[33]	1631 (ND)	ND	Incl	UTROPIA	2014		Dx MI, 30-day
Miller-Hodges 2018[41]	2193/4789 (46)	64 (±16)	Incl	UK	2013-2014		Dx MI, 30-day
**Troponin T assays manufactured by Roche**							
0 and 1h protocol							
Reichlin 2011[42]	836/1197 (70)	64 (51-76)	Excl	APACE	2007-2009		Dx MI
Reichlin 2012[43]	872/1197 (73)	64 (51-75)	Excl	APACE	2006-2009		Dx MI, 30-day
Reichlin 2015a[44]	1320/1656 (80)	60 (49-73)	Excl	APACE	2007-2009		Dx MI, 30-day
Mokhtari 2016[45]	1038 (ND)	61 (± 18)	Incl	Sweden	2013-2014		Dx MI, 30-day
Mokhtari 2017[46]	1020/1167 (87)	61 (±17)	Incl	Sweden	2013-2014	Excl	30-day
Mueller 2016[††][47]	1282 (ND)	62 (50-74)	Excl	TRAPID-AMI	2011-2013		Dx MI, 30-day
Pickering 2016a[2]	2222 (ND)	60 (± 14)	Incl	ADAPT, ADAPT-ADP, EDACS-ADP, RING	ADAPT: 2007-2011; ADAPT-ADP: 2010-2012; EDACS: 2013-2014; RING: ND	Excl	Dx MI
Shiozaki 2017[48]	413 (ND)	72 (59-81)	Incl	Japan	2014-2017		Dx MI, 30-day
Twerenbold 2018a[6]	2767 /4168 (78)<br>487	58 (47-70)[‡‡]<br>79 (73-84)[§§]	Excl<br>Excl	APACE<br>APACE	ND<br>ND		DX MI, 30-day<br>DX MI, 30-day
Twerenbold 2018b[7]	4368/5631 (78)	62 (50-74)	P-Incl	APACE, BACC	2006-2016		DX MI, 30-day
Boeddinghaus 2018a[8]	5890/6744 (87)	61 (49-74)	P-Incl	APACE, BACC, TRAPID-AMI	2006-2015		Dx MI, 30-day
Mueller-Hennessen2019[49]	922/1282 (72)	62 (49-74)	Excl	TRAPID-AMI	ND		Dx MI, 30-day
Wildi 2019[9]	3020/4169 (72)	60 (49-73)	Excl	APACE	2006-2015	P-need	Dx MI, 30-day
Chew 2019[50]	1646/1646 (100)	59 (49-69)	Excl	RAPID-TnT	2015-2019	Excl	30-day
Nestelberger 2019a[10]	3123/3980 (78)	61 (49-74)	Excl	APACE	2006-2015		Dx MI, 30-day
Twerenbold 2019[51]	2296 (ND)	60 (49-71)	Incl	Switzerland, Argentina	2015-2017		30-day
Amann 2019[52]	1317 (ND)	72[‖‖]	Incl	FAST-MI	2015-2016		Dx MI, 30-day
Andruchow 2020[53]	350/608 (58)	60 (52-72)	Excl	Canada, Alberta	2014-2016		Dx MI, 30-day
Cappellini 2019[54]	7176/14170 (51)	73 (59-82)	Incl	Italy, Desio	2011-2016		Dx MI
Shiozaki 2020[55]	1074/1183 (91)	72 (60-80)	Excl	Japan, Taiwan	2014-2019		30-day
Boeddinghaus 2019[11]	937/1288 (73)	60 (48-74)	Excl	APACE	2011-2015		Dx MI
Boeddinghaus 2020[14]	962/1328 (72)	ND	Excl	APACE	2011-2014		Dx MI, 30-day
Andruchow 2020[53]	350/2911 (12)	61[‖‖]	P-Inc	APPROACH	2014-2016		Dx MI, 30-day
Allen 2021[56]	1462/1583 (92)	58 (±13)	Inc	STOP CP	2017-2018		30-day
Dongxu 2021[57]	577/600 (96)	66 (59-75)	P-Inc	China	2017-2018		Dx MI, 30-day
Lopez-Ayala 2021[58]	2076/6684 (31)	63	P-Inc	APACE	APACE: 2006-2018		Dx MI, 30-day
Olsson 2021[59]	1031(ND)	61 (± 18)	Incl	Sweden	2013-2014		30-day
Ruangsomboon 2021[60]	350 (ND)	66 (±15)	P-Incl	Thailand	ND	P-need	Dx MI, 30-day
Sörensen 2021a[98]	565 (ND)	65 (52-75)	Incl	BACC	2013-2016		Dx MI
Nestelberger 2022[15]	892/6021 (15)	61 (49-74)	Excl	APACE	2006-2018		Dx MI
0 and 2h protocol							
Aldous 2011a[61]	939 (ND)	65 (56-76)	Incl	New Zealand	2007-2009		Dx MI
Reichlin 2011[42]	590/1197 (49)	64 (51-76)	Excl	APACE	2007-2009		Dx MI
Cullen 2014[20]	1571/1959 (80)	60[‖‖]	Incl	Australia/New Zealand	2007-2011		Dx MI
Parsonage 2014[††][62]	764 (ND)	55 (±15)	Incl	Australia	2008-2011		Dx MI, 30-day
Reichlin 2015b[63]	1665/2788 (60)	59[‖‖]	P-incl	APACE, Australia	APACE: 2006-2011, Australia: 2008-2011		30-day
Parsonage 2016[30]	3374/3580 (94)	61[‖‖]	P-Incl	APACE, Australia/New Zealand	Australia/New Zealand: 2008-2011; APACE: 2006-2013		30-day
Wildi 2017[23]	2525/4417 (57)	62[‖‖]	P-Incl	APACE, ADAPT	APACE: 2006-2013; ADAPT: 2007-2011	P-need	Dx MI, 30-day
McRae 2017[64]	722/7131 (10)	59 (50-69)	Excl	APPROACH	2013		Dx MI, 30-day
Borna 2018[65]	751/1165 (64)	64 (50-74)	Incl	Sweden	2013-2014	P-need	30-day
Wildi 2019[9]	2473/4169 (59)	62 (50-74)	Excl	APACE	2006-2015	P-need	Dx MI, 30-day
Lin 2019[66]	2444/2474 (99)	55 (47-64)	Excl	Singapore	2010-2013		30-day
Nestelberger 2019b[25]	2411/2979 (81)	56[‖‖]	P-Incl	APACE, ADAPT, IMPACT	APACE: ND, ADAPT: 2007-2011, IMPACT: 2011-2014		Dx MI, 30-day
Andruchow 2020[53]	550/608 (90)	60 (52-72)	Excl	Canada, Alberta	2014-2016		Dx MI, 30-day
Koechlin 2021[27]	2227/4587 (49)	61 (50-74)	Excl	APACE	ND		Dx MI
Wildi 2021[67]	2127 (ND)	61 (49-73)	Excl	APACE	ND		Dx MI
Nestelberger 2022[17]	691/6021 (11)	61 (49-74)	Excl	APACE	2006-2018		Dx MI
0 and 3h protocol							
Pickering 2016b[31]	1098 (ND)	60 (±14)	Incl	ADAPT, ADAPT-ADP, EDACS-ADP, RING	ADAPT: 2007-2011; ADAPT-ADP: 2010-2012; EDACS:	Excl	Dx MI

Author/publish year[*]	Number of patients[†]	Age[‡], year	Renal dysfunction[‖]	Study name or region	Enrolment years	Need of ECG[¶]	Clinical outcomes[**]
					2011-2014; RING: ND		
Wildi 2016[29]	2727/2922 (93)	62 (49-74)	Excl	APACE	2006-2013		Dx MI
Peacock 2018[††,68]	1679 (ND)	55 (47-64)	Incl	USA	2011-2015		Dx MI
Wildi 2019[9]	1908/4169 (46)	63 (50-76)	Excl	APACE	2006-2015	P-need	Dx MI, 30-day
Cappellini 2019[54]	7176/14170 (51)	73 (59-82)	Incl	Italy, Desio	2011-2016		Dx MI
Dupuy 2021[38]	160 (ND)	73 (±17)	Incl	Montpellier, France	ND		Dx MI
Steiro 2021[69]	932/984 (95)	63 (52-74)	Incl	WESTCOR	2015-2017	P-need	30-day
Miscellaneous							
Aldous 2011b[††,70]	332 (ND)	64 (53-74)	Incl	New Zealand	2006-2007		Dx MI
Aldous 2012[††,71]	385 (ND)	65 (56-76)	Incl	New Zealand	2007-2010		Dx MI
Irfan 2013[72]	830 (ND)	64 (51-75)	Excl	APACE	2006-2009		Dx MI
Gimenez 2014[39]	2226/2956 (75)	62 (50-75)	Excl	APACE	2006-2012		Dx MI
Biener 2015[73]	658/721 (91)	ND	Incl	Germany	ND		Dx MI
Nowak 2018[74]	569/1699 (33)	55 (49-63)	Incl	REACTION-US	2013-2015		Dx MI, 30-day
Lin 2019[66]	2444/2474 (99)	55 (47-64)	Excl	Singapore	2010-2013		Dx MI
**Troponin I assays manufactured by Siemens**							
0 and 1h protocol							
Jaeger 2016[75]	1500/1883 (80)	62 (49-74)	Excl	APACE	ND		Dx MI, 30-day
Boeddinghaus 2018b[76]	1347/1876 (72)	62 (49-75)	Excl	APACE	2006-2013		30-day
Chapman 2019[77]	1951/406 (21)	62 (±14)	Incl	High-STEACS	2013-2017	P-need	30-day
Nowak 2020[78]	2063/2470 (84)	56 (48-65)	Incl	HIGH-US	2015-2016		Dx MI, 30-day
Andersen 2021[79]	1003/1479 (68)	64 (52-74)	P-Incl	RACING-MI	2016-2019		Dx MI
Sörensen 2021b[16]	1800 (ND)	65 (52-75)	Incl	BACC	2013-2014		Dx MI
Nestelberger 2022[17]	418/6021 (7)	61 (49-74)	Excl	APACE	2006-2018		Dx MI
0 and 2h protocol							
Boeddinghaus 2018b[76]	1033/1876 (55)	62 (49-75)	Excl	APACE	2006-2013		30-day
Nestelberger 2022[17]	313/6021 (5)	61 (49-74)	Excl	APACE	2006-2018		Dx MI
0 and 3h protocol							
Wildi 2016[29]	1809/2922 (62)	62 (49-74)	Excl	APACE	2006-2013		Dx MI
Chapman 2019[77]	1920/1951 (98)	62 (±14)	Incl	High-STEACS	2013-2017	P-need	30-day
Sörensen 2021b[16]	1800 (ND)	65 (52-75)	Incl	BACC	2013-2014		Dx MI
Miscellaneous							
Irfan 2013[72]	830 (ND)	64 (51-75)	Excl	APACE	2006-2009		Dx MI
Nowak 2020[78]	1863/2470 (75)	56 (48-65)	Incl	HIGH-US	2015-2016		Dx MI, 30-day
Andersen 2021[79]	1003/1479 (68)	64 (52-74)	P-Incl	RACING-MI	2016-2019		Dx MI
**Troponin I assays manufactured by Beckman**							
0 and 1h protocol							
McCord 2021[80]	552/569 (97)	56 (±11)	Incl	REACTION-US	2013-2015		Dx MI
Nestelberger 2022[17]	278/6021 (5)	61 (49-74)	Excl	APACE	2006-2018		Dx MI
0 and 2h protocol							
Greenslade 2018[81]	1811/2314 (78)	53 (±14)	Incl	ADAPT, IMPACT	ADAPT: 2008-2011; IMPACT: 2011-2014	P-need	30-day
Nestelbergr 2019b[25]	2411/2979 (81)	56[‖‖]	P-Incl	APACE, ADAPT, IMPACT	APACE: ND, ADAPT: 2007-2011, IMPACT: 2011-2014		Dx MI, 30-day
Nestelberger 2022[17]	217/6021 (4)	61 (49-74)	Excl	APACE	2006-2018		Dx MI
0 and 3h protocol							
Wildi 2016[44]	1110/2922 (38)	62 (49-74)	Excl	APACE	2006-2013		Dx MI
Peacock 2020[29]	1049/1929 (54)	56[‖‖]	P-Incl	USA	2012-2013		Dx MI
Miscellaneous							
Irfan 2013[72]	830 (ND)	64 (51-75)	Excl	APACE	2006-2009		Dx MI
Peacock 2020[82]	1049/1929 (54)	56[‖‖]	Excl	USA	2012-2013		Dx MI
**Other assays**							
0 and 1h protocol							
Boeddinghaus 2019[11] (VITROS)	1039/1288 (81)	60 (48-74)	Excl	APACE	2011-2015		Dx MI, 30-day
Neuman 2019[12] (Singulex Clarity)	1534 (ND)	65[‖‖]	Incl	BACC	ND		Dx MI
Sörensen 2019[13] (PATHFAST)	1279 (ND)	65[‖‖]	Incl	BACC	2015-2016		Dx MI
Boeddinghaus 2020[14] (Triage True)	1084/1328 (82)	60 (47-73)	Excl	APACE	2011-2014		Dx MI, 30-day
Kavsak 2020a[83] (VITROS)	906/1366 (66)	68[‖‖]	Incl	ROMI-3	2013-2017		Dx MI, 30-day
Nestelberger 2022[17] (Triage True)	204/6021 (3)	61 (49-74)	Excl	APACE	2006-2018		Dx MI
Nestelberger 2022[17] (VITROS)	160/6021 (3)	61 (49-74)	Excl	APACE	2006-2018		Dx MI
0 and 2h protocol							

Author/publish year[*]	Number of patients[†]	Age[‡], year	Renal dysfunction[‖]	Study name or region	Enrolment years	Need of ECG[¶]	Clinical outcomes[**]
Nestelberger 2022[17] (Triage True)	204/6021 (3)	61 (49-74)	Excl	APACE	2006-2018		Dx MI
Nestelberger 2022[17] (VITROS)	160/6021 (3)	61 (49-74)	Excl	APACE	2006-2018		Dx MI
0 and 3h protocol							
Schofer 2017[84] (Singulex)	1560/1699 (92)	62 (49-74)	Incl	Germany	2007-2008		Dx MI
Kavsak 2020b[85] (VITROS)	906/1355 (66)	68[‖‖]	Incl	ROMI-3	2013-2017		Dx MI, 30-day
Dupuy 2021[38] (VITROS)	160	73 (±17)	Incl	Montpellier, France	ND		Dx MI
Miscellaneous							
Schreiber 2012[86] (Singulex)	465/486 (96)	67 (21-98)[¶¶]	Incl	USA	2005-2006		Dx MI, 30-day

ADAPT = 2-hour Accelerated Diagnostic Protocol to Assess Patients with Chest Pain Symptoms using Contemporary Troponins as the Only Biomarker; ADAPT-ADP = 2-hour Accelerated Diagnostic Protocol to Assess Patients with Chest Pain Symptoms using Contemporary Troponins as the Only Biomarker - Accelerated Diagnostic Pathway; APACE = Advantageous Predictors of Acute Coronary Syndromes Evaluation study; APPOACH = Alberta Provincial PRoject for Outcome Assessment in Coronary Heart Disease registry; ASPECT = Asia Pacific Evaluation of Chest pain Trial; BACC = Biomarkers in Acute Cardiac Care study; CKD = chronic kidney diseases; 30-day = 30-day clinical outcomes; Dx = diagnosis; EDACS-ADP = Emergency Department Assessment of Chest Pain Score - Accelerated Diagnostic Pathway; Excl = excluded; FASTEST = Fast Assessment of Thoracic pain in the Emergency department using high-Sensitive Troponins; High-STEACS = High-Sensitivity Troponin in the Evaluation of Patients With Acute Coronary Syndrome; IMPACT = Improved Assessment of Chest Pain Trial; Incl = included; IQR = interquartile range; MI = myocardial infarction; ND = no data; P-Incl = partially included; P-need = partially needed; RING = Reducing the Time Interval for Identifying New Guideline Defined Myocardial Infarction in Patients with Suspected Acute Coronary Syndrome; SD = standard deviation; TRAPID-AMI = High-sensitivity cardiac Troponin T assay for RAPID rule-out of AMI; USA = United States of America; UTROPIA = Use of Abbott High Sensitivity Troponin I Assay In Acute Coronary Syndromes, RAPID-TnT = Rapid Assessment of Possible ACS in the Emergency Department with High-Sensitivity Troponin T, ROMI-3 = Rule-out MI 3h.

*Underlined studies are representative reports with the largest sample size of unique study participants among reports based on (partially) overlapping cohorts.

†The number of analysed/enrolled patients; the number of enrolled patients is presented only if the relevant data were reported.

‡Median (interquartile range) or mean ± SD.

||Renal dysfunction refers to terminal kidney disease that requires regular haemodialysis. “Partially included” indicates a mixed study that consisted of several cohorts including patients with or without renal dysfunction.

¶Need of ECG in the assessment of participant eligibility. “Partially needed” (shown as “P-need”) indicates that at least one of the participating study sites or included cohorts needed ECG findings for assessing the participant eligibility. “Excluded” (shown as “Excl”) indicates patients with ischemia on ECG were excluded.

**Studies that assessed the diagnostic accuracy of myocardial infarction and 30-day clinical outcomes are indicated as Dx MI and 30-day, respectively.

††A study that included patients suspected of having ST-elevation MI in addition to patients suspected of having non-ST elevation MI.

‡‡Normal renal function.

§§Renal dysfunction.

||||Weighted average.

¶¶Range.

Appendix Table 2. Excluded articles and reasons for exclusion.

Authors	Reasons
Agewall 2007[87]	Only patients admitted in the coronary care units
Ahmadi 2017[88]	Only patients with positive hs-cTn
Alcalai 2021[89]	Single, baseline hs-cTn only
Aldous 2011[90]	Hs-cTn assessed at baseline only
Aldous 2012a[91]	No accuracy data
Aldous 2012c[92]	No accuracy data
Alghamdi 2020[93]	Use of a non-hs-cTn
Al–Saleh 2014[94]	Review article
Alushi 2021[95]	Irrelevant clinical context
Anand 2021[96]	Irrelevant clinical context
Apple 2006[97]	Use of a non-hs-cTn
Apple 2009[98]	Use of a non-hs-cTn
Astley 2014[99]	Study protocol
Avest 2016[100]	Single, baseline hs-cTn only
Bahrmann 2012[101]	Irrelevant clinical context
Bahrmann 2013[102]	Irrelevant clinical context
Bahrmann 2016[103]	Irrelevant
Bali 2021[104]	Use of a non-hs-cTn
Ballocca 2017[105]	Patients with positive hs-cTn only
Bandstein 2014[106]	Single, baseline hs-cTn only
Bandstein 2016[107]	Irrelevant clinical context
Bandstein 2017[108]	Single, baseline hs-cTn only
Bellini 2021[109]	Irrelevant clinical context
Bhardwaj 2011[110]	Single, baseline hs-cTn only
Biener 2013[111]	Irrelevant clinical context
Birkhahn 2011[112]	No accuracy data
Bjurman 2013[113]	Only patients admitted in the coronary care units
Body 2011[114]	Single, baseline hs-cTn only
Body 2015a[115]	Single, baseline hs-cTn only
Body 2015b[116]	Single, baseline hs-cTn only
Body 2016[117]	Single, baseline hs-cTn only
Body 2020[118]	Single, baseline hs-cTn only
Boeckel 2015[119]	Use of a non-hs-cTn.
Boeddinghaus 2017[120]	Patients with positive hs-cTn only
Bonaca 2013[121]	Single, baseline hs-cTn only
Borna 2014[122]	Only patients admitted in the coronary care units

Borna 2016[123]	Only patients admitted in the hospitals
Bove 2017[124]	Only patients admitted in the hospitals
Braga 2011[125]	No accuracy data
Brichko 2018[126]	Patients with negative hs-cTn only
Bularga 2019 [127]	Single, baseline hs-cTn only
Caglar 2020[128]	Irrelevant clinical context
Carlton 2016[129]	Single, baseline hs-cTn only
Carlton 2016[130]	Irrelevant
Carlton 2018[131]	Irrelevant
Carlton 2020 [132]	Single, baseline hs-cTn only
Cavender 2017[133]	Irrelevant
Celik 2011[134]	Case series of patients with ACS
Chapman 2017[135]	Review article
Charpentier 2016 [136]	Guidelines
Chenevier–Gobeaux 2016[137]	Irrelevant clinical context
Chew 2016[138]	Irrelevant
Chew 2018[139]	Single, baseline hs-cTn only
Christ 2010[140]	Single, baseline hs-cTn only
Christ 2018[141]	Use of a non-hs-cTn
Christenson 2020[142]	No accuracy data
Chuang 2016[143]	Irrelevant clinical context
Clerico 2019[144]	Irrelevant clinical context
Colak 2017[145]	Article not obtainable
Collinson 2006a[146]	Single, baseline hs-cTn only
Collinson 2006b[147]	Single, baseline hs-cTn only
Collinson 2015[148]	No accuracy data
Conde 2013[149]	No accuracy data
Cook 2021[150]	Single, baseline hs-cTn only
Corsini 2015[151]	No accuracy data
Cramer 2007[152]	Use of a non-hs-cTn
Cullen 2013[153]	Use of a non-hs-cTn
Cullen 2016[154]	Irrelevant
Dadkhah 2017[155]	Irrelevant
Daly 2012[156]	Patients submitted to the catheter laboratory only
Davarani 2012[157]	Use of a non-hs-cTn
Dawson 2013[158]	Irrelevant clinical context
Diercks 2012[159]	Use of a non-hs-cTn
Druey 2015[160]	Use of a non-hs-cTn

Eggers 2011[161]	Use of a non-hs-cTn
Etaher 2021[162]	Irrelevant clinical context
Fan 2014[163]	No accuracy data
Fanaroff 2015[164]	Irrelevant
Freund 2011[165]	Single, baseline hs-cTn only
Frisoli 2017[166]	Irrelevant
Gassenmaier 2012[167]	Irrelevant clinical context
Giannitsis 2010[168]	Only patients admitted in the chest pain unit
Giannitsis 2010a[169]	Only patients with confirmed NSTE-ACS
Gimenez 2016[170]	Single, baseline hs-cTn only and no accuracy data
Gimenez 2020[171]	Irrelevant clinical context
Gimenez 2021[171]	Irrelevant clinical context
Giner–Caro 2013[172]	Letter
Goodman 2015[173]	Irrelevant clinical context
Gratzel 2015[174]	No accuracy data
Graven 2021[175]	Irrelevant clinical context
Greenslade 2018[176]	Single, baseline hs-cTn only
Grinstein 2015[177]	Only patients with NSTEMI
Haaf 2014[178]	Single, baseline hs-cTn only
Haider 2017[179]	Single, baseline hs-cTn only
Halim 2009[180]	Single, baseline hs-cTn only
Hall 2012[181]	No accuracy data
Hochholzer 2011[182]	Single, baseline hs-cTn only
Hochholzer 2014[183]	Patients submitted to the catheter laboratory only
Hochholzer 2016[184]	Guidelines
Hoeller 2013[185]	Single, baseline hs-cTn only
Hromadka 2012[186]	Irrelevant clinical context
Huang 2015[187]	Single, baseline hs-cTn only
Huang 2016[188]	Single, baseline hs-cTn only
Ilva 2009[189]	Single, baseline hs-cTn only
Invernizzi 2013[190]	Review article
Isiksacan 2017[191]	Single, baseline hs-cTn only
Iwasaki 2017[192]	Use of a non-hs-cTn
Jairam 2011[193]	Irrelevant clinical context
Johannessen 2020[194]	Irrelevant clinical context
Karady 2021[195]	Use of a non-hs-cTn
Karon 2018[196]	Irrelevant clinical context

Kavsak 2006[197]	Use of a non-hs-cTn
Kavsak 2009[198]	Single, baseline hs-cTn only
Kavsak 2012[199]	Single, baseline hs-cTn only
Kavsak 2017[200]	Single, baseline hs-cTn only
Kavsak 2018a[201]	Single, baseline hs-cTn only
Kavsak 2018b[202]	Single, baseline hs-cTn only
Kavsak 2020[203]	Single, baseline hs-cTn only
Kelly 2011[204]	Single, baseline hs-cTn only
Kelly 2014a[205]	Patients with negative hs-cTn only
Kelly 2014b[206]	Irrelevant
Kelly 2015[207]	Irrelevant
Kemper 2017[208]	Use of a non-hs-cTn
Khan 2021[209]	Irrelevant clinical cotext
Khoshnood 2020[210]	Single, baseline hs-cTn only
Kienbacher 2021[211]	No accuracy data
Kim 2013[212]	Irrelevant
Kimenai 2020[213]	Only patients admitted in the coronary care units
Kitamura 2013[214]	Single, baseline hs-cTn only
Kremneva 2016[215]	Use of a non-hs-cTn.
Krintus 2014[216]	Irrelevant
Kumar 2016[217]	Two-gate design (patients with MI and healthy subjects)
Lambrakis 2021[218]	Irrelevant clinical context
Lee 2010[219]	No accuracy data
Lehmacher 2020[220]	Irrelevant clinical context
Li 2015[221]	Meta-analysis.
Lim 2008[222]	Irrelevant
Lim 2020[194]	Single, baseline hs-cTn only
Lin 2012[223]	Review article.
Lindahl 2010[224]	Patients with ACS only
Love 2016[225]	Irrelevant clinical context
Lynn 2011[226]	Irrelevant clinical context
Maag 2015[227]	Irrelevant clinical context
MacRae 2006[228]	No accuracy data
Madsen 2015[229]	Only patients with positive hs-cTn
Mahler 2013[230]	Irrelevant
Marjot 2017[231]	Single, baseline hs-cTn only
Markota 2011[232]	Use of a non-hs-cTn.
Masotti 2018[233]	Irrelevant

May 2014[234]	Single, baseline hs-cTn only
McRae 2015[235]	Commentary
McRae 2017[64]	Single, baseline hs-cTn only
Melki 2011[236]	Only patients admitted in the coronary care units
Melki 2012[237]	Irrelevant
Melki 2015[238]	Irrelevant
Meune 2011[239]	Single, baseline hs-cTn only
Meune 2012[240]	Irrelevant
Miller 2008[241]	Irrelevant clinical context
Mills 2011[242]	Irrelevant
Mills 2012[243]	Single, baseline hs-cTn only
Moal 2007[244]	Irrelevant
Mohsen 2016[245]	No accuracy data
Mokhtari 2016[246]	Single, baseline hs-cTn only
Moliner 2020[247]	No accuracy data
Mueller 2012a[248]	Single, baseline hs-cTn only
Mueller 2012b[249]	Irrelevant clinical context
Mueller 2013[250]	Review article.
Mueller 2014[251]	Irrelevant clinical context
Mueller–Hennessen 2017[252]	Only patients with positive hs-cTn
Mungai 2020[253]	Irrelevant clinical context
Nacke 2014[254]	Only patients with ACS
Nadarajah 2021[255]	Clinical guidance
Narain 2008[256]	Only patients with ACS
Nejatian 2017[257]	Irrelevant clinical context
Nestelberger 2016[258]	No accuracy data
Nestelberger 2021a[259]	Irrelevant clinical context
Nestelberger 2021b[260]	Irrelevant clinical context
Neumann 2017[261]	Single, baseline hs-cTn only
Nilsson 2021[262]	Irrelevant clinical context
Normann 2012[263]	Irrelevant clinical context
Nowak 2021[264]	No accuracy data
Olivieri 2012[265]	No accuracy data
Omland 2011[266]	Review article
Osredkar 2021[267]	Use of non-hs-cTn
Palamalai 2013[268]	Use of non-hs-cTn
Paoloni 2010[269]	No accuracy data
Papendick 2017[270]	Study protocol

Parikh 2015[271]	Meta-analysis
Patsias 2017[272]	Only patients with positive hs-cTn
Peck 2016[273]	Irrelevant clinical context
Pickering 2018[274]	Irrelevant clinical context
Pickering 2020[275]	Irrelevant clinical context
Puelacher 2019[276]	Irrelevant clinical context
Rao 2012[277]	Irrelevant
Reichlin 2009[278]	Single, baseline hs-cTn only
Reichlin 2013[279]	Case series
Reiter 2011[280]	Review article
Ren 2021[281]	Single, baseline hs-cTn only
Renaud 2008[282]	Use of non-hs-cTn.
Roberts 2014[283]	Not a primary study
Roos 2017a[284]	Irrelevant clinical context
Roos 2017b[285]	Irrelevant clinical context
Rottger 2017[286]	Irrelevant clinical context
Rubini 2013[287]	No accuracy data
Rudolph 2012[288]	Irrelevant
Saad 2015a[289]	Single, baseline hs-cTn only
Saad 2015b[289]	Single, baseline hs-cTn only
Saenger 2010[290]	Conference abstract.
Saenger 2011[291]	Irrelevant
Sanchis 2012[292]	Only patients with positive hs-cTn
Sanchis 2014[293]	Irrelevant
Sanchis 2016[294]	Irrelevant
Sandoval 2015[295]	No accuracy data
Sandoval 2017[296]	Single, baseline hs-cTn only
Sandoval 2017[297]	Single, baseline hs-cTn only
Sandoval 2020[37]	Irrelevant clinical context
Santalo 2013[298]	Multi-gate design of case-mix
Schonemann–Lund 2015[299]	Irrelevant clinical context
Shah 2015[300]	Single, baseline hs-cTn only
Shah 2015 [301]	Single, baseline hs-cTn only
Shin 2020[302]	Irrelevant clinical context
Shortt 2015[303]	Single, baseline hs-cTn only
Shortt 2017[304]	Single, baseline hs-cTn only
Simpson 2014[305]	Irrelevant clinical context
Singer 2017[306]	Use of non-hs-cTn

Slagman 2017[307]	Use of non-hs-cTn
Sorensen 2017[308]	Irrelevant
Sorensen 2019[309]	Single, baseline hs-cTn only
Stengaard 2013[310]	Use of non-hs-cTn
Stopyra 2016[311]	Irrelevant
Stopyra 2020[312]	Irrelevant clinical context
Stoyanov 2020[313]	Irrelevant clinical context
Storrow 2015a[314]	Use of non-hs-cTn
Storrow 2015b[315]	Use of non-hs-cTn
Straface 2008[316]	Use of non-hs-cTn
Su 2015[317]	Irrelevant clinical context
Suh 2018[318]	Use of non-hs-cTn
Suzuki 2018[319]	Use of non-hs-cTn
Takakuwa 2009[320]	Use of non-hs-cTn
Tecson 2017[321]	Use of non-hs-cTn
Than 2011[322]	Use of non-hs-cTn
Than 2012[323]	Use of non-hs-cTn
Than 2014[324]	Irrelevant
Thelin 2015[325]	No accuracy data
Truong 2012[326]	Irrelevant
Twerenbold 2015[327]	Single, baseline hs-cTn only
Vafaie 2014[328]	Educational article
van der Laarse 2013[329]	Use of non-hs-cTn.
Van Hise 2018[330]	Irrelevant
Vasile 2017[331]	Review article
Vasudevan 2017[332]	Use of non-hs-cTn.
Venge 2009a[333]	Two–gate design study
Venge 2009b[334]	Irrelevant clinical context
Wang 2015a[335]	Only patients with COPD
Wang 2015b[336]	Two-gate design study (patients with AMI and healthy subjects)
Wassef 2015a[337]	Only patients admitted in the coronary care units
Wassef 2015b[337]	Only patients admitted in the Cardiology department
Wassie 2020[338]	Use of non-hs-cTn
Weber 2011[339]	Irrelevant clinical context
Wereski 2021[340]	Irrelevant clinical context
Westwood 2015[341]	Review article
Wildi 2013[342]	Patients with positive hs-cTn were excluded.
Willeit 2017[343]	Meta-analysis

Wolf 2014[344]	Patients with ACS only
Wong 2010[345]	Irrelevant clinical context
Yang 2021[346]	Irrelevant clinical context
Yean 2020[347]	Irrelevant clinical context
Yokoyama 2018[348]	Irrelevant clinical context
Zhao 2021[349]	Irrelevant clinical context

AMI = acute myocardial infarction; ACS = acute coronary syndrome; COPD = chronic obstructive pulmonary disease; hs–cTn = high sensitivity cardiac troponin, NSTE-ACS = non-ST-elevation acute coronary syndrome

Appendix Table 3. Institution and geographic characteristics of included study cohorts.

Cohort name		Europe		Australasia		North America		Asia	
(N of participants)	Institutions	City	Country	City	Country	City	Country	City	Country
ADAPT[323] (1635)[***]	Christchurch Hospital			Christchurch	New Zealand				
	Royal Brisbane and Women's Hospital			Brisbane	Australia				
ADAPT-ADP[324] (406)[***]	Christchurch Hospital			Christchurch	New Zealand				
APACE[350] (4368) [***]	Hospital Clinic Barcelona	Barcelona	Spain						
	Hospital Clinic San Carlos	Madrid	Spain						
	Hospital del Mar	Barcelona	Spain						
	Kantonsspital Baselland, Standort Bruderholz	Bottmingen	Switzerland						
	Kantonsspital Baselland, Standort Liestal	Liestal	Switzerland						
	Kantonsspital Olten	Olten	Switzerland						
	Klinik St. Anna	Luzern	Switzerland						
	Masaryk University	Brno	Czech						
	Medical University of Silesia	Silesia	Poland						
	San Martino Hospital	San Martino	Italy						
	Spital Limmattal	Schlieren	Switzerland						
	University Hospital of Basal	Basel	Switzerland						
	University Hospital of Zurich	Zurich	Switzerland						
APPROACH[351] (722)	University of Calgary					Calgary	Canada		
	University of Alberta					Edomonton	Canada		

Cohort name		Europe		Australasia		North America		Asia	
(N of participants)	Institutions	City	Country	City	Country	City	Country	City	Country
ASPECT[322] (ND)	Alberta Health Services					Calgary	Canada		
	Apollo Hospital							Delhi	India
	Chang Gung Memorial Hospital							Taipei	Taiwan
	China Medl University Hospital							Taipei	Taiwan
	Christchurch Hospital			Christchurch	New Zealand				
	Far Eastern Memorial Hospital							Taipei	Taiwan
	Mackay Memorial Hospital							Taipei	Taiwan
	National Cardiovascular Centre Harapan Kita							Jakarta	Indonesia
	Peoples' Hospital							Beijing	China
	Queen Elizabeth Hospital							Hong Kong	China
	Ramathibodi Hospital							Bangkok	Thailand
	Royal Brisbane and Women's Hospital			Brisbane	Australia				
	Severance Hospital							Seoul	Korea
	Singapore General Hospital							Singapore	Singapore
	Xuanwu Hospital							Beijing	China
BACC[352] (3500)[***]	University Heart Center Hamburg	Hamburg	Germany						
EDACS-ADP[353] (128)[***]	Christchurch Hospital			Christchurch	New Zealand				

Cohort name		Europe		Australasia		North America		Asia	
(N of participants)	Institutions	City	Country	City	Country	City	Country	City	Country
FASTEST[24] (605)	Uppsala University	Uppsala	Sweden						
	Karolinska Institute	Huddinge	Sweden						
	Karolinska University Hospital	Stockholm	Sweden						
FAST-MI[52] (1317)	University Heart Center Freiburg	Bad Krozingen	Germany						
Hamburg[28] (1818)	Federal Armed Forces Hospital Koblenz	Hamburg	Germany						
	Johannes Gutenberg-University Mainz	Hamburg	Germany						
	University Hospital Hamburg Eppendorf	Hamburg	Germany						
High STEACS[354] (1886)	Royal Infirmary of Edinburgh	Edinburgh	UK						
HIGH-US [78] (2346)	Baylor College of Medicine					Houston	USA		
	Beth Israel Deaconess					Boston	USA		
	Brigham and Women’s					Boston	USA		
	Carolinas Medical Center					Charlotte	USA		
	Case Medical Center					Cleveland	USA		
	Detroit Receiving Wayne State					Detroit	USA		
	Duke University					Durham	USA		
	Hackensack University					Hackensack	USA		
	Hennepin County Medical Center					Minneapolis	USA		
	Henry Ford Hospital					Detroit	USA		
	Indiana University					Indianapolis	USA		
	Mayo Clinic					Rochester	USA		

Cohort name		Europe		Australasia		North America		Asia	
(N of participants)	Institutions	City	Country	City	Country	City	Country	City	Country
	New York Methodist					Brooklyn	USA		
	Research & Development Institute, Van Nuys; Providence Tarzana Medical Center					Tarzana	USA		
	Sinai Grace Wayne State					Detroit	USA		
	St. Vincent Hospital					Worcester	USA		
	Stony Brooks					Stony Brooks	USA		
	Tampa General Hopital					Tampa	USA		
	The Miriam hospital					Providence	USA		
	Thomas Jefferson University					Philadelphia	USA		
	UC Davis					Davis	USA		
	UC San Francisco					San Francisco	USA USA		
	University of Cincinnati					Cincinnati	USA		
	University of Maryland					Baltimore	USA		
	University of New Mexico					Albuquerque	USA		
	University of Texas Medical Branch					Galveston	USA		
	University of Virginia					Charlottesvill e	USA		
	Virginia Common Health University					Richmond	USA		
	Wake Forrest University					Winston-Salem	USA		
IMPACT[355] (ND)	Royal Brisbane and Women's Hospital			Brisbane	Australia				
Italy[54] (6403)	Desio Hospital	Desio	Italy						

Cohort name		Europe		Australasia		North America		Asia	
(N of participants)	Institutions	City	Country	City	Country	City	Country	City	Country
Juntendo[48] (1074)***	Juntendo University Hospital							Tokyo	Japan
RACING-MI[79] (1003)	Randers Regional Hospital	Randers	Denmark						
RAPID-TnT [50] (1646)	Flinders Medical Centre - Bedford Park			Adelaide	Australia				
	The Royal Adelaide Hospital -			Adelaide	Australia				
	Noarlunga Health Service - Noarlunga Centre			Adelaide	Australia				
	Lyell McEwin Hospital - Elizabeth Vale			Adelaide	Australia				
	The Queen Elizabeth Hospital -			Woodville	Australia				
REACTION-US[80] (552)	Henry Ford Hopital					Detroit	USA		
RING[356] (148)***	McMaster University					Hamilton	Canada		
ROMI-3[83] (935)***	McMaster University					Hamilton	Canada		
Shanghai[57] (577)	Zhongshan Hospital							Shanghai	China
Singapore[66] (2444)	Singapore General Hospital								Singapore
Skåne[45 65] (1038)***	Skåne University Hospital	Lund	Sweden						
STOP CP[56] (1642)	UC Davis					Sacramento	USA		

Cohort name		Europe		Australasia		North America		Asia	
(N of participants)	Institutions	City	Country	City	Country	City	Country	City	Country
	University of Florida					Gainesville	USA		
	University of Maryland					Baltimore	USA		
	Henry Ford Health System					Detroit	USA		
	Wake Forest University					Winston-Salem	USA		
	University of Utah					Salt Lake City	USA		
TRAPID-AMI[47] (1282)[***]	Central Manchester U niversity Hospital	Manchester	UK						
	Cliniques University Saint-Luc (UCLouvain)	Brussel	Belgium						
	Henry Ford Health System					Detroit	USA		
	Institut d'Investigacions Biomèdiques Sant Pau	Barcelona	Spain						
	Karolinska Institute	Huddinge	Sweden						
	Liverpool Hosp ital and University New South Wales			Liverpool	Australia				
	Nuremberg Paracelsus Medical University	Nuremberg	Germany						
	University Hospital of Basal	Basel	Switzerland						
	Heidelberg University Hospital	Heidelberg	Germany						
	University Maryland					Baltimore	USA		
	Uppsala University	Uppsala	Sweden						
US multicenter[68] (1679)	Charleston Area Medical Center					Charleston	USA		
	Cleveland Clinic Foundation					Cleveland	USA		

Cohort name		Europe		Australasia		North America		Asia	
(N of participants)	Institutions	City	Country	City	Country	City	Country	City	Country
	Cooper University Hospital					Camden	USA		
	Duke University					Durham	USA		
	Health Science Center of Houston Medical School					Houston	USA		
	Henry Ford Health System					Detroit	USA		
	Indianapolis University					Indianapolis	USA		
	Mayo Clinic					Rochester	USA		
	Minneapolis Heart Institute Foundation					Minneapolis	USA		
	Newton Wellesley Hospital					Newton	USA		
	Prince George's Hospital					Maryland	USA		
	San Francisco General Hospital					San Francisco	USA		
	South Shore Hospital					Weymouth	USA		
	Thomas Jefferson University Hospital					Philadelphia	USA		
	University North Carolina					Chapel Hill	USA		
UTROPIA[357] (1631)	Hennepin County Medical Center					Minneapolis	USA		
WESTCOR[69] (932)	Haukeland University Hospital	Bergen	Norway						

ADAPT = 2-hour Accelerated Diagnostic Protocol to Assess Patients with Chest Pain Symptoms using Contemporary Troponins as the Only Biomarker; ADAPT–ADP = 2-hour Accelerated Diagnostic Protocol to Assess Patients with Chest Pain Symptoms using Contemporary Troponins as the Only Biomarker - Accelerated Diagnostic Pathway; AMI = acute myocardial infarction; APACE = Advantageous Predictors of Acute Coronary Syndromes Evaluation study; APPOACH = Alberta Provincial PRoject for Outcome Assessment in Coronary Heart Disease registry; ASPECT = Asia Pacific Evaluation of Chest pain Trial; BACC = Biomarkers in Acute Cardiac Care study; EDACS–ADP = Emergency Department Assessment of Chest Pain Score – Accelerated Diagnostic Pathway; FASTEST = Fast ASsessment of Thoracic pain in the Emergency department using high-Sensitive Troponins; High-STEACS = High-Sensitivity Troponin in the Evaluation of Patients

With Acute Coronary Syndrome; IMPACT = Improved Assessment of Chest Pain Trial; IQR = interquartile range; RACING-MI= Rapid use of high-sensitive cardiac troponin I for ruling-in and ruling-out of acute myocardial infarction; REACTION-US= Rapid Evaluation of ACuTe Myocardial InfarctiON in the US; RING = Reducing the Time Interval for Identifying New Guideline Defined Myocardial Infarction in Patients with Suspected Acute Coronary Syndrome; TRAPID–AMI = High-sensitivity cardiac Troponin T assay for RAPID rule-out of AMI; UTROPIA = Use of Abbott High Sensitivity Troponin I Assay In Acute Coronary Syndromes

***The largest number represented when multiple publications were derived from the same study cohort.

Appendix Figure 1. Geographical locations of eligible studies*

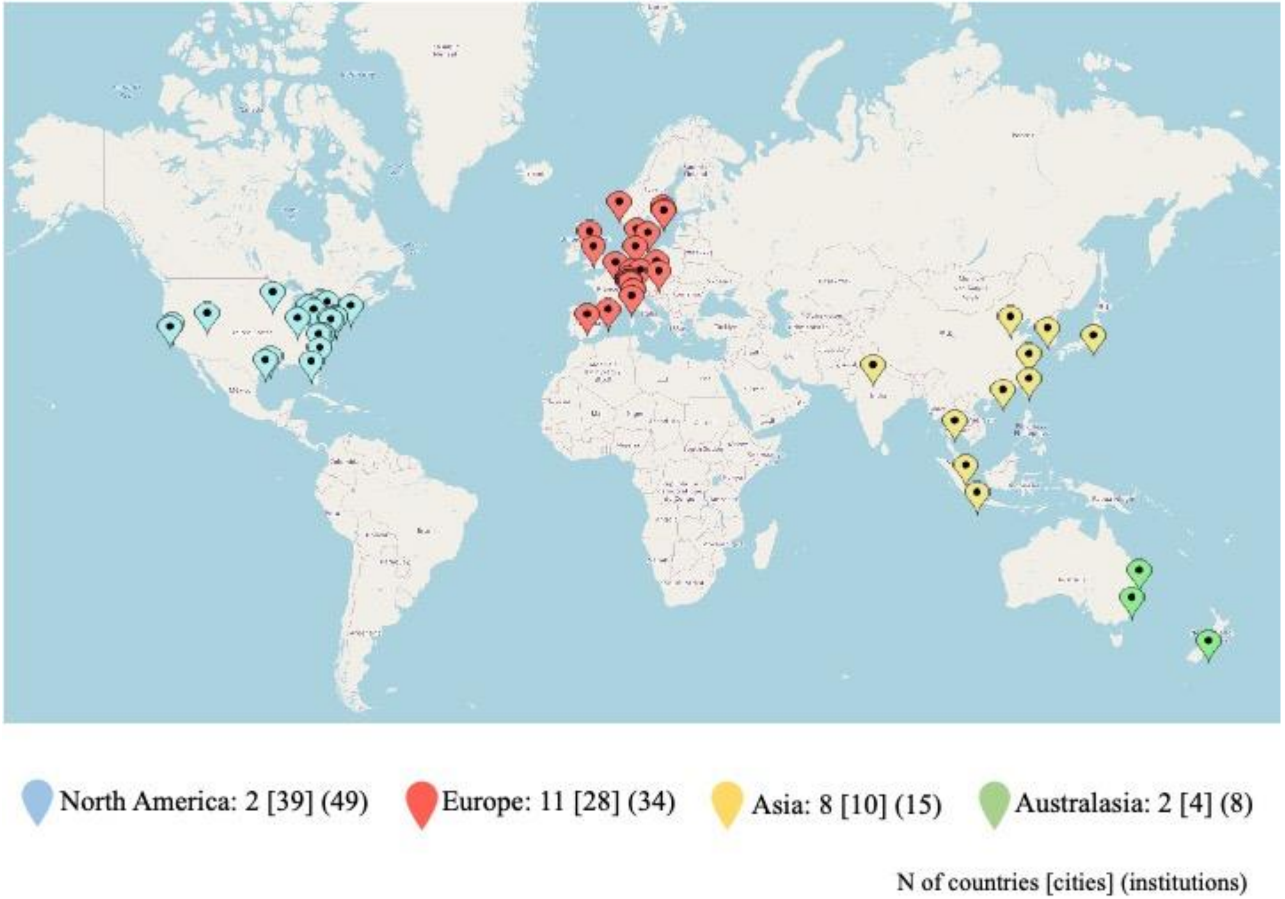

*Non-overlapping geographical regions (city as the unit) covered by the included research institution(s) are plotted. See Appendix Table 2 for details.

References

1. Rubini Gimenez M, Twerenbold R, Jaeger C, et al. One-hour rule-in and rule-out of acute myocardial infarction using high-sensitivity cardiac troponin I. *Am J Med* 2015;128(8):861-70 e4. doi: 10.1016/j.amjmed.2015.01.046
2. Pickering JW, Greenslade JH, Cullen L, et al. Assessment of the European Society of Cardiology 0-Hour/1-Hour Algorithm to Rule-Out and Rule-In Acute Myocardial Infarction. *Circulation* 2016;134(20):1532-41. doi: 10.1161/CIRCULATIONAHA.116.022677
3. Neumann JT, Sorensen NA, Schwemer T, et al. Diagnosis of Myocardial Infarction Using a High-Sensitivity Troponin I 1-Hour Algorithm. *JAMA Cardiol* 2016;1(4):397-404. doi: 10.1001/jamacardio.2016.0695
4. Boeddinghaus J, Nestelberger T, Twerenbold R, et al. Direct Comparison of 4 Very Early Rule-Out Strategies for Acute Myocardial Infarction Using High-Sensitivity Cardiac Troponin I. *Circulation* 2017;135(17):1597-611. doi: 10.1161/CIRCULATIONAHA.116.025661
5. Neumann JT, Sorensen NA, Ojeda F, et al. Early diagnosis of acute myocardial infarction using high-sensitivity troponin I. *PLoS One* 2017;12(3):e0174288. doi: 10.1371/journal.pone.0174288
6. Twerenbold R, Badertscher P, Boeddinghaus J, et al. 0/1-Hour Triage Algorithm for Myocardial Infarction in Patients With Renal Dysfunction. *Circulation* 2018;137(5):436-51. doi: 10.1161/CIRCULATIONAHA.117.028901
7. Twerenbold R, Neumann JT, Sorensen NA, et al. Prospective Validation of the 0/1-h Algorithm for Early Diagnosis of Myocardial Infarction. *J Am Coll Cardiol* 2018;72(6):620-32. doi: 10.1016/j.jacc.2018.05.040
8. Boeddinghaus J, Nestelberger T, Twerenbold R, et al. Impact of age on the performance of the ESC 0/1h-algorithms for early diagnosis of myocardial infarction. *Eur Heart J* 2018;39(42):3780-94. doi: 10.1093/eurheartj/ehy514
9. Wildi K, Boeddinghaus J, Nestelberger T, et al. Comparison of fourteen rule-out strategies for acute myocardial infarction. *Int J Cardiol* 2019;283:41-47. doi: 10.1016/j.ijcard.2018.11.140
10. Nestelberger T, Boeddinghaus J, Wussler D, et al. Predicting Major Adverse Events in Patients With Acute Myocardial Infarction. *J Am Coll Cardiol* 2019;74(7):842-54. doi: 10.1016/j.jacc.2019.06.025
11. Boeddinghaus J, Twerenbold R, Nestelberger T, et al. Clinical Use of a New High-Sensitivity Cardiac Troponin I Assay in Patients with Suspected Myocardial Infarction. *Clin Chem* 2019;65(11):1426-36. doi: 10.1373/clinchem.2019.304725
12. Neumann JT, Sorensen NA, Rubsamen N, et al. Evaluation of a new ultra-sensitivity troponin I assay in patients with suspected myocardial infarction. *Int J Cardiol* 2019;283:35-40. doi: 10.1016/j.ijcard.2018.12.001
13. Sorensen NA, Neumann JT, Ojeda F, et al. Diagnostic Evaluation of a High-Sensitivity Troponin I Point-of-Care Assay. *Clin Chem* 2019;65(12):1592-601. doi: 10.1373/clinchem.2019.307405
14. Boeddinghaus J, Nestelberger T, Koechlin L, et al. Early Diagnosis of Myocardial Infarction With Point-of-Care High-Sensitivity Cardiac Troponin I. *J Am Coll Cardiol* 2020;75(10):1111-24. doi: 10.1016/j.jacc.2019.12.065
15. Sörensen NA, Neumann JT, Ojeda F, et al. Differences in measurement of high-sensitivity troponin in an on-demand and batch-wise setting. *European Heart Journal Acute Cardiovascular Care* 2021;10(3):302-09. doi: 10.1177/2048872620924198
16. Sorensen NA, Gossling A, Neumann JT, et al. Diagnostic Validation of a High-Sensitivity Cardiac Troponin I Assay. *Clin Chem* 2021;67(9):1230-39. doi: 10.1093/clinchem/hvab070
17. Nestelberger T, Boeddinghaus J, Gimenez MR, et al. Direct comparison of high-sensitivity cardiac troponin T and I in

the early differentiation of type 1 vs. type 2 myocardial infarction. *Eur Heart J Acute Cardiovasc Care* 2022;11(1):62-74. doi: 10.1093/ehjacc/zuab039

18. Cullen L, Mueller C, Parsonage WA, et al. Validation of high-sensitivity troponin I in a 2-hour diagnostic strategy to assess 30-day outcomes in emergency department patients with possible acute coronary syndrome. *J Am Coll Cardiol* 2013;62(14):1242-49. doi: 10.1016/j.jacc.2013.02.078
19. Aldous S, Mark Richards A, George PM, et al. Comparison of new point-of-care troponin assay with high sensitivity troponin in diagnosing myocardial infarction. *Int J Cardiol* 2014;177(1):182-6. doi: 10.1016/j.ijcard.2014.09.026
20. Cullen L, Aldous S, Than M, et al. Comparison of high sensitivity troponin T and I assays in the diagnosis of non-ST elevation acute myocardial infarction in emergency patients with chest pain. *Clin Biochem* 2014;47(6):321-6. doi: 10.1016/j.clinbiochem.2013.11.019
21. Eggers KM, Aldous S, Greenslade JH, et al. Two-hour diagnostic algorithms for early assessment of patients with acute chest pain--Implications of lowering the cardiac troponin I cut-off to the 97.5th percentile. *Clin Chim Acta* 2015;445:19-24. doi: 10.1016/j.cca.2015.03.002
22. Boeddinghaus J, Reichlin T, Cullen L, et al. Two-Hour Algorithm for Triage toward Rule-Out and Rule-In of Acute Myocardial Infarction by Use of High-Sensitivity Cardiac Troponin I. *Clin Chem* 2016;62(3):494-504. doi: 10.1373/clinchem.2015.249508
23. Wildi K, Cullen L, Twerenbold R, et al. Direct Comparison of 2 Rule-Out Strategies for Acute Myocardial Infarction: 2-h Accelerated Diagnostic Protocol vs 2-h Algorithm. *Clin Chem* 2017;63(7):1227-36. doi: 10.1373/clinchem.2016.268359
24. Lindahl B, Jernberg T, Badertscher P, et al. An algorithm for rule-in and rule-out of acute myocardial infarction using a novel troponin I assay. *Heart* 2017;103(2):125-31. doi: 10.1136/heartjnl-2016-309951
25. Nestelberger T, Boeddinghaus J, Greenslade J, et al. Two-Hour Algorithm for Rapid Triage of Suspected Acute Myocardial Infarction Using a High-Sensitivity Cardiac Troponin I Assay. *Clin Chem* 2019;65(11):1437-47. doi: 10.1373/clinchem.2019.305193
26. Kavsak PA, Hewitt MK, Mondoux SE, et al. Diagnostic Performance of Serial High-Sensitivity Cardiac Troponin Measurements in the Emergency Setting. *J Cardiovasc Dev Dis* 2021;8(8) doi: 10.3390/jcdd8080097
27. Koechlin L, Boeddinghaus J, Nestelberger T, et al. Performance of the ESC 0/2h-algorithm using high-sensitivity cardiac troponin I in the early diagnosis of myocardial infarction. *Am Heart J* 2021;242:132-37. doi: 10.1016/j.ahj.2021.08.008
28. Keller T, Zeller T, Ojeda F, et al. Serial Changes in Highly Sensitive Troponin I Assay and Early Diagnosis of Myocardial Infarction. *JAMA* 2011;306(24):2684-93. doi: 10.1001/jama.2011.1896
29. Wildi K, Nelles B, Twerenbold R, et al. Safety and efficacy of the 0 h/3 h protocol for rapid rule out of myocardial infarction. *Am Heart J* 2016;181:16-25. doi: 10.1016/j.ahj.2016.07.013
30. Parsonage WA, Mueller C, Greenslade JH, et al. Validation of NICE diagnostic guidance for rule out of myocardial infarction using high-sensitivity troponin tests. *Heart* 2016;102(16):1279-86. doi: 10.1136/heartjnl-2016-309270
31. Pickering JW, Greenslade JH, Cullen L, et al. Validation of presentation and 3 h high-sensitivity troponin to rule-in and rule-out acute myocardial infarction. *Heart* 2016;102(16):1270-8. doi: 10.1136/heartjnl-2015-308505
32. Chapman AR, Anand A, Boeddinghaus J, et al. Comparison of the Efficacy and Safety of Early Rule-Out Pathways for

Acute Myocardial Infarction. *Circulation* 2017;135(17):1586-96. doi: 10.1161/CIRCULATIONAHA.116.025021

33. Sandoval Y, Smith SW, Thordsen SE, et al. Diagnostic Performance of High Sensitivity Compared with Contemporary Cardiac Troponin I for the Diagnosis of Acute Myocardial Infarction. *Clin Chem* 2017;63(10):1594-604. doi: 10.1373/clinchem.2017.272930
34. Chapman AR, Hesse K, Andrews J, et al. High-Sensitivity Cardiac Troponin I and Clinical Risk Scores in Patients With Suspected Acute Coronary Syndrome. *Circulation* 2018;138(16):1654-65. doi: 10.1161/CIRCULATIONAHA.118.036426
35. Gunsolus I, Sandoval Y, Smith SW, et al. Renal Dysfunction Influences the Diagnostic and Prognostic Performance of High-Sensitivity Cardiac Troponin I. *J Am Soc Nephrol* 2018;29(2):636-43. doi: 10.1681/ASN.2017030341
36. Kim JW, Kim H, Yun YM, et al. Absolute Change in High-Sensitivity Cardiac Troponin I at Three Hours After Presentation is Useful for Diagnosing Acute Myocardial Infarction in the Emergency Department. *Ann Lab Med* 2020;40(6):474-80. doi: 10.3343/alm.2020.40.6.474
37. Sandoval Y, Smith SW, Schulz K, et al. Comparison of 0/3-Hour Rapid Rule-Out Strategies Using High-Sensitivity Cardiac Troponin I in a US Emergency Department. *Circ Cardiovasc Qual Outcomes* 2020;13(7):e006565. doi: 10.1161/CIRCOUTCOMES.120.006565
38. Dupuy AM, Badiou S, Montagnon V, et al. Analytical assessment and performance of the 0/3h algorithm with novel high sensitivity cardiac troponin I. *Clin Chim Acta* 2021;519:111-17. doi: 10.1016/j.cca.2021.04.003
39. Rubini Gimenez M, Twerenbold R, Reichlin T, et al. Direct comparison of high-sensitivity-cardiac troponin I vs. T for the early diagnosis of acute myocardial infarction. *Eur Heart J* 2014;35(34):2303-11. doi: 10.1093/eurheartj/ehu188
40. Pickering JW, Young JM, George P, et al. The utility of presentation and 4-hour high sensitivity troponin I to rule-out acute myocardial infarction in the emergency department. *Clin Biochem* 2015;48(18):1219-24. doi: 10.1016/j.clinbiochem.2015.07.033
41. Miller-Hodges E, Anand A, Shah ASV, et al. High-Sensitivity Cardiac Troponin and the Risk Stratification of Patients With Renal Impairment Presenting With Suspected Acute Coronary Syndrome. *Circulation* 2018;137(5):425-35. doi: 10.1161/CIRCULATIONAHA.117.030320
42. Reichlin T, Irfan A, Twerenbold R, et al. Utility of absolute and relative changes in cardiac troponin concentrations in the early diagnosis of acute myocardial infarction. *Circulation* 2011;124(2):136-45. doi: 10.1161/CIRCULATIONAHA.111.023937
43. Reichlin T, Schindler C, Drexler B, et al. One-hour rule-out and rule-in of acute myocardial infarction using high-sensitivity cardiac troponin T. *Arch Intern Med* 2012;172(16):1211-8. doi: 10.1001/archinternmed.2012.3698
44. Reichlin T, Twerenbold R, Wildi K, et al. Prospective validation of a 1-hour algorithm to rule-out and rule-in acute myocardial infarction using a high-sensitivity cardiac troponin T assay. *CMAJ* 2015;187(8):E243-52. doi: 10.1503/cmaj.141349/-/DC1
45. Mokhtari A, Borna C, Gilje P, et al. A 1-h Combination Algorithm Allows Fast Rule-Out and Rule-In of Major Adverse Cardiac Events. *J Am Coll Cardiol* 2016;67(13):1531-40. doi: 10.1016/j.jacc.2016.01.059
46. Mokhtari A, Lindahl B, Schiopu A, et al. A 0-Hour/1-Hour Protocol for Safe, Early Discharge of Chest Pain Patients. *Acad Emerg Med* 2017;24(8):983-92. doi: 10.1111/acem.13224

47. Mueller C, Giannitsis E, Christ M, et al. Multicenter Evaluation of a 0-Hour/1-Hour Algorithm in the Diagnosis of Myocardial Infarction With High-Sensitivity Cardiac Troponin T. *Ann Emerg Med* 2016;68(1):76-87 e4. doi: 10.1016/j.annemergmed.2015.11.013

48. Shiozaki M, Inoue K, Suwa S, et al. Utility of the 0-hour/1-hour high-sensitivity cardiac troponin T algorithm in Asian patients with suspected non-ST elevation myocardial infarction. *Int J Cardiol* 2017;249:32-35. doi: 10.1016/j.ijcard.2017.09.009

49. Mueller-Hennessen M, Lindahl B, Giannitsis E, et al. Combined testing of copeptin and high-sensitivity cardiac troponin T at presentation in comparison to other algorithms for rapid rule-out of acute myocardial infarction. *Int J Cardiol* 2019;276:261-67. doi: 10.1016/j.ijcard.2018.10.084

50. Chew DP, Lambrakis K, Blyth A, et al. A Randomized Trial of a 1-Hour Troponin T Protocol in Suspected Acute Coronary Syndromes: The Rapid Assessment of Possible Acute Coronary Syndrome in the Emergency Department With High-Sensitivity Troponin T Study (RAPID-TnT). *Circulation* 2019;140(19):1543-56. doi: 10.1161/CIRCULATIONAHA.119.042891

51. Twerenbold R, Costabel JP, Nestelberger T, et al. Outcome of Applying the ESC 0/1-hour Algorithm in Patients With Suspected Myocardial Infarction. *J Am Coll Cardiol* 2019;74(4):483-94. doi: 10.1016/j.jacc.2019.05.046

52. Amann M, Gaiser F, Schwenk SI, et al. Evaluation of a 1-hour troponin algorithm for diagnosing myocardial infarction in high-risk patients admitted to a chest pain unit: the prospective FAST-MI cohort study. *BMJ Open* 2019;9(11):e032124. doi: 10.1136/bmjopen-2019-032124

53. Andruchow JE, Boyne T, Seiden-Long I, et al. Prospective comparative evaluation of the European Society of Cardiology (ESC) 1-hour and a 2-hour rapid diagnostic algorithm for myocardial infarction using high-sensitivity troponin-T. *CJEM* 2020;22(5):712-20. doi: 10.1017/cem.2020.349

54. Cappellini F, Falbo R, Saltafossi D, et al. Development of an algorithm for ruling-out non-ST elevation myocardial infarction in the emergency department using high sensitivity troponin T assay. *Clin Chim Acta* 2019;495:1-7. doi: 10.1016/j.cca.2019.03.1625

55. Shiozaki M, Inoue K, Suwa S, et al. Implementing the European Society of Cardiology 0-h/1-h algorithm in patients presenting very early after chest pain. *Int J Cardiol* 2020;320:1-6. doi: 10.1016/j.ijcard.2020.07.037

56. Allen BR, Christenson RH, Cohen SA, et al. Diagnostic Performance of High-Sensitivity Cardiac Troponin T Strategies and Clinical Variables in a Multisite US Cohort. *Circulation* 2021;143(17):1659-72. doi: 10.1161/CIRCULATIONAHA.120.049298

57. Dongxu C, Yannan Z, Yilin Y, et al. Evaluation of the 0 h/1 h high-sensitivity cardiac troponin T algorithm in diagnosis of non-ST-segment elevation myocardial infarction (NSTEMI) in Han population. *Clin Chem Lab Med* 2021;59(4):757-64. doi: 10.1515/cclm-2020-0367

58. Lopez-Ayala P, Nestelberger T, Boeddinghaus J, et al. Novel Criteria for the Observe-Zone of the ESC 0/1h-hs-cTnT Algorithm. *Circulation* 2021;144(10):773-87. doi: 10.1161/CIRCULATIONAHA.120.052982

59. Olsson P, Khoshnood A, Mokhtari A, et al. Glucose and high-sensitivity troponin T predict a low risk of major adverse cardiac events in emergency department chest pain patients. *Scand Cardiovasc J* 2021;55(6):354-61. doi: 10.1080/14017431.2021.1987512

60. Ruangsomboon O, Thirawattanasoot N, Chakorn T, et al. The utility of the 1-hour high-sensitivity cardiac troponin T

algorithm compared with and combined with five early rule-out scores in high-acuity chest pain emergency patients. *Int J Cardiol* 2021;322:23-28. doi: 10.1016/j.ijcard.2020.08.099

61. Aldous SJ, Richards AM, Cullen L, et al. Early dynamic change in high-sensitivity cardiac troponin T in the investigation of acute myocardial infarction. *Clin Chem* 2011;57(8):1154-60. doi: 10.1373/clinchem.2010.161166
62. Parsonage WA, Greenslade JH, Hammett CJ, et al. Validation of an accelerated high-sensitivity troponin T assay protocol in an Australian cohort with chest pain. *Med J Aust* 2014;200(3):161-5. doi: 10.5694/mja13.10466
63. Reichlin T, Cullen L, Parsonage WA, et al. Two-hour algorithm for triage toward rule-out and rule-in of acute myocardial infarction using high-sensitivity cardiac troponin T. *Am J Med* 2015;128(4):369-79 e4. doi: 10.1016/j.amjmed.2014.10.032
64. McRae AD, Innes G, Graham M, et al. Undetectable Concentrations of a Food and Drug Administration-approved High-sensitivity Cardiac Troponin T Assay to Rule Out Acute Myocardial Infarction at Emergency Department Arrival. *Acad Emerg Med* 2017;24(10):1267-77. doi: 10.1111/acem.13229
65. Borna C, Kollberg K, Larsson D, et al. The objective CORE score allows early rule out in acute chest pain patients. *Scand Cardiovasc J* 2018;52(6):308-14. doi: 10.1080/14017431.2018.1546891
66. Lin Z, Lim SH, Chua SJT, et al. High-sensitivity troponin T and long-term adverse cardiac events among patients presenting with suspected acute coronary syndrome in Singapore. *Singapore Med J* 2019;60(8):418-26. doi: 10.11622/smedj.2019013
67. Wildi K, Lopez-Ayala P, Koechlin L, et al. Validation of the Novel European Society of Cardiology 0/2-hour Algorithm Using Hs-cTnT in the Early Diagnosis of Myocardial Infarction. *Am J Cardiol* 2021;154:128-30. doi: 10.1016/j.amjcard.2021.06.003
68. Peacock WF, Baumann BM, Bruton D, et al. Efficacy of High-Sensitivity Troponin T in Identifying Very-Low-Risk Patients With Possible Acute Coronary Syndrome. *JAMA Cardiol* 2018;3(2):104-11. doi: 10.1001/jamacardio.2017.4625
69. Steiro OT, Tjora HL, Langorgen J, et al. Clinical risk scores identify more patients at risk for cardiovascular events within 30 days as compared to standard ACS risk criteria: the WESTCOR study. *Eur Heart J Acute Cardiovasc Care* 2021;10(3):287-301. doi: 10.1093/ehjacc/zuaa016
70. Aldous SJ, Florkowski CM, Crozier IG, et al. Comparison of high sensitivity and contemporary troponin assays for the early detection of acute myocardial infarction in the emergency department. *Ann Clin Biochem* 2011;48(Pt 3):241-8. doi: 10.1258/acb.2010.010219
71. Aldous S, Pemberton C, Richards AM, et al. High-sensitivity troponin T for early rule-out of myocardial infarction in recent onset chest pain. *Emerg Med J* 2012;29(10):805-10. doi: 10.1136/emermed-2011-200222
72. Irfan A, Reichlin T, Twerenbold R, et al. Early diagnosis of myocardial infarction using absolute and relative changes in cardiac troponin concentrations. *Am J Med* 2013;126(9):781-88 e2. doi: 10.1016/j.amjmed.2013.02.031
73. Biener M, Mueller M, Vafaie M, et al. Impact of leading presenting symptoms on the diagnostic performance of high-sensitivity cardiac troponin T and on outcomes in patients with suspected acute coronary syndrome. *Clin Chem* 2015;61(5):744-51. doi: 10.1373/clinchem.2014.235317
74. Nowak RM, Gandolfo CM, Jacobsen G, et al. Ultrarapid Rule-out for Acute Myocardial Infarction Using the Generation 5 Cardiac Troponin T Assay: Results From the REACTION-US Study. *Ann Emerg Med*

2018;72(6):654-64. doi: 10.1016/j.annemergmed.2018.06.021

75. Jaeger C, Wildi K, Twerenbold R, et al. One-hour rule-in and rule-out of acute myocardial infarction using high-sensitivity cardiac troponin I. *Am Heart J* 2016;171(1):92-102 e1-5. doi: 10.1016/j.ahj.2015.07.022
76. Boeddinghaus J, Twerenbold R, Nestelberger T, et al. Clinical Validation of a Novel High-Sensitivity Cardiac Troponin I Assay for Early Diagnosis of Acute Myocardial Infarction. *Clin Chem* 2018;64(9):1347-60. doi: 10.1373/clinchem.2018.286906
77. Chapman AR, Fujisawa T, Lee KK, et al. Novel high-sensitivity cardiac troponin I assay in patients with suspected acute coronary syndrome. *Heart* 2019;105(8):616-22. doi: 10.1136/heartjnl-2018-314093
78. Nowak RM, Christenson RH, Jacobsen G, et al. Performance of Novel High-Sensitivity Cardiac Troponin I Assays for 0/1-Hour and 0/2- to 3-Hour Evaluations for Acute Myocardial Infarction: Results From the HIGH-US Study. *Ann Emerg Med* 2020;76(1):1-13. doi: 10.1016/j.annemergmed.2019.12.008
79. Andersen CF, Bang C, Lauridsen KG, et al. External validation of a high-sensitive troponin I algorithm for rapid evaluation of acute myocardial infarction in a Danish cohort. *Eur Heart J Acute Cardiovasc Care* 2021;10(9):1056-64. doi: 10.1093/ehjacc/zuab062
80. McCord J, Hana A, Cook B, et al. The role of cardiac testing with the 0/1-hour high-sensitivity cardiac troponin algorithm evaluating for acute myocardial infarction. *Am Heart J* 2021;233:68-77. doi: 10.1016/j.ahj.2020.12.015
81. Greenslade JH, Carlton EW, Van Hise C, et al. Diagnostic Accuracy of a New High-Sensitivity Troponin I Assay and Five Accelerated Diagnostic Pathways for Ruling Out Acute Myocardial Infarction and Acute Coronary Syndrome. *Ann Emerg Med* 2018;71(4):439-51 e3. doi: 10.1016/j.annemergmed.2017.10.030
82. Peacock WF, Christenson R, Diercks DB, et al. Myocardial Infarction Can Be Safely Excluded by High-sensitivity Troponin I Testing 3 Hours After Emergency Department Presentation. *Acad Emerg Med* 2020 doi: 10.1111/acem.13922
83. Kavsak PA, Mondoux SE, Ma J, et al. Comparison of two biomarker only algorithms for early risk stratification in patients with suspected acute coronary syndrome. *Int J Cardiol* 2020;319:140-43. doi: 10.1016/j.ijcard.2020.06.066
84. Schofer N, Brunner F, Schlüter M, et al. Gender-specific diagnostic performance of a new highsensitivity cardiac troponin I assay for detection of acute myocardial infarction. *Eur Heart J* 2017;6(1):60-68. doi: 10.1177/2048872615626660
85. Kavsak PA, Mondoux SE, Sherbino J, et al. Clinical evaluation of Ortho Clinical Diagnostics high-sensitivity cardiac Troponin I assay in patients with symptoms suggestive of acute coronary syndrome. *Clin Biochem* 2020;80:48-51. doi: 10.1016/j.clinbiochem.2020.04.003
86. Schreiber DH, Agbo C, Wu AH. Short-term (90 min) diagnostic performance for acute non-ST segment elevation myocardial infarction and 30-day prognostic evaluation of a novel third-generation high sensitivity troponin I assay. *Clin Biochem* 2012;45(16-17):1295-301. doi: 10.1016/j.clinbiochem.2012.06.005
87. Agewall S, Olsson T, Lowbeer C. Usefulness of troponin levels below the diagnostic cut-off level for acute myocardial infarction in predicting prognosis in unselected patients admitted to the coronary care unit. *Am J Cardiol* 2007;99(10):1357-9. doi: 10.1016/j.amjcard.2006.12.059
88. Ahmadi AA, Alemzadeh-Ansari MJ, Jenab Y, et al. Comparison of High-Sensitive Cardiac Troponin-T Changes

between Non-ST-Segment Elevation Myocardial Infarction and Non-Coronary Artery Patients. *Iranian Red Crescent Medical Journal* 2017;19(10) doi: 10.5812/ircmj.55663

89. Alcalai R, Varshisky B, Marhig A, et al. Assessing the Performance of a Novel Point-of-Care Qualitative Assay for Early Diagnosis of Acute Coronary Syndrome. *Cardiology* 2021;146(1):34-41. doi: 10.1159/000511435
90. Aldous SJ, Florkowski CM, Crozier IG, et al. The performance of high sensitivity troponin for the diagnosis of acute myocardial infarction is underestimated. *Clin Chem Lab Med* 2011;50(4):727-9. doi: 10.1515/cclm.2011.830
91. Aldous SJ, Richards MA, Cullen L, et al. A new improved accelerated diagnostic protocol safely identifies low-risk patients with chest pain in the emergency department. *Acad Emerg Med* 2012;19(5):510-6. doi: 10.1111/j.1553-2712.2012.01352.x
92. Aldous S, Richards M, Cullen L, et al. Diagnostic and prognostic utility of early measurement with high-sensitivity troponin T assay in patients presenting with chest pain. *CMAJ* 2012;184(5):E260-8. doi: 10.1503/cmaj.110773
93. Alghamdi A, Reynard C, Morris N, et al. Diagnostic accuracy of the Troponin-only Manchester Acute Coronary Syndromes (T-MACS) decision aid with a point-of-care cardiac troponin assay. *Emerg Med J* 2020;37(4):223-28. doi: 10.1136/emermed-2019-208882
94. Al-Saleh A, Alazzoni A, Al Shalash S, et al. Performance of the high-sensitivity troponin assay in diagnosing acute myocardial infarction: systematic review and meta-analysis. *CMAJ Open* 2014;2(3):E199-207. doi: 10.9778/cmajo.20130074
95. Alushi B, Jost-Brinkmann F, Kastrati A, et al. High-Sensitivity Cardiac Troponin T in Patients with Severe Chronic Kidney Disease and Suspected Acute Coronary Syndrome. *J Clin Med* 2021;10(18) doi: 10.3390/jcm10184216
96. Anand A, Lee KK, Chapman AR, et al. High-Sensitivity Cardiac Troponin on Presentation to Rule Out Myocardial Infarction: A Stepped-Wedge Cluster Randomized Controlled Trial. *Circulation* 2021;143(23):2214-24. doi: 10.1161/CIRCULATIONAHA.120.052380
97. Apple F, Ler R, Chung A, et al. Point-of-care i-STAT cardiac troponin I for assessment of patients with symptoms suggestive of acute coronary syndrome. *Clin Chem* 2006;52(2):322-5
98. Apple FS, Pearce LA, Smith SW, et al. Role of monitoring changes in sensitive cardiac troponin I assay results for early diagnosis of myocardial infarction and prediction of risk of adverse events. *Clin Chem* 2009;55(5):930-7. doi: 10.1373/clinchem.2008.114728
99. Astley CM, Beltrame JF, Zeitz C, et al. Study design of embracing high-sensitivity troponin effectively: the value of more information: a randomized comparison. *Contemp Clin Trials* 2014;39(2):183-90. doi: 10.1016/j.cct.2014.08.012
100. Ter Avest E, Visser A, Reitsma B, et al. Point-of-care troponin T is inferior to high-sensitivity troponin T for ruling out acute myocardial infarction in the emergency department. *Eur J Emerg Med* 2016;23(2):95-101. doi: 10.1186/cc10270
101. Bahrmann P, Heppner HJ, Christ M, et al. Early detection of non-ST-elevation myocardial infarction in geriatric patients by a new high-sensitive cardiac troponin T assay. *Aging Clin Exp Res* 2012;24(3):290-4. doi: 10.3275/7927
102. Bahrmann P, Christ M, Bahrmann A, et al. A 3-hour diagnostic algorithm for non-ST-elevation myocardial infarction using high-sensitivity cardiac troponin T in unselected older patients presenting to the emergency department. *J*

Am Med Dir Assoc 2013;14(6):409-16. doi: 10.1016/j.jamda.2012.12.005

103. Bahrmann P, Bertsch T, Sieber CC, et al. Management of patients with chest pain presenting to the emergency department: in need for the implementation of the 1 h rapid rule-out algorithm using high-sensitivity troponin I assays in clinical practice. *Ann Transl Med* 2016;4(1):18. doi: 10.3978/j.issn.2305-5839.2015.12.31
104. Bali T, Boda S, Reynard C, et al. The association between cardiac risk factors and the probability of acute myocardial infarction in the emergency department: analysis from a multicentre prospective observational study in the high sensitivity troponin era. *Eur J Emerg Med* 2021;28(3):233-7. doi: 10.1097/MEJ.0000000000000809.
105. Ballocca F, D'Ascenzo F, Moretti C, et al. High sensitive TROponin levels In Patients with Chest pain and kidney disease: A multicenter registry - The TROPIC study. *Cardiol J* 2017;24(2):139-50. doi: 10.5603/CJ.a2017.0025
106. Bandstein N, Ljung R, Johansson M, et al. Undetectable high-sensitivity cardiac troponin T level in the emergency department and risk of myocardial infarction. *J Am Coll Cardiol* 2014;63(23):2569-78. doi: 10.1016/j.jacc.2014.03.017
107. Bandstein N, Ljung R, Holzmann MJ. Risk of revisits to the emergency department in admitted versus discharged patients with chest pain but without myocardial infarction in relation to high-sensitivity cardiac troponin T levels. *Int J Cardiol* 2016;203:341-6. doi: 10.1016/j.ijcard.2015.10.170
108. Bandstein N, Wikman A, Ljung R, et al. Survival and resource utilization in patients with chest pain evaluated with cardiac troponin T compared with high-sensitivity cardiac troponin T. *Int J Cardiol* 2017;245:43-48. doi: 10.1016/j.ijcard.2017.05.111
109. Bellini C, Cinci F, Bova G, et al. Methodology to Evaluate Clinical Impact of 0/3 Hour High-Sensitivity Cardiac Troponin T Protocol on Managing Acute Coronary Syndrome in Daily Emergency Department Practice. *Lab Med* 2021;52(5):452-59. doi: 10.1093/labmed/lmaa118
110. Bhardwaj A, Truong QA, Peacock WF, et al. A multicenter comparison of established and emerging cardiac biomarkers for the diagnostic evaluation of chest pain in the emergency department. *Am Heart J* 2011;162(2):276-82 e1. doi: 10.1016/j.ahj.2011.05.022
111. Biener M, Mueller M, Vafaie M, et al. Comparison of a 3-hour versus a 6-hour sampling-protocol using high-sensitivity cardiac troponin T for rule-out and rule-in of non-STEMI in an unselected emergency department population. *Int J Cardiol* 2013;167(4):1134-40. doi: 10.1016/j.ijcard.2012.09.122
112. Birkhahn RH, Haines E, Wen W, et al. Estimating the clinical impact of bringing a multimarker cardiac panel to the bedside in the ED. *Am J Emerg Med* 2011;29(3):304-8. doi: 10.1016/j.ajem.2009.12.007
113. Bjurman C, Larsson M, Johanson P, et al. Small changes in troponin T levels are common in patients with non-ST-segment elevation myocardial infarction and are linked to higher mortality. *J Am Coll Cardiol* 2013;62(14):1231-38. doi: 10.1016/j.jacc.2013.06.050
114. Body R, Carley S, McDowell G, et al. Rapid exclusion of acute myocardial infarction in patients with undetectable troponin using a high-sensitivity assay. *J Am Coll Cardiol* 2011;58(13):1332-9. doi: 10.1016/j.jacc.2011.06.026
115. Body R, Burrows G, Carley S, et al. High-sensitivity cardiac troponin t concentrations below the limit of detection to exclude acute myocardial infarction: a prospective evaluation. *Clin Chem* 2015;61(7):983-9. doi: 10.1373/clinchem.2014.231530
116. Body R, Burrows G, Carley S, et al. Rapid exclusion of acute myocardial infarction in patients with undetectable

troponin using a sensitive troponin I assay. *Ann Clin Biochem* 2015;52(Pt 5):543-9. doi: 10.1177/0004563215576976

117. Body R, Mueller C, Giannitsis E, et al. The Use of Very Low Concentrations of High-sensitivity Troponin T to Rule Out Acute Myocardial Infarction Using a Single Blood Test. *Acad Emerg Med* 2016;23(9):1004-13. doi: 10.1111/acem.13012
118. Body R, Morris N, Reynard C, et al. Comparison of four decision aids for the early diagnosis of acute coronary syndromes in the emergency department. *Emerg Med J* 2020;37(1):8-13. doi: 10.1136/emermed-2019-208898
119. Boeckel JN, Palapies L, Zeller T, et al. Estimation of Values below the Limit of Detection of a Contemporary Sensitive Troponin I Assay Improves Diagnosis of Acute Myocardial Infarction. *Clin Chem* 2015;61(9):1197-206. doi: 10.1373/clinchem.2015.238949
120. Boeddinghaus J, Reichlin T, Nestelberger T, et al. Early diagnosis of acute myocardial infarction in patients with mild elevations of cardiac troponin. *Clin Res Cardiol* 2017;106(6):457-67. doi: 10.1007/s00392-016-1075-9
121. Bonaca MP, Ruff CT, Kosowsky J, et al. Evaluation of the diagnostic performance of current and next-generation assays for cardiac troponin I in the BWH-TIMI ED Chest Pain Study. *Eur Heart J Acute Cardiovasc Care* 2013;2(3):195-202. doi: 10.1177/2048872613486249
122. Borna C, Thelin J, Ohlin B, et al. High-sensitivity troponin T as a diagnostic tool for acute coronary syndrome in the real world: an observational study. *Eur J Emerg Med* 2014;21(3):181-8. doi: 10.1097/MEJ.0b013e328362a71b
123. Borna C, Frostred KL, Ekelund U. Predictive role of high sensitivity troponin T within four hours from presentation of acute coronary syndrome in elderly patients. *BMC Emerg Med* 2016;16:1. doi: 10.1186/s12873-015-0064-z
124. Bove J, Hochman S, Miller J, et al. Effectiveness of 2-hour Troponin in High-risk Patients With Suspected Acute Coronary Syndrome. *Crit Pathw Cardiol* 2017;16(2):53-57. doi: 10.1097/HPC.0000000000000111
125. Braga F, Dolci A, Cavallero A, et al. Evaluation of the sensitivity of two highly sensitive troponin assays for early detection of non ST-elevation myocardial infarction (NSTEMI). *Biochimica Clinica* 2011;35(3):186-89.
126. Brichko L, Schneider HG, Chan W, et al. Rapid and safe discharge from the emergency department: A single troponin to exclude acute myocardial infarction. *Emerg Med Australas* 2018;30(4):486-93. doi: 10.1111/1742-6723.12919
127. Bularga A, Lee KK, Stewart S, et al. High-Sensitivity Troponin and the Application of Risk Stratification Thresholds in Patients With Suspected Acute Coronary Syndrome. *Circulation* 2019;140(19):1557-68. doi: 10.1161/CIRCULATIONAHA.119.042866
128. Caglar B, Serin S. Diagnostic Performance of One-hour Delta High Sensitive Troponin in Acute Coronary Syndrome. *J Coll Physicians Surg Pak* 2020;30(9):906-11. doi: 10.29271/jcpsp.2020.09.906
129. Carlton E, Greenslade J, Cullen L, et al. Evaluation of High-Sensitivity Cardiac Troponin I Levels in Patients With Suspected Acute Coronary Syndrome. *JAMA Cardiol* 2016;1(4):405-12. doi: 10.1001/jamacardio.2016.1309
130. Carlton E, Khattab A, Greaves K. Beyond triage: the diagnostic accuracy of emergency department nursing staff risk assessment in patients with suspected acute coronary syndromes. *Emerg Med j* 2016;33(2):99-104. doi: 10.1136/emermed-2015-20478
131. Carlton EW, Pickering JW, Greenslade J, et al. Assessment of the 2016 National Institute for Health and Care Excellence high-sensitivity troponin rule-out strategy. *Heart* 2018;104(8):665-72. doi: 10.1136/heartjnl-2017-311983

132. Carlton EW, Ingram J, Taylor H, et al. Limit of detection of troponin discharge strategy versus usual care: randomised controlled trial. *Heart* 2020;106(20):1586-94. doi: 10.1136/heartjnl-2020-316692

133. Cavender MA, White WB, Jarolim P, et al. Serial Measurement of High-Sensitivity Troponin I and Cardiovascular Outcomes in Patients With Type 2 Diabetes Mellitus in the EXAMINE Trial (Examination of Cardiovascular Outcomes With Alogliptin Versus Standard of Care). *Circulation* 2017;135(20):1911-21. doi: 10.1161/CIRCULATIONAHA.116.024632

134. Celik S, Giannitsis E, Wollert KC, et al. Cardiac troponin T concentrations above the 99th percentile value as measured by a new high-sensitivity assay predict long-term prognosis in patients with acute coronary syndromes undergoing routine early invasive strategy. *Clin Res Cardiol* 2011;100(12):1077-85. doi: 10.1007/s00392-011-0344-x

135. Chapman AR, Lee KK, McAllister DA, et al. Association of High-Sensitivity Cardiac Troponin I Concentration With Cardiac Outcomes in Patients With Suspected Acute Coronary Syndrome. *JAMA* 2017;318(19):1913-24. doi: 10.1001/jama.2017.17488

136. Charpentier S, Chenevier-Gobeaux C. [2015 ESC guidelines: 1-hour rule-out and rule-in of acute myocardial infarction with high-sensitive troponin T]. *Presse Med* 2016;45(10):859-64. doi: 10.1016/j.lpm.2016.05.023

137. Chenevier-Gobeaux C, Meune C, Lefevre G, et al. A single value of high-sensitive troponin T below the limit of detection is not enough for ruling out non ST elevation myocardial infarction in the emergency department. *Clin Biochem* 2016;49(15):1113-17. doi: 10.1016/j.clinbiochem.2016.05.021

138. Chew DP, Zeitz C, Worthley M, et al. Randomized Comparison of High-Sensitivity Troponin Reporting in Undifferentiated Chest Pain Assessment. *Circ Cardiovasc Qual Outcomes* 2016;9(5):542-53. doi: 10.1161/CIRCOUTCOMES.115.002488

139. Chew G, Frost F, Mullen L, et al. A direct comparison of decision rules for early discharge of suspected acute coronary syndromes in the era of high sensitivity troponin. *Eur Heart J Acute Cardiovasc Care* 2018;8(5):421-31. doi: 10.1177/2048872618755369

140. Christ M, Popp S, Pohlmann H, et al. Implementation of high sensitivity cardiac troponin T measurement in the emergency department. *Am J Med* 2010;123(12):1134-42. doi: 10.1016/j.amjmed.2010.07.015

141. Christ M, Geier F, Blaschke S, et al. Clinical performance of a new point-of-care cardiac troponin I test. *Clin Chem Lab Med* 2018;56(8):1336-44. doi: 10.1515/cclm-2017-0693

142. Christenson RH, Duh SH, Apple FA, et al. Pivotal findings for a high-sensitivity cardiac troponin assay: Results of the HIGH-US study. *Clin Biochem* 2020;78:32-39. doi: 10.1016/j.clinbiochem.2019.10.013

143. Chuang A, Hancock DG, Horsfall M, et al. The predictive value of high sensitivity-troponin velocity within the first 6h of presentation for cardiac outcomes regardless of acute coronary syndrome diagnosis. *Int J Cardiol* 2016;204:106-11. doi: 10.1016/j.ijcard.2015.11.132

144. Clerico A, Ripoli A, Zaninotto M, et al. Head-to-head comparison of plasma cTnI concentration values measured with three high-sensitivity methods in a large Italian population of healthy volunteers and patients admitted to emergency department with acute coronary syndrome: A multi-center study. *Clin Chim Acta* 2019;496:25-34. doi: 10.1016/j.cca.2019.06.012

145. Čolak T, Mikulić I, Landeka K, et al. Predictive Value of High Sensitive Troponin I Assay in Acute Coronary Syndrome Compared to Classic Biochemical Markers. *Psychiatr Danub* 2017;29:823-29.

146. Collinson P, Gaze D, Morris B, et al. Comparison of biomarker strategies for rapid rule out of myocardial infarction in the emergency department using ACC/ESC diagnostic criteria. *Ann Clin Biochem* 2006;43(4):273-80.

147. Collinson PO, Gaze DC, Bainbridge K, et al. Utility of admission cardiac troponin and "Ischemia Modified Albumin" measurements for rapid evaluation and rule out of suspected acute myocardial infarction in the emergency department. *Emerg Med J* 2006;23(4):256-61. doi: 10.1136/emj.2005.028241

148. Collinson PO, Gaze D, Goodacre S. The clinical and diagnostic performance characteristics of the high sensitivity Abbott cardiac troponin I assay. *Clin Biochem* 2015;48(4-5):275-81. doi: 10.1016/j.clinbiochem.2014.12.017

149. Unger P, Dedobbeleer C, Vanden Eynden F, et al. Mitral regurgitation after transcatheter aortic valve replacement: does the prosthesis matter? *Int J Cardiol* 2013;168(2):1706-9. doi: 10.1016/j.ijcard.2013.03.055

150. Cook B, McCord J, Hudson M, et al. Baseline High Sensitivity Cardiac Troponin I Level Below Limit of Quantitation Rules Out Acute Myocardial Infarction in the Emergency Department. *Crit Pathw Cardiol* 2021;20(1):4-9. doi: 10.1097/HPC.0000000000000230

151. Corsini A, Vagnarelli F, Bugani G, et al. Impact of high-sensitivity Troponin T on hospital admission, resources utilization, and outcomes. *Eur Heart J Acute Cardiovasc Care* 2015;4(2):148-57. doi: 10.1177/2048872614547687

152. Cramer GE, Kievit PC, Brouwer MA, et al. Lack of concordance between a rapid bedside and conventional laboratory method of cardiac troponin testing: impact on risk stratification of patients suspected of acute coronary syndrome. *Clin Chim Acta* 2007;381(2):164-6. doi: 10.1016/j.cca.2007.03.001

153. Cullen L, Parsonage WA, Greenslade J, et al. Delta troponin for the early diagnosis of AMI in emergency patients with chest pain. *Int J Cardiol* 2013;168(3):2602-8. doi: 10.1016/j.ijcard.2013.03.044

154. Cullen L, Greenslade JH, Carlton EW, et al. Sex-specific versus overall cut points for a high sensitivity troponin I assay in predicting 1-year outcomes in emergency patients presenting with chest pain. *Heart* 2016;102(2):120-6. doi: 10.1136/heartjnl-2015-308506

155. Dadkhah S, Almuwaqqat Z, Sulaiman S, et al. Sensitive Troponin I and Stress Testing in the Emergency Department for the Early Management of Chest Pain Using 2-Hour Protocol. *Crit Pathw Cardiol* 2017;16(3):89-92. doi: 10.1097/HPC.0000000000000115

156. Daly MJ, Adgey JA, Harbinson MT. Improved detection of acute myocardial infarction in patients with chest pain and significant left main stem coronary stenosis. *QJM* 2012;105(2):127-35. doi: 10.1093/qjmed/hcr134

157. Davarani H, Afzalimoghadam M, Hosseinnejad H, et al. Increasing serum troponin I and early prognosis in patients with chest pain or angina equivalent symptoms in the emergency department. *Iran J Public Health* 2012;41(2):63-69.

158. Dawson C, Benger JR, Bayly G. Serial high-sensitivity troponin measurements for the rapid exclusion of acute myocardial infarction in low-risk patients. *Emerg Med J* 2013;30(7):593-4. doi: 10.1136/emermed-2012-201574

159. Diercks DB, Peacock WFt, Hollander JE, et al. Diagnostic accuracy of a point-of-care troponin I assay for acute myocardial infarction within 3 hours after presentation in early presenters to the emergency department with chest pain. *Am Heart J* 2012;163(1):74-80 e4. doi: 10.1016/j.ahj.2011.09.028

160. Druey S, Wildi K, Twerenbold R, et al. Early rule-out and rule-in of myocardial infarction using sensitive cardiac Troponin I. *Int J Cardiol* 2015;195:163-70. doi: 10.1016/j.ijcard.2015.05.079

161. Eggers KM, Jaffe AS, Venge P, et al. Clinical implications of the change of cardiac troponin I levels in patients with acute chest pain - an evaluation with respect to the Universal Definition of Myocardial Infarction. *Clin Chim Acta* 2011;412(1-2):91-7. doi: 10.1016/j.cca.2010.09.020
162. Etaher A, Chew DP, Frost S, et al. Prognostic Implications of High-Sensitivity Troponin T Levels Among Patients Attending Emergency Departments and Evaluated for an Acute Coronary Syndrome. *Am J Med* 2021;134(8):1019-28 e1. doi: 10.1016/j.amjmed.2021.03.005
163. Fan LY, Yu P, Yu SS, et al. Age-specific 99th percentile cutoff of high-sensitivity cardiac troponin T for early prediction of non-ST-segment elevation myocardial infarction (NSTEMI) in middle-aged patients. *J Clin Lab Anal* 2014;28(1):10-5. doi: 10.1002/jcla.21636
164. Fanaroff AC, Schulteis RD, Pieper KS, et al. Simplified Predictive Instrument to Rule Out Acute Coronary Syndromes in a High-Risk Population. *J Am Heart Assoc* 2015;4(12) doi: 10.1161/JAHA.115.002351
165. Freund Y, Chenevier-Gobeaux C, Bonnet P, et al. High-sensitivity versus conventional troponin in the emergency department for the diagnosis of acute myocardial infarction. *Crit Care* 2011
166. Frisoli TM, Nowak R, Evans KL, et al. Henry Ford HEART Score Randomized Trial: Rapid Discharge of Patients Evaluated for Possible Myocardial Infarction. *Circ Cardiovasc Qual Outcomes* 2017;10(10) doi: 10.1161/CIRCOUTCOMES.117.003617
167. Gassenmaier T, Buchner S, Birner C, et al. High-sensitive Troponin I in acute cardiac conditions: implications of baseline and sequential measurements for diagnosis of myocardial infarction. *Atherosclerosis* 2012;222(1):116-22. doi: 10.1016/j.atherosclerosis.2012.02.007
168. Giannitsis E, Becker M, Kurz K, et al. High-sensitivity cardiac troponin T for early prediction of evolving non-ST-segment elevation myocardial infarction in patients with suspected acute coronary syndrome and negative troponin results on admission. *Clin Chem* 2010;56(4):642-50. doi: 10.1373/clinchem.2009.134460
169. Giannitsis E, Kurz K, Hallermayer K, et al. Analytical validation of a high-sensitivity cardiac troponin T assay. *Clin Chem* 2010;56(2):254-61. doi: 10.1373/clinchem.2009.132654
170. Rubini Gimenez M, Twerenbold R, Boeddinghaus J, et al. Clinical Effect of Sex-Specific Cutoff Values of High-Sensitivity Cardiac Troponin T in Suspected Myocardial Infarction. *JAMA Cardiol* 2016;1(8):912-20. doi: 10.1001/jamacardio.2016.2882
171. Rubini Gimenez M, Wildi K, Wussler D, et al. Early kinetics of cardiac troponin in suspected acute myocardial infarction. *Rev Esp Cardiol (Engl Ed)* 2020 doi: 10.1016/j.rec.2020.04.008 [published Online First: 2020/05/27]
172. Giner-Caro JA, Caballero L, Casas-Pina T, et al. High sensitive cardiac troponin T in the management of uncertain chest pain. *Int J Cardiol* 2013;168(4):4422-3. doi: 10.1016/j.ijcard.2013.05.015
173. Goodman DA, Kavsak PA, Hill SA, et al. Presenting characteristics of patients undergoing cardiac troponin measurements in the emergency department. *CJEM* 2015;17(1):62-6. doi: 10.2310/8000.2013.131298
174. Gratzel P. Neuer Algorithmus bei Infarktverdacht. *MMW Fortschritte der Medizin* 2015;157(16):9.
175. Graven T, Klykken B, Kleinau O, et al. Measurement of high-sensitivity troponin-I in suspected coronary related chest pain in Emergency Departments. *Tidsskr Nor Laegeforen* 2021;141:2021-12. doi: 10.4045/tidsskr.21.0037
176. Greenslade J, Cho E, Van Hise C, et al. Evaluating Rapid Rule-out of Acute Myocardial Infarction Using a High-Sensitivity Cardiac Troponin I Assay at Presentation. *Clin Chem* 2018;64(5):820-29. doi:

10.1373/clinchem.2017.283887

177. Grinstein J, Bonaca MP, Jarolim P, et al. Prognostic implications of low level cardiac troponin elevation using high-sensitivity cardiac troponin T. *Clin Cardiol* 2015;38(4):230-5. doi: 10.1002/clc.22379
178. Haaf P, Reichlin T, Twerenbold R, et al. Risk stratification in patients with acute chest pain using three high-sensitivity cardiac troponin assays. *Eur Heart J* 2014;35(6):365-75. doi: 10.1093/eurheartj/eht218
179. Haider DG, Klemenz T, Fiedler GM, et al. High sensitive cardiac troponin T: Testing the test. *Int J Cardiol* 2017;228:779-83. doi: 10.1016/j.ijcard.2016.10.043
180. Halim SA, Mulgund J, Chen AY, et al. Use of guidelines-recommended management and outcomes among women and men with low-level troponin elevation: insights from CRUSADE. *Circ Cardiovasc Qual Outcomes* 2009;2(3):199-206. doi: 10.1161/CIRCOUTCOMES.108.810127
181. Hall T, Hallén J, Agewall S, et al. Changes in diagnosing non-ST-segment elevation myocardial infarction after the introduction of a new high-sensitivity cardiac troponin T assay: a single-centre experience. *Clin Lab* 2012;58(9-10):1029-36.
182. Hochholzer W, Reichlin T, Twerenbold R, et al. Incremental value of high-sensitivity cardiac troponin T for risk prediction in patients with suspected acute myocardial infarction. *Clin Chem* 2011;57(9):1318-26. doi: 10.1373/clinchem.2011.162073
183. Hochholzer W, Valina CM, Stratz C, et al. High-sensitivity cardiac troponin for risk prediction in patients with and without coronary heart disease. *Int J Cardiol* 2014;176(2):444-9. doi: 10.1016/j.ijcard.2014.07.094
184. Hochholzer W, Neumann F. The New 2015 ESC Guidelines for the Management of Acute Coronary Syndromes in Patients Presenting Without Persistent ST-segment Elevation. *Dtsch Med Wochenschr* 2016;141(11):782-5. doi: 10.1055/s-0042-107115
185. Hoeller R, Rubini Gimenez M, Reichlin T, et al. Normal presenting levels of high-sensitivity troponin and myocardial infarction. *Heart* 2013;99(21):1567-72. doi: 10.1136/heartjnl-2013-303643
186. Hromádka M, Rajdl D, Trefil L, et al. Diagnostic sensitivity of high-sensitivity troponin T in acute myocardial infarction in patients with chest pain. *Cor et Vasa* 2012;54(7-8):e227-e31. doi: 10.1016/j.crvasa.2012.06.002
187. Huang H, Zhu S, Wang W, et al. Diagnosis of acute myocardial infarction in patients with renal insufficiency using high-sensitivity troponin T. *Clin Chem Lab Med* 2015;53(5):723-30. doi: 10.1515/cclm-2014-0715
188. Huang HL, Zhu S, Wang WQ, et al. Diagnosis of Acute Myocardial Infarction in Hemodialysis Patients With High-Sensitivity Cardiac Troponin T Assay. *Arch Pathol Lab Med* 2016;140(1):75-80. doi: 10.5858/arpa.2014-0580-OA
189. Ilva T, Lund J, Porela P, et al. Early markers of myocardial injury: cTnI is enough. *Clin Chim Acta* 2009;400(1-2):82-5. doi: 10.1016/j.cca.2008.10.005
190. Invernizzi L, Doka M, Cappellini F, et al. Effectiveness of highly sensitive troponin T assay for early diagnosis of acute myocardial infarction (AMI). *Biochimica clinica* 2013;37(1):36-39.
191. Işıksaçan N, Bıyık İ, Ertürk M, et al. Comparison of high sensitive and conventional troponin assays in diagnosis of acute myocardial infarction. *Turk J Biochem* 2017;42(1):77-85. doi: 10.1515/tjb-2016-0270
192. Iwasaki M, Yamazaki K, Ikeda N, et al. Point of care assessment of cardiac troponin T level in CKD patients with chest symptom. *Ren Fail* 2017;39(1):166-72. doi: 10.1080/0886022X.2016.1256311

193. Jairam S, Jones P, Samaraie L, et al. Clinical diagnosis and outcomes for Troponin T 'positive' patients assessed by a high sensitivity compared with a 4th generation assay. *Emerg Med Australas* 2011;23(4):490-501. doi: 10.1111/j.1742-6723.2011.01446.x

194. Johannessen TR, Vallersnes OM, Halvorsen S, et al. Pre-hospital One-Hour Troponin in a Low-Prevalence Population of Acute Coronary Syndrome: OUT-ACS study. *Open Heart* 2020;7(2) doi: 10.1136/openhrt-2020-001296

195. Karady J, Mayrhofer T, Ferencik M, et al. Discordance of High-Sensitivity Troponin Assays in Patients With Suspected Acute Coronary Syndromes. *J Am Coll Cardiol* 2021;77(12):1487-99. doi: 10.1016/j.jacc.2021.01.046

196. Karon BS, Wockenfus AM, Hartung KJ, et al. Comparing analytical outliers and the percent of emergency department patients with results above the 99th percentile upper reference limit for 2 conventional and one high sensitivity troponin assay. *Clin Biochem* 2018;53:104-09. doi: 10.1016/j.clinbiochem.2018.01.001

197. Kavsak PA, MacRae AR, Palomaki GE, et al. Health outcomes categorized by current and previous definitions of acute myocardial infarction in an unselected cohort of troponin-naive emergency department patients. *Clin Chem* 2006;52(11):2028-35. doi: 10.1373/clinchem.2006.073403

198. Kavsak PA, Wang X, Ko DT, et al. Short- and long-term risk stratification using a next-generation, high-sensitivity research cardiac troponin I (hs-cTnI) assay in an emergency department chest pain population. *Clin Chem* 2009;55(10):1809-15. doi: 10.1373/clinchem.2009.127241

199. Kavsak P, Hill S, Bhanich SW, et al. Biomarkers for Predicting Serious Cardiac Outcomes at 72 Hours in Patients Presenting Early after Chest Pain Onset with Symptoms of Acute Coronary Syndromes. *Clin Chem* 2012;58(1):298-302. doi: 10.1373/clinchem.2011.172064

200. Kavsak PA, Shortt C, Ma J, et al. A laboratory score at presentation to rule-out serious cardiac outcomes or death in patients presenting with symptoms suggestive of acute coronary syndrome. *Clin Chim Acta* 2017;469:69-74. doi: 10.1016/j.cca.2017.03.021

201. Kavsak P, Worster A, Shortt C, et al. High-sensitivity Cardiac Troponin Concentrations at Emergency Department Presentation in Females and Males With an Acute Cardiac Outcome. *Ann Clin Biochem* 2018;55(5):604-07. doi: 10.1177/0004563217743997

202. Kavsak PA, Worster A, Shortt C, et al. Performance of high-sensitivity cardiac troponin in the emergency department for myocardial infarction and a composite cardiac outcome across different estimated glomerular filtration rates. *Clin Chim Acta* 2018;479:166-70. doi: 10.1016/j.cca.2018.01.034

203. Kavsak PA, Cerasuolo JO, Ko DT, et al. Using the clinical chemistry score in the emergency department to detect adverse cardiac events: a diagnostic accuracy study. *CMAJ Open* 2020;8(4):E676-E84. doi: 10.9778/cmajo.20200047

204. Kelly AM. Performance of a sensitive troponin assay in the early diagnosis of acute myocardial infarction in the emergency department. *Emerg Med Australas* 2011;23(2):181-5. doi: 10.1111/j.1742-6723.2011.01388.x

205. Kelly A, Klim S. What is the 30-day rate of adverse cardiac events in chest pain patients with ED troponin I assays≤99th centile using a contemporary sensitive assay? An exploratory analysis. *Eur J Emerg Med* 2014;21(4):276-80. doi: 10.1097/MEJ.0000000000000079

206. Kelly AM, Klim S. Prospective external validation of an accelerated (2-h) acute coronary syndrome rule-out process using a contemporary troponin assay. *Int J Emerg Med* 2014;7:42. doi: 10.1186/s12245-014-0042-3

207. Kelly AM, Klim S. Does undetectable troponin I at presentation using a contemporary sensitive assay rule out myocardial infarction? A cohort study. *Emerg Med J* 2015;32(10):760-3. doi: 10.1136/emermed-2014-204442

208. Kemper DW, Semjonow V, de Theije F, et al. Analytical evaluation of a new point of care system for measuring cardiac Troponin I. *Clin Biochem* 2017;50(4-5):174-80. doi: 10.1016/j.clinbiochem.2016.11.011

209. Khan E, Lambrakis K, Blyth A, et al. Classification performance of clinical risk scoring in suspected acute coronary syndrome beyond a rule-out troponin profile. *Eur Heart J Acute Cardiovasc Care* 2021;10(9):1038-47. doi: 10.1093/ehjacc/zuab040

210. Khoshnood A, Erlandsson M, Isma N, et al. Diagnostic accuracy of troponin T measured >/=6h after symptom onset for ruling out myocardial infarction. *Scand Cardiovasc J* 2020;54(3):153-61. doi: 10.1080/14017431.2019.1699248

211. Kienbacher CL, Fuhrmann V, van Tulder R, et al. Impact of more conservative European Society of Cardiology guidelines on the management of patients with acute chest pain. *Int J Clin Pract* 2021;75(6):e14133. doi: 10.1111/ijcp.14133

212. Kim J, Gaddam S, Wu WC, et al. Stratified reporting of high sensitivity troponin I assay is associated with suboptimal management of patients with acute coronary syndrome and intermediate troponin elevation. *J Clin Lab Anal* 2013;27(5):402-6. doi: 10.1002/jcla.21618

213. Kimenai DM, Lindahl B, Jernberg T, et al. Sex-specific effects of implementing a high-sensitivity troponin I assay in patients with suspected acute coronary syndrome: results from SWEDEHEART registry. *Sci Rep* 2020;10(1):15227. doi: 10.1038/s41598-020-72204-2

214. Kitamura M, Hata N, Takayama T, et al. High-sensitivity cardiac troponin T for earlier diagnosis of acute myocardial infarction in patients with initially negative troponin T test--comparison between cardiac markers. *J Cardiol* 2013;62(6):336-42. doi: 10.1016/j.jjcc.2013.06.005

215. Kremneva L, Suplotov S, Shalaev S. Estimation of highly sensitive troponin tests in the diagnosis of acute coronary syndrome. *Ration Pharmacother Cardiol* 2016;12(2) doi: 10.20996/1819-6446-2016-12-2-204-209

216. Krintus M, Kozinski M, Boudry P, et al. European multicenter analytical evaluation of the Abbott ARCHITECT STAT high sensitive troponin I immunoassay. *Clin Chem Lab Med* 2014;52(11):1657-65. doi: 10.1515/cclm-2014-0107

217. Kumar A, Swamy R, Ramaswamy. Study on conventional and novel cardiac biomarkers in acute myocardial infarction. *Asian J Pharm Clin Res* 2016;9(2):327-31.

218. Lambrakis K, Papendick C, French JK, et al. Late Outcomes of the RAPID-TnT Randomized Controlled Trial: 0/1-Hour High-Sensitivity Troponin T Protocol in Suspected ACS. *Circulation* 2021;144(2):113-25. doi: 10.1161/CIRCULATIONAHA.121.055009

219. Lee H, Kerr D, O'H Ici D, et al. Clinical Significance of Initial Troponin I in the Grey Zone in Emergency Department Chest Pain Patients: A Retrospective Pilot Study. *Emerg Med J* 2010;27(4):302-4. doi: 10.1136/emj.2009.077669

220. Lehmacher J, Neumann JT, Sorensen NA, et al. Predictive Value of Serial ECGs in Patients with Suspected Myocardial Infarction. *J Clin Med* 2020;9(7) doi: 10.3390/jcm9072303

221. Li WJ, Chen XM, Nie XY, et al. Early diagnostic and prognostic utility of high-sensitive troponin assays in acute myocardial infarction: a meta-analysis. *Intern Med J* 2015;45(7):748-56. doi: 10.1111/imj.12642

222. Lim KD, Yan AT, Casanova A, et al. Quantitative troponin elevation does not provide incremental prognostic value

beyond comprehensive risk stratification in patients with non-ST-segment elevation acute coronary syndromes. *Am Heart J* 2008;155(4):718-24. doi: 10.1016/j.ahj.2007.11.012

223. Lin S, Yokoyama H, Rac VE, et al. Novel biomarkers in diagnosing cardiac ischemia in the emergency department: a systematic review. *Resuscitation* 2012;83(6):684-91. doi: 10.1016/j.resuscitation.2011.12.015

224. Lindahl B, Venge P, James S. The new high-sensitivity cardiac troponin T assay improves risk assessment in acute coronary syndromes. *Am Heart J* 2010;160(2):224-9. doi: 10.1016/j.ahj.2010.05.023

225. Love SA, Sandoval Y, Smith SW, et al. Incidence of Undetectable, Measurable, and Increased Cardiac Troponin I Concentrations Above the 99th Percentile Using a High-Sensitivity vs a Contemporary Assay in Patients Presenting to the Emergency Department. *Clin Chem* 2016;62(8):1115-9. doi: 10.1373/clinchem.2016.256305

226. Lynn JS, Singh A, Snoey ER. Can We Exclude the Diagnosis of Non-ST Segment Myocardial Infarction on the Basis of a Single Troponin I and a Symptom Duration >/=8 Hours? *ISRN Cardiol* 2011;2011:364728. doi: 10.5402/2011/364728

227. Maag R, Sun S, Hannon M, et al. Positive predictive value of an elevated cardiac troponin for type I myocardial infarction in ED patients based on the chief complaint. *Am J Emerg Med* 2015;33(4):516-20. doi: 10.1016/j.ajem.2015.01.003

228. Macrae AR, Kavsak PA, Lustig V, et al. Assessing the requirement for the 6-hour interval between specimens in the American Heart Association Classification of Myocardial Infarction in Epidemiology and Clinical Research Studies. *Clin Chem* 2006;52(5):812-8. doi: 10.1373/clinchem.2005.059550

229. Madsen T, Stewart M, Smyres C, et al. Significance of an Indeterminate Troponin I in Patients Evaluated for Chest Pain in an Emergency Department Observation Unit. *Crit Pathw Cardiol* 2015;14(4):146-9. doi: 10.1097/HPC.0000000000000054

230. Mahler S, Miller C, Hollander J, et al. Identifying patients for early discharge: performance of decision rules among patients with acute chest pain. *Int J Cardiol* 2013;168(2):795-802. doi: 10.1016/j.ijcard.2012.10.010

231. Marjot J, Kaier TE, Henderson K, et al. A single centre prospective cohort study addressing the effect of a rule-in/rule-out troponin algorithm on routine clinical practice. *Eur Heart J Acute Cardiovasc Care* 2019;8(5):404-11. doi: 10.1177/2048872617746850

232. Markota A, Bernhardt M, Palfy M. Comparison of point-of-care and laboratory troponin I assays. *Zdravniški vestnik2011* 2011;80(12):905-8.

233. Masotti S, Prontera C, Musetti V, et al. Evaluation of analytical performance of a new high-sensitivity immunoassay for cardiac troponin I. *Clin Chem Lab Med* 2018;56(3):492-501. doi: 10.1515/cclm-2017-0387

234. Bohula May EA, Bonaca MP, Jarolim P, et al. Prognostic performance of a high-sensitivity cardiac troponin I assay in patients with non-ST-elevation acute coronary syndrome. *Clin Chem* 2014;60(1):158-64. doi: 10.1373/clinchem.2013.206441

235. McRae A, Andruchow J. Highly-sensitive troponin T algorithm facilitates early discharge of low-risk chest pain patients within 1 h of emergency department arrival. *Evid Based Med* 2015;20(4):144. doi: 10.1136/ebmed-2015-110224

236. Melki D, Lind S, Agewall S, et al. Diagnostic value of high sensitive troponin T in chest pain patients with no persistent ST-elevations. *Scand Cardiovasc J* 2011;45(4):198-204. doi: 10.3109/14017431.2011.565792

237. Melki D, Lind S, Agewall S, et al. Prognostic value of combining high sensitive troponin T and N-terminal pro B-type natriuretic peptide in chest pain patients with no persistent ST-elevation. *Clin Chim Acta* 2012;413(9-10):933-7. doi: 10.1016/j.cca.2012.02.008
238. Melki D, Lugnegard J, Alfredsson J, et al. Implications of Introducing High-Sensitivity Cardiac Troponin T Into Clinical Practice: Data From the SWEDEHEART Registry. *J Am Coll Cardiol* 2015;65(16):1655-64. doi: 10.1016/j.jacc.2015.02.044
239. Meune C, Balmelli C, Twerenbold R, et al. Patients with acute coronary syndrome and normal high-sensitivity troponin. *Am J Med* 2011;124(12):1151-7. doi: 10.1016/j.amjmed.2011.07.032
240. Meune C, Reichlin T, Irfan A, et al. How safe is the outpatient management of patients with acute chest pain and mildly increased cardiac troponin concentrations? *Clin Chem* 2012;58(5):916-24. doi: 10.1373/clinchem.2011.178053
241. Miller C, Fermann G, Lindsell C, et al. Initial risk stratification and presenting characteristics of patients with evolving myocardial infarctions. *Emerg Med J* 2008;25(8):492-7. doi: 10.1136/emj.2007.052183
242. Mills N, Churchhouse A, Lee K, et al. Implementation of a sensitive troponin I assay and risk of recurrent myocardial infarction and death in patients with suspected acute coronary syndrome. *JAMA* 2011;305(12):1210-6. doi: 10.1001/jama.2011.338
243. Mills NL, Lee KK, McAllister DA, et al. Implications of lowering threshold of plasma troponin concentration in diagnosis of myocardial infarction: cohort study. *BMJ* 2012;344:e1533. doi: 10.1136/bmj.e1533
244. Le Moal E, Giuliani I, Bertinchant JP, et al. Earlier detection of myocardial infarction by an improved cardiac TnI assay. *Clin Biochem* 2007;40(13-14):1065-73. doi: 10.1016/j.clinbiochem.2007.05.004
245. Mohsen M, Shawky A. The diagnostic utility of High-Sensitivity Cardiac Troponin T in acute coronary syndrome. *The Egyptian Heart Journal* 2016;68(1):1-9. doi: 10.1016/j.ehj.2014.12.003
246. Mokhtari A, Lindahl B, Smith J, et al. Diagnostic Accuracy of High-Sensitivity Cardiac Troponin T at Presentation Combined With History and ECG for Ruling Out Major Adverse Cardiac Events. *Ann Emerg Med* 2016;68(6):649-58.e3. doi: 10.1016/j.annemergmed.2016.06.008
247. Velilla Moliner J, Gros Bañeres B, Povar Marco J, et al. Diagnostic performance of high sensitivity troponin in non-ST elevation acute coronary syndrome. *Medicina Intensiva (English Edition)* 2020;44(2):88-95. doi: 10.1016/j.medine.2019.11.001
248. Mueller M, Celik S, Biener M, et al. Diagnostic and prognostic performance of a novel high-sensitivity cardiac troponin T assay compared to a contemporary sensitive cardiac troponin I assay in patients with acute coronary syndrome. *Clin Res Cardiol* 2012;101(10):837-45. doi: 10.1007/s00392-012-0469-6
249. Mueller M, Biener M, Vafaie M, et al. Absolute and relative kinetic changes of high-sensitivity cardiac troponin T in acute coronary syndrome and in patients with increased troponin in the absence of acute coronary syndrome. *Clin Chem* 2012;58(1):209-18. doi: 10.1373/clinchem.2011.171827
250. Mueler C. Use of high-sensitivity troponin for the diagnosis of acute myocardial infarction. *Coron Artery Dis* 2013;24(8):710-2. doi: 10.1097/MCA.0000000000000049
251. Mueller M, Biener M, Vafaie M, et al. Prognostic performance of kinetic changes of high-sensitivity troponin T in acute coronary syndrome and in patients with increased troponin without acute coronary syndrome. *Int J Cardiol*

2014;174(3):524-9. doi: 10.1016/j.ijcard.2014.04.110

252. Mueller-Hennessen M, Mueller C, Giannitsis E, et al. Serial Sampling of High-Sensitivity Cardiac Troponin T May Not Be Required for Prediction of Acute Myocardial Infarction Diagnosis in Chest Pain Patients with Highly Abnormal Concentrations at Presentation. *Clin Chem* 2017;63(2):542-51. doi: 10.1373/clinchem.2016.258392
253. Mungai E, Hamilton B, Burns D. Comparison of High-Sensitivity Troponin T Assay to Conventional Troponin T Assay for Rule Out of Acute Coronary Syndrome in the Emergency Department. *Adv Emerg Nurs J* 2020;42(4):304-14.
254. Nacke L, Blanchet J, Reyes G, et al. Effectiveness of different cutoff points of high-sensitivity troponin T to diagnose myocardial infarction. *Revista de la Federacion Argentina de Cardiologia* 2014;43(3):141-45.
255. Nadarajah R, Gale C. The management of acute coronary syndromes in patients presenting without persistent ST-segment elevation: key points from the ESC 2020 Clinical Practice Guidelines for the general and emergency physician. *Clin Med (Lond)* 2021;21(2):e206-e11. doi: 10.7861/clinmed.2020-0879
256. Narain S, Gupta N, Sethi R, et al. Clinical Correlation of Multiple Biomarkers for Risk Assessment in Patients With Acute Coronary Syndrome. *Indian Heart J* 2008;60(6):536-42.
257. Nejatian A, Omstedt A, Hoijer J, et al. Outcomes in Patients With Chest Pain Discharged After Evaluation Using a High-Sensitivity Troponin T Assay. *J Am Coll Cardiol* 2017;69(21):2622-30. doi: 10.1016/j.jacc.2017.03.586
258. Nestelberger T, Wildi K, Boeddinghaus J, et al. Characterization of the observe zone of the ESC 2015 high-sensitivity cardiac troponin 0h/1h-algorithm for the early diagnosis of acute myocardial infarction. *Int J Cardiol* 2016;207:238-45. doi: 10.1016/j.ijcard.2016.01.112
259. Nestelberger T, Boeddinghaus J, Lopez-Ayala P, et al. Cardiovascular Biomarkers in the Early Discrimination of Type 2 Myocardial Infarction. *JAMA Cardiol* 2021;6(7):771-80. doi: 10.1001/jamacardio.2021.0669
260. Nestelberger T, Lopez-Ayala P, Boeddinghaus J, et al. External Validation and Extension of a Clinical Score for the Discrimination of Type 2 Myocardial Infarction. *J Clin Med* 2021;10(6) doi: 10.3390/jcm10061264
261. Neumann JT, Sorensen NA, Ojeda F, et al. Immediate Rule-Out of Acute Myocardial Infarction Using Electrocardiogram and Baseline High-Sensitivity Troponin I. *Clin Chem* 2017;63(1):394-402. doi: 10.1373/clinchem.2016.262659
262. Nilsson T, Johannesson E, Lundager Forberg J, et al. Diagnostic accuracy of the HEART Pathway and EDACS-ADP when combined with a 0-hour/1-hour hs-cTnT protocol for assessment of acute chest pain patients. *Emerg Med J* 2021;38(11):808-13. doi: 10.1136/emermed-2020-210833
263. Normann J, Mueller M, Biener M, et al. Effect of older age on diagnostic and prognostic performance of high-sensitivity troponin T in patients presenting to an emergency department. *Am Heart J* 2012;164(5):698-705 e4. doi: 10.1016/j.ahj.2012.08.003
264. Nowak RM, Jacobsen G, Limkakeng A, Jr., et al. Outpatient versus observation/inpatient management of emergency department patients rapidly ruled-out for acute myocardial infarction: Findings from the HIGH-US study. *Am Heart J* 2021;231:6-17. doi: 10.1016/j.ahj.2020.10.067
265. Olivieri F, Galeazzi R, Giavarina D, et al. Aged-related increase of high sensitive Troponin T and its implication in acute myocardial infarction diagnosis of elderly patients. *Mech Ageing Dev* 2012;133(5):300-5. doi: 10.1016/j.mad.2012.03.005

266. Omland T. New Sensitive Cardiac Troponin Assays for the Early Diagnosis of Myocardial Infarction. *Drugs Today (Barc)* 2011;47(4):303-12. doi: 10.1358/dot.2011.47.4.1533082

267. Osredkar J, Krivic K, Fabjan T, et al. Point-of-care high-sensitivity assay on PATHFAST as the backup in the emergency room. *Medicine Access @ Point of Care* 2021;5 doi: 10.1177/23992026211055095

268. Palamalai V, Murakami MM, Apple FS. Diagnostic performance of four point of care cardiac troponin I assays to rule in and rule out acute myocardial infarction. *Clin Biochem* 2013;46(16-17):1631-5. doi: 10.1016/j.clinbiochem.2013.06.026

269. Paoloni R, Kumar P, Janu M. Pilot study of high-sensitivity troponin T testing to facilitate safe early disposition decisions in patients presenting to the emergency department with chest pain. *Intern Med J* 2010;40(3):188-92. doi: 10.1111/j.1445-5994.2009.01962.x

270. Papendick C, Blyth A, Seshadri A, et al. A randomized trial of a 1-hour troponin T protocol in suspected acute coronary syndromes: Design of the Rapid Assessment of Possible ACS In the emergency Department with high sensitivity Troponin T (RAPID-TnT) study. *Am Heart J* 2017;190:25-33. doi: 10.1016/j.ahj.2017.05.004

271. Parikh RH, Seliger SL, de Lemos J, et al. Prognostic Significance of High-Sensitivity Cardiac Troponin T Concentrations between the Limit of Blank and Limit of Detection in Community-Dwelling Adults: A Metaanalysis. *Clin Chem* 2015;61(12):1524-31. doi: 10.1373/clinchem.2015.244160

272. Patsias I, Swanson B, Hudson M, et al. he (Dis) Utility of a Change in Troponin I for Diagnosis of Non-ST-Segment Elevation Myocardial Infarction in an Observation Unit. *Crit Pathw Cardiol* 2017;16(3):105-08. doi: 10.1097/HPC.0000000000000120

273. Peck D, Knott J, Lefkovits J. Clinical impact of a high-sensitivity troponin assay introduction on patients presenting to the emergency department. *Emerg Med Australas* 2016;28(3):273-8. doi: 10.1111/1742-6723.12566

274. Pickering JW, Flaws D, Smith SW, et al. A Risk Assessment Score and Initial High-sensitivity Troponin Combine to Identify Low Risk of Acute Myocardial Infarction in the Emergency Department. *Acad Emerg Med* 2018;25(4):434-43. doi: 10.1111/acem.13343

275. Pickering JW, Young JM, George PM, et al. Early kinetic profiles of troponin I and T measured by high-sensitivity assays in patients with myocardial infarction. *Clin Chim Acta* 2020;505:15-25. doi: 10.1016/j.cca.2020.02.009

276. Puelacher C, Gugala M, Adamson PD, et al. Incidence and outcomes of unstable angina compared with non-ST-elevation myocardial infarction. *Heart* 2019;105(18):1423-31. doi: 10.1136/heartjnl-2018-314305

277. Rao M, Panduranga P, Al-Mukhaini M, et al. Predictive value of a 4-hour accelerated diagnostic protocol in patients with suspected ischemic chest pain presenting to an emergency department. *Oman Med J* 2012;27(3):207-11. doi: 10.5001/omj.2012.47

278. Reichlin T, Hochholzer W, Bassetti S, et al. Early diagnosis of myocardial infarction with sensitive cardiac troponin assays. *N Engl J Med* 2009;361(9):858-67. doi: 10.1056/NEJMoa0900428

279. Reichlin T, Twerenbold R, Maushart C, et al. Risk stratification in patients with unstable angina using absolute serial changes of 3 high-sensitive troponin assays. *Am Heart J* 2013;165(3):371-8 e3. doi: 10.1016/j.ahj.2012.11.010

280. Reiter M, Reichlin T, Twerenbold R, et al. Diagnosis of acute myocardial infarction using highly sensitive cardiac troponin assays. *Eur Cardiol* 2011;7(1):18-20. doi: 10.15420/ecr.2011.7.1.18

281. Ren D, Huang T, Liu X, et al. High-sensitive cardiac troponin for the diagnosis of acute myocardial infarction in

different chronic kidney disease stages. *BMC Cardiovasc Disord* 2021;21(1):100. doi: 10.1186/s12872-020-01746-0

282. Renaud B, Maison P, Ngako A, et al. Impact of point-of-care testing in the emergency department evaluation and treatment of patients with suspected acute coronary syndromes. *Acad Emerg Med* 2008;15(3):216-24. doi: 10.1111/j.1553-2712.2008.00069.x
283. Roberts A. Biomarkers: hs-cTnT measurement to rule out MI. *Nat Rev Cardiol* 2014;11(6):311. doi: 10.1038/nrcardio.2014.52
284. Roos A, Bandstein N, Lundback M, et al. Stable High-Sensitivity Cardiac Troponin T Levels and Outcomes in Patients With Chest Pain. *J Am Coll Cardiol* 2017;70(18):2226-36. doi: 10.1016/j.jacc.2017.08.064
285. Roos A, Hellgren A, Rafatnia F, et al. Investigations, findings, and follow-up in patients with chest pain and elevated high-sensitivity cardiac troponin T levels but no myocardial infarction. *Int J Cardiol* 2017;232:111-16. doi: 10.1016/j.ijcard.2017.01.044
286. Röttger E, de Vries-Spithoven S, Reitsma J, et al. Safety of a 1-hour Rule-out High-sensitive Troponin T Protocol in Patients With Chest Pain at the Emergency Department. *Crit Pathw Cardiol* 2017;16(4):129-34. doi: 10.1097/HPC.0000000000000135
287. Rubini Gimenez M, Hoeller R, Reichlin T, et al. Rapid rule out of acute myocardial infarction using undetectable levels of high-sensitivity cardiac troponin. *Int J Cardiol* 2013;168(4):3896-901. doi: 10.1016/j.ijcard.2013.06.049
288. Rudolph V, Keller T, Schulz A, et al. Diagnostic and prognostic performance of myeloperoxidase plasma levels compared with sensitive troponins in patients admitted with acute onset chest pain. *Circ Cardiovasc Genet* 2012;5(5):561-8. doi: 10.1161/CIRCGENETICS.111.962290
289. Saad YM, McEwan J, Shugman IM, et al. Use of a high-sensitivity troponin T assay in the assessment and disposition of patients attending a tertiary Australian emergency department: a cross-sectional pilot study. *Emerg Med Australas* 2015;27(5):405-11. doi: 10.1111/1742-6723.12430
290. Saenger A, Korpi-Steiner N, Bryant S, et al. Utilization of a High Sensitive Troponin T Assay Optimizes Serial Sampling in the Diagnosis of Acute Myocardial Infarction Compared to Multiple Contemporary Troponin Assays. *Circ* 2010;122(21):A21588.
291. Saenger AK, Beyrau R, Braun S, et al. Multicenter analytical evaluation of a high-sensitivity troponin T assay. *Clin Chim Acta* 2011;412(9-10):748-54. doi: 10.1016/j.cca.2010.12.034
292. Sanchis J, Bardaji A, Bosch X, et al. Usefulness of high-sensitivity troponin T for the evaluation of patients with acute chest pain and no or minimal myocardial damage. *Am Heart J* 2012;164(2):194-200 e1. doi: 10.1016/j.ahj.2012.05.015
293. Sanchis J, Garcia-Blas S, Mainar L, et al. High-sensitivity versus conventional troponin for management and prognosis assessment of patients with acute chest pain. *Heart* 2014;100(20):1591-6. doi: 10.1136/heartjnl-2013-305440
294. Sanchis J, Abellan L, Garcia-Blas S, et al. Usefulness of delta troponin for diagnosis and prognosis assessment of non-ST-segment elevation acute chest pain. *Eur Heart J Acute Cardiovasc Care* 2016;5(5):399-406. doi: 10.1177/2048872615593534
295. Sandoval Y, Smith SW, Schulz KM, et al. Diagnosis of type 1 and type 2 myocardial infarction using a high-sensitivity cardiac troponin I assay with sex-specific 99th percentiles based on the third universal definition of myocardial

infarction classification system. *Clin Chem* 2015;61(4):657-63. doi: 10.1373/clinchem.2014.236638

296. Sandoval Y, Smith SW, Love SA, et al. Single High-Sensitivity Cardiac Troponin I to Rule Out Acute Myocardial Infarction. *Am J Med* 2017;130(9):1076-83 e1. doi: 10.1016/j.amjmed.2017.02.032
297. Sandoval Y, Smith SW, Shah AS, et al. Rapid Rule-Out of Acute Myocardial Injury Using a Single High-Sensitivity Cardiac Troponin I Measurement. *Clin Chem* 2017;63(1):369-76. doi: 10.1373/clinchem.2016.264523
298. Santalo M, Martin A, Velilla J, et al. Using high-sensitivity troponin T: the importance of the proper gold standard. *Am J Med* 2013;126(8):709-17. doi: 10.1016/j.amjmed.2013.03.003
299. Schonemann-Lund M, Schoos MM, Iversen K, et al. Retrospective Evaluation of Two Fast-track Strategies to Rule Out Acute Coronary Syndrome in a Real-life Chest Pain Population. *J Emerg Med* 2015;49(6):833-42. doi: 10.1016/j.jemermed.2015.06.026
300. Shah ASV, Anand A, Sandoval Y, et al. High-sensitivity cardiac troponin I at presentation in patients with suspected acute coronary syndrome: a cohort study. *The Lancet* 2015;386(10012):2481-88. doi: 10.1016/s0140-6736(15)00391-8
301. Shah AS, Griffiths M, Lee KK, et al. High sensitivity cardiac troponin and the under-diagnosis of myocardial infarction in women: prospective cohort study. *BMJ* 2015;350:g7873. doi: 10.1136/bmj.g7873
302. Shin YS, Ahn S, Kim YJ, et al. External validation of the emergency department assessment of chest pain score accelerated diagnostic pathway (EDACS-ADP). *Am J Emerg Med* 2020;38(11):2264-70. doi: 10.1016/j.ajem.2019.09.019
303. Shortt C, Phan K, Hill SA, et al. An approach to rule-out an acute cardiovascular event or death in emergency department patients using outcome-based cutoffs for high-sensitivity cardiac troponin assays and glucose. *Clin Biochem* 2015;48(4-5):282-7. doi: 10.1016/j.clinbiochem.2014.11.010
304. Shortt C, Ma J, Clayton N, et al. Rule-In and Rule-Out of Myocardial Infarction Using Cardiac Troponin and Glycemic Biomarkers in Patients with Symptoms Suggestive of Acute Coronary Syndrome. *Clin Chem* 2017;63(1):403-14. doi: 10.1373/clinchem.2016.261545
305. Simpson AJ, Potter JM, Koerbin G, et al. Use of observed within-person variation of cardiac troponin in emergency department patients for determination of biological variation and percentage and absolute reference change values. *Clin Chem* 2014;60(6):848-54. doi: 10.1373/clinchem.2013.219410
306. Singer AJ, Than MP, Smith S, et al. Missed myocardial infarctions in ED patients prospectively categorized as low risk by established risk scores. *Am J Emerg Med* 2017;35(5):704-09. doi: 10.1016/j.ajem.2017.01.003
307. Slagman A, von Recum J, Mockel M, et al. Diagnostic performance of a high-sensitive troponin T assay and a troponin T point of care assay in the clinical routine of an Emergency Department: A clinical cohort study. *Int J Cardiol* 2017;230:454-60. doi: 10.1016/j.ijcard.2016.12.085
308. Sorensen NA, Neumann JT, Ojeda F, et al. Challenging the 99th percentile: A lower troponin cutoff leads to low mortality of chest pain patients. *Int J Cardiol* 2017;232:289-93. doi: 10.1016/j.ijcard.2016.12.167
309. Sorensen NA, Ludwig S, Makarova N, et al. Prognostic Value of a Novel and Established High-Sensitivity Troponin I Assay in Patients Presenting with Suspected Myocardial Infarction. *Biomolecules* 2019;9(9) doi: 10.3390/biom9090469
310. Stengaard C, Sorensen JT, Ladefoged SA, et al. Quantitative point-of-care troponin T measurement for diagnosis and

prognosis in patients with a suspected acute myocardial infarction. *Am J Cardiol* 2013;112(9):1361-6. doi: 10.1016/j.amjcard.2013.06.026

311. Stopyra JP, Miller CD, Hiestand BC, et al. Chest Pain Risk Stratification: A Comparison of the 2-Hour Accelerated Diagnostic Protocol (ADAPT) and the HEART Pathway. *Crit Pathw Cardiol* 2016;15(2):46-9. doi: 10.1097/HPC.0000000000000072
312. Stopyra J, Snavely AC, Hiestand B, et al. Comparison of accelerated diagnostic pathways for acute chest pain risk stratification. *Heart* 2020;106(13):977-84. doi: 10.1136/heartjnl-2019-316426
313. Stoyanov KM, Biener M, Hund H, et al. Effects of crowding in the emergency department on the diagnosis and management of suspected acute coronary syndrome using rapid algorithms: an observational study. *BMJ Open* 2020;10(10):e041757. doi: 10.1136/bmjopen-2020-041757
314. Storrow AB, Christenson RH, Nowak RM, et al. Diagnostic performance of cardiac Troponin I for early rule-in and rule-out of acute myocardial infarction: Results of a prospective multicenter trial. *Clin Biochem* 2015;48(4-5):254-9. doi: 10.1016/j.clinbiochem.2014.08.018
315. Storrow AB, Nowak RM, Diercks DB, et al. Absolute and relative changes (delta) in troponin I for early diagnosis of myocardial infarction: Results of a prospective multicenter trial. *Clin Biochem* 2015;48(4-5):260-7. doi: 10.1016/j.clinbiochem.2014.09.012
316. Straface AL, Myers JH, Kirchick HJ, et al. A rapid point-of-care cardiac marker testing strategy facilitates the rapid diagnosis and management of chest pain patients in the emergency department. *Am J Clin Pathol* 2008;129(5):788-95. doi: 10.1309/9GGNMURLJWJD88W3
317. Su Q, Guo Y, Liu H, et al. Diagnostic Role of High-Sensitivity Cardiac Troponin T in Acute Myocardial Infarction and Cardiac Noncoronary Artery Disease. *Arch Med Res* 2015;46(3):193-8. doi: 10.1016/j.arcmed.2015.03.005
318. Suh D, Keller DI, Hof D, et al. Rule-out of non-ST elevation myocardial infarction by five point of care cardiac troponin assays according to the 0 h/3 h algorithm of the European Society of Cardiology. *Clin Chem Lab Med* 2018;56(4):649-57. doi: 10.1515/cclm-2017-0486
319. Suzuki K, Komukai K, Nakata K, et al. The Usefulness and Limitations of Point-of-care Cardiac Troponin Measurement in the Emergency Department. *Intern Med* 2018;57(12):1673-80. doi: 10.2169/internalmedicine.0098-17
320. Takakuwa KM, Ou F-S, Peterson ED, et al. The Usage Patterns of Cardiac Bedside Markers Employing Point-of-Care Testing for Troponin in Non-ST-Segment Elevation Acute Coronary Syndrome: Results from CRUSADE. *Clinical Cardiology* 2009;32(9):498-505. doi: 10.1002/clc.20626
321. Tecson K, Arnold W, Barrett T, et al. Interpretation of positive troponin results among patients with and without myocardial infarction. *Proc (Bayl Univ Med Cent)* 2017;30(1):11-15. doi: 10.1080/08998280.2017.11929513
322. Than M, Cullen L, Reid CM, et al. A 2-h diagnostic protocol to assess patients with chest pain symptoms in the Asia-Pacific region (ASPECT): a prospective observational validation study. *The Lancet* 2011;377(9771):1077-84. doi: 10.1016/s0140-6736(11)60310-3
323. Than M, Cullen L, Aldous S, et al. 2-Hour Accelerated Diagnostic Protocol to Assess Patients With Chest Pain Symptoms Using Contemporary Troponins as the Only Biomarker. *Journal of the American College of Cardiology* 2012;59(23):2091-8. doi: 10.1016/j.jacc.2012.02.035

324. Than M, Aldous S, Lord SJ, et al. A 2-hour diagnostic protocol for possible cardiac chest pain in the emergency department: a randomized clinical trial. *JAMA Intern Med* 2014;174(1):51-8. doi: 10.1001/jamainternmed.2013.11362

325. Thelin J, Melander O, Ohlin B. Early rule-out of acute coronary syndrome using undetectable levels of high sensitivity troponin T. *Eur Heart J Acute Cardiovasc Care* 2015;4(5):403-9. doi: 10.1177/2048872614554107

326. Truong QA, Bayley J, Hoffmann U, et al. Multi-marker strategy of natriuretic peptide with either conventional or high-sensitivity troponin-T for acute coronary syndrome diagnosis in emergency department patients with chest pain: from the "Rule Out Myocardial Infarction using Computer Assisted Tomography" (ROMICAT) trial. *Am Heart J* 2012;163(6):972-79 e1. doi: 10.1016/j.ahj.2012.03.010

327. Twerenbold R, Wildi K, Jaeger C, et al. Optimal Cutoff Levels of More Sensitive Cardiac Troponin Assays for the Early Diagnosis of Myocardial Infarction in Patients With Renal Dysfunction. *Circulation* 2015;131(23):2041-50. doi: 10.1161/CIRCULATIONAHA.114.014245

328. Vafaie M, Biener M, Mueller M, et al. Analytically false or true positive elevations of high sensitivity cardiac troponin: a systematic approach. *Heart* 2014;100(6):508-14. doi: 10.1136/heartjnl-2012-303202

329. van der Laarse A, Cobbaert CM, Gorgels AP, et al. Will future troponin measurement overrule the ECG as the primary diagnostic tool in patients with acute coronary syndrome? *J Electrocardiol* 2013;46(4):312-7. doi: 10.1016/j.jelectrocard.2013.02.007

330. Van Hise CB, Greenslade JH, Parsonage W, et al. External validation of heart-type fatty acid binding protein, high-sensitivity cardiac troponin, and electrocardiography as rule-out for acute myocardial infarction. *Clin Biochem* 2018;52:161-63. doi: 10.1016/j.clinbiochem.2017.10.001

331. Vasile VC, Jaffe AS. High-Sensitivity Cardiac Troponin for the Diagnosis of Patients with Acute Coronary Syndromes. *Curr Cardiol Rep* 2017;19(10):92. doi: 10.1007/s11886-017-0904-4

332. Vasudevan A, Singer AJ, DeFilippi C, et al. Renal Function and Scaled Troponin in Patients Presenting to the Emergency Department with Symptoms of Myocardial Infarction. *Am J Nephrol* 2017;45(4):304-09. doi: 10.1159/000458451

333. Venge P, Johnston N, Lindahl B, et al. Normal plasma levels of cardiac troponin I measured by the high-sensitivity cardiac troponin I access prototype assay and the impact on the diagnosis of myocardial ischemia. *J Am Coll Cardiol* 2009;54(13):1165-72. doi: 10.1016/j.jacc.2009.05.051

334. Venge P, James S, Jansson L, et al. Clinical performance of two highly sensitive cardiac troponin I assays. *Clin Chem* 2009;55(1):109-16. doi: 10.1373/clinchem.2008.106500

335. Wang W, Huang H, Zhu S, et al. High-Sensitivity Cardiac Troponin T in Patients with Acute Myocardial Infarction in Acute Exacerbation of Chronic Obstructive Pulmonary Disease. *Clin lab* 2015;61(8):1083-93.

336. Wang JA, Qin Y, Lv J, et al. Clinical application of high-sensitivity cardiac troponin T test in acute myocardial infarction diagnosis. *Genet Mol Res* 2015;14(4):17959-65. doi: 10.4238/2015.December.22.21

337. Wassef AW, Hiebert B, Saeed MF, et al. Novel high-sensitivity troponin assay requires higher cut-off value to separate acute myocardial infarction from non-acute myocardial infarction in a high-risk population. *Can J Physiol Pharmacol* 2015;93(10):873-7. doi: 10.1139/cjpp-2014-0473

338. Wassie M, Lee MS, Sun BC, et al. Single vs Serial Measurements of Cardiac Troponin Level in the Evaluation of

Patients in the Emergency Department With Suspected Acute Myocardial Infarction. *JAMA Netw Open* 2021;4(2):e2037930. doi: 10.1001/jamanetworkopen.2020.37930

339. Weber M, Bazzino O, Navarro Estrada JL, et al. Improved diagnostic and prognostic performance of a new high-sensitive troponin T assay in patients with acute coronary syndrome. *Am Heart J* 2011;162(1):81-8. doi: 10.1016/j.ahj.2011.04.007
340. Wereski R, Kimenai DM, Taggart C, et al. Cardiac Troponin Thresholds and Kinetics to Differentiate Myocardial Injury and Myocardial Infarction. *Circulation* 2021;144(7):528-38. doi: 10.1161/CIRCULATIONAHA.121.054302
341. Westwood M, van Asselt T, Ramaekers B, et al. High-sensitivity troponin assays for the early rule-out or diagnosis of acute myocardial infarction in people with acute chest pain: a systematic review and cost-effectiveness analysis. *Health Technol Assess* 2015;19(44):1-234. doi: 10.3310/hta19440
342. Wildi K, Reichlin T, Twerenbold R, et al. Serial changes in high-sensitivity cardiac troponin I in the early diagnosis of acute myocardial infarction. *Int J Cardiol* 2013;168(4):4103-10. doi: 10.1016/j.ijcard.2013.07.078
343. Willeit P, Welsh P, Evans JDW, et al. High-Sensitivity Cardiac Troponin Concentration and Risk of First-Ever Cardiovascular Outcomes in 154,052 Participants. *J Am Coll Cardiol* 2017;70(5):558-68. doi: 10.1016/j.jacc.2017.05.062
344. Wolf S, Kaur R, McKeown W, et al. Noise Versus Signal: The Clinical Implications of an Increasingly Sensitive Troponin Assay for Patients With Suspected Acute Coronary Syndrome. *Crit Pathw Cardiol* 2014;13(3):89-95. doi: 10.1097/HPC.0000000000000020
345. Wong P, Rao G, Innasimuthu A, et al. Validation of a prediction score model to distinguish acute coronary syndromes from other conditions causing raised cardiac troponin T levels. *Coron Artery Dis* 2010;21(6):363-8. doi: 10.1097/MCA.0b013e32833d18d8
346. Yang HS, Shemesh A, Li J, et al. No increase in the incidence of cardiac troponin I concentration above the 99th percentile by Siemens Centaur high-sensitivity compared to the contemporary assay. *Clin Biochem* 2021;89:77-80. doi: 10.1016/j.clinbiochem.2020.12.001
347. Yean KS, Abd. Wahab MB, Zakaria MIB. A study on modified accelerated diagnostic protocol to safely discharge low-risk chest pain patients in emergency department. *Hong Kong Journal of Emergency Medicine* 2019;27(3):134-45. doi: 10.1177/1024907918820750
348. Yokoyama H, Higuma T, Endo T, et al. "30-minute-delta" of high-sensitivity troponin I improves diagnostic performance in acute myocardial infarction. *J Cardiol* 2018;71(2):144-48. doi: 10.1016/j.jjcc.2017.08.003
349. Zhao Y, Sivaswamy A, Lee MK, et al. A feasibility study for CODE-MI: High-sensitivity cardiac troponin-Optimizing the diagnosis of acute myocardial infarction/injury in women. *Am Heart J* 2021;234:60-70. doi: 10.1016/j.ahj.2021.01.008
350. Advantageous Predictors of Acute Coronary Syndromes Evaluation (APACE) Study (APACE) [Available from: https://www.clinicaltrials.gov/ct2/show/NCT00470587 accessed 16 May 2020.
351. Ghali W, Knudtson M. Overview of the Alberta Provincial Project for Outcome Assessment in Coronary Heart Disease. On behalf of the APPROACH investigators. *Can J Cardiol* 2000;16(10):1225-30.
352. Biomarkers in Acute Cardiac Care (BACC) [Available from: https://clinicaltrials.gov/ct2/show/NCT02355457

accessed 2nd August 2020.

353. Than MP, Pickering JW, Aldous SJ, et al. Effectiveness of EDACS Versus ADAPT Accelerated Diagnostic Pathways for Chest Pain: A Pragmatic Randomized Controlled Trial Embedded Within Practice. *Ann Emerg Med* 2016;68(1):93-102 e1. doi: 10.1016/j.annemergmed.2016.01.001

354. High-Sensitivity Troponin in the Evaluation of Patients With Acute Coronary Syndrome (High-STEACS) [Available from: https://clinicaltrials.gov/ct2/show/NCT01852123 accessed 2nd August 2020.

355. Cullen L, Greenslade JH, Hawkins T, et al. Improved Assessment of Chest pain Trial (IMPACT): assessing patients with possible acute coronary syndromes. *Med J Aust* 2017;207(5):195-200. doi: 10.5694/mja16.01351

356. Kavsak P, Worster A, You J, et al. Ninety-minute vs 3-h performance of high-sensitivity cardiac troponin assays for predicting hospitalization for acute coronary syndrome. *Clin Chem* 2013;59(9):1407–10. doi: 10.1373/clinchem.2013.208595

357. Use of Abbott High Sensitivity Troponin I Assay In Acute Coronary Syndromes (UTROPIA) [Available from: https://clinicaltrials.gov/ct2/show/NCT02060760 accessed 2nd August 2020.
