# Supplementary material for: Serial high-sensitivity cardiac troponin testing for the diagnosis of myocardial infarction: a scoping review
Source: BMJ Open. 2022 Nov 22;12(11):e066429. doi: 10.1136/bmjopen-2022-066429 (PMC9685223; doi:10.1136/bmjopen-2022-066429)
Supplement: Author's manuscript [file bmjopen-2022-066429.draft_revisions.pdf]

BMJ Open is committed to open peer review. As part of this commitment we make the peer review history of every article we publish publicly available.

When an article is published we post the peer reviewers' comments and the authors' responses online. We also post the versions of the paper that were used during peer review. These are the versions that the peer review comments apply to.

The versions of the paper that follow are the versions that were submitted during the peer review process. They are not the versions of record or the final published versions. They should not be cited or distributed as the published version of this manuscript.

BMJ Open is an open access journal and the full, final, typeset and author-corrected version of record of the manuscript is available on our site with no access controls, subscription charges or pay-per-view fees (http://bmjopen.bmj.com).

If you have any questions on BMJ Open's open peer review process please email info.bmjopen@bmj.com

BMJ Open

Serial high-sensitivity cardiac troponin testing for the diagnosis of myocardial infarction: A scoping review

Journal:	*BMJ Open*
Manuscript ID	bmjopen-2022-066429
Article Type:	Original research
Date Submitted by the Author:	12-Jul-2022
Complete List of Authors:	Ohtake, Hirotaka; Fujita Health University, Department of Emergency and General Internal Medicine<br>Terasawa, Teruhiko; Fujita Health University, Department of Emergency and General Internal Medicine<br>Zhelev, Zhivko; University of Exeter, University of Exeter Medical School<br>Iwata, Mitsunaga; Fujita Health University, Department of Emergency and General Internal Medicine<br>Rogers, Morwenna; University of Exeter, NIHR CLAHRC South West Peninsula<br>Peters, Jaime; University of Exeter, Peninsula Technology Assessment Group (PenTAG); University of Exeter, Peninsula Technology Assessment Group (PenTAG)<br>Hyde, Chris; University of Exeter, Exeter Test Group
Keywords:	Coronary heart disease < CARDIOLOGY, Ischaemic heart disease < CARDIOLOGY, Myocardial infarction < CARDIOLOGY, Cardiology < INTERNAL MEDICINE

SCHOLARONE™
Manuscripts

I, the Submitting Author has the right to grant and does grant on behalf of all authors of the Work (as defined in the below author licence), an exclusive licence and/or a non-exclusive licence for contributions from authors who are: i) UK Crown employees; ii) where BMJ has agreed a CC-BY licence shall apply, and/or iii) in accordance with the terms applicable for US Federal Government officers or employees acting as part of their official duties; on a worldwide, perpetual, irrevocable, royalty-free basis to BMJ Publishing Group Ltd ("BMJ") its licensees and where the relevant Journal is co-owned by BMJ to the co-owners of the Journal, to publish the Work in this journal and any other BMJ products and to exploit all rights, as set out in our licence.

The Submitting Author accepts and understands that any supply made under these terms is made by BMJ to the Submitting Author unless you are acting as an employee on behalf of your employer or a postgraduate student of an affiliated institution which is paying any applicable article publishing charge ("APC") for Open Access articles. Where the Submitting Author wishes to make the Work available on an Open Access basis (and intends to pay the relevant APC), the terms of reuse of such Open Access shall be governed by a Creative Commons licence – details of these licences and which Creative Commons licence will apply to this Work are set out in our licence referred to above.

Other than as permitted in any relevant BMJ Author's Self Archiving Policies, I confirm this Work has not been accepted for publication elsewhere, is not being considered for publication elsewhere and does not duplicate material already published. I confirm all authors consent to publication of this Work and authorise the granting of this licence.

For peer review only

Serial high-sensitivity cardiac troponin testing for the diagnosis of myocardial infarction: A scoping review

Hirotaka Ohtake[1], Teruhiko Terasawa[1], Zhivko Zhelev[2], Mitsunaga Iwata[1], Morwenna Rogers[3], Jaime L Peters[2], Chris Hyde[2]

[1] Department of Emergency and General Internal Medicine, Fujita Health University School of Medicine, Toyoake, Japan

[2] Exeter Test Group, University of Exeter Medical School, University of Exeter, Exeter, Devon, UK

[3] NIHR ARC South West Peninsula, University of Exeter Medical School, Exeter, UK.

Corresponding author: Teruhiko Terasawa, MD, PhD; Department of Emergency and General Internal Medicine, Fujita Health University School of Medicine; 1-98 Dengakugakubo, Kutsukake-cho, Toyoake, Aichi 470-1192, Japan; terasawa@fujita-hu.ac.jp

For peer review only - http://bmjopen.bmj.com/site/about/guidelines.xhtml

Abstract

***Objectives**:* We aimed to assess the diversity and practices of existing studies on several assays and algorithms for serial measurements of high-sensitivity cardiac troponin (hs-cTn) for risk stratification and the diagnosis of myocardial infarction (MI) and 30-day outcomes in patients suspected of having non-ST-segment elevation MI (NSTEMI).

Methods: We searched multiple databases for studies published between January 2006 and November 2021. Studies that assessed the diagnostic accuracy of serial hs-cTn testing in patients suspected of having NSTEMI in the emergency department (ED) were eligible. Data were analysed using the scoping review method.

Results: We included 86 publications mainly from research centres in Europe, North America, and Australasia. Two hs-cTn assays, manufactured by Abbott (43/86) and Roche (53/86), dominated the evaluations. The studies most commonly measured the concentrations of hs-cTn at two time points, at presentation and a few hours thereafter, to assess the two- or three-strata algorithm for diagnosing or ruling out MI. Although data from 83 studies (97%) were prospectively collected, 0 to 90% of the eligible patients were excluded from the analysis due to missing blood samples or the lack of a final diagnosis in 53 studies (62%) that reported relevant data. Only 19 studies (22%) reported on head-to-head comparisons of alternative assays.

Conclusion: Evidence on the accuracy of serial hs-cTn testing was largely derived from selected research institutions and relied on two specific assays. The proportions of the eligible patients excluded from the study raise concerns about directly applying the study findings to clinical practice in frontline EDs.

For peer review only

For peer review only - http://bmjopen.bmj.com/site/about/guidelines.xhtml

Strengths and limitations of this study

- This is the first scoping review that explored the diversity of study methodologies adopted in studies on testing with serial high-sensitivity cardiac troponin measurements in patients with suspected non-ST elevation myocardial infarction in the emergency department.
- The review method included a comprehensive literature search, duplicate assessment of eligibility, double extraction of data, and descriptive synthesis through tables and graphs.
- The assessed specifications included the regions and sources of studies, targeted participants and their study flow, troponin assays, sampling algorithms and direct comparisons thereof, and definitions of outcomes.
- The quantitative results about the accuracy and clinical outcomes were not assessed because this was beyond the scope of the review.

For peer review only - http://bmjopen.bmj.com/site/about/guidelines.xhtml

INTRODUCTION

Acute coronary syndrome (ACS) is considered a major cause of death worldwide.[1 2] Acute myocardial infarction (AMI) is a form of ACS, which represents permanent cellular damage in the affected myocardium due to ischemia. AMI is clinically subcategorized into ST-segment elevation myocardial infarction (STEMI) and non-ST-segment elevation myocardial infarction (NSTEMI). Each type of AMI has a unique prognosis, and their managements differ substantially. Since STEMI is an acute life-threatening condition, prompt reperfusion therapy is essential. In contrast, because the prognosis of NSTEMI varies depending on its etiology, accurate diagnosis and risk stratification based on medical history, electrocardiogram (ECG) findings, and cardiac biomarker concentrations are of paramount significance in patients suspected of ACS.[3 4]

For the clinical management of NSTEMI, cardiac troponin (cTn) has been used as the mainstay of clinical diagnosis since 2000.[5-8] To avoid unnecessary hospital admissions and expedite the diagnostic process, high-sensitivity cardiac Tn (hs-cTn), a group of more sensitive cTn assays,[9] has been introduced into clinical practice since 2010. Although several primary studies and meta-analyses on the single measurement of hs-cTn reported their high sensitivity and specificity,[10-12] several challenges persist. First, blood concentrations of hs-cTn troponin take two to three hours to increase, and they may not be detectable within three hours from the onset of AMI.[3 13] Second, despite its high sensitivity, elevated concentrations of hs-cTn are observed in several clinical conditions other than AMI, including acute myocardial injury (e.g. acute heart failure, and tachyarrhythmia) and chronic myocardial injury (e.g. structural heart disease and chronic heart failure).[3 8 13] To differentiate these conditions, serial measurements of hs-

For peer review only - http://bmjopen.bmj.com/site/about/guidelines.xhtml

cTn, i.e., assessing the absolute and/or relative changes of repeated measurements, were proposed to increase the specificity for diagnosing acute MI. Based on several studies on serial hs-cTn testing algorithms with high sensitivity and high negative predictive value, the current clinical guidelines on NSTE-ACS recommend serial measurements of hs-cTn at presentation and after 1-3 hours.[3 4]

However, the comparative effectiveness of management strategies based on serial hs-cTn measurements has not been fully elucidated, because several alternative assays are clinically available and existing reports are from studies with different designs and inconsistent testing algorithms. Thus, this study aimed to explore the diversity of the methodologies used in primary studies on serial hs-cTn measurements in patients suspected of having ACS in the emergency department (ED). We constructed an evidence map of existing studies on serial hs-cTn testing for diagnosing NSTEMI and predicting 30-day clinical outcomes. We critically appraised the currently available evidence and highlighted the issues that need to be addressed in future research.

METHODS

This study is a focused analysis performed in conjunction with a registered systematic review project (PROSPERO registration number CRD42018106379). The protocol for the original systematic review is available at https://bmjopen.bmj.com/content/9/3/e026012.long.[10] Since it was a secondary analysis of publicly available data, no ethical review was required. This report followed the PRISMA Extension for Scoping Reviews.[14]

Data search

For peer review only - http://bmjopen.bmj.com/site/about/guidelines.xhtml

We searched Ovid MEDLINE, EMBASE, Science Citation Index, and Cochrane Database of Systematic Reviews for studies published between 1 January 2006 and 17 November 2021 with no restrictions of language or publication status. The search terms included “chest pain”, “acute coronary syndrome,” “myocardial infarction,” “cardiac troponin”, “emergency room” and their synonyms.[10] The full search strategy is available in **Appendix**. We excluded editorials, letters, comments, conference abstracts, review articles, and meta-analyses. Also, we excluded studies assessing clinical prediction rules (e.g., Global Registry of Acute Coronary Events (GRACE) Risk Score[15]).

Study eligibility

We included prospective and retrospective studies that evaluated patients aged ≥18 years who were suspected of having NSTEMI in an ED and had two or more serial troponin measurements using an hs-cTn assay. Eligible were studies that reported the diagnostic accuracy of AMI and/or 30-day clinical outcomes. We only included single-gate design studies, i.e., studies that consisted of a single group of subjects based on a single eligibility criteria.[16] Studies that included mixed populations of patients—with suspected STEMI and NSTEMI—were included only when data for the patients with suspected NSTEMI were separately extractable. Studies that exclusively assessed patients with suspected STEMI were excluded. Two investigators double-screened the titles and abstracts and examined the full-text articles to assess eligibility. We defined hs-cTn as assays that satisfied the requirements of the International Clinical Federation of Clinical Chemistry and Laboratory Medicine (i.e., <10% coefficient of variation at the 99th percentile, and ≥ 50% measurable concentrations above the limit of detection for both males and females).[9] Discrepancies were resolved by consensus.

For peer review only - http://bmjopen.bmj.com/site/about/guidelines.xhtml

Data extraction

The following data were extracted: 1) publication and study characteristics: authors, journal name, publication year, enrolment years, number of eligible and included patients, study design, the name of the study cohort(s), geographical region(s), participant age and use of ECG to exclude patients; 2) test characteristics: assays, the timing of blood sampling, cut-off values, algorithms adopted (binary testing algorithms for ruling out MI *vs.* three-strata testing algorithms for stratifying patients into three different risk groups, i.e., high-, intermediate-, and low-risk groups commonly referred to as "rule-in," "observational zone," and "rule-out" for MI diagnosis); 3) reference standard characteristics: specific diagnostic criteria of MI, such as those defined in clinical guidelines and/or versions of the universal definition of MI, and the assessors of the final diagnoses.

Operationalisation

Our target population was a group of patients suspected of having NSTEMI who presented at the ED. We recorded the numbers of patients suspected of having NSTEMI who presented at the ED, patients who completed one or more study-specific serial testing algorithm(s), and patients who were assessed for test accuracy and/or 30-day clinical outcomes. A testing algorithm was specified based on the number and timing of hs-cTn measurements. The results for the algorithm were typically reported as a single value measured at presentation, together with an absolute or a relative difference between specific measurement time points (typically at presentation and a few hours later), which was categorised as delta or percent change in the hs-cTn concentrations.

For peer review only - http://bmjopen.bmj.com/site/about/guidelines.xhtml

We classified the studies that involved two hs-cTn measurements into three groups, namely, 0 and 1h, 0 and 2h, and 0 and 3h, based on the blood sampling timing (in hours) of the first and second samples. Other algorithms involving three or more blood samples were grouped into a separate category, labelled as “others.” Assays were classified by specific manufacturers, i.e., Abbott (Abbott Laboratories, Illinois, USA), Roche (Roche Diagnostics, Basel, Switzerland), Siemens (Siemens Healthcare, Erlangen, Germany), and Beckman (Beckman Coulter, California, USA). Assays by other manufacturers were categorised as “miscellaneous.”

To assess the evidence, we used the study with the largest sample to avoid double-counting when multiple studies reported (partially) overlapping patient populations. For the study locations, we assumed that each specific research institution involved in the study assessed patients residing within its geographical region only. Comparative studies were defined as studies that adopted a paired design to assess multiple assays on the same study participants and directly compared the diagnostic accuracy for AMI or 30-day clinical outcomes. This review did not standardise the definition of AMI or the 30-day clinical outcomes and adopted the study-reported outcome definitions as specifically reported.

Analyses

We considered each publication to be the unit of analysis and performed descriptive analyses using percentages or medians and ranges. We combined data as a weighted average only if the pertinent data were available for specific subgroups. The assessed design specifications included the regions and sources of studies, characteristics of

For peer review only - http://bmjopen.bmj.com/site/about/guidelines.xhtml

targeted participants and their study flow, specific troponin assays used, sampling algorithms with their operational characteristics, direct comparisons of two or more algorithms and/or assays, and definitions of the index MI and 30-day outcomes.

The volume of clinical evidence was assessed with graphs and tables using Stata 17.0 (Stata Corp. College Station, TX, USA). The graphical presentation of the study locations was constructed using Google Maps (Google Inc., Mountain View, CA, USA) and Mapcustomizer (available at https://www.mapcustomizer.com/).

Patient and public involvement

We did not involve patients or the public in the preparation of this scoping review.

RESULTS

Inclusion of primary studies

Figure 1 shows the PRISMA flow diagram for this scoping review. Our search yielded 6838 articles; 6230 of them were excluded after examining the titles and abstracts. After excluding 549 perused full-text articles, we finally included 86 publications, including 72 reports on test accuracy and 52 on 30-day clinical outcomes (**Appendix Table 1**).[17-102] The excluded articles are listed in **Appendix Table 2**.

Study characteristics

Eighty-three studies (97%) were based on prospectively collected blood samples, and the median sample size was 1391 (range, 160-6403) (**Appendix Table 1**). The most commonly assessed assays were manufactured by Abbot (n=41) and Roche (n=53),

For peer review only - http://bmjopen.bmj.com/site/about/guidelines.xhtml

followed by those manufactured by Siemens (n=10) and Beckman (n=6). Three studies assessed point-of-care assays that met the definition of hs-cTn.[76 80 102] Serial hs-cTn testing was predominantly assessed in Europe and North America and the participating institutions were limited to specific research centres (**Appendix Table 1**, **Appendix Table 3**, **and Appendix Figure 1**). In contrast, only a few research centres per country from Australasia and Asia participated and provided pertinent data.

For peer review only

Of the 86 cohorts that reported accuracy and/or 30-day outcome data using a specific assay and a sampling algorithm, only 42 (49%) were considered unique, which included 78,606 non-overlapping patients (**Appendix Table 3**). Of the 42 studies, 12 (29%) studies that assessed the assays manufactured by Abbott involved unique cohorts (seven, three, and eight reports on the 0 and 1h, 0 and 2h, and 0 and 3h protocols, respectively). Similarly, data from 25 of 42 studies (60%) that assessed the assays manufactured by Roche involved unique cohorts (16, five, and four reports on the 0 and 1h, 0 and 2h, and 0 and 3h protocols, respectively).

Nineteen studies compared two or more assays using a paired design (**Table 1**). The most commonly reported comparison was between the assays by Abbott and Roche (17 studies on test accuracy and six on 30-day outcomes). Few studies have performed head-to-head comparisons involving the same patients. Two studies compared hs-cTn with an earlier generation non-hs cTn assay.[17 51]

Table 1. Direct comparisons of alternative high-sensitivity cardiac troponin assays*

**Hs-cTn assays**	**Abbott**	**Roche**	**Siemens**	**Beckman**
**0 and 1h protocol**				
**Abbott**		6 (10731)	0	0

For peer review only - http://bmjopen.bmj.com/site/about/guidelines.xhtml

**Roche**	8 (13779)		0	0
**Siemens**	2 (1235)	1 (418)		0
**Beckman**	1 (278)	1 (278)	1 (278)	
0 and 2h protocol				
Abbott		2 (4063)	0	1 (1118)
Roche	5 (7497)		0	1 (1118)
Siemens	1 (313)	1 (313)		0
Beckman	1 (1118)	1 (1118)	1 (217)	
0 and 3h protocol				
Abbott		1 (2945)	0	0
Roche	4 (6419)		0	0
Siemens	1 (1809)	1 (1809)		0
Beckman	1 (1110)	1 (1110)	1 (1110)	
Miscellaneous				
Abbott		0	0	0
Roche	1 (1735)		0	0
Siemens	0	1 (830)		0
Beckman	0	1 (830)	1 (830)	

* For each algorithm (i.e., 0 and 1h, 0 and 2h, and 0 and 3h protocols and a combined, miscellaneous protocol group described in the left-most column), cells in the lower left and upper right parts (separated by a right-lower diagonal line comprising black closed cells) show the volume of comparative evidence on diagnostic accuracy of acute myocardial infarction and 30-day clinical outcomes, respectively. Each cell represents a specific comparison between assays described in the leftmost column and the top row. The number of comparative studies that compared a specific pair of assays is followed by the number of assessed patients in parentheses.
Hs-cTn = high-sensitivity cardiac troponin

Typically, the studies reported only the number of enrolled patients (i.e., patients suspected of having NSTEMI who were eligible for and agreed to participate in the study), and the complete data on all clinically relevant patients (i.e., the number of all patients suspected of having NSTEMI who presented at the ED) were missing. Fifty-two studies (60%) reported quantitative data on patients who failed to complete a study-

For peer review only

specific serial testing algorithm(s) or patients whose diagnosis or 30-day clinical outcomes could not be established (**Appendix Table 1**). Various proportions (median 20%; range, 0%-90%) of enrolled patients were excluded from the analysis, typically due to missing blood samples or the lack of a final diagnosis.

Patient characteristics

The mean or median participant age ranged from 53 to 73 years, and most studies involved patients in their 40s to 70s (**Appendix Table 1**). Only one study[56] specifically focused on 0 and 1h protocol for a subgroup of elderly patients derived from three cohorts, specifically, patients aged 70 years or older. Of 86 studies, 17 (20%) excluded patients with chronic kidney disease (CKD) or those requiring regular haemodialysis. In contrast, only four studies specifically focused on 0 and 1h, 3h or 6-12h protocol for a subgroup of patients with renal dysfunction, which were derived from four cohorts.[61 62 64 65] Only a single study specifically focused on 0 and 3h protocol for a subgroup of female patients only derived from three cohorts. No further studies that focused on these sub populations have been found through the update search and manual search based on the reference lists.[52]

Testing algorithms

Forty-seven studies (55%) assessed the three-strata algorithms. These studies stratified patients into "rule-out (low-risk)," "observational zone (intermediate-risk)," and "rule-in (high-risk)" groups according to two sets of diagnostic criteria based on the concentrations of hs-cTn at baseline, 1-3 hours, and/or the difference between the hs-cTn concentrations of the two samples. Other studies conventionally categorised the

For peer review only - http://bmjopen.bmj.com/site/about/guidelines.xhtml

patients into two strata (i.e. "rule-out (low-risk)" and "rule-in (high-risk)" groups) based on a single set of criteria. The majority of data were based on assays manufactured by either Abbott or Roche, and the 0 and 1h algorithm was the most frequently reported (**Figure 2**).

Outcomes

Eighty-four studies (98%) adopted the universal definition of MI (versions 2007, 2012, and/or 2018)[6-8] to establish a diagnosis of AMI. A few of these studies also followed the guidelines proposed by the American College of Cardiology (ACC) (1/86, 1%),[103] the ACC and the American Heart Association (AHA) guidelines (1/86, 1%),[104] ACC and the European Society of Cardiology (ESC)[105], and the ACC/AHA[104 106] and ESC[3 107] guidelines (4/86, 5%) in addition to either version of the universal definition. Two studies[17 21] relied on the ACC guidelines[103] alone. The adjudicators of the clinical diagnosis of AMI were cardiologists in 72 studies (84%). The studies variably reported 30-day outcomes; of the 51 studies that reported one or more 30-day outcomes, 11 (22%) reported all-cause mortality, and 41 (80%) reported cardiac death, whereas 36 (68%) reported major adverse cardiac events, a composite outcome including AMI, as well as cardiac death or death from all causes. Other reported clinical outcomes observed within 30 days included urgent revascularization, percutaneous coronary intervention, coronary artery bypass graft (19/51, 37%), and ventricular arrhythmia (11/51, 22%). Twenty-seven studies (53%) also reported long-term clinical outcomes, which included events that developed within up to two years.[34-37 45 57 62 65 75 94 95]

Discussion

For peer review only - http://bmjopen.bmj.com/site/about/guidelines.xhtml

To the best of our knowledge, this is the first scoping review to comprehensively assess the reports on testing with serial hs-cTn measurements in patients with suspected NSTEMI in the ED. We summarised how studies measured serial hs-cTn and assessed its diagnostic accuracy for AMI and the 30-day clinical outcomes. Our results showed that most existing data were based on the Abbott cTnI or Roche cTnT assays, and the timing of the blood measurements and the diagnostic algorithms varied. The number of studies assessing these two assays using the 0 and 1h, 0 and 2h, and 0 and 3h protocols has been continuously increasing since 2011 when guidelines[29] recommended serial hs-cTn measurements; fewer studies have assessed other assays and/or alternative algorithms. Limited data on patients with CKD or older adults, as well as data stratified by sex, were reported,[62 64] which were deemed still under evaluation. Most studies followed the universal definition to diagnose the index MI. However, in addition to North America or Europe, only a limited number of research teams involving several specialty institutions in specific countries have contributed to the current evidence. Most importantly, the studies excluded variable proportions of eligible patients from the analysis due to missing blood samples or concrete final diagnoses.

Strengths

We comprehensively explored the existing evidence, focusing on how the studies were designed, analysed, and reported and, the implications for clinical practice of the identified limitations and concerns. Previously reported systematic reviews focused on a two-strata rule-out strategy using unique serial measurements of hs-cTnT assay only[108] or the 0 and 1h three-strata strategy only[109] regardless of the assays assessed, and they

For peer review only - http://bmjopen.bmj.com/site/about/guidelines.xhtml

did not perform a comprehensive field synopsis covering all relevant information. The objective of our scoping review is to describe the diversity in the adopted study methodologies together with their potential limitations following the standard scoping review methods. Therefore, this review should be an additional view that follows the recently published critical appraisal of the current evidence base,[110 111] both of which will help identify the current evidence gaps as well as help design future studies.

Limitation

This scoping review performed a focused analysis on the reported study methodologies and did not address the primary objectives of the originally planned systematic review. Therefore, several limitations need to be discussed. First, we did not assess the quantitative results on accuracy and other clinical outcomes because this was beyond the scope of the present scoping review. Second, we focused on studies that assessed only test accuracy and 30-day outcomes. Therefore, data on studies that evaluated long-term outcomes have not been addressed. Third, since we excluded conference abstracts, this review may have missed newer relevant publications. Fourth, the recently developed clinical prediction rules (e.g., History, ECG, Age, Risk factors and Troponin (HEART) Score,[112] Emergency Department Assessment of Chest Pain (EDACS) Score[113], and GRACE Risk Score[15]) have consistently incorporated hs-cTn concentrations as a component variable.[40] We excluded these studies that used hs-cTn as part of risk prediction models. Finally, several studies have reported that higher hs-cTn concentrations were associated with increased mortality in patients with suspected ACS; however, our review did not address this association.[34 36 37 45 57 62 65]

For peer review only - http://bmjopen.bmj.com/site/about/guidelines.xhtml

Clinical implications

Recently, a pooled study conducted by Neumann et al. proposed a high-accurate risk-assessment tool for NSTEMI using a serial hs-cTn measurements based on an international consortium of prospectively registered data from 15 cohorts. Of these, nine cohorts were also assessed in this scoping review, at least some portions of which should have contributed to the dataset deriving the risk-assessment tool. Given the methodological concerns raised in this review, depending on how the study methodologies in the rest of the data have been addressed and/or improved, uncertainties still exist in its individualized application in real-life clinical practice. First, ACS and MI are common in elderly populations as well as patients with CKD,[114 115] while the eligible studies in our review mainly focused on middle-aged populations, and approximately a third of the studies excluded patients with impaired renal function. Additionally, hs-cTnI concentrations are generally higher in men than in women,[116] which bases the use of sex-specific cut-offs recommended by the fourth Universal definition of MI in contrast to the uniform cut-off adopted in the risk-assessment tool.[117] The recent critical appraisal report[111] is also in line with this individualized approach. Therefore, the optimal sample timings and cut-off values need to be validated on more individualized bases taking account of age, sex, and renal function.[118 119] Second, our review found that the existing data were largely based on two assays by two manufacturers (i.e., assays manufactured by Abbott and Roche); evidence is sparse for the others. Third, most of the included studies were conducted in specialised centres in Europe, North America, or Australasia. A recent meta-analysis of diagnostic accuracy that focused on only the 0 and 1h algorithm[109] pointed out that sensitivity was not universally high across cohorts, as reported in the primary studies in these specialised

For peer review only - http://bmjopen.bmj.com/site/about/guidelines.xhtml

centres; reproducibility of the excellent results appeared to be limited for the studies from Asia. Given this observation, validation in these regions is required. Fourth, the studies included in our review missed variable proportions of clinically relevant patients who presented at EDs with suspected NSTEMI. This appears to stem, at least in part, from convenience sampling. The failure to apply gold standard tests to all participants, may also have been responsible for the excluded cases without an established diagnosis of the cause of chest pain, which is inevitable in real-life clinical settings. These methodological weaknesses would have distorted, at least to some extent, the typical disease spectrum of clinically relevant populations. Our review failed to address how this patient loss affected the study results.

CONCLUSIONS

Data on diagnostic test accuracy and short-term outcomes by serial hs-cTn measurements were largely derived from particular research institutions in Europe, North America, or Australasia and based mainly on two specific assays. The exclusion of variable proportions of eligible patients, which was inevitable even in well-conducted prospective studies, raised concerns regarding the studies' generalizability and direct applications in real-world ED clinical practice.

For peer review only - http://bmjopen.bmj.com/site/about/guidelines.xhtml

Acknowledgments

The authors thank Drs Chihiro Kato and Jun Shinohara for assisting with data extraction.

Contributors

TT and ZZ lead the protocol development. HO, TT, ZZ, MI, MR, JLP, and CH drafted and revised the protocol. MR performed the literature searches. HO, TT, ZZ, JLP determined the eligibility of primary studies and acquired the data. HO and TT analysed the data. HO, TT, ZZ, MI, MR, JLP, and CH interpreted the findings. HO and TT drafted the first version of the report. HO, TT, ZZ, MI, MR, JLP, and CH critically read the manuscript and provided feedback for revision. HO, TT, ZZ, MI, MR, JLP, and CH read and approved the final manuscript.

Competing interests

The authors declare no conflicts of interest associated with this study.

Funding

This study was supported in part by a research grant from the Ministry of Education, Culture, Sports, Science, and Technology (MEXT) of Japan (18K08902) and by the National Institute for Health Research (NIHR) Collaboration for Leadership in Applied Health Research and Care South West Peninsula (NIHR CLAHRC South West Peninsula; grant no, N/A). The funders had no involvement in the study design, data collection and analysis, decision to publish, or preparation of the manuscript. The views expressed in this publication are those of the author(s) and not necessarily those of

For peer review only - http://bmjopen.bmj.com/site/about/guidelines.xhtml

MEXT in Japan, the National Institute for Health Research, or the Department of Health and Social Care.

Ethics statements

Patient consent for publication

Not required.

Ethics approval

Since this scoping review was a secondary analysis of publicly available data, no ethical review was required.

Data Availability Statement

All data relevant to the study are included in the article or uploaded as supplementary information.

ORCID iDs

Hirotaka Ohtake: https://orcid.org/0000-0002-0223-4789
Teruhiko Terasawa: https://orcid.org/0000-0002-0975-391X
Zhivko Zhelev: https://orcid.org/0000-0002-0106-2401
Mitsunaga Iwata: https://orcid.org/0000-0001-8979-4801
Morwenna Rogers: https://orcid.org/0000-0002-6039-238X
Jaime L Peters: https://orcid.org/0000-0003-1778-3518
Chris Hyde: https://orcid.org/0000-0002-7349-0616

For peer review only

REFERENCES

1. Organization WH. Cardiovascular diseases (CVDs): Key facts 2017 [Available from: https://www.who.int/en/news-room/fact-sheets/detail/cardiovascular-diseases-(cvds) accessed 10 May 2020.
2. Reed GW, Rossi JE, Cannon CP. Acute myocardial infarction. *The Lancet* 2017;389(10065):197-210. doi: 10.1016/s0140-6736(16)30677-8
3. Collet JP, Thiele H, Barbato E, et al. 2020 ESC Guidelines for the management of acute coronary syndromes in patients presenting without persistent ST-segment elevation. *Eur Heart J* 2021;42(14):1289-367. doi: 10.1093/eurheartj/ehaa575
4. Amsterdam EA, Wenger NK, Brindis RG, et al. 2014 AHA/ACC Guideline for the Management of Patients with Non-ST-Elevation Acute Coronary Syndromes: a report of the American College of Cardiology/American Heart Association Task Force on Practice Guidelines. *J Am Coll Cardiol* 2014;64(24):e139-e228. doi: 10.1016/j.jacc.2014.09.017
5. Alpert J, Thygesen K, Antman E, et al. Myocardial infarction redefined—a consensus document of The Joint European Society of Cardiology/American College of Cardiology committee for the redefinition of myocardial infarction. *J Am Coll Cardiol* 2000;36(3):959-69. doi: 10.1016/s0735-1097(00)00804-4
6. Thygesen K, Alpert JS, White HD, et al. Universal definition of myocardial infarction. *Circulation* 2007;116(22):2634-53. doi: 10.1161/CIRCULATIONAHA.107.187397
7. Thygesen K, Alpert JS, Jaffe AS, et al. Third universal definition of myocardial infarction. *Circulation* 2012;126(16):2020-35. doi: 10.1161/CIR.0b013e31826e1058
8. Thygesen K, Alpert JS, Jaffe AS, et al. Fourth universal definition of myocardial infarction (2018). *Eur Heart J* 2019;40(3):237-69. doi: 10.1093/eurheartj/ehy462
9. High-Sensitivity* Cardiac Troponin I and T Assay Analytical Characteristics Designated by Manufacturer IFCC Committee on Clinical Applications of Cardiac Bio-Markers [Available from: https://www.ifcc.org/media/477656/high-sensitivity-cardiac-troponin-i-and-t-assay-analytical-characteristics-designated-by-manufacturer-v012019.pdf.
10. Zhelev Z, Ohtake H, Iwata M, et al. Diagnostic accuracy of contemporary and high-sensitivity cardiac troponin assays used in serial testing, versus single-sample testing as a comparator, to triage patients suspected of acute non-ST-segment

For peer review only - http://bmjopen.bmj.com/site/about/guidelines.xhtml

elevation myocardial infarction: a systematic review protocol. *BMJ Open* 2019;9(3):e026012. doi: 10.1136/bmjopen-2018-026012

11. Pickering JW, Than MP, Cullen L, et al. Rapid Rule-out of Acute Myocardial Infarction With a Single High-Sensitivity Cardiac Troponin T Measurement Below the Limit of Detection: A Collaborative Meta-analysis. *Ann Intern Med* 2017;166(10):715-24. doi: 10.7326/M16-2562
12. Chapman AR, Lee KK, McAllister DA, et al. Association of High-Sensitivity Cardiac Troponin I Concentration With Cardiac Outcomes in Patients With Suspected Acute Coronary Syndrome. *JAMA* 2017;318(19):1913-24. doi: 10.1001/jama.2017.17488
13. Weber M, Bazzino O, Navarro Estrada JL, et al. Improved diagnostic and prognostic performance of a new high-sensitive troponin T assay in patients with acute coronary syndrome. *Am Heart J* 2011;162(1):81-8. doi: 10.1016/j.ahj.2011.04.007
14. Tricco AC, Lillie E, Zarin W, et al. PRISMA Extension for Scoping Reviews (PRISMA-ScR): Checklist and Explanation. *Ann Intern Med* 2018;169(7):467-73. doi: 10.7326/M18-0850
15. Fox KA, Dabbous OH, Goldberg RJ, et al. Prediction of risk of death and myocardial infarction in the six months after presentation with acute coronary syndrome: prospective multinational observational study (GRACE). *BMJ* 2006;333(7578):1091. doi: 10.1136/bmj.38985.646481.55
16. Rutjes AW, Reitsma JB, Vandenbroucke JP, et al. Case-control and two-gate designs in diagnostic accuracy studies. *Clin Chem* 2005;51(8):1335-41. doi: 10.1373/clinchem.2005.048595
17. Aldous SJ, Florkowski CM, Crozier IG, et al. Comparison of high sensitivity and contemporary troponin assays for the early detection of acute myocardial infarction in the emergency department. *Ann Clin Biochem* 2011;48(Pt 3):241-8. doi: 10.1258/acb.2010.010219
18. Aldous SJ, Richards AM, Cullen L, et al. Early dynamic change in high-sensitivity cardiac troponin T in the investigation of acute myocardial infarction. *Clin Chem* 2011;57(8):1154-60. doi: 10.1373/clinchem.2010.161166
19. Keller T, Zeller T, Ojeda F, et al. Serial Changes in Highly Sensitive Troponin I Assay and Early Diagnosis of Myocardial Infarction. *JAMA* 2011;306(24):2684-93. doi: 10.1001/jama.2011.1896
20. Reichlin T, Irfan A, Twerenbold R, et al. Utility of absolute and relative changes in cardiac troponin concentrations in the early diagnosis of acute myocardial

For peer review only - http://bmjopen.bmj.com/site/about/guidelines.xhtml

infarction. *Circulation* 2011;124(2):136-45. doi: 10.1161/CIRCULATIONAHA.111.023937

21. Aldous S, Pemberton C, Richards AM, et al. High-sensitivity troponin T for early rule-out of myocardial infarction in recent onset chest pain. *Emerg Med J* 2012;29(10):805-10. doi: 10.1136/emermed-2011-200222
22. Reichlin T, Schindler C, Drexler B, et al. One-hour rule-out and rule-in of acute myocardial infarction using high-sensitivity cardiac troponin T. *Arch Intern Med* 2012;172(16):1211-8. doi: 10.1001/archinternmed.2012.3698
23. Schreiber DH, Agbo C, Wu AH. Short-term (90 min) diagnostic performance for acute non-ST segment elevation myocardial infarction and 30-day prognostic evaluation of a novel third-generation high sensitivity troponin I assay. *Clin Biochem* 2012;45(16-17):1295-301. doi: 10.1016/j.clinbiochem.2012.06.005
24. Cullen L, Mueller C, Parsonage WA, et al. Validation of high-sensitivity troponin I in a 2-hour diagnostic strategy to assess 30-day outcomes in emergency department patients with possible acute coronary syndrome. *J Am Coll Cardiol* 2013;62(14):1242-49. doi: 10.1016/j.jacc.2013.02.078
25. Irfan A, Reichlin T, Twerenbold R, et al. Early diagnosis of myocardial infarction using absolute and relative changes in cardiac troponin concentrations. *Am J Med* 2013;126(9):781-88 e2. doi: 10.1016/j.amjmed.2013.02.031
26. Aldous S, Mark Richards A, George PM, et al. Comparison of new point-of-care troponin assay with high sensitivity troponin in diagnosing myocardial infarction. *Int J Cardiol* 2014;177(1):182-6. doi: 10.1016/j.ijcard.2014.09.026
27. Cullen L, Aldous S, Than M, et al. Comparison of high sensitivity troponin T and I assays in the diagnosis of non-ST elevation acute myocardial infarction in emergency patients with chest pain. *Clin Biochem* 2014;47(6):321-6. doi: 10.1016/j.clinbiochem.2013.11.019
28. Rubini Gimenez M, Twerenbold R, Reichlin T, et al. Direct comparison of high-sensitivity-cardiac troponin I vs. T for the early diagnosis of acute myocardial infarction. *Eur Heart J* 2014;35(34):2303-11. doi: 10.1093/eurheartj/ehu188
29. Parsonage WA, Greenslade JH, Hammett CJ, et al. Validation of an accelerated high-sensitivity troponin T assay protocol in an Australian cohort with chest pain. *Med J Aust* 2014;200(3):161-5. doi: 10.5694/mja13.10466
30. Biener M, Mueller M, Vafaie M, et al. Impact of leading presenting symptoms on the diagnostic performance of high-sensitivity cardiac troponin T and on outcomes in patients with suspected acute coronary syndrome. *Clin Chem* 2015;61(5):744-51. doi: 10.1373/clinchem.2014.235317

For peer review only - http://bmjopen.bmj.com/site/about/guidelines.xhtml

31. Eggers KM, Aldous S, Greenslade JH, et al. Two-hour diagnostic algorithms for early assessment of patients with acute chest pain--Implications of lowering the cardiac troponin I cut-off to the 97.5th percentile. *Clin Chim Acta* 2015;445:19-24. doi: 10.1016/j.cca.2015.03.002
32. Rubini Gimenez M, Twerenbold R, Jaeger C, et al. One-hour rule-in and rule-out of acute myocardial infarction using high-sensitivity cardiac troponin I. *Am J Med* 2015;128(8):861-70 e4. doi: 10.1016/j.amjmed.2015.01.046
33. Pickering JW, Young JM, George P, et al. The utility of presentation and 4-hour high sensitivity troponin I to rule-out acute myocardial infarction in the emergency department. *Clin Biochem* 2015;48(18):1219-24. doi: 10.1016/j.clinbiochem.2015.07.033
34. Reichlin T, Twerenbold R, Wildi K, et al. Prospective validation of a 1-hour algorithm to rule-out and rule-in acute myocardial infarction using a high-sensitivity cardiac troponin T assay. *CMAJ* 2015;187(8):E243-52. doi: 10.1503/cmaj.141349/-/DC1
35. Reichlin T, Cullen L, Parsonage WA, et al. Two-hour algorithm for triage toward rule-out and rule-in of acute myocardial infarction using high-sensitivity cardiac troponin T. *Am J Med* 2015;128(4):369-79 e4. doi: 10.1016/j.amjmed.2014.10.032
36. Jaeger C, Wildi K, Twerenbold R, et al. One-hour rule-in and rule-out of acute myocardial infarction using high-sensitivity cardiac troponin I. *Am Heart J* 2016;171(1):92-102 e1-5. doi: 10.1016/j.ahj.2015.07.022
37. Boeddinghaus J, Reichlin T, Cullen L, et al. Two-Hour Algorithm for Triage toward Rule-Out and Rule-In of Acute Myocardial Infarction by Use of High-Sensitivity Cardiac Troponin I. *Clin Chem* 2016;62(3):494-504. doi: 10.1373/clinchem.2015.249508
38. Mokhtari A, Borna C, Gilje P, et al. A 1-h Combination Algorithm Allows Fast Rule-Out and Rule-In of Major Adverse Cardiac Events. *J Am Coll Cardiol* 2016;67(13):1531-40. doi: 10.1016/j.jacc.2016.01.059
39. Mueller C, Giannitsis E, Christ M, et al. Multicenter Evaluation of a 0-Hour/1-Hour Algorithm in the Diagnosis of Myocardial Infarction With High-Sensitivity Cardiac Troponin T. *Ann Emerg Med* 2016;68(1):76-87 e4. doi: 10.1016/j.annemergmed.2015.11.013
40. Neumann JT, Sorensen NA, Schwemer T, et al. Diagnosis of Myocardial Infarction Using a High-Sensitivity Troponin I 1-Hour Algorithm. *JAMA Cardiol* 2016;1(4):397-404. doi: 10.1001/jamacardio.2016.0695

For peer review only

41. Parsonage WA, Mueller C, Greenslade JH, et al. Validation of NICE diagnostic guidance for rule out of myocardial infarction using high-sensitivity troponin tests. *Heart* 2016;102(16):1279-86. doi: 10.1136/heartjnl-2016-309270

42. Pickering JW, Greenslade JH, Cullen L, et al. Assessment of the European Society of Cardiology 0-Hour/1-Hour Algorithm to Rule-Out and Rule-In Acute Myocardial Infarction. *Circulation* 2016;134(20):1532-41. doi: 10.1161/CIRCULATIONAHA.116.022677

43. Pickering JW, Greenslade JH, Cullen L, et al. Validation of presentation and 3 h high-sensitivity troponin to rule-in and rule-out acute myocardial infarction. *Heart* 2016;102(16):1270-8. doi: 10.1136/heartjnl-2015-308505

44. Wildi K, Nelles B, Twerenbold R, et al. Safety and efficacy of the 0 h/3 h protocol for rapid rule out of myocardial infarction. *Am Heart J* 2016;181:16-25. doi: 10.1016/j.ahj.2016.07.013

45. Boeddinghaus J, Nestelberger T, Twerenbold R, et al. Direct Comparison of 4 Very Early Rule-Out Strategies for Acute Myocardial Infarction Using High-Sensitivity Cardiac Troponin I. *Circulation* 2017;135(17):1597-611. doi: 10.1161/CIRCULATIONAHA.116.025661

46. Chapman AR, Anand A, Boeddinghaus J, et al. Comparison of the Efficacy and Safety of Early Rule-Out Pathways for Acute Myocardial Infarction. *Circulation* 2017;135(17):1586-96. doi: 10.1161/CIRCULATIONAHA.116.025021

47. Lindahl B, Jernberg T, Badertscher P, et al. An algorithm for rule-in and rule-out of acute myocardial infarction using a novel troponin I assay. *Heart* 2017;103(2):125-31. doi: 10.1136/heartjnl-2016-309951

48. McRae AD, Innes G, Graham M, et al. Comparative Evaluation of 2-Hour Rapid Diagnostic Algorithms for Acute Myocardial Infarction Using High-Sensitivity Cardiac Troponin T. *Can J Cardiol* 2017;33(8):1006-12. doi: 10.1016/j.cjca.2017.04.010

49. Mokhtari A, Lindahl B, Schiopu A, et al. A 0-Hour/1-Hour Protocol for Safe, Early Discharge of Chest Pain Patients. *Acad Emerg Med* 2017;24(8):983-92. doi: 10.1111/acem.13224

50. Neumann JT, Sorensen NA, Ojeda F, et al. Early diagnosis of acute myocardial infarction using high-sensitivity troponin I. *PLoS One* 2017;12(3):e0174288. doi: 10.1371/journal.pone.0174288

51. Sandoval Y, Smith SW, Thordsen SE, et al. Diagnostic Performance of High Sensitivity Compared with Contemporary Cardiac Troponin I for the Diagnosis

For peer review only

For peer review only - http://bmjopen.bmj.com/site/about/guidelines.xhtml

of Acute Myocardial Infarction. *Clin Chem* 2017;63(10):1594-604. doi: 10.1373/clinchem.2017.272930

52. Schofer N, Brunner F, Schlüter M, et al. Gender-specific diagnostic performance of a new highsensitivity cardiac troponin I assay for detection of acute myocardial infarction. *Eur Heart J* 2017;6(1):60-68. doi: 10.1177/2048872615626660
53. Shiozaki M, Inoue K, Suwa S, et al. Utility of the 0-hour/1-hour high-sensitivity cardiac troponin T algorithm in Asian patients with suspected non-ST elevation myocardial infarction. *Int J Cardiol* 2017;249:32-35. doi: 10.1016/j.ijcard.2017.09.009
54. Wildi K, Cullen L, Twerenbold R, et al. Direct Comparison of 2 Rule-Out Strategies for Acute Myocardial Infarction: 2-h Accelerated Diagnostic Protocol vs 2-h Algorithm. *Clin Chem* 2017;63(7):1227-36. doi: 10.1373/clinchem.2016.268359
55. Peacock WF, Baumann BM, Bruton D, et al. Efficacy of High-Sensitivity Troponin T in Identifying Very-Low-Risk Patients With Possible Acute Coronary Syndrome. *JAMA Cardiol* 2018;3(2):104-11. doi: 10.1001/jamacardio.2017.4625
56. Boeddinghaus J, Nestelberger T, Twerenbold R, et al. Impact of age on the performance of the ESC 0/1h-algorithms for early diagnosis of myocardial infarction. *Eur Heart J* 2018;39(42):3780-94. doi: 10.1093/eurheartj/ehy514
57. Boeddinghaus J, Twerenbold R, Nestelberger T, et al. Clinical Validation of a Novel High-Sensitivity Cardiac Troponin I Assay for Early Diagnosis of Acute Myocardial Infarction. *Clin Chem* 2018;64(9):1347-60. doi: 10.1373/clinchem.2018.286906
58. Borna C, Kollberg K, Larsson D, et al. The objective CORE score allows early rule out in acute chest pain patients. *Scand Cardiovasc J* 2018;52(6):308-14. doi: 10.1080/14017431.2018.1546891
59. Chapman AR, Hesse K, Andrews J, et al. High-Sensitivity Cardiac Troponin I and Clinical Risk Scores in Patients With Suspected Acute Coronary Syndrome. *Circulation* 2018;138(16):1654-65. doi: 10.1161/CIRCULATIONAHA.118.036426
60. Greenslade JH, Carlton EW, Van Hise C, et al. Diagnostic Accuracy of a New High-Sensitivity Troponin I Assay and Five Accelerated Diagnostic Pathways for Ruling Out Acute Myocardial Infarction and Acute Coronary Syndrome. *Ann Emerg Med* 2018;71(4):439-51 e3. doi: 10.1016/j.annemergmed.2017.10.030

For peer review only - http://bmjopen.bmj.com/site/about/guidelines.xhtml

61. Gunsolus I, Sandoval Y, Smith SW, et al. Renal Dysfunction Influences the Diagnostic and Prognostic Performance of High-Sensitivity Cardiac Troponin I. *J Am Soc Nephrol* 2018;29(2):636-43. doi: 10.1681/ASN.2017030341
62. Miller-Hodges E, Anand A, Shah ASV, et al. High-Sensitivity Cardiac Troponin and the Risk Stratification of Patients With Renal Impairment Presenting With Suspected Acute Coronary Syndrome. *Circulation* 2018;137(5):425-35. doi: 10.1161/CIRCULATIONAHA.117.030320
63. Nowak RM, Gandolfo CM, Jacobsen G, et al. Ultrarapid Rule-out for Acute Myocardial Infarction Using the Generation 5 Cardiac Troponin T Assay: Results From the REACTION-US Study. *Ann Emerg Med* 2018;72(6):654-64. doi: 10.1016/j.annemergmed.2018.06.021
64. Twerenbold R, Badertscher P, Boeddinghaus J, et al. 0/1-Hour Triage Algorithm for Myocardial Infarction in Patients With Renal Dysfunction. *Circulation* 2018;137(5):436-51. doi: 10.1161/CIRCULATIONAHA.117.028901
65. Twerenbold R, Neumann JT, Sorensen NA, et al. Prospective Validation of the 0/1-h Algorithm for Early Diagnosis of Myocardial Infarction. *J Am Coll Cardiol* 2018;72(6):620-32. doi: 10.1016/j.jacc.2018.05.040
66. Amann M, Gaiser F, Schwenk SI, et al. Evaluation of a 1-hour troponin algorithm for diagnosing myocardial infarction in high-risk patients admitted to a chest pain unit: the prospective FAST-MI cohort study. *BMJ Open* 2019;9(11):e032124. doi: 10.1136/bmjopen-2019-032124
67. Boeddinghaus J, Twerenbold R, Nestelberger T, et al. Clinical Use of a New High-Sensitivity Cardiac Troponin I Assay in Patients with Suspected Myocardial Infarction. *Clin Chem* 2019;65(11):1426-36. doi: 10.1373/clinchem.2019.304725
68. Cappellini F, Falbo R, Saltafossi D, et al. Development of an algorithm for ruling-out non-ST elevation myocardial infarction in the emergency department using high sensitivity troponin T assay. *Clin Chim Acta* 2019;495:1-7. doi: 10.1016/j.cca.2019.03.1625
69. Chapman AR, Fujisawa T, Lee KK, et al. Novel high-sensitivity cardiac troponin I assay in patients with suspected acute coronary syndrome. *Heart* 2019;105(8):616-22. doi: 10.1136/heartjnl-2018-314093
70. Chew DP, Lambrakis K, Blyth A, et al. A Randomized Trial of a 1-Hour Troponin T Protocol in Suspected Acute Coronary Syndromes: The Rapid Assessment of Possible Acute Coronary Syndrome in the Emergency Department With High-

For peer review only - http://bmjopen.bmj.com/site/about/guidelines.xhtml

Sensitivity Troponin T Study (RAPID-TnT). *Circulation* 2019;140(19):1543-56. doi: 10.1161/CIRCULATIONAHA.119.042891

71. Lin Z, Lim SH, Chua SJT, et al. High-sensitivity troponin T and long-term adverse cardiac events among patients presenting with suspected acute coronary syndrome in Singapore. *Singapore Med J* 2019;60(8):418-26. doi: 10.11622/smedj.2019013
72. Mueller-Hennessen M, Lindahl B, Giannitsis E, et al. Combined testing of copeptin and high-sensitivity cardiac troponin T at presentation in comparison to other algorithms for rapid rule-out of acute myocardial infarction. *Int J Cardiol* 2019;276:261-67. doi: 10.1016/j.ijcard.2018.10.084
73. Nestelberger T, Boeddinghaus J, Wussler D, et al. Predicting Major Adverse Events in Patients With Acute Myocardial Infarction. *J Am Coll Cardiol* 2019;74(7):842-54. doi: 10.1016/j.jacc.2019.06.025
74. Nestelberger T, Boeddinghaus J, Greenslade J, et al. Two-Hour Algorithm for Rapid Triage of Suspected Acute Myocardial Infarction Using a High-Sensitivity Cardiac Troponin I Assay. *Clin Chem* 2019;65(11):1437-47. doi: 10.1373/clinchem.2019.305193
75. Neumann JT, Sorensen NA, Rubsamen N, et al. Evaluation of a new ultra-sensitivity troponin I assay in patients with suspected myocardial infarction. *Int J Cardiol* 2019;283:35-40. doi: 10.1016/j.ijcard.2018.12.001
76. Sorensen NA, Neumann JT, Ojeda F, et al. Diagnostic Evaluation of a High-Sensitivity Troponin I Point-of-Care Assay. *Clin Chem* 2019;65(12):1592-601. doi: 10.1373/clinchem.2019.307405
77. Twerenbold R, Costabel JP, Nestelberger T, et al. Outcome of Applying the ESC 0/1-hour Algorithm in Patients With Suspected Myocardial Infarction. *J Am Coll Cardiol* 2019;74(4):483-94. doi: 10.1016/j.jacc.2019.05.046
78. Wildi K, Boeddinghaus J, Nestelberger T, et al. Comparison of fourteen rule-out strategies for acute myocardial infarction. *Int J Cardiol* 2019;283:41-47. doi: 10.1016/j.ijcard.2018.11.140
79. Andruchow JE, Boyne T, Seiden-Long I, et al. Prospective comparative evaluation of the European Society of Cardiology (ESC) 1-hour and a 2-hour rapid diagnostic algorithm for myocardial infarction using high-sensitivity troponin-T. *CJEM* 2020;22(5):712-20. doi: 10.1017/cem.2020.349
80. Boeddinghaus J, Nestelberger T, Koechlin L, et al. Early Diagnosis of Myocardial Infarction With Point-of-Care High-Sensitivity Cardiac Troponin I. *J Am Coll Cardiol* 2020;75(10):1111-24. doi: 10.1016/j.jacc.2019.12.065

For peer review only - http://bmjopen.bmj.com/site/about/guidelines.xhtml

81. Kavsak PA, Mondoux SE, Ma J, et al. Comparison of two biomarker only algorithms for early risk stratification in patients with suspected acute coronary syndrome. *Int J Cardiol* 2020;319:140-43. doi: 10.1016/j.ijcard.2020.06.066
82. Kavsak PA, Mondoux SE, Sherbino J, et al. Clinical evaluation of Ortho Clinical Diagnostics high-sensitivity cardiac Troponin I assay in patients with symptoms suggestive of acute coronary syndrome. *Clin Biochem* 2020;80:48-51. doi: 10.1016/j.clinbiochem.2020.04.003
83. Kim JW, Kim H, Yun YM, et al. Absolute Change in High-Sensitivity Cardiac Troponin I at Three Hours After Presentation is Useful for Diagnosing Acute Myocardial Infarction in the Emergency Department. *Ann Lab Med* 2020;40(6):474-80. doi: 10.3343/alm.2020.40.6.474
84. Nowak RM, Christenson RH, Jacobsen G, et al. Performance of Novel High-Sensitivity Cardiac Troponin I Assays for 0/1-Hour and 0/2- to 3-Hour Evaluations for Acute Myocardial Infarction: Results From the HIGH-US Study. *Ann Emerg Med* 2020;76(1):1-13. doi: 10.1016/j.annemergmed.2019.12.008
85. Peacock WF, Christenson R, Diercks DB, et al. Myocardial Infarction Can Be Safely Excluded by High-sensitivity Troponin I Testing 3 Hours After Emergency Department Presentation. *Acad Emerg Med* 2020 doi: 10.1111/acem.13922
86. Sandoval Y, Smith SW, Schulz K, et al. Comparison of 0/3-Hour Rapid Rule-Out Strategies Using High-Sensitivity Cardiac Troponin I in a US Emergency Department. *Circ Cardiovasc Qual Outcomes* 2020;13(7):e006565. doi: 10.1161/CIRCOUTCOMES.120.006565
87. Shiozaki M, Inoue K, Suwa S, et al. Implementing the European Society of Cardiology 0-h/1-h algorithm in patients presenting very early after chest pain. *Int J Cardiol* 2020;320:1-6. doi: 10.1016/j.ijcard.2020.07.037
88. Allen BR, Christenson RH, Cohen SA, et al. Diagnostic Performance of High-Sensitivity Cardiac Troponin T Strategies and Clinical Variables in a Multisite US Cohort. *Circulation* 2021;143(17):1659-72. doi: 10.1161/CIRCULATIONAHA.120.049298
89. Andersen CF, Bang C, Lauridsen KG, et al. External validation of a high-sensitive troponin I algorithm for rapid evaluation of acute myocardial infarction in a Danish cohort. *Eur Heart J Acute Cardiovasc Care* 2021;10(9):1056-64. doi: 10.1093/ehjacc/zuab062
90. Dongxu C, Yannan Z, Yilin Y, et al. Evaluation of the 0 h/1 h high-sensitivity cardiac troponin T algorithm in diagnosis of non-ST-segment elevation

For peer review only - http://bmjopen.bmj.com/site/about/guidelines.xhtml

myocardial infarction (NSTEMI) in Han population. *Clin Chem Lab Med* 2021;59(4):757-64. doi: 10.1515/cclm-2020-0367

91. Dupuy AM, Badiou S, Montagnon V, et al. Analytical assessment and performance of the 0/3h algorithm with novel high sensitivity cardiac troponin I. *Clin Chim Acta* 2021;519:111-17. doi: 10.1016/j.cca.2021.04.003

92. Kavsak PA, Hewitt MK, Mondoux SE, et al. Diagnostic Performance of Serial High-Sensitivity Cardiac Troponin Measurements in the Emergency Setting. *J Cardiovasc Dev Dis* 2021;8(8) doi: 10.3390/jcdd8080097

93. Koechlin L, Boeddinghaus J, Nestelberger T, et al. Performance of the ESC 0/2h-algorithm using high-sensitivity cardiac troponin I in the early diagnosis of myocardial infarction. *Am Heart J* 2021;242:132-37. doi: 10.1016/j.ahj.2021.08.008

94. Lopez-Ayala P, Nestelberger T, Boeddinghaus J, et al. Novel Criteria for the Observe-Zone of the ESC 0/1h-hs-cTnT Algorithm. *Circulation* 2021;144(10):773-87. doi: 10.1161/CIRCULATIONAHA.120.052982

95. McCord J, Hana A, Cook B, et al. The role of cardiac testing with the 0/1-hour high-sensitivity cardiac troponin algorithm evaluating for acute myocardial infarction. *Am Heart J* 2021;233:68-77. doi: 10.1016/j.ahj.2020.12.015

96. Olsson P, Khoshnood A, Mokhtari A, et al. Glucose and high-sensitivity troponin T predict a low risk of major adverse cardiac events in emergency department chest pain patients. *Scand Cardiovasc J* 2021;55(6):354-61. doi: 10.1080/14017431.2021.1987512

97. Ruangsomboon O, Thirawattanasoot N, Chakorn T, et al. The utility of the 1-hour high-sensitivity cardiac troponin T algorithm compared with and combined with five early rule-out scores in high-acuity chest pain emergency patients. *Int J Cardiol* 2021;322:23-28. doi: 10.1016/j.ijcard.2020.08.099

98. Sörensen NA, Neumann JT, Ojeda F, et al. Differences in measurement of high-sensitivity troponin in an on-demand and batch-wise setting. *European Heart Journal Acute Cardiovascular Care* 2021;10(3):302-09. doi: 10.1177/2048872620924198

99. Sorensen NA, Gossling A, Neumann JT, et al. Diagnostic Validation of a High-Sensitivity Cardiac Troponin I Assay. *Clin Chem* 2021;67(9):1230-39. doi: 10.1093/clinchem/hvab070

100. Steiro OT, Tjora HL, Langorgen J, et al. Clinical risk scores identify more patients at risk for cardiovascular events within 30 days as compared to standard ACS

For peer review only - http://bmjopen.bmj.com/site/about/guidelines.xhtml

risk criteria: the WESTCOR study. *Eur Heart J Acute Cardiovasc Care* 2021;10(3):287-301. doi: 10.1093/ehjacc/zuaa016

101. Wildi K, Lopez-Ayala P, Koechlin L, et al. Validation of the Novel European Society of Cardiology 0/2-hour Algorithm Using Hs-cTnT in the Early Diagnosis of Myocardial Infarction. *Am J Cardiol* 2021;154:128-30. doi: 10.1016/j.amjcard.2021.06.003

102. Nestelberger T, Boeddinghaus J, Gimenez MR, et al. Direct comparison of high-sensitivity cardiac troponin T and I in the early differentiation of type 1 vs. type 2 myocardial infarction. *Eur Heart J Acute Cardiovasc Care* 2022;11(1):62-74. doi: 10.1093/ehjacc/zuab039

103. Cannon CP, Battler A, Brindis RG, et al. American College of Cardiology key data elements and definitions for measuring the clinical management and outcomes of patients with acute coronary syndromes: A report of the American College of Cardiology Task Force on Clinical Data Standards (Acute Coronary Syndromes Writing Committee). *Journal of the American College of Cardiology* 2001;38(7):2114-30. doi: 10.1016/s0735-1097(01)01702-8

104. Anderson JL, Adams CD, Antman EM, et al. ACC/AHA 2007 guidelines for the management of patients with unstable angina/non-ST-Elevation myocardial infarction: a report of the American College of Cardiology/American Heart Association Task Force on Practice Guidelines (Writing Committee to Revise the 2002 Guidelines for the Management of Patients With Unstable Angina/Non-ST-Elevation Myocardial Infarction) developed in collaboration with the American College of Emergency Physicians, the Society for Cardiovascular Angiography and Interventions, and the Society of Thoracic Surgeons endorsed by the American Association of Cardiovascular and Pulmonary Rehabilitation and the Society for Academic Emergency Medicine. *J Am Coll Cardiol* 2007;50(7):e1-e157. doi: 10.1016/j.jacc.2007.02.013

105. Apple FS, Wu AH, Jaffe AS. European Society of Cardiology and American College of Cardiology guidelines for redefinition of myocardial infarction: how to use existing assays clinically and for clinical trials. *Am Heart J* 2002;144(6):981-6. doi: 10.1067/mhj.2002.124048

106. Anderson JL, Adams CD, Antman EM, et al. 2012 ACCF/AHA focused update incorporated into the ACCF/AHA 2007 guidelines for the management of patients with unstable angina/non-ST-elevation myocardial infarction: a report of the American College of Cardiology Foundation/American Heart Association

For peer review only - http://bmjopen.bmj.com/site/about/guidelines.xhtml

Task Force on Practice Guidelines. *J Am Coll Cardiol* 2013;61(23):e179-347. doi: 10.1016/j.jacc.2013.01.014

107. Hamm CW, Bassand JP, Agewall S, et al. ESC Guidelines for the management of acute coronary syndromes in patients presenting without persistent ST-segment elevation: The Task Force for the management of acute coronary syndromes (ACS) in patients presenting without persistent ST-segment elevation of the European Society of Cardiology (ESC). *Eur Heart J* 2011;32(23):2999-3054. doi: 10.1093/eurheartj/ehr236

108. Arslan M, Dedic A, Boersma E, et al. Serial high-sensitivity cardiac troponin T measurements to rule out acute myocardial infarction and a single high baseline measurement for swift rule-in: A systematic review and meta-analysis. *Eur Heart J Acute Cardiovasc Care* 2020;9(1):14-22. doi: 10.1177/2048872618819421

109. Chiang CH, Chiang CH, Lee GH, et al. Safety and efficacy of the European Society of Cardiology 0/1-hour algorithm for diagnosis of myocardial infarction: systematic review and meta-analysis. *Heart* 2020;106(13):985-91. doi: 10.1136/heartjnl-2019-316343

110. Apple FS, Collinson PO, Kavsak PA, et al. Getting Cardiac Troponin Right: Appraisal of the 2020 European Society of Cardiology Guidelines for the Management of Acute Coronary Syndromes in Patients Presenting without Persistent ST-Segment Elevation by the International Federation of Clinical Chemistry and Laboratory Medicine Committee on Clinical Applications of Cardiac Bio-Markers. *Clin Chem* 2021;67(5):730-35. doi: 10.1093/clinchem/hvaa337

111. Giannitsis E, Blankenberg S, Christenson RH, et al. Critical appraisal of the 2020 ESC guideline recommendations on diagnosis and risk assessment in patients with suspected non-ST-segment elevation acute coronary syndrome. *Clin Res Cardiol* 2021;110(9):1353-68. doi: 10.1007/s00392-021-01821-2

112. Six A, Backus B, Kelder J. Chest pain in the emergency room: value of the HEART score. *Neth Heart J* 2008;16(6):191-6. doi: 10.1007/BF03086144

113. Than M, Flaws D, Sanders S, et al. Development and validation of the Emergency Department Assessment of Chest pain Score and 2 h accelerated diagnostic protocol. *Emerg Med Australas* 2014;26(1):34-44. doi: 10.1111/1742-6723.12164

114. Fox CS, Muntner P, Chen AY, et al. Use of evidence-based therapies in short-term outcomes of ST-segment elevation myocardial infarction and non-ST-segment

For peer review only

For peer review only - http://bmjopen.bmj.com/site/about/guidelines.xhtml

elevation myocardial infarction in patients with chronic kidney disease: a report from the National Cardiovascular Data Acute Coronary Treatment and Intervention Outcomes Network registry. *Circulation* 2010;121(3):357-65. doi: 10.1161/CIRCULATIONAHA.109.865352

115. Bhatnagar P, Wickramasinghe K, Williams J, et al. The epidemiology of cardiovascular disease in the UK 2014. *Heart* 2015;101(15):1182-9. doi: 10.1136/heartjnl-2015-307516

116. Apple FS, Sandoval Y, Jaffe AS, et al. Cardiac Troponin Assays: Guide to Understanding Analytical Characteristics and Their Impact on Clinical Care. *Clin Chem* 2017;63(1):73-81. doi: 10.1373/clinchem.2016.255109

117. Neumann JT, Twerenbold R, Ojeda F, et al. Application of High-Sensitivity Troponin in Suspected Myocardial Infarction. *N Engl J Med* 2019;380(26):2529-40. doi: 10.1056/NEJMoa1803377

118. Twerenbold R, Wildi K, Jaeger C, et al. Optimal Cutoff Levels of More Sensitive Cardiac Troponin Assays for the Early Diagnosis of Myocardial Infarction in Patients With Renal Dysfunction. *Circulation* 2015;131(23):2041-50. doi: 10.1161/CIRCULATIONAHA.114.014245

119. Dubin R, Li Y, He J, et al. Predictors of high sensitivity cardiac troponin T in chronic kidney disease patients: a cross-sectional study in the chronic renal insufficiency cohort (CRIC). *BMC Nephrology* 2013;14(229) doi: 10.1186/1471-2369-14-229

For peer review only

For peer review only - http://bmjopen.bmj.com/site/about/guidelines.xhtml

Figure legends

Figure 1. PRISMA Flow Diagram

Figure 2. Cumulative number of study participants assessed by specific testing protocols and assays for serial high-sensitivity cardiac troponin measurements*

* The numbers of cumulative participants are based on simple summations of all participants assessed in the relevant studies. These numbers may be overestimated due to overlapping inclusions of patients from same cohorts and/or institutions.

For peer review only

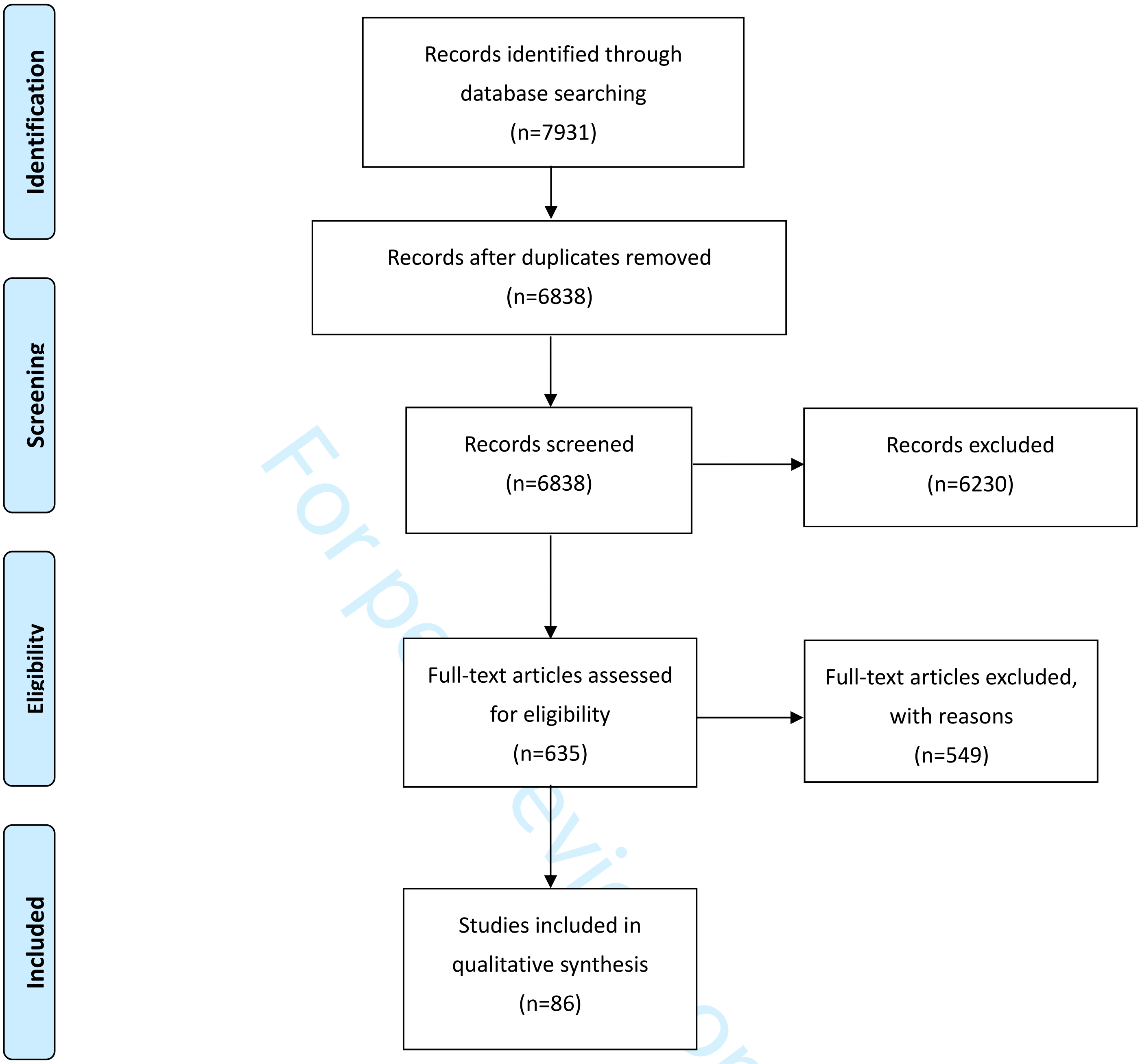
Identification
Screening
Eligibility
Included
Records identified through database searching (n=7931)
Records after duplicates removed (n=6838)
Records screened (n=6838)
Records excluded (n=6230)
Full-text articles assessed for eligibility (n=635)
Full-text articles excluded, with reasons (n=549)
Studies included in qualitative synthesis (n=86)

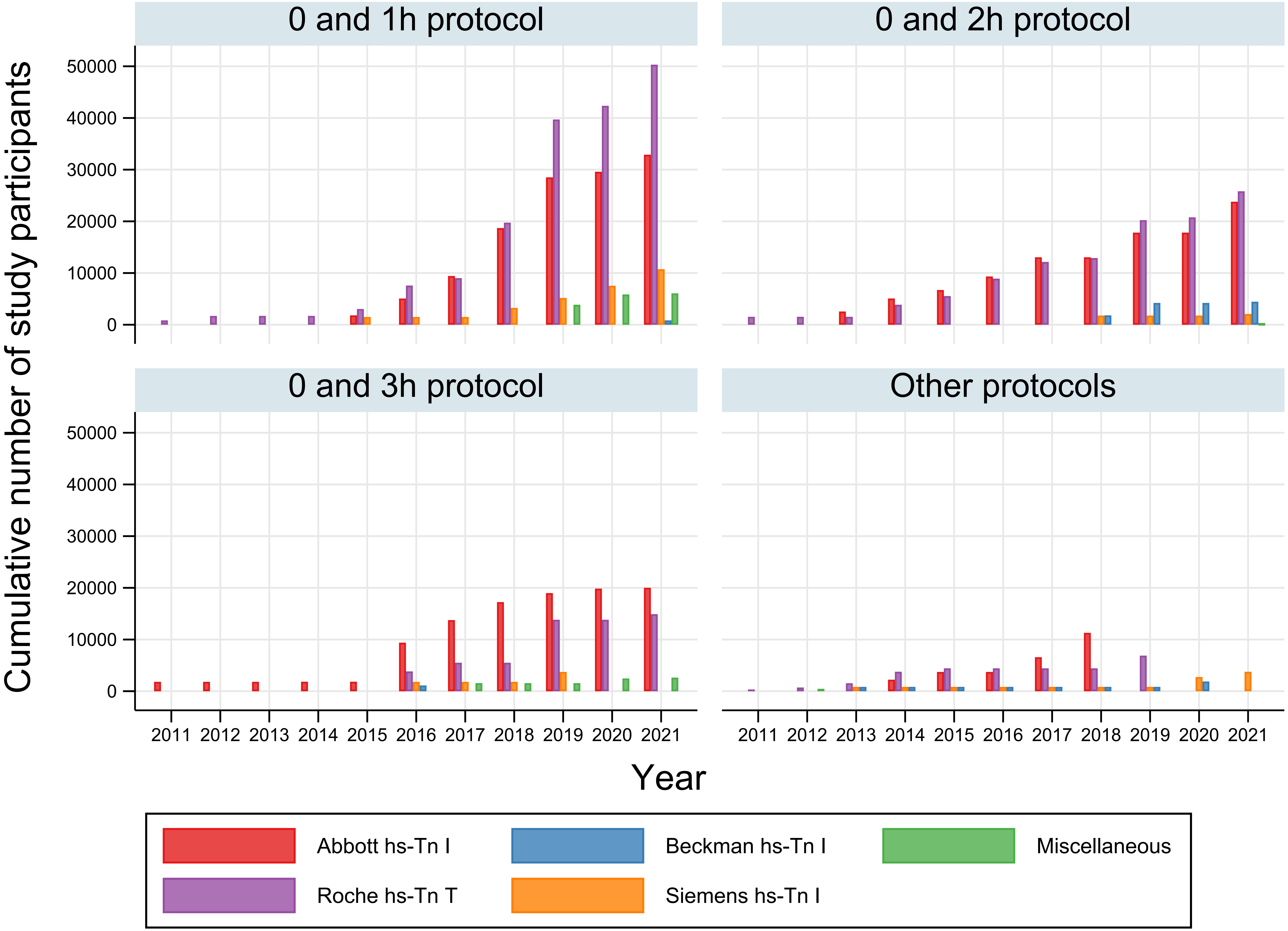
0 and 1h protocol
0 and 2h protocol
0 and 3h protocol
Other protocols
Cumulative number of study participants
0
10000
20000
30000
40000
50000
2011 2012 2013 2014 2015 2016 2017 2018 2019 2020 2021
Year
Abbott hs-Tn I
Beckman hs-Tn I
Miscellaneous
Roche hs-Tn T
Siemens hs-Tn I

Supplementary material

Search Strategy

MEDLINE by Ovid

1 exp Chest Pain/
2 (chest adj2 pain*).ti,ab.
3 chest discomfort.ti,ab.
4 exp Acute Coronary Syndrome/
5 acute coronary syndrome*.ti,ab.
6 ACS.ti,ab.
7 exp Angina, Unstable/
8 unstable angina.ti,ab.
9 Myocardial Infarction/di
10 myocardial infarction.ti,ab.
11 heart attack.ti,ab.
12 or/1-11
13 cardiac troponin*.ti,ab.
14 high* sensitiv* troponin*.ti,ab.
15 hs-ctn*.ti,ab.
16 elecsys.ti,ab.
17 architectstat.ti,ab.
18 architect stat.ti,ab.
19 accutni*.ti,ab.
20 accutnl*.ti,ab.
21 centaur ultra.ti,ab.
22 HISCL.ti,ab.
23 vidas.ti,ab.
24 lumipulse.ti,ab.
25 serial troponin.ti,ab.
26 contemporary troponin.ti,ab.
27 heart adp.ti,ab.
28 (EDACS and troponin).ti,ab.
29 (Adapt adp and troponin).ti,ab.
30 (GRACE and troponin).ti,ab.
31 (TIMI and troponin).ti,ab
32 or/13-31

For peer review only

33 exp Emergency Service, Hospital/
34 (emergency adj (room* or department*)).ti,ab.
35 (ER or ED).ti,ab.
36 (presenting or presented).ti,ab.
37 presentation.ti,ab
38 (admission or admitted).ti,ab.
39 chest pain unit.ti,ab.
40 or/33-39
41 12 and 32 and 40

Embase by Elsevier

1 exp Chest Pain/
2 (chest adj2 pain*).ti,ab.
3 chest discomfort.ti,ab.
4 exp Acute Coronary Syndrome/
5 acute coronary syndrome*.ti,ab.
6 ACS.ti,ab.
7 exp Angina, Unstable/
8 unstable angina.ti,ab.
9 Myocardial Infarction/di
10 myocardial infarction.ti,ab.
11 heart attack.ti,ab.
12 or/1-11
13 cardiac troponin*.ti,ab.
14 high* sensitiv* troponin*.ti,ab.
15 hs-ctn*.ti,ab.
16 elecsys.ti,ab.
17 architectstat.ti,ab.
18 architect stat.ti,ab.
19 accutni*.ti,ab.
20 accutnl*.ti,ab.
21 centaur ultra.ti,ab.
22 HISCL.ti,ab.
23 vidas.ti,ab.
24 lumipulse.ti,ab.
25 serial troponin.ti,ab.

For peer review only

26 or/13-25

27 exp Emergency Service, Hospital/

28 (emergency adj (room* or department*)).ti,ab.

29 (ER or ED).ti,ab.

30 (presenting or presented).ti,ab.

31 presentation.ti,ab.

32 (admission or admitted).ti,ab.

33 chest pain unit.ti,ab.

34 or/27-33

35 12 and 26 and 34

36 limit 35 to yr="2006 -Current"

Cochrane Database of Systematic Reviews and CENTRAL database by the Cochrane Library

#1 MeSH descriptor: [Chest Pain] this term only

#2 (chest next pain):ti,ab

#3 (chest next discomfort):ti,ab

#4 MeSH descriptor: [Acute Coronary Syndrome] explode all trees

#5 ACS:ti,ab

#6 (acute next coronary next syndrome):ti,ab

#7 MeSH descriptor: [Coronary Artery Disease] explode all trees

#8 (coronary next artery next disease):ti,ab

#9 MeSH descriptor: [Angina, Unstable] explode all trees

#10 (unstable next angina):ti,ab

#11 MeSH descriptor: [Myocardial Infarction] explode all trees

#12 (myocardial next infarction):ti,ab

#13 (heart next attack):ti,ab

#14 #1 or #2 or #3 or #4 or #5 or #6 or #7 or #8 or #9 or #10 or #11 or #12 or #13

#15 MeSH descriptor: [Troponin] explode all trees

#16 troponin:ti,ab

#17 hs-ctn*:ti,ab

#18 #15 or #16 or #17

#19 MeSH descriptor: [Emergency Service, Hospital] explode all trees

#20 (emergency next (room* or department*)):ti,ab

#21 (ER or ED):ti,ab

#22 (presenting or presented):ti,ab

For peer review only

#23 presentation:ti,ab

#24 (admission or admitted):ti,ab

#25 "chest pain unit":ti,ab

#26 #19 or #20 or #21 or #22 or #23 or #24 or #25

#27 #14 and #18 and #26

Science Citation Index

#1 TITLE: (high* sensitiv* troponin)

#2 TITLE: ("serial troponin" or "serial cardiac troponin")

#3 TOPIC: ("myocardial infarction*" or "heart attack*" or "heart failure")

#4 TOPIC: (diagnosi* or detect* or identif*)

#5 #2 OR #1

#6 #5 AND #4 AND #3

For peer review only

Appendix Table 1. Included studies reporting the diagnostic accuracy of AMI and 30-day clinical outcomes.

Author/publish year[*]	Number of patients[†]	Age[‡], year	Renal dysfunction[‖]	Study name or region	Enrolment years	Need of ECG[¶]	Clinical outcomes[**]
**Troponin I assays manufactured by Abbott**							
0 and 1h protocol							
Gimenez 2015[1]	1811/2377 (76)	62 (49-62)	Excl	APACE	2006-2012		30-day
Pickering 2016a[2]	2222 (ND)	60 (±14)	Incl	ADAPT, ADAPT-ADP, EDACS-ADP, RING	ADAPT: 2007-2011; ADAPT-ADP: 2010-2012; EDACS: 2013-2014; RING: ND	Excl	Dx MI
Neumann 2016[3]	1040 (ND)	65 (52-75)	Incl	BACC	2013-2014		Dx MI
Boeddinghaus 2017[4]	2828/3642 (78)	62 (49-74)	Excl	APACE	2006-2015		Dx MI, 30-day
Neumann 2017[5]	1516 (ND)	65 (51-75)	Incl	BACC	2013-2016		Dx MI
Twerenbold 2018a[6]	2504<br>445 /4168 (71)	58 (47-70)[‡‡]<br>79 (73-84)[§§]	Excl<br>Incl	APACE<br>APACE	ND<br>ND		Dx MI, 30-day<br>Dx MI, 30-day
Twerenbold 2018b[7]	3500/5631 (62)	62[‖‖]	P-Incl	APACE, BACC	2006-2016		Dx MI, 30-day
Boeddinghaus 2018a[8]	4269/5136 (83)	62 (49-74)	P-Incl	APACE, BACC, TRAPID-AMI	2006-2015		Dx MI, 30-day
Wildi 2019[9]	2945/4169 (71)	62 (49-74)	Excl	APACE	2006-2015	P-need	Dx MI, 30-day
Nestelberger 2019a[10]	2828/3980 (71)	62 (49-74)	Excl	APACE	2006-2015		Dx MI, 30-day
Boeddinghaus 2019[11]	937/1288 (73)	60 (48-74)	Excl	APACE	2011-2015		Dx MI
Neuman 2019[12]	1534 (ND)	65[‖‖]	Incl	BACC	ND		Dx MI
Sörensen 2019[13]	1279 (ND)	65[‖‖]	Incl	BACC	2015-2016		Dx MI
Boeddinghaus 2020[14]	962/1328 (72)	60 (47-73)	Excl	APACE	2011-2014		Dx MI, 30-day
Sörensen 2021a[15]	937 (ND)	65 (52-75)	Incl	BACC	2013-2016		Dx MI
Sörensen 2021b[16]	1800 (ND)	65 (52-75)	Incl	BACC	2013-2014		Dx MI
Nestelberger 2022[17]	862/6021 (14)	61 (49-74)	Excl	APACE	2006-2018		Dx MI
0 and 2h protocol							
Cullen 2013[18]	2544 (ND)	61[‖‖]	P-Incl	APACE, ADAPT	APACE: 2006-2008; ADAPT: 2007-2011	P-need	30-day
Aldous 2014[19]	962 (ND)	66 (56-76)	Incl	ASPECT, ADAPT	2007-2010		Dx MI, 30-day
Cullen 2014[20]	1959/1571 (80)	60[‖‖]	Incl	Australia/New Zealand	2007-2011		Dx MI
Eggers 2015[21]	1624 (ND)	61 (±15)	Incl	ADAPT	2007-2011		Dx MI
Boeddinghaus 2016[22]	2629 (ND)	62[‖‖]	P-Incl	APACE, ADAPT	APACE: 2006-2012; ADAPT: 2007-2011	P-need	Dx MI, 30-day
Wildi 2017[23]	2525/4417 (57)	62[‖‖]	P-Incl	APACE, ADAPT	APACE: 2006-2013; ADAPT: 2007-2011	P-need	Dx MI, 30-day
Lindahl 2017[24]	1197 (ND)	64[‖‖]	P-Incl	APACE, FASTEST	APACE: 2008-2014, FASTEST: 2013-2014		Dx MI, 30-day
Wildi 2019[9]	2357/4169 (57)	62 (50-74)	Excl	APACE	2006-2015	P-need	Dx MI, 30-day
Nestelberger 2019b[25]	2411/2979 (81)	56[‖‖]	P-Incl	APACE, ADAPT, IMPACT	APACE: ND, ADAPT: 2007-2011, IMPACT: 2011-2014		Dx MI, 30-day
Kavsak 2021[26]	935/1366 (68)	69[‖‖]	Incl	ROMI-3	2013		30-day
Koechlin 2021[27]	1863/4587 (41)	61 (50-74)	Excl	APACE	ND		Dx MI
Nestelberger 2022[17]	660/6021 (11)	61 (49-74)	Excl	APACE	2006-2018		Dx MI
0 and 3h protocol							
Keller 2011[††28]	1818 (ND)	61 (±14)	Incl	Germany	2007-2008		Dx MI
Neumann 2016[3]	1040 (ND)	65 (52-75)	Incl	BACC	2013-2014		Dx MI
Wildi 2016[29]	2226/2922 (76)	62 (49-74)	Excl	APACE	2006-2013		Dx MI
Parsonage 2016[30]	3128/3580 (87)	61[‖‖]	P-Incl	APACE, Australia/New Zealand	Australia/New Zealand: 2008-2011; APACE: 2006-2013		30-day
Pickering 2016b[31]	1172 (ND)	60 (±14)	Incl	ADAPT, ADAPT-ADP, EDACS-ADP, RING	ADAPT: 2007-2011; ADAPT-ADP: 2010-2012; EDACS: 2013-2014; RING: ND	Excl	Dx MI
Neumann 2017[5]	1516 (ND)	65 (51-75)	Incl	BACC	2013-2016		Dx MI
Chapman 2017[32]	1218/1292 (94)	62 (±14)	Incl	High-STEACS	2013-2015		Dx MI, 30-day
Sandoval 2017[33]	1068 (ND)	ND	Incl	UTROPIA	2014		
Chapman 2018[34]	1935/1951 (99)	62 (±14)	Incl	High-STEACS	2013-2017	P-need	Dx MI, 30-day
Gunsolus 2018[35]	1555 (ND)	58 (±15)	Incl	UTROPIA	2014		Dx MI
Wildi 2019[9]	1742/4169 (42)	63 (50-76)	Excl	APACE	2006-2015	P-need	Dx MI, 30-day
Kim 2020[36]	281/362 (78)	59[‖‖]	ND	Korea, Seoul	2015-2016		Dx MI
Sandoval 2020[37]	582 (ND)	ND	Incl	UTROPIA	2014		30-day
Dupuy 2021[38]	ND	73 (±17)	Incl	Montpellier, France	ND		Dx MI
Miscellaneous							
Gimenez 2014[39]	2226/2956 (75)	62 (50-75)	Excl	APACE	2006-2012		Dx MI
Pickering 2015[40]	368 (ND)	61 (±13)	Incl	Australia/New Zealand	2010-2012	P-need	Dx MI
Chapman 2017[32]	1218/1292 (94)	62 (±14)	Incl	High-STEACS	2013-2015		Dx MI, 30-day

For peer review only

Author/publish year[*]	Number of patients[†]	Age[‡], year	Renal dysfunction[ǁ]	Study name or region	Enrolment years	Need of ECG[¶]	Clinical outcomes[**]
Sandoval 2017[33]	1631 (ND)	ND	Incl	UTROPIA	2014		Dx MI, 30-day
Miller-Hodges 2018[41]	2193/4789 (46)	64 (±16)	Incl	UK	2013-2014		Dx MI, 30-day
**Troponin T assays manufactured by Roche**							
0 and 1h protocol							
Reichlin 2011[42]	836/1197 (70)	64 (51-76)	Excl	APACE	2007-2009		Dx MI
Reichlin 2012[43]	872/1197 (73)	64 (51-75)	Excl	APACE	2006-2009		Dx MI, 30-day
Reichlin 2015a[44]	1320/1656 (80)	60 (49-73)	Excl	APACE	2007-2009		Dx MI, 30-day
Mokhtari 2016[45]	1038 (ND)	61 (± 18)	Incl	Sweden	2013-2014		Dx MI, 30-day
Mokhtari 2017[46]	1020/1167 (87)	61 (±17)	Incl	Sweden	2013-2014	Excl	30-day
Mueller 2016[††47]	1282 (ND)	62 (50-74)	Excl	TRAPID-AMI	2011-2013		Dx MI, 30-day
Pickering 2016a[2]	2222 (ND)	60 (± 14)	Incl	ADAPT, ADAPT-ADP, EDACS-ADP, RING	ADAPT: 2007-2011; ADAPT-ADP: 2010-2012; EDACS: 2013-2014; RING: ND	Excl	Dx MI
Shiozaki 2017[48]	413 (ND)	72 (59-81)	Incl	Japan	2014-2017		Dx MI, 30-day
Twerenbold 2018a[6]	2767 /4168 (78)	58 (47-70)[‡‡]	Excl	APACE	ND		DX MI, 30-day
	487	79 (73-84)[§§]	Excl	APACE	ND		DX MI, 30-day
Twerenbold 2018b[7]	4368/5631 (78)	62 (50-74)	P-Incl	APACE, BACC	2006-2016		DX MI, 30-day
Boeddinghaus 2018a[8]	5890/6744 (87)	61 (49-74)	P-Incl	APACE, BACC, TRAPID-AMI	2006-2015		Dx MI, 30-day
Mueller-Hennessen2019[49]	922/1282 (72)	62 (49-74)	Excl	TRAPID-AMI	ND		Dx MI, 30-day
Wildi 2019[9]	3020/4169 (72)	60 (49-73)	Excl	APACE	2006-2015	P-need	Dx MI, 30-day
Chew 2019[50]	1646/1646 (100)	59 (49-69)	Excl	RAPID-TnT	2015-2019	Excl	30-day
Nestelberger 2019a[10]	3123/3980 (78)	61 (49-74)	Excl	APACE	2006-2015		Dx MI, 30-day
Twerenbold 2019[51]	2296 (ND)	60 (49-71)	Incl	Switzerland, Argentina	2015-2017		30-day
Amann 2019[52]	1317 (ND)	72[ǁǁ]	Incl	FAST-MI	2015-2016		Dx MI, 30-day
Andruchow 2020[53]	350/608 (58)	60 (52-72)	Excl	Canada, Alberta	2014-2016		Dx MI, 30-day
Cappellini 2019[54]	7176/14170 (51)	73 (59-82)	Incl	Italy, Desio	2011-2016		Dx MI
Shiozaki 2020[55]	1074/1183 (91)	72 (60-80)	Excl	Japan, Taiwan	2014-2019		30-day
Boeddinghaus 2019[11]	937/1288 (73)	60 (48-74)	Excl	APACE	2011-2015		Dx MI
Boeddinghaus 2020[14]	962/1328 (72)	ND	Excl	APACE	2011-2014		Dx MI, 30-day
Andruchow 2020[53]	350/2911 (12)	61[ǁǁ]	P-Inc	APPROACH	2014-2016		Dx MI, 30-day
Allen 2021[56]	1462/1583 (92)	58 (±13)	Inc	STOP CP	2017-2018		30-day
Dongxu 2021[57]	577/600 (96)	66 (59-75)	P-Inc	China	2017-2018		Dx MI, 30-day
Lopez-Ayala 2021[58]	2076/6684 (31)	63	P-Inc	APACE	APACE: 2006-2018		Dx MI, 30-day
Olsson 2021[59]	1031(ND)	61 (± 18)	Incl	Sweden	2013-2014		30-day
Ruangsomboon 2021[60]	350 (ND)	66 (±15)	P-Incl	Thailand	ND	P-need	Dx MI, 30-day
Sörensen 2021a[98]	565 (ND)	65 (52-75)	Incl	BACC	2013-2016		Dx MI
Nestelberger 2022[15]	892/6021 (15)	61 (49-74)	Excl	APACE	2006-2018		Dx MI
0 and 2h protocol							
Aldous 2011a[61]	939 (ND)	65 (56-76)	Incl	New Zealand	2007-2009		Dx MI
Reichlin 2011[42]	590/1197 (49)	64 (51-76)	Excl	APACE	2007-2009		Dx MI
Cullen 2014[20]	1571/1959 (80)	60[ǁǁ]	Incl	Australia/New Zealand	2007-2011		Dx MI
Parsonage 2014[††62]	764 (ND)	55 (±15)	Incl	Australia	2008-2011		Dx MI, 30-day
Reichlin 2015b[63]	1665/2788 (60)	59[ǁǁ]	P-incl	APACE, Australia	APACE: 2006-2011, Australia: 2008-2011		30-day
Parsonage 2016[30]	3374/3580 (94)	61[ǁǁ]	P-Incl	APACE, Australia/New Zealand	Australia/New Zealand: 2008-2011; APACE: 2006-2013		30-day
Wildi 2017[23]	2525/4417 (57)	62[ǁǁ]	P-Incl	APACE, ADAPT	APACE: 2006-2013; ADAPT: 2007-2011	P-need	Dx MI, 30-day
McRae 2017[64]	722/7131 (10)	59 (50-69)	Excl	APPROACH	2013		Dx MI, 30-day
Borna 2018[65]	751/1165 (64)	64 (50-74)	Incl	Sweden	2013-2014	P-need	30-day
Wildi 2019[9]	2473/4169 (59)	62 (50-74)	Excl	APACE	2006-2015	P-need	Dx MI, 30-day
Lin 2019[66]	2444/2474 (99)	55 (47-64)	Excl	Singapore	2010-2013		30-day
Nestelberger 2019b[25]	2411/2979 (81)	56[ǁǁ]	P-Incl	APACE, ADAPT, IMPACT	APACE: ND, ADAPT: 2007-2011, IMPACT: 2011-2014		Dx MI, 30-day
Andruchow 2020[53]	550/608 (90)	60 (52-72)	Excl	Canada, Alberta	2014-2016		Dx MI, 30-day
Koechlin 2021[27]	2227/4587 (49)	61 (50-74)	Excl	APACE	ND		Dx MI
Wildi 2021[67]	2127 (ND)	61 (49-73)	Excl	APACE	ND		Dx MI
Nestelberger 2022[17]	691/6021 (11)	61 (49-74)	Excl	APACE	2006-2018		Dx MI
0 and 3h protocol							
Pickering 2016b[31]	1098 (ND)	60 (±14)	Incl	ADAPT, ADAPT-ADP, EDACS-ADP, RING	ADAPT: 2007-2011; ADAPT-ADP: 2010-2012; EDACS:	Excl	Dx MI

Author/publish year*	Number of patients†	Age‡, year	Renal dysfunction‖	Study name or region	Enrolment years	Need of ECG¶	Clinical outcomes**
					2011-2014; RING: ND		
Wildi 2016[29]	2727/2922 (93)	62 (49-74)	Excl	APACE	2006-2013		Dx MI
Peacock 2018[††68]	1679 (ND)	55 (47-64)	Incl	USA	2011-2015		Dx MI
Wildi 2019[9]	1908/4169 (46)	63 (50-76)	Excl	APACE	2006-2015	P-need	Dx MI, 30-day
Cappellini 2019[54]	7176/14170 (51)	73 (59-82)	Incl	Italy, Desio	2011-2016		Dx MI
Dupuy 2021[38]	160 (ND)	73 (±17)	Incl	Montpellier, France	ND		Dx MI
Steiro 2021[69]	932/984 (95)	63 (52-74)	Incl	WESTCOR	2015-2017	P-need	30-day
Miscellaneous							
Aldous 2011b[††70]	332 (ND)	64 (53-74)	Incl	New Zealand	2006-2007		Dx MI
Aldous 2012[††71]	385 (ND)	65 (56-76)	Incl	New Zealand	2007-2010		Dx MI
Irfan 2013[72]	830 (ND)	64 (51-75)	Excl	APACE	2006-2009		Dx MI
Gimenez 2014[39]	2226/2956 (75)	62 (50-75)	Excl	APACE	2006-2012		Dx MI
Biener 2015[73]	658/721 (91)	ND	Incl	Germany	ND		Dx MI
Nowak 2018[74]	569/1699 (33)	55 (49-63)	Incl	REACTION-US	2013-2015		Dx MI, 30-day
Lin 2019[66]	2444/2474 (99)	55 (47-64)	Excl	Singapore	2010-2013		Dx MI
**Troponin I assays manufactured by Siemens**							
0 and 1h protocol							
Jaeger 2016[75]	1500/1883 (80)	62 (49-74)	Excl	APACE	ND		Dx MI, 30-day
Boeddinghaus 2018b[76]	1347/1876 (72)	62 (49-75)	Excl	APACE	2006-2013		30-day
Chapman 2019[77]	1951/406 (21)	62 (±14)	Incl	High-STEACS	2013-2017	P-need	30-day
Nowak 2020[78]	2063/2470 (84)	56 (48-65)	Incl	HIGH-US	2015-2016		Dx MI, 30-day
Andersen 2021[79]	1003/1479 (68)	64 (52-74)	P-Incl	RACING-MI	2016-2019		Dx MI
Sörensen 2021b[16]	1800 (ND)	65 (52-75)	Incl	BACC	2013-2014		Dx MI
Nestelberger 2022[17]	418/6021 (7)	61 (49-74)	Excl	APACE	2006-2018		Dx MI
0 and 2h protocol							
Boeddinghaus 2018b[76]	1033/1876 (55)	62 (49-75)	Excl	APACE	2006-2013		30-day
Nestelberger 2022[17]	313/6021 (5)	61 (49-74)	Excl	APACE	2006-2018		Dx MI
0 and 3h protocol							
Wildi 2016[29]	1809/2922 (62)	62 (49-74)	Excl	APACE	2006-2013		Dx MI
Chapman 2019[77]	1920/1951 (98)	62 (±14)	Incl	High-STEACS	2013-2017	P-need	30-day
Sörensen 2021b[16]	1800 (ND)	65 (52-75)	Incl	BACC	2013-2014		Dx MI
Miscellaneous							
Irfan 2013[72]	830 (ND)	64 (51-75)	Excl	APACE	2006-2009		Dx MI
Nowak 2020[78]	1863/2470 (75)	56 (48-65)	Incl	HIGH-US	2015-2016		Dx MI, 30-day
Andersen 2021[79]	1003/1479 (68)	64 (52-74)	P-Incl	RACING-MI	2016-2019		Dx MI
**Troponin I assays manufactured by Beckman**							
0 and 1h protocol							
McCord 2021[80]	552/569 (97)	56 (±11)	Incl	REACTION-US	2013-2015		Dx MI
Nestelberger 2022[17]	278/6021 (5)	61 (49-74)	Excl	APACE	2006-2018		Dx MI
0 and 2h protocol							
Greenslade 2018[81]	1811/2314 (78)	53 (±14)	Incl	ADAPT, IMPACT	ADAPT: 2008-2011; IMPACT: 2011-2014	P-need	30-day
Nestelbergr 2019b[25]	2411/2979 (81)	56[‖‖]	P-Incl	APACE, ADAPT, IMPACT	APACE: ND, ADAPT: 2007-2011, IMPACT: 2011-2014		Dx MI, 30-day
Nestelberger 2022[17]	217/6021 (4)	61 (49-74)	Excl	APACE	2006-2018		Dx MI
0 and 3h protocol							
Wildi 2016[44]	1110/2922 (38)	62 (49-74)	Excl	APACE	2006-2013		Dx MI
Peacock 2020[29]	1049/1929 (54)	56[‖‖]	P-Incl	USA	2012-2013		Dx MI
Miscellaneous							
Irfan 2013[72]	830 (ND)	64 (51-75)	Excl	APACE	2006-2009		Dx MI
Peacock 2020[82]	1049/1929 (54)	56[‖‖]	Excl	USA	2012-2013		Dx MI
**Other assays**							
0 and 1h protocol							
Boeddinghaus 2019[11] (VITROS)	1039/1288 (81)	60 (48-74)	Excl	APACE	2011-2015		Dx MI, 30-day
Neuman 2019[12] (Singulex Clarity)	1534 (ND)	65[‖‖]	Incl	BACC	ND		Dx MI
Sörensen 2019[13] (PATHFAST)	1279 (ND)	65[‖‖]	Incl	BACC	2015-2016		Dx MI
Boeddinghaus 2020[14] (Triage True)	1084/1328 (82)	60 (47-73)	Excl	APACE	2011-2014		Dx MI, 30-day
Kavsak 2020a[83] (VITROS)	906/1366 (66)	68[‖‖]	Incl	ROMI-3	2013-2017		Dx MI, 30-day
Nestelberger 2022[17] (Triage True)	204/6021 (3)	61 (49-74)	Excl	APACE	2006-2018		Dx MI
Nestelberger 2022[17] (VITROS)	160/6021 (3)	61 (49-74)	Excl	APACE	2006-2018		Dx MI
0 and 2h protocol							

For peer review only

Author/publish year*	Number of patients†	Age‡, year	Renal dysfunction‖	Study name or region	Enrolment years	Need of ECG¶	Clinical outcomes**
Nestelberger 2022[17] (Triage True)	204/6021 (3)	61 (49-74)	Excl	APACE	2006-2018		Dx MI
Nestelberger 2022[17] (VITROS)	160/6021 (3)	61 (49-74)	Excl	APACE	2006-2018		Dx MI
0 and 3h protocol							
Schofer 2017[84] (Singulex)	1560/1699 (92)	62 (49-74)	Incl	Germany	2007-2008		Dx MI
Kavsak 2020b[85] (VITROS)	906/1355 (66)	68‖‖	Incl	ROMI-3	2013-2017		Dx MI, 30-day
Dupuy 2021[38] (VITROS)	160	73 (±17)	Incl	Montpellier, France	ND		Dx MI
Miscellaneous							
Schreiber 2012[86] (Singulex)	465/486 (96)	67 (21-98)¶¶	Incl	USA	2005-2006		Dx MI, 30-day

ADAPT = 2-hour Accelerated Diagnostic Protocol to Assess Patients with Chest Pain Symptoms using Contemporary Troponins as the Only Biomarker; ADAPT-ADP = 2-hour Accelerated Diagnostic Protocol to Assess Patients with Chest Pain Symptoms using Contemporary Troponins as the Only Biomarker - Accelerated Diagnostic Pathway; APACE = Advantageous Predictors of Acute Coronary Syndromes Evaluation study; APPOACH = Alberta Provincial PRoject for Outcome Assessment in Coronary Heart Disease registry; ASPECT = Asia Pacific Evaluation of Chest pain Trial; BACC = Biomarkers in Acute Cardiac Care study; CKD = chronic kidney diseases; 30-day = 30-day clinical outcomes; Dx = diagnosis; EDACS-ADP = Emergency Department Assessment of Chest Pain Score - Accelerated Diagnostic Pathway; Excl = excluded; FASTEST = Fast Assessment of Thoracic pain in the Emergency department using high-Sensitive Troponins; High-STEACS = High-Sensitivity Troponin in the Evaluation of Patients With Acute Coronary Syndrome; IMPACT = Improved Assessment of Chest Pain Trial; Incl = included; IQR = interquartile range; MI = myocardial infarction; ND = no data; P-Incl = partially included; P-need = partially needed; RING = Reducing the Time Interval for Identifying New Guideline Defined Myocardial Infarction in Patients with Suspected Acute Coronary Syndrome; SD = standard deviation; TRAPID-AMI = High-sensitivity cardiac Troponin T assay for RAPID rule-out of AMI; USA = United States of America; UTROPIA = Use of Abbott High Sensitivity Troponin I Assay In Acute Coronary Syndromes, RAPID-TnT = Rapid Assessment of Possible ACS in the Emergency Department with High-Sensitivity Troponin T, ROMI-3 = Rule-out MI 3h.

*Underlined studies are representative reports with the largest sample size of unique study participants among reports based on (partially) overlapping cohorts.

†The number of analysed/enrolled patients; the number of enrolled patients is presented only if the relevant data were reported.

‡Median (interquartile range) or mean ± SD.

‖Renal dysfunction refers to terminal kidney disease that requires regular haemodialysis. "Partially included" indicates a mixed study that consisted of several cohorts including patients with or without renal dysfunction.

¶Need of ECG in the assessment of participant eligibility. "Partially needed" (shown as "P-need") indicates that at least one of the participating study sites or included cohorts needed ECG findings for assessing the participant eligibility. "Excluded" (shown as "Excl") indicates patients with ischemia on ECG were excluded.

**Studies that assessed the diagnostic accuracy of myocardial infarction and 30-day clinical outcomes are indicated as Dx MI and 30-day, respectively.

††A study that included patients suspected of having ST-elevation MI in addition to patients suspected of having non-ST elevation MI.

‡‡Normal renal function.

§§Renal dysfunction.

‖‖Weighted average.

For peer review only

¶¶Range.

For peer review only

Appendix Table 2. Excluded articles and reasons for exclusion.

Authors	Reasons
Agewall 2007[87]	Only patients admitted in the coronary care units
Ahmadi 2017[88]	Only patients with positive hs-cTn
Alcalai 2021[89]	Single, baseline hs-cTn only
Aldous 2011[90]	Hs-cTn assessed at baseline only
Aldous 2012a[91]	No accuracy data
Aldous 2012c[92]	No accuracy data
Alghamdi 2020[93]	Use of a non-hs-cTn
Al–Saleh 2014[94]	Review article
Alushi 2021[95]	Irrelevant clinical context
Anand 2021[96]	Irrelevant clinical context
Apple 2006[97]	Use of a non-hs-cTn
Apple 2009[98]	Use of a non-hs-cTn
Astley 2014[99]	Study protocol
Avest 2016[100]	Single, baseline hs-cTn only
Bahrmann 2012[101]	Irrelevant clinical context
Bahrmann 2013[102]	Irrelevant clinical context
Bahrmann 2016[103]	Irrelevant
Bali 2021[104]	Use of a non-hs-cTn
Ballocca 2017[105]	Patients with positive hs-cTn only
Bandstein 2014[106]	Single, baseline hs-cTn only
Bandstein 2016[107]	Irrelevant clinical context
Bandstein 2017[108]	Single, baseline hs-cTn only
Bellini 2021[109]	Irrelevant clinical context
Bhardwaj 2011[110]	Single, baseline hs-cTn only
Biener 2013[111]	Irrelevant clinical context
Birkhahn 2011[112]	No accuracy data
Bjurman 2013[113]	Only patients admitted in the coronary care units
Body 2011[114]	Single, baseline hs-cTn only
Body 2015a[115]	Single, baseline hs-cTn only
Body 2015b[116]	Single, baseline hs-cTn only
Body 2016[117]	Single, baseline hs-cTn only
Body 2020[118]	Single, baseline hs-cTn only
Boeckel 2015[119]	Use of a non-hs-cTn.
Boeddinghaus 2017[120]	Patients with positive hs-cTn only
Bonaca 2013[121]	Single, baseline hs-cTn only
Borna 2014[122]	Only patients admitted in the coronary care units

For peer review only

Borna 2016[123]	Only patients admitted in the hospitals
Bove 2017[124]	Only patients admitted in the hospitals
Braga 2011[125]	No accuracy data
Brichko 2018[126]	Patients with negative hs-cTn only
Bularga 2019 [127]	Single, baseline hs-cTn only
Caglar 2020[128]	Irrelevant clinical context
Carlton 2016[129]	Single, baseline hs-cTn only
Carlton 2016[130]	Irrelevant
Carlton 2018[131]	Irrelevant
Carlton 2020 [132]	Single, baseline hs-cTn only
Cavender 2017[133]	Irrelevant
Celik 2011[134]	Case series of patients with ACS
Chapman 2017[135]	Review article
Charpentier 2016 [136]	Guidelines
Chenevier–Gobeaux 2016[137]	Irrelevant clinical context
Chew 2016[138]	Irrelevant
Chew 2018[139]	Single, baseline hs-cTn only
Christ 2010[140]	Single, baseline hs-cTn only
Christ 2018[141]	Use of a non-hs-cTn
Christenson 2020[142]	No accuracy data
Chuang 2016[143]	Irrelevant clinical context
Clerico 2019[144]	Irrelevant clinical context
Colak 2017[145]	Article not obtainable
Collinson 2006a[146]	Single, baseline hs-cTn only
Collinson 2006b[147]	Single, baseline hs-cTn only
Collinson 2015[148]	No accuracy data
Conde 2013[149]	No accuracy data
Cook 2021[150]	Single, baseline hs-cTn only
Corsini 2015[151]	No accuracy data
Cramer 2007[152]	Use of a non-hs-cTn
Cullen 2013[153]	Use of a non-hs-cTn
Cullen 2016[154]	Irrelevant
Dadkhah 2017[155]	Irrelevant
Daly 2012[156]	Patients submitted to the catheter laboratory only
Davarani 2012[157]	Use of a non-hs-cTn
Dawson 2013[158]	Irrelevant clinical context
Diercks 2012[159]	Use of a non-hs-cTn
Druey 2015[160]	Use of a non-hs-cTn

For peer review only

Eggers 2011[161]	Use of a non-hs-cTn
Etaher 2021[162]	Irrelevant clinical context
Fan 2014[163]	No accuracy data
Fanaroff 2015[164]	Irrelevant
Freund 2011[165]	Single, baseline hs-cTn only
Frisoli 2017[166]	Irrelevant
Gassenmaier 2012[167]	Irrelevant clinical context
Giannitsis 2010[168]	Only patients admitted in the chest pain unit
Giannitsis 2010a[169]	Only patients with confirmed NSTE-ACS
Gimenez 2016[170]	Single, baseline hs-cTn only and no accuracy data
Gimenez 2020[171]	Irrelevant clinical context
Gimenez 2021[171]	Irrelevant clinical context
Giner–Caro 2013[172]	Letter
Goodman 2015[173]	Irrelevant clinical context
Gratzel 2015[174]	No accuracy data
Graven 2021[175]	Irrelevant clinical context
Greenslade 2018[176]	Single, baseline hs-cTn only
Grinstein 2015[177]	Only patients with NSTEMI
Haaf 2014[178]	Single, baseline hs-cTn only
Haider 2017[179]	Single, baseline hs-cTn only
Halim 2009[180]	Single, baseline hs-cTn only
Hall 2012[181]	No accuracy data
Hochholzer 2011[182]	Single, baseline hs-cTn only
Hochholzer 2014[183]	Patients submitted to the catheter laboratory only
Hochholzer 2016[184]	Guidelines
Hoeller 2013[185]	Single, baseline hs-cTn only
Hromadka 2012[186]	Irrelevant clinical context
Huang 2015[187]	Single, baseline hs-cTn only
Huang 2016[188]	Single, baseline hs-cTn only
Ilva 2009[189]	Single, baseline hs-cTn only
Invernizzi 2013[190]	Review article
Isiksacan 2017[191]	Single, baseline hs-cTn only
Iwasaki 2017[192]	Use of a non-hs-cTn
Jairam 2011[193]	Irrelevant clinical context
Johannessen 2020[194]	Irrelevant clinical context
Karady 2021[195]	Use of a non-hs-cTn
Karon 2018[196]	Irrelevant clinical context

For peer review only

Kavsak 2006[197]	Use of a non-hs-cTn
Kavsak 2009[198]	Single, baseline hs-cTn only
Kavsak 2012[199]	Single, baseline hs-cTn only
Kavsak 2017[200]	Single, baseline hs-cTn only
Kavsak 2018a[201]	Single, baseline hs-cTn only
Kavsak 2018b[202]	Single, baseline hs-cTn only
Kavsak 2020[203]	Single, baseline hs-cTn only
Kelly 2011[204]	Single, baseline hs-cTn only
Kelly 2014a[205]	Patients with negative hs-cTn only
Kelly 2014b[206]	Irrelevant
Kelly 2015[207]	Irrelevant
Kemper 2017[208]	Use of a non-hs-cTn
Khan 2021[209]	Irrelevant clinical cotext
Khoshnood 2020[210]	Single, baseline hs-cTn only
Kienbacher 2021[211]	No accuracy data
Kim 2013[212]	Irrelevant
Kimenai 2020[213]	Only patients admitted in the coronary care units
Kitamura 2013[214]	Single, baseline hs-cTn only
Kremneva 2016[215]	Use of a non-hs-cTn.
Krintus 2014[216]	Irrelevant
Kumar 2016[217]	Two-gate design (patients with MI and healthy subjects)
Lambrakis 2021[218]	Irrelevant clinical context
Lee 2010[219]	No accuracy data
Lehmacher 2020[220]	Irrelevant clinical context
Li 2015[221]	Meta-analysis.
Lim 2008[222]	Irrelevant
Lim 2020[194]	Single, baseline hs-cTn only
Lin 2012[223]	Review article.
Lindahl 2010[224]	Patients with ACS only
Love 2016[225]	Irrelevant clinical context
Lynn 2011[226]	Irrelevant clinical context
Maag 2015[227]	Irrelevant clinical context
MacRae 2006[228]	No accuracy data
Madsen 2015[229]	Only patients with positive hs-cTn
Mahler 2013[230]	Irrelevant
Marjot 2017[231]	Single, baseline hs-cTn only
Markota 2011[232]	Use of a non-hs-cTn.
Masotti 2018[233]	Irrelevant

For peer review only

May 2014[234]	Single, baseline hs-cTn only
McRae 2015[235]	Commentary
McRae 2017[64]	Single, baseline hs-cTn only
Melki 2011[236]	Only patients admitted in the coronary care units
Melki 2012[237]	Irrelevant
Melki 2015[238]	Irrelevant
Meune 2011[239]	Single, baseline hs-cTn only
Meune 2012[240]	Irrelevant
Miller 2008[241]	Irrelevant clinical context
Mills 2011[242]	Irrelevant
Mills 2012[243]	Single, baseline hs-cTn only
Moal 2007[244]	Irrelevant
Mohsen 2016[245]	No accuracy data
Mokhtari 2016[246]	Single, baseline hs-cTn only
Moliner 2020[247]	No accuracy data
Mueller 2012a[248]	Single, baseline hs-cTn only
Mueller 2012b[249]	Irrelevant clinical context
Mueller 2013[250]	Review article.
Mueller 2014[251]	Irrelevant clinical context
Mueller–Hennessen 2017[252]	Only patients with positive hs-cTn
Mungai 2020[253]	Irrelevant clinical context
Nacke 2014[254]	Only patients with ACS
Nadarajah 2021[255]	Clinical guidance
Narain 2008[256]	Only patients with ACS
Nejatian 2017[257]	Irrelevant clinical context
Nestelberger 2016[258]	No accuracy data
Nestelberger 2021a[259]	Irrelevant clinical context
Nestelberger 2021b[260]	Irrelevant clinical context
Neumann 2017[261]	Single, baseline hs-cTn only
Nilsson 2021[262]	Irrelevant clinical context
Normann 2012[263]	Irrelevant clinical context
Nowak 2021[264]	No accuracy data
Olivieri 2012[265]	No accuracy data
Omland 2011[266]	Review article
Osredkar 2021[267]	Use of non-hs-cTn
Palamalai 2013[268]	Use of non-hs-cTn
Paoloni 2010[269]	No accuracy data
Papendick 2017[270]	Study protocol

For peer review only

Parikh 2015[271]	Meta-analysis
Patsias 2017[272]	Only patients with positive hs-cTn
Peck 2016[273]	Irrelevant clinical context
Pickering 2018[274]	Irrelevant clinical context
Pickering 2020[275]	Irrelevant clinical context
Puelacher 2019[276]	Irrelevant clinical context
Rao 2012[277]	Irrelevant
Reichlin 2009[278]	Single, baseline hs-cTn only
Reichlin 2013[279]	Case series
Reiter 2011[280]	Review article
Ren 2021[281]	Single, baseline hs-cTn only
Renaud 2008[282]	Use of non-hs-cTn.
Roberts 2014[283]	Not a primary study
Roos 2017a[284]	Irrelevant clinical context
Roos 2017b[285]	Irrelevant clinical context
Rottger 2017[286]	Irrelevant clinical context
Rubini 2013[287]	No accuracy data
Rudolph 2012[288]	Irrelevant
Saad 2015a[289]	Single, baseline hs-cTn only
Saad 2015b[289]	Single, baseline hs-cTn only
Saenger 2010[290]	Conference abstract.
Saenger 2011[291]	Irrelevant
Sanchis 2012[292]	Only patients with positive hs-cTn
Sanchis 2014[293]	Irrelevant
Sanchis 2016[294]	Irrelevant
Sandoval 2015[295]	No accuracy data
Sandoval 2017[296]	Single, baseline hs-cTn only
Sandoval 2017[297]	Single, baseline hs-cTn only
Sandoval 2020[37]	Irrelevant clinical context
Santalo 2013[298]	Multi-gate design of case-mix
Schonemann–Lund 2015[299]	Irrelevant clinical context
Shah 2015[300]	Single, baseline hs-cTn only
Shah 2015 [301]	Single, baseline hs-cTn only
Shin 2020[302]	Irrelevant clinical context
Shortt 2015[303]	Single, baseline hs-cTn only
Shortt 2017[304]	Single, baseline hs-cTn only
Simpson 2014[305]	Irrelevant clinical context
Singer 2017[306]	Use of non-hs-cTn

For peer review only

Slagman 2017[307]	Use of non-hs-cTn
Sorensen 2017[308]	Irrelevant
Sorensen 2019[309]	Single, baseline hs-cTn only
Stengaard 2013[310]	Use of non-hs-cTn
Stopyra 2016[311]	Irrelevant
Stopyra 2020[312]	Irrelevant clinical context
Stoyanov 2020[313]	Irrelevant clinical context
Storrow 2015a[314]	Use of non-hs-cTn
Storrow 2015b[315]	Use of non-hs-cTn
Straface 2008[316]	Use of non-hs-cTn
Su 2015[317]	Irrelevant clinical context
Suh 2018[318]	Use of non-hs-cTn
Suzuki 2018[319]	Use of non-hs-cTn
Takakuwa 2009[320]	Use of non-hs-cTn
Tecson 2017[321]	Use of non-hs-cTn
Than 2011[322]	Use of non-hs-cTn
Than 2012[323]	Use of non-hs-cTn
Than 2014[324]	Irrelevant
Thelin 2015[325]	No accuracy data
Truong 2012[326]	Irrelevant
Twerenbold 2015[327]	Single, baseline hs-cTn only
Vafaie 2014[328]	Educational article
van der Laarse 2013[329]	Use of non-hs-cTn.
Van Hise 2018[330]	Irrelevant
Vasile 2017[331]	Review article
Vasudevan 2017[332]	Use of non-hs-cTn.
Venge 2009a[333]	Two–gate design study
Venge 2009b[334]	Irrelevant clinical context
Wang 2015a[335]	Only patients with COPD
Wang 2015b[336]	Two-gate design study (patients with AMI and healthy subjects)
Wassef 2015a[337]	Only patients admitted in the coronary care units
Wassef 2015b[337]	Only patients admitted in the Cardiology department
Wassie 2020[338]	Use of non-hs-cTn
Weber 2011[339]	Irrelevant clinical context
Wereski 2021[340]	Irrelevant clinical context
Westwood 2015[341]	Review article
Wildi 2013[342]	Patients with positive hs-cTn were excluded.
Willeit 2017[343]	Meta-analysis

For peer review only

Wolf 2014[344]	Patients with ACS only
Wong 2010[345]	Irrelevant clinical context
Yang 2021[346]	Irrelevant clinical context
Yean 2020[347]	Irrelevant clinical context
Yokoyama 2018[348]	Irrelevant clinical context
Zhao 2021[349]	Irrelevant clinical context

AMI = acute myocardial infarction; ACS = acute coronary syndrome; COPD = chronic obstructive pulmonary disease; hs–cTn = high sensitivity cardiac troponin, NSTE-ACS = non-ST-elevation acute coronary syndrome

For peer review only

Appendix Table 3. Institution and geographic characteristics of included study cohorts.

Cohort name		Europe		Australasia		North America		Asia	
(N of participants)	Institutions	City	Country	City	Country	City	Country	City	Country
ADAPT[323] (1635)***	Christchurch Hospital			Christchurch	New Zealand				
	Royal Brisbane and Women's Hospital			Brisbane	Australia				
ADAPT-ADP[324] (406)***	Christchurch Hospital			Christchurch	New Zealand				
APACE[350] (4368) ***	Hospital Clinic Barcelona	Barcelona	Spain						
	Hospital Clinic San Carlos	Madrid	Spain						
	Hospital del Mar	Barcelona	Spain						
	Kantonsspital Baselland, Standort Bruderholz	Bottmingen	Switzerland						
	Kantonsspital Baselland, Standort Liestal	Liestal	Switzerland						
	Kantonsspital Olten	Olten	Switzerland						
	Klinik St. Anna	Luzern	Switzerland						
	Masaryk University	Brno	Czech						
	Medical University of Silesia	Silesia	Poland						
	San Martino Hospital	San Martino	Italy						
	Spital Limmattal	Schlieren	Switzerland						
	University Hospital of Basal	Basel	Switzerland						
	University Hospital of Zurich	Zurich	Switzerland						
APPROACH[351] (722)	University of Calgary					Calgary	Canada		
	University of Alberta					Edomonton	Canada		

Cohort name		Europe		Australasia		North America		Asia	
(N of participants)	Institutions	City	Country	City	Country	City	Country	City	Country
ASPECT[322] (ND)	Alberta Health Services					Calgary	Canada		
	Apollo Hospital							Delhi	India
	Chang Gung Memorial Hospital							Taipei	Taiwan
	China Medl University Hospital							Taipei	Taiwan
	Christchurch Hospital			Christchurch	New Zealand				
	Far Eastern Memorial Hospital							Taipei	Taiwan
	Mackay Memorial Hospital							Taipei	Taiwan
	National Cardiovascular Centre Harapan Kita							Jakarta	Indonesia
	Peoples' Hospital							Beijing	China
	Queen Elizabeth Hospital							Hong Kong	China
	Ramathibodi Hospital							Bangkok	Thailand
	Royal Brisbane and Women's Hospital			Brisbane	Australia				
	Severance Hospital							Seoul	Korea
	Singapore General Hospital							Singapore	Singapore
	Xuanwu Hospital							Beijing	China
BACC[352] (3500)[***]	University Heart Center Hamburg	Hamburg	Germany						
EDACS-ADP[353] (128)[***]	Christchurch Hospital			Christchurch	New Zealand				

Cohort name		Europe		Australasia		North America		Asia	
(N of participants)	Institutions	City	Country	City	Country	City	Country	City	Country
FASTEST[24] (605)	Uppsala University	Uppsala	Sweden						
	Karolinska Institute	Huddinge	Sweden						
	Karolinska University Hospital	Stockholm	Sweden						
FAST-MI[52] (1317)	University Heart Center Freiburg	Bad Krozingen	Germany						
Hamburg[28] (1818)	Federal Armed Forces Hospital Koblenz	Hamburg	Germany						
	Johannes Gutenberg-University Mainz	Hamburg	Germany						
	University Hospital Hamburg Eppendorf	Hamburg	Germany						
High STEACS[354] (1886)	Royal Infirmary of Edinburgh	Edinburgh	UK						
HIGH-US [78] (2346)	Baylor College of Medicine					Houston	USA		
	Beth Israel Deaconess					Boston	USA		
	Brigham and Women's					Boston	USA		
	Carolinas Medical Center					Charlotte	USA		
	Case Medical Center					Cleveland	USA		
	Detroit Receiving Wayne State					Detroit	USA		
	Duke University					Durham	USA		
	Hackensack University					Hackensack	USA		
	Hennepin County Medical Center					Minneapolis	USA		
	Henry Ford Hospital					Detroit	USA		
	Indiana University					Indianapolis	USA		
	Mayo Clinic					Rochester	USA		

For peer review only

Cohort name		Europe		Australasia		North America		Asia	
(N of participants)	Institutions	City	Country	City	Country	City	Country	City	Country
	New York Methodist					Brooklyn	USA		
	Research & Development Institute, Van Nuys; Providence Tarzana Medical Center					Tarzana	USA		
	Sinai Grace Wayne State					Detroit	USA		
	St. Vincent Hospital					Worcester	USA		
	Stony Brooks					Stony Brooks	USA		
	Tampa General Hopital					Tampa	USA		
	The Miriam hospital					Providence	USA		
	Thomas Jefferson University					Philadelphia	USA		
	UC Davis					Davis	USA		
	UC San Francisco					San Francisco	USA<br>USA		
	University of Cincinnati					Cincinnati	USA		
	University of Maryland					Baltimore	USA		
	University of New Mexico					Albuquerque	USA		
	University of Texas Medical Branch					Galveston	USA		
	University of Virginia					Charlottesvill e	USA		
	Virginia Common Health University					Richmond	USA		
	Wake Forrest University					Winston-Salem	USA		
IMPACT[355] (ND)	Royal Brisbane and Women's Hospital			Brisbane	Australia				
Italy[54] (6403)	Desio Hospital	Desio	Italy						

For peer review only

Cohort name		Europe		Australasia		North America		Asia	
(N of participants)	Institutions	City	Country	City	Country	City	Country	City	Country
Juntendo[48] (1074)***	Juntendo University Hospital							Tokyo	Japan
RACING-MI[79] (1003)	Randers Regional Hospital	Randers	Denmark						
RAPID-TnT [50] (1646)	Flinders Medical Centre - Bedford Park			Adelaide	Australia				
	The Royal Adelaide Hospital -			Adelaide	Australia				
	Noarlunga Health Service - Noarlunga Centre			Adelaide	Australia				
	Lyell McEwin Hospital - Elizabeth Vale			Adelaide	Australia				
	The Queen Elizabeth Hospital -			Woodville	Australia				
REACTION-US[80] (552)	Henry Ford Hopital					Detroit	USA		
RING[356] (148)***	McMaster University					Hamilton	Canada		
ROMI-3[83] (935)***	McMaster University					Hamilton	Canada		
Shanghai[57] (577)	Zhongshan Hospital							Shanghai	China
Singapore[66] (2444)	Singapore General Hospital								Singapore
Skåne[45 65] (1038)***	Skåne University Hospital	Lund	Sweden						
STOP CP[56] (1642)	UC Davis					Sacramento	USA		

For peer review only

Cohort name		Europe		Australasia		North America		Asia	
(N of participants)	Institutions	City	Country	City	Country	City	Country	City	Country
	University of Florida					Gainesville	USA		
	University of Maryland					Baltimore	USA		
	Henry Ford Health System					Detroit	USA		
	Wake Forest University					Winston-Salem	USA		
	University of Utah					Salt Lake City	USA		
TRAPID-AMI[47] (1282)***	Central Manchester U niversity Hospital	Manchester	UK						
	Cliniques University Saint-Luc (UCLouvain)	Brussel	Belgium						
	Henry Ford Health System					Detroit	USA		
	Institut d'Investigacions Biomèdiques Sant Pau	Barcelona	Spain						
	Karolinska Institute	Huddinge	Sweden						
	Liverpool Hosp ital and University New South Wales			Liverpool	Australia				
	Nuremberg Paracelsus Medical University	Nuremberg	Germany						
	University Hospital of Basal	Basel	Switzerland						
	Heidelberg University Hospital	Heidelberg	Germany						
	University Maryland					Baltimore	USA		
	Uppsala University	Uppsala	Sweden						
US multicenter[68] (1679)	Charleston Area Medical Center					Charleston	USA		
	Cleveland Clinic Foundation					Cleveland	USA		

For peer review only

Cohort name		Europe		Australasia		North America		Asia	
(N of participants)	Institutions	City	Country	City	Country	City	Country	City	Country
	Cooper University Hospital					Camden	USA		
	Duke University					Durham	USA		
	Health Science Center of Houston Medical School					Houston	USA		
	Henry Ford Health System					Detroit	USA		
	Indianapolis University					Indianapolis	USA		
	Mayo Clinic					Rochester	USA		
	Minneapolis Heart Institute Foundation					Minneapolis	USA		
	Newton Wellesley Hospital					Newton	USA		
	Prince George's Hospital					Maryland	USA		
	San Francisco General Hospital					San Francisco	USA		
	South Shore Hospital					Weymouth	USA		
	Thomas Jefferson University Hospital					Philadelphia	USA		
	University North Carolina					Chapel Hill	USA		
UTROPIA[357] (1631)	Hennepin County Medical Center					Minneapolis	USA		
WESTCOR[69] (932)	Haukeland University Hospital	Bergen	Norway						

ADAPT = 2-hour Accelerated Diagnostic Protocol to Assess Patients with Chest Pain Symptoms using Contemporary Troponins as the Only Biomarker; ADAPT–ADP = 2-hour Accelerated Diagnostic Protocol to Assess Patients with Chest Pain Symptoms using Contemporary Troponins as the Only Biomarker - Accelerated Diagnostic Pathway; AMI = acute myocardial infarction; APACE = Advantageous Predictors of Acute Coronary Syndromes Evaluation study; APPOACH = Alberta Provincial PRoject for Outcome Assessment in Coronary Heart Disease registry; ASPECT = Asia Pacific Evaluation of Chest pain Trial; BACC = Biomarkers in Acute Cardiac Care study; EDACS–ADP = Emergency Department Assessment of Chest Pain Score – Accelerated Diagnostic Pathway; FASTEST = Fast ASsessment of Thoracic pain in the Emergency department using high-Sensitive Troponins; High-STEACS = High-Sensitivity Troponin in the Evaluation of Patients

For peer review only

With Acute Coronary Syndrome; IMPACT = Improved Assessment of Chest Pain Trial; IQR = interquartile range; RACING-MI= Rapid use of high-sensitive cardiac troponin I for ruling-in and ruling-out of acute myocardial infarction; REACTION-US= Rapid Evaluation of ACuTe Myocardial InfarctiON in the US; RING = Reducing the Time Interval for Identifying New Guideline Defined Myocardial Infarction in Patients with Suspected Acute Coronary Syndrome; TRAPID–AMI = High-sensitivity cardiac Troponin T assay for RAPID rule-out of AMI; UTROPIA = Use of Abbott High Sensitivity Troponin I Assay In Acute Coronary Syndromes

***The largest number represented when multiple publications were derived from the same study cohort.

For peer review only

Appendix Figure 1. Geographical locations of eligible studies*

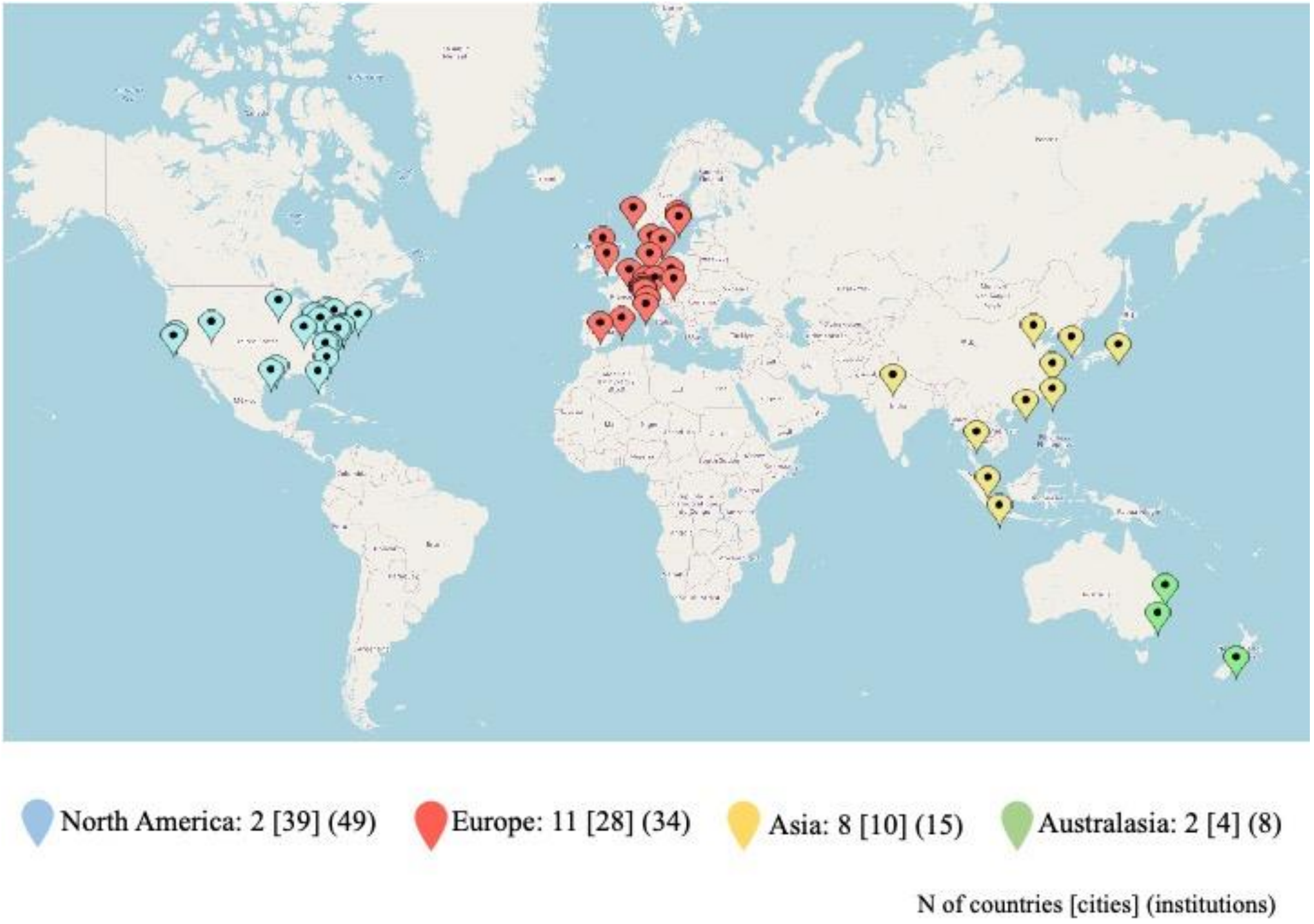

*Non-overlapping geographical regions (city as the unit) covered by the included research institution(s) are plotted. See Appendix Table 2 for details.

References

1. Rubini Gimenez M, Twerenbold R, Jaeger C, et al. One-hour rule-in and rule-out of acute myocardial infarction using high-sensitivity cardiac troponin I. *Am J Med* 2015;128(8):861-70 e4. doi: 10.1016/j.amjmed.2015.01.046
2. Pickering JW, Greenslade JH, Cullen L, et al. Assessment of the European Society of Cardiology 0-Hour/1-Hour Algorithm to Rule-Out and Rule-In Acute Myocardial Infarction. *Circulation* 2016;134(20):1532-41. doi: 10.1161/CIRCULATIONAHA.116.022677
3. Neumann JT, Sorensen NA, Schwemer T, et al. Diagnosis of Myocardial Infarction Using a High-Sensitivity Troponin I 1-Hour Algorithm. *JAMA Cardiol* 2016;1(4):397-404. doi: 10.1001/jamacardio.2016.0695
4. Boeddinghaus J, Nestelberger T, Twerenbold R, et al. Direct Comparison of 4 Very Early Rule-Out Strategies for Acute Myocardial Infarction Using High-Sensitivity Cardiac Troponin I. *Circulation* 2017;135(17):1597-611. doi: 10.1161/CIRCULATIONAHA.116.025661
5. Neumann JT, Sorensen NA, Ojeda F, et al. Early diagnosis of acute myocardial infarction using high-sensitivity troponin I. *PLoS One* 2017;12(3):e0174288. doi: 10.1371/journal.pone.0174288
6. Twerenbold R, Badertscher P, Boeddinghaus J, et al. 0/1-Hour Triage Algorithm for Myocardial Infarction in Patients With Renal Dysfunction. *Circulation* 2018;137(5):436-51. doi: 10.1161/CIRCULATIONAHA.117.028901
7. Twerenbold R, Neumann JT, Sorensen NA, et al. Prospective Validation of the 0/1-h Algorithm for Early Diagnosis of Myocardial Infarction. *J Am Coll Cardiol* 2018;72(6):620-32. doi: 10.1016/j.jacc.2018.05.040
8. Boeddinghaus J, Nestelberger T, Twerenbold R, et al. Impact of age on the performance of the ESC 0/1h-algorithms for early diagnosis of myocardial infarction. *Eur Heart J* 2018;39(42):3780-94. doi: 10.1093/eurheartj/ehy514
9. Wildi K, Boeddinghaus J, Nestelberger T, et al. Comparison of fourteen rule-out strategies for acute myocardial infarction. *Int J Cardiol* 2019;283:41-47. doi: 10.1016/j.ijcard.2018.11.140
10. Nestelberger T, Boeddinghaus J, Wussler D, et al. Predicting Major Adverse Events in Patients With Acute Myocardial Infarction. *J Am Coll Cardiol* 2019;74(7):842-54. doi: 10.1016/j.jacc.2019.06.025
11. Boeddinghaus J, Twerenbold R, Nestelberger T, et al. Clinical Use of a New High-Sensitivity Cardiac Troponin I Assay in Patients with Suspected Myocardial Infarction. *Clin Chem* 2019;65(11):1426-36. doi: 10.1373/clinchem.2019.304725
12. Neumann JT, Sorensen NA, Rubsamen N, et al. Evaluation of a new ultra-sensitivity troponin I assay in patients with suspected myocardial infarction. *Int J Cardiol* 2019;283:35-40. doi: 10.1016/j.ijcard.2018.12.001
13. Sorensen NA, Neumann JT, Ojeda F, et al. Diagnostic Evaluation of a High-Sensitivity Troponin I Point-of-Care Assay. *Clin Chem* 2019;65(12):1592-601. doi: 10.1373/clinchem.2019.307405
14. Boeddinghaus J, Nestelberger T, Koechlin L, et al. Early Diagnosis of Myocardial Infarction With Point-of-Care High-Sensitivity Cardiac Troponin I. *J Am Coll Cardiol* 2020;75(10):1111-24. doi: 10.1016/j.jacc.2019.12.065
15. Sörensen NA, Neumann JT, Ojeda F, et al. Differences in measurement of high-sensitivity troponin in an on-demand and batch-wise setting. *European Heart Journal Acute Cardiovascular Care* 2021;10(3):302-09. doi: 10.1177/2048872620924198
16. Sorensen NA, Gossling A, Neumann JT, et al. Diagnostic Validation of a High-Sensitivity Cardiac Troponin I Assay. *Clin Chem* 2021;67(9):1230-39. doi: 10.1093/clinchem/hvab070
17. Nestelberger T, Boeddinghaus J, Gimenez MR, et al. Direct comparison of high-sensitivity cardiac troponin T and I in

For peer review only

the early differentiation of type 1 vs. type 2 myocardial infarction. *Eur Heart J Acute Cardiovasc Care* 2022;11(1):62-74. doi: 10.1093/ehjacc/zuab039

18. Cullen L, Mueller C, Parsonage WA, et al. Validation of high-sensitivity troponin I in a 2-hour diagnostic strategy to assess 30-day outcomes in emergency department patients with possible acute coronary syndrome. *J Am Coll Cardiol* 2013;62(14):1242-49. doi: 10.1016/j.jacc.2013.02.078
19. Aldous S, Mark Richards A, George PM, et al. Comparison of new point-of-care troponin assay with high sensitivity troponin in diagnosing myocardial infarction. *Int J Cardiol* 2014;177(1):182-6. doi: 10.1016/j.ijcard.2014.09.026
20. Cullen L, Aldous S, Than M, et al. Comparison of high sensitivity troponin T and I assays in the diagnosis of non-ST elevation acute myocardial infarction in emergency patients with chest pain. *Clin Biochem* 2014;47(6):321-6. doi: 10.1016/j.clinbiochem.2013.11.019
21. Eggers KM, Aldous S, Greenslade JH, et al. Two-hour diagnostic algorithms for early assessment of patients with acute chest pain--Implications of lowering the cardiac troponin I cut-off to the 97.5th percentile. *Clin Chim Acta* 2015;445:19-24. doi: 10.1016/j.cca.2015.03.002
22. Boeddinghaus J, Reichlin T, Cullen L, et al. Two-Hour Algorithm for Triage toward Rule-Out and Rule-In of Acute Myocardial Infarction by Use of High-Sensitivity Cardiac Troponin I. *Clin Chem* 2016;62(3):494-504. doi: 10.1373/clinchem.2015.249508
23. Wildi K, Cullen L, Twerenbold R, et al. Direct Comparison of 2 Rule-Out Strategies for Acute Myocardial Infarction: 2-h Accelerated Diagnostic Protocol vs 2-h Algorithm. *Clin Chem* 2017;63(7):1227-36. doi: 10.1373/clinchem.2016.268359
24. Lindahl B, Jernberg T, Badertscher P, et al. An algorithm for rule-in and rule-out of acute myocardial infarction using a novel troponin I assay. *Heart* 2017;103(2):125-31. doi: 10.1136/heartjnl-2016-309951
25. Nestelberger T, Boeddinghaus J, Greenslade J, et al. Two-Hour Algorithm for Rapid Triage of Suspected Acute Myocardial Infarction Using a High-Sensitivity Cardiac Troponin I Assay. *Clin Chem* 2019;65(11):1437-47. doi: 10.1373/clinchem.2019.305193
26. Kavsak PA, Hewitt MK, Mondoux SE, et al. Diagnostic Performance of Serial High-Sensitivity Cardiac Troponin Measurements in the Emergency Setting. *J Cardiovasc Dev Dis* 2021;8(8) doi: 10.3390/jcdd8080097
27. Koechlin L, Boeddinghaus J, Nestelberger T, et al. Performance of the ESC 0/2h-algorithm using high-sensitivity cardiac troponin I in the early diagnosis of myocardial infarction. *Am Heart J* 2021;242:132-37. doi: 10.1016/j.ahj.2021.08.008
28. Keller T, Zeller T, Ojeda F, et al. Serial Changes in Highly Sensitive Troponin I Assay and Early Diagnosis of Myocardial Infarction. *JAMA* 2011;306(24):2684-93. doi: 10.1001/jama.2011.1896
29. Wildi K, Nelles B, Twerenbold R, et al. Safety and efficacy of the 0 h/3 h protocol for rapid rule out of myocardial infarction. *Am Heart J* 2016;181:16-25. doi: 10.1016/j.ahj.2016.07.013
30. Parsonage WA, Mueller C, Greenslade JH, et al. Validation of NICE diagnostic guidance for rule out of myocardial infarction using high-sensitivity troponin tests. *Heart* 2016;102(16):1279-86. doi: 10.1136/heartjnl-2016-309270
31. Pickering JW, Greenslade JH, Cullen L, et al. Validation of presentation and 3 h high-sensitivity troponin to rule-in and rule-out acute myocardial infarction. *Heart* 2016;102(16):1270-8. doi: 10.1136/heartjnl-2015-308505
32. Chapman AR, Anand A, Boeddinghaus J, et al. Comparison of the Efficacy and Safety of Early Rule-Out Pathways for

For peer review only

Acute Myocardial Infarction. *Circulation* 2017;135(17):1586-96. doi: 10.1161/CIRCULATIONAHA.116.025021

33. Sandoval Y, Smith SW, Thordsen SE, et al. Diagnostic Performance of High Sensitivity Compared with Contemporary Cardiac Troponin I for the Diagnosis of Acute Myocardial Infarction. *Clin Chem* 2017;63(10):1594-604. doi: 10.1373/clinchem.2017.272930
34. Chapman AR, Hesse K, Andrews J, et al. High-Sensitivity Cardiac Troponin I and Clinical Risk Scores in Patients With Suspected Acute Coronary Syndrome. *Circulation* 2018;138(16):1654-65. doi: 10.1161/CIRCULATIONAHA.118.036426
35. Gunsolus I, Sandoval Y, Smith SW, et al. Renal Dysfunction Influences the Diagnostic and Prognostic Performance of High-Sensitivity Cardiac Troponin I. *J Am Soc Nephrol* 2018;29(2):636-43. doi: 10.1681/ASN.2017030341
36. Kim JW, Kim H, Yun YM, et al. Absolute Change in High-Sensitivity Cardiac Troponin I at Three Hours After Presentation is Useful for Diagnosing Acute Myocardial Infarction in the Emergency Department. *Ann Lab Med* 2020;40(6):474-80. doi: 10.3343/alm.2020.40.6.474
37. Sandoval Y, Smith SW, Schulz K, et al. Comparison of 0/3-Hour Rapid Rule-Out Strategies Using High-Sensitivity Cardiac Troponin I in a US Emergency Department. *Circ Cardiovasc Qual Outcomes* 2020;13(7):e006565. doi: 10.1161/CIRCOUTCOMES.120.006565
38. Dupuy AM, Badiou S, Montagnon V, et al. Analytical assessment and performance of the 0/3h algorithm with novel high sensitivity cardiac troponin I. *Clin Chim Acta* 2021;519:111-17. doi: 10.1016/j.cca.2021.04.003
39. Rubini Gimenez M, Twerenbold R, Reichlin T, et al. Direct comparison of high-sensitivity-cardiac troponin I vs. T for the early diagnosis of acute myocardial infarction. *Eur Heart J* 2014;35(34):2303-11. doi: 10.1093/eurheartj/ehu188
40. Pickering JW, Young JM, George P, et al. The utility of presentation and 4-hour high sensitivity troponin I to rule-out acute myocardial infarction in the emergency department. *Clin Biochem* 2015;48(18):1219-24. doi: 10.1016/j.clinbiochem.2015.07.033
41. Miller-Hodges E, Anand A, Shah ASV, et al. High-Sensitivity Cardiac Troponin and the Risk Stratification of Patients With Renal Impairment Presenting With Suspected Acute Coronary Syndrome. *Circulation* 2018;137(5):425-35. doi: 10.1161/CIRCULATIONAHA.117.030320
42. Reichlin T, Irfan A, Twerenbold R, et al. Utility of absolute and relative changes in cardiac troponin concentrations in the early diagnosis of acute myocardial infarction. *Circulation* 2011;124(2):136-45. doi: 10.1161/CIRCULATIONAHA.111.023937
43. Reichlin T, Schindler C, Drexler B, et al. One-hour rule-out and rule-in of acute myocardial infarction using high-sensitivity cardiac troponin T. *Arch Intern Med* 2012;172(16):1211-8. doi: 10.1001/archinternmed.2012.3698
44. Reichlin T, Twerenbold R, Wildi K, et al. Prospective validation of a 1-hour algorithm to rule-out and rule-in acute myocardial infarction using a high-sensitivity cardiac troponin T assay. *CMAJ* 2015;187(8):E243-52. doi: 10.1503/cmaj.141349/-/DC1
45. Mokhtari A, Borna C, Gilje P, et al. A 1-h Combination Algorithm Allows Fast Rule-Out and Rule-In of Major Adverse Cardiac Events. *J Am Coll Cardiol* 2016;67(13):1531-40. doi: 10.1016/j.jacc.2016.01.059
46. Mokhtari A, Lindahl B, Schiopu A, et al. A 0-Hour/1-Hour Protocol for Safe, Early Discharge of Chest Pain Patients. *Acad Emerg Med* 2017;24(8):983-92. doi: 10.1111/acem.13224

For peer review only

47. Mueller C, Giannitsis E, Christ M, et al. Multicenter Evaluation of a 0-Hour/1-Hour Algorithm in the Diagnosis of Myocardial Infarction With High-Sensitivity Cardiac Troponin T. *Ann Emerg Med* 2016;68(1):76-87 e4. doi: 10.1016/j.annemergmed.2015.11.013

48. Shiozaki M, Inoue K, Suwa S, et al. Utility of the 0-hour/1-hour high-sensitivity cardiac troponin T algorithm in Asian patients with suspected non-ST elevation myocardial infarction. *Int J Cardiol* 2017;249:32-35. doi: 10.1016/j.ijcard.2017.09.009

49. Mueller-Hennessen M, Lindahl B, Giannitsis E, et al. Combined testing of copeptin and high-sensitivity cardiac troponin T at presentation in comparison to other algorithms for rapid rule-out of acute myocardial infarction. *Int J Cardiol* 2019;276:261-67. doi: 10.1016/j.ijcard.2018.10.084

50. Chew DP, Lambrakis K, Blyth A, et al. A Randomized Trial of a 1-Hour Troponin T Protocol in Suspected Acute Coronary Syndromes: The Rapid Assessment of Possible Acute Coronary Syndrome in the Emergency Department With High-Sensitivity Troponin T Study (RAPID-TnT). *Circulation* 2019;140(19):1543-56. doi: 10.1161/CIRCULATIONAHA.119.042891

51. Twerenbold R, Costabel JP, Nestelberger T, et al. Outcome of Applying the ESC 0/1-hour Algorithm in Patients With Suspected Myocardial Infarction. *J Am Coll Cardiol* 2019;74(4):483-94. doi: 10.1016/j.jacc.2019.05.046

52. Amann M, Gaiser F, Schwenk SI, et al. Evaluation of a 1-hour troponin algorithm for diagnosing myocardial infarction in high-risk patients admitted to a chest pain unit: the prospective FAST-MI cohort study. *BMJ Open* 2019;9(11):e032124. doi: 10.1136/bmjopen-2019-032124

53. Andruchow JE, Boyne T, Seiden-Long I, et al. Prospective comparative evaluation of the European Society of Cardiology (ESC) 1-hour and a 2-hour rapid diagnostic algorithm for myocardial infarction using high-sensitivity troponin-T. *CJEM* 2020;22(5):712-20. doi: 10.1017/cem.2020.349

54. Cappellini F, Falbo R, Saltafossi D, et al. Development of an algorithm for ruling-out non-ST elevation myocardial infarction in the emergency department using high sensitivity troponin T assay. *Clin Chim Acta* 2019;495:1-7. doi: 10.1016/j.cca.2019.03.1625

55. Shiozaki M, Inoue K, Suwa S, et al. Implementing the European Society of Cardiology 0-h/1-h algorithm in patients presenting very early after chest pain. *Int J Cardiol* 2020;320:1-6. doi: 10.1016/j.ijcard.2020.07.037

56. Allen BR, Christenson RH, Cohen SA, et al. Diagnostic Performance of High-Sensitivity Cardiac Troponin T Strategies and Clinical Variables in a Multisite US Cohort. *Circulation* 2021;143(17):1659-72. doi: 10.1161/CIRCULATIONAHA.120.049298

57. Dongxu C, Yannan Z, Yilin Y, et al. Evaluation of the 0 h/1 h high-sensitivity cardiac troponin T algorithm in diagnosis of non-ST-segment elevation myocardial infarction (NSTEMI) in Han population. *Clin Chem Lab Med* 2021;59(4):757-64. doi: 10.1515/cclm-2020-0367

58. Lopez-Ayala P, Nestelberger T, Boeddinghaus J, et al. Novel Criteria for the Observe-Zone of the ESC 0/1h-hs-cTnT Algorithm. *Circulation* 2021;144(10):773-87. doi: 10.1161/CIRCULATIONAHA.120.052982

59. Olsson P, Khoshnood A, Mokhtari A, et al. Glucose and high-sensitivity troponin T predict a low risk of major adverse cardiac events in emergency department chest pain patients. *Scand Cardiovasc J* 2021;55(6):354-61. doi: 10.1080/14017431.2021.1987512

60. Ruangsomboon O, Thirawattanasoot N, Chakorn T, et al. The utility of the 1-hour high-sensitivity cardiac troponin T

For peer review only

algorithm compared with and combined with five early rule-out scores in high-acuity chest pain emergency patients. *Int J Cardiol* 2021;322:23-28. doi: 10.1016/j.ijcard.2020.08.099

61. Aldous SJ, Richards AM, Cullen L, et al. Early dynamic change in high-sensitivity cardiac troponin T in the investigation of acute myocardial infarction. *Clin Chem* 2011;57(8):1154-60. doi: 10.1373/clinchem.2010.161166
62. Parsonage WA, Greenslade JH, Hammett CJ, et al. Validation of an accelerated high-sensitivity troponin T assay protocol in an Australian cohort with chest pain. *Med J Aust* 2014;200(3):161-5. doi: 10.5694/mja13.10466
63. Reichlin T, Cullen L, Parsonage WA, et al. Two-hour algorithm for triage toward rule-out and rule-in of acute myocardial infarction using high-sensitivity cardiac troponin T. *Am J Med* 2015;128(4):369-79 e4. doi: 10.1016/j.amjmed.2014.10.032
64. McRae AD, Innes G, Graham M, et al. Undetectable Concentrations of a Food and Drug Administration-approved High-sensitivity Cardiac Troponin T Assay to Rule Out Acute Myocardial Infarction at Emergency Department Arrival. *Acad Emerg Med* 2017;24(10):1267-77. doi: 10.1111/acem.13229
65. Borna C, Kollberg K, Larsson D, et al. The objective CORE score allows early rule out in acute chest pain patients. *Scand Cardiovasc J* 2018;52(6):308-14. doi: 10.1080/14017431.2018.1546891
66. Lin Z, Lim SH, Chua SJT, et al. High-sensitivity troponin T and long-term adverse cardiac events among patients presenting with suspected acute coronary syndrome in Singapore. *Singapore Med J* 2019;60(8):418-26. doi: 10.11622/smedj.2019013
67. Wildi K, Lopez-Ayala P, Koechlin L, et al. Validation of the Novel European Society of Cardiology 0/2-hour Algorithm Using Hs-cTnT in the Early Diagnosis of Myocardial Infarction. *Am J Cardiol* 2021;154:128-30. doi: 10.1016/j.amjcard.2021.06.003
68. Peacock WF, Baumann BM, Bruton D, et al. Efficacy of High-Sensitivity Troponin T in Identifying Very-Low-Risk Patients With Possible Acute Coronary Syndrome. *JAMA Cardiol* 2018;3(2):104-11. doi: 10.1001/jamacardio.2017.4625
69. Steiro OT, Tjora HL, Langorgen J, et al. Clinical risk scores identify more patients at risk for cardiovascular events within 30 days as compared to standard ACS risk criteria: the WESTCOR study. *Eur Heart J Acute Cardiovasc Care* 2021;10(3):287-301. doi: 10.1093/ehjacc/zuaa016
70. Aldous SJ, Florkowski CM, Crozier IG, et al. Comparison of high sensitivity and contemporary troponin assays for the early detection of acute myocardial infarction in the emergency department. *Ann Clin Biochem* 2011;48(Pt 3):241-8. doi: 10.1258/acb.2010.010219
71. Aldous S, Pemberton C, Richards AM, et al. High-sensitivity troponin T for early rule-out of myocardial infarction in recent onset chest pain. *Emerg Med J* 2012;29(10):805-10. doi: 10.1136/emermed-2011-200222
72. Irfan A, Reichlin T, Twerenbold R, et al. Early diagnosis of myocardial infarction using absolute and relative changes in cardiac troponin concentrations. *Am J Med* 2013;126(9):781-88 e2. doi: 10.1016/j.amjmed.2013.02.031
73. Biener M, Mueller M, Vafaie M, et al. Impact of leading presenting symptoms on the diagnostic performance of high-sensitivity cardiac troponin T and on outcomes in patients with suspected acute coronary syndrome. *Clin Chem* 2015;61(5):744-51. doi: 10.1373/clinchem.2014.235317
74. Nowak RM, Gandolfo CM, Jacobsen G, et al. Ultrarapid Rule-out for Acute Myocardial Infarction Using the Generation 5 Cardiac Troponin T Assay: Results From the REACTION-US Study. *Ann Emerg Med*

For peer review only

2018;72(6):654-64. doi: 10.1016/j.annemergmed.2018.06.021

75. Jaeger C, Wildi K, Twerenbold R, et al. One-hour rule-in and rule-out of acute myocardial infarction using high-sensitivity cardiac troponin I. *Am Heart J* 2016;171(1):92-102 e1-5. doi: 10.1016/j.ahj.2015.07.022

76. Boeddinghaus J, Twerenbold R, Nestelberger T, et al. Clinical Validation of a Novel High-Sensitivity Cardiac Troponin I Assay for Early Diagnosis of Acute Myocardial Infarction. *Clin Chem* 2018;64(9):1347-60. doi: 10.1373/clinchem.2018.286906

77. Chapman AR, Fujisawa T, Lee KK, et al. Novel high-sensitivity cardiac troponin I assay in patients with suspected acute coronary syndrome. *Heart* 2019;105(8):616-22. doi: 10.1136/heartjnl-2018-314093

78. Nowak RM, Christenson RH, Jacobsen G, et al. Performance of Novel High-Sensitivity Cardiac Troponin I Assays for 0/1-Hour and 0/2- to 3-Hour Evaluations for Acute Myocardial Infarction: Results From the HIGH-US Study. *Ann Emerg Med* 2020;76(1):1-13. doi: 10.1016/j.annemergmed.2019.12.008

79. Andersen CF, Bang C, Lauridsen KG, et al. External validation of a high-sensitive troponin I algorithm for rapid evaluation of acute myocardial infarction in a Danish cohort. *Eur Heart J Acute Cardiovasc Care* 2021;10(9):1056-64. doi: 10.1093/ehjacc/zuab062

80. McCord J, Hana A, Cook B, et al. The role of cardiac testing with the 0/1-hour high-sensitivity cardiac troponin algorithm evaluating for acute myocardial infarction. *Am Heart J* 2021;233:68-77. doi: 10.1016/j.ahj.2020.12.015

81. Greenslade JH, Carlton EW, Van Hise C, et al. Diagnostic Accuracy of a New High-Sensitivity Troponin I Assay and Five Accelerated Diagnostic Pathways for Ruling Out Acute Myocardial Infarction and Acute Coronary Syndrome. *Ann Emerg Med* 2018;71(4):439-51 e3. doi: 10.1016/j.annemergmed.2017.10.030

82. Peacock WF, Christenson R, Diercks DB, et al. Myocardial Infarction Can Be Safely Excluded by High-sensitivity Troponin I Testing 3 Hours After Emergency Department Presentation. *Acad Emerg Med* 2020 doi: 10.1111/acem.13922

83. Kavsak PA, Mondoux SE, Ma J, et al. Comparison of two biomarker only algorithms for early risk stratification in patients with suspected acute coronary syndrome. *Int J Cardiol* 2020;319:140-43. doi: 10.1016/j.ijcard.2020.06.066

84. Schofer N, Brunner F, Schlüter M, et al. Gender-specific diagnostic performance of a new highsensitivity cardiac troponin I assay for detection of acute myocardial infarction. *Eur Heart J* 2017;6(1):60-68. doi: 10.1177/2048872615626660

85. Kavsak PA, Mondoux SE, Sherbino J, et al. Clinical evaluation of Ortho Clinical Diagnostics high-sensitivity cardiac Troponin I assay in patients with symptoms suggestive of acute coronary syndrome. *Clin Biochem* 2020;80:48-51. doi: 10.1016/j.clinbiochem.2020.04.003

86. Schreiber DH, Agbo C, Wu AH. Short-term (90 min) diagnostic performance for acute non-ST segment elevation myocardial infarction and 30-day prognostic evaluation of a novel third-generation high sensitivity troponin I assay. *Clin Biochem* 2012;45(16-17):1295-301. doi: 10.1016/j.clinbiochem.2012.06.005

87. Agewall S, Olsson T, Lowbeer C. Usefulness of troponin levels below the diagnostic cut-off level for acute myocardial infarction in predicting prognosis in unselected patients admitted to the coronary care unit. *Am J Cardiol* 2007;99(10):1357-9. doi: 10.1016/j.amjcard.2006.12.059

88. Ahmadi AA, Alemzadeh-Ansari MJ, Jenab Y, et al. Comparison of High-Sensitive Cardiac Troponin-T Changes

For peer review only

between Non-ST-Segment Elevation Myocardial Infarction and Non-Coronary Artery Patients. *Iranian Red Crescent Medical Journal* 2017;19(10) doi: 10.5812/ircmj.55663

89. Alcalai R, Varshisky B, Marhig A, et al. Assessing the Performance of a Novel Point-of-Care Qualitative Assay for Early Diagnosis of Acute Coronary Syndrome. *Cardiology* 2021;146(1):34-41. doi: 10.1159/000511435

90. Aldous SJ, Florkowski CM, Crozier IG, et al. The performance of high sensitivity troponin for the diagnosis of acute myocardial infarction is underestimated. *Clin Chem Lab Med* 2011;50(4):727-9. doi: 10.1515/cclm.2011.830

91. Aldous SJ, Richards MA, Cullen L, et al. A new improved accelerated diagnostic protocol safely identifies low-risk patients with chest pain in the emergency department. *Acad Emerg Med* 2012;19(5):510-6. doi: 10.1111/j.1553-2712.2012.01352.x

92. Aldous S, Richards M, Cullen L, et al. Diagnostic and prognostic utility of early measurement with high-sensitivity troponin T assay in patients presenting with chest pain. *CMAJ* 2012;184(5):E260-8. doi: 10.1503/cmaj.110773

93. Alghamdi A, Reynard C, Morris N, et al. Diagnostic accuracy of the Troponin-only Manchester Acute Coronary Syndromes (T-MACS) decision aid with a point-of-care cardiac troponin assay. *Emerg Med J* 2020;37(4):223-28. doi: 10.1136/emermed-2019-208882

94. Al-Saleh A, Alazzoni A, Al Shalash S, et al. Performance of the high-sensitivity troponin assay in diagnosing acute myocardial infarction: systematic review and meta-analysis. *CMAJ Open* 2014;2(3):E199-207. doi: 10.9778/cmajo.20130074

95. Alushi B, Jost-Brinkmann F, Kastrati A, et al. High-Sensitivity Cardiac Troponin T in Patients with Severe Chronic Kidney Disease and Suspected Acute Coronary Syndrome. *J Clin Med* 2021;10(18) doi: 10.3390/jcm10184216

96. Anand A, Lee KK, Chapman AR, et al. High-Sensitivity Cardiac Troponin on Presentation to Rule Out Myocardial Infarction: A Stepped-Wedge Cluster Randomized Controlled Trial. *Circulation* 2021;143(23):2214-24. doi: 10.1161/CIRCULATIONAHA.120.052380

97. Apple F, Ler R, Chung A, et al. Point-of-care i-STAT cardiac troponin I for assessment of patients with symptoms suggestive of acute coronary syndrome. *Clin Chem* 2006;52(2):322-5

98. Apple FS, Pearce LA, Smith SW, et al. Role of monitoring changes in sensitive cardiac troponin I assay results for early diagnosis of myocardial infarction and prediction of risk of adverse events. *Clin Chem* 2009;55(5):930-7. doi: 10.1373/clinchem.2008.114728

99. Astley CM, Beltrame JF, Zeitz C, et al. Study design of embracing high-sensitivity troponin effectively: the value of more information: a randomized comparison. *Contemp Clin Trials* 2014;39(2):183-90. doi: 10.1016/j.cct.2014.08.012

100. Ter Avest E, Visser A, Reitsma B, et al. Point-of-care troponin T is inferior to high-sensitivity troponin T for ruling out acute myocardial infarction in the emergency department. *Eur J Emerg Med* 2016;23(2):95-101. doi: 10.1186/cc10270

101. Bahrmann P, Heppner HJ, Christ M, et al. Early detection of non-ST-elevation myocardial infarction in geriatric patients by a new high-sensitive cardiac troponin T assay. *Aging Clin Exp Res* 2012;24(3):290-4. doi: 10.3275/7927

102. Bahrmann P, Christ M, Bahrmann A, et al. A 3-hour diagnostic algorithm for non-ST-elevation myocardial infarction using high-sensitivity cardiac troponin T in unselected older patients presenting to the emergency department. *J*

For peer review only

Am Med Dir Assoc 2013;14(6):409-16. doi: 10.1016/j.jamda.2012.12.005

103. Bahrmann P, Bertsch T, Sieber CC, et al. Management of patients with chest pain presenting to the emergency department: in need for the implementation of the 1 h rapid rule-out algorithm using high-sensitivity troponin I assays in clinical practice. *Ann Transl Med* 2016;4(1):18. doi: 10.3978/j.issn.2305-5839.2015.12.31

104. Bali T, Boda S, Reynard C, et al. The association between cardiac risk factors and the probability of acute myocardial infarction in the emergency department: analysis from a multicentre prospective observational study in the high sensitivity troponin era. *Eur J Emerg Med* 2021;28(3):233-7. doi: 10.1097/MEJ.0000000000000809.

105. Ballocca F, D'Ascenzo F, Moretti C, et al. High sensitive TROponin levels In Patients with Chest pain and kidney disease: A multicenter registry - The TROPIC study. *Cardiol J* 2017;24(2):139-50. doi: 10.5603/CJ.a2017.0025

106. Bandstein N, Ljung R, Johansson M, et al. Undetectable high-sensitivity cardiac troponin T level in the emergency department and risk of myocardial infarction. *J Am Coll Cardiol* 2014;63(23):2569-78. doi: 10.1016/j.jacc.2014.03.017

107. Bandstein N, Ljung R, Holzmann MJ. Risk of revisits to the emergency department in admitted versus discharged patients with chest pain but without myocardial infarction in relation to high-sensitivity cardiac troponin T levels. *Int J Cardiol* 2016;203:341-6. doi: 10.1016/j.ijcard.2015.10.170

108. Bandstein N, Wikman A, Ljung R, et al. Survival and resource utilization in patients with chest pain evaluated with cardiac troponin T compared with high-sensitivity cardiac troponin T. *Int J Cardiol* 2017;245:43-48. doi: 10.1016/j.ijcard.2017.05.111

109. Bellini C, Cinci F, Bova G, et al. Methodology to Evaluate Clinical Impact of 0/3 Hour High-Sensitivity Cardiac Troponin T Protocol on Managing Acute Coronary Syndrome in Daily Emergency Department Practice. *Lab Med* 2021;52(5):452-59. doi: 10.1093/labmed/lmaa118

110. Bhardwaj A, Truong QA, Peacock WF, et al. A multicenter comparison of established and emerging cardiac biomarkers for the diagnostic evaluation of chest pain in the emergency department. *Am Heart J* 2011;162(2):276-82 e1. doi: 10.1016/j.ahj.2011.05.022

111. Biener M, Mueller M, Vafaie M, et al. Comparison of a 3-hour versus a 6-hour sampling-protocol using high-sensitivity cardiac troponin T for rule-out and rule-in of non-STEMI in an unselected emergency department population. *Int J Cardiol* 2013;167(4):1134-40. doi: 10.1016/j.ijcard.2012.09.122

112. Birkhahn RH, Haines E, Wen W, et al. Estimating the clinical impact of bringing a multimarker cardiac panel to the bedside in the ED. *Am J Emerg Med* 2011;29(3):304-8. doi: 10.1016/j.ajem.2009.12.007

113. Bjurman C, Larsson M, Johanson P, et al. Small changes in troponin T levels are common in patients with non-ST-segment elevation myocardial infarction and are linked to higher mortality. *J Am Coll Cardiol* 2013;62(14):1231-38. doi: 10.1016/j.jacc.2013.06.050

114. Body R, Carley S, McDowell G, et al. Rapid exclusion of acute myocardial infarction in patients with undetectable troponin using a high-sensitivity assay. *J Am Coll Cardiol* 2011;58(13):1332-9. doi: 10.1016/j.jacc.2011.06.026

115. Body R, Burrows G, Carley S, et al. High-sensitivity cardiac troponin t concentrations below the limit of detection to exclude acute myocardial infarction: a prospective evaluation. *Clin Chem* 2015;61(7):983-9. doi: 10.1373/clinchem.2014.231530

116. Body R, Burrows G, Carley S, et al. Rapid exclusion of acute myocardial infarction in patients with undetectable

For peer review only

troponin using a sensitive troponin I assay. *Ann Clin Biochem* 2015;52(Pt 5):543-9. doi: 10.1177/0004563215576976

117. Body R, Mueller C, Giannitsis E, et al. The Use of Very Low Concentrations of High-sensitivity Troponin T to Rule Out Acute Myocardial Infarction Using a Single Blood Test. *Acad Emerg Med* 2016;23(9):1004-13. doi: 10.1111/acem.13012
118. Body R, Morris N, Reynard C, et al. Comparison of four decision aids for the early diagnosis of acute coronary syndromes in the emergency department. *Emerg Med J* 2020;37(1):8-13. doi: 10.1136/emermed-2019-208898
119. Boeckel JN, Palapies L, Zeller T, et al. Estimation of Values below the Limit of Detection of a Contemporary Sensitive Troponin I Assay Improves Diagnosis of Acute Myocardial Infarction. *Clin Chem* 2015;61(9):1197-206. doi: 10.1373/clinchem.2015.238949
120. Boeddinghaus J, Reichlin T, Nestelberger T, et al. Early diagnosis of acute myocardial infarction in patients with mild elevations of cardiac troponin. *Clin Res Cardiol* 2017;106(6):457-67. doi: 10.1007/s00392-016-1075-9
121. Bonaca MP, Ruff CT, Kosowsky J, et al. Evaluation of the diagnostic performance of current and next-generation assays for cardiac troponin I in the BWH-TIMI ED Chest Pain Study. *Eur Heart J Acute Cardiovasc Care* 2013;2(3):195-202. doi: 10.1177/2048872613486249
122. Borna C, Thelin J, Ohlin B, et al. High-sensitivity troponin T as a diagnostic tool for acute coronary syndrome in the real world: an observational study. *Eur J Emerg Med* 2014;21(3):181-8. doi: 10.1097/MEJ.0b013e328362a71b
123. Borna C, Frostred KL, Ekelund U. Predictive role of high sensitivity troponin T within four hours from presentation of acute coronary syndrome in elderly patients. *BMC Emerg Med* 2016;16:1. doi: 10.1186/s12873-015-0064-z
124. Bove J, Hochman S, Miller J, et al. Effectiveness of 2-hour Troponin in High-risk Patients With Suspected Acute Coronary Syndrome. *Crit Pathw Cardiol* 2017;16(2):53-57. doi: 10.1097/HPC.0000000000000111
125. Braga F, Dolci A, Cavallero A, et al. Evaluation of the sensitivity of two highly sensitive troponin assays for early detection of non ST-elevation myocardial infarction (NSTEMI). *Biochimica Clinica* 2011;35(3):186-89.
126. Brichko L, Schneider HG, Chan W, et al. Rapid and safe discharge from the emergency department: A single troponin to exclude acute myocardial infarction. *Emerg Med Australas* 2018;30(4):486-93. doi: 10.1111/1742-6723.12919
127. Bularga A, Lee KK, Stewart S, et al. High-Sensitivity Troponin and the Application of Risk Stratification Thresholds in Patients With Suspected Acute Coronary Syndrome. *Circulation* 2019;140(19):1557-68. doi: 10.1161/CIRCULATIONAHA.119.042866
128. Caglar B, Serin S. Diagnostic Performance of One-hour Delta High Sensitive Troponin in Acute Coronary Syndrome. *J Coll Physicians Surg Pak* 2020;30(9):906-11. doi: 10.29271/jcpsp.2020.09.906
129. Carlton E, Greenslade J, Cullen L, et al. Evaluation of High-Sensitivity Cardiac Troponin I Levels in Patients With Suspected Acute Coronary Syndrome. *JAMA Cardiol* 2016;1(4):405-12. doi: 10.1001/jamacardio.2016.1309
130. Carlton E, Khattab A, Greaves K. Beyond triage: the diagnostic accuracy of emergency department nursing staff risk assessment in patients with suspected acute coronary syndromes. *Emerg Med j* 2016;33(2):99-104. doi: 10.1136/emermed-2015-20478
131. Carlton EW, Pickering JW, Greenslade J, et al. Assessment of the 2016 National Institute for Health and Care Excellence high-sensitivity troponin rule-out strategy. *Heart* 2018;104(8):665-72. doi: 10.1136/heartjnl-2017-311983

For peer review only

132. Carlton EW, Ingram J, Taylor H, et al. Limit of detection of troponin discharge strategy versus usual care: randomised controlled trial. *Heart* 2020;106(20):1586-94. doi: 10.1136/heartjnl-2020-316692

133. Cavender MA, White WB, Jarolim P, et al. Serial Measurement of High-Sensitivity Troponin I and Cardiovascular Outcomes in Patients With Type 2 Diabetes Mellitus in the EXAMINE Trial (Examination of Cardiovascular Outcomes With Alogliptin Versus Standard of Care). *Circulation* 2017;135(20):1911-21. doi: 10.1161/CIRCULATIONAHA.116.024632

134. Celik S, Giannitsis E, Wollert KC, et al. Cardiac troponin T concentrations above the 99th percentile value as measured by a new high-sensitivity assay predict long-term prognosis in patients with acute coronary syndromes undergoing routine early invasive strategy. *Clin Res Cardiol* 2011;100(12):1077-85. doi: 10.1007/s00392-011-0344-x

135. Chapman AR, Lee KK, McAllister DA, et al. Association of High-Sensitivity Cardiac Troponin I Concentration With Cardiac Outcomes in Patients With Suspected Acute Coronary Syndrome. *JAMA* 2017;318(19):1913-24. doi: 10.1001/jama.2017.17488

136. Charpentier S, Chenevier-Gobeaux C. [2015 ESC guidelines: 1-hour rule-out and rule-in of acute myocardial infarction with high-sensitive troponin T]. *Presse Med* 2016;45(10):859-64. doi: 10.1016/j.lpm.2016.05.023

137. Chenevier-Gobeaux C, Meune C, Lefevre G, et al. A single value of high-sensitive troponin T below the limit of detection is not enough for ruling out non ST elevation myocardial infarction in the emergency department. *Clin Biochem* 2016;49(15):1113-17. doi: 10.1016/j.clinbiochem.2016.05.021

138. Chew DP, Zeitz C, Worthley M, et al. Randomized Comparison of High-Sensitivity Troponin Reporting in Undifferentiated Chest Pain Assessment. *Circ Cardiovasc Qual Outcomes* 2016;9(5):542-53. doi: 10.1161/CIRCOUTCOMES.115.002488

139. Chew G, Frost F, Mullen L, et al. A direct comparison of decision rules for early discharge of suspected acute coronary syndromes in the era of high sensitivity troponin. *Eur Heart J Acute Cardiovasc Care* 2018;8(5):421-31. doi: 10.1177/2048872618755369

140. Christ M, Popp S, Pohlmann H, et al. Implementation of high sensitivity cardiac troponin T measurement in the emergency department. *Am J Med* 2010;123(12):1134-42. doi: 10.1016/j.amjmed.2010.07.015

141. Christ M, Geier F, Blaschke S, et al. Clinical performance of a new point-of-care cardiac troponin I test. *Clin Chem Lab Med* 2018;56(8):1336-44. doi: 10.1515/cclm-2017-0693

142. Christenson RH, Duh SH, Apple FA, et al. Pivotal findings for a high-sensitivity cardiac troponin assay: Results of the HIGH-US study. *Clin Biochem* 2020;78:32-39. doi: 10.1016/j.clinbiochem.2019.10.013

143. Chuang A, Hancock DG, Horsfall M, et al. The predictive value of high sensitivity-troponin velocity within the first 6h of presentation for cardiac outcomes regardless of acute coronary syndrome diagnosis. *Int J Cardiol* 2016;204:106-11. doi: 10.1016/j.ijcard.2015.11.132

144. Clerico A, Ripoli A, Zaninotto M, et al. Head-to-head comparison of plasma cTnI concentration values measured with three high-sensitivity methods in a large Italian population of healthy volunteers and patients admitted to emergency department with acute coronary syndrome: A multi-center study. *Clin Chim Acta* 2019;496:25-34. doi: 10.1016/j.cca.2019.06.012

145. Čolak T, Mikulić I, Landeka K, et al. Predictive Value of High Sensitive Troponin I Assay in Acute Coronary Syndrome Compared to Classic Biochemical Markers. *Psychiatr Danub* 2017;29:823-29.

For peer review only - http://bmjopen.bmj.com/site/about/guidelines.xhtml

146. Collinson P, Gaze D, Morris B, et al. Comparison of biomarker strategies for rapid rule out of myocardial infarction in the emergency department using ACC/ESC diagnostic criteria. *Ann Clin Biochem* 2006;43(4):273-80.

147. Collinson PO, Gaze DC, Bainbridge K, et al. Utility of admission cardiac troponin and "Ischemia Modified Albumin" measurements for rapid evaluation and rule out of suspected acute myocardial infarction in the emergency department. *Emerg Med J* 2006;23(4):256-61. doi: 10.1136/emj.2005.028241

148. Collinson PO, Gaze D, Goodacre S. The clinical and diagnostic performance characteristics of the high sensitivity Abbott cardiac troponin I assay. *Clin Biochem* 2015;48(4-5):275-81. doi: 10.1016/j.clinbiochem.2014.12.017

149. Unger P, Dedobbeleer C, Vanden Eynden F, et al. Mitral regurgitation after transcatheter aortic valve replacement: does the prosthesis matter? *Int J Cardiol* 2013;168(2):1706-9. doi: 10.1016/j.ijcard.2013.03.055

150. Cook B, McCord J, Hudson M, et al. Baseline High Sensitivity Cardiac Troponin I Level Below Limit of Quantitation Rules Out Acute Myocardial Infarction in the Emergency Department. *Crit Pathw Cardiol* 2021;20(1):4-9. doi: 10.1097/HPC.0000000000000230

151. Corsini A, Vagnarelli F, Bugani G, et al. Impact of high-sensitivity Troponin T on hospital admission, resources utilization, and outcomes. *Eur Heart J Acute Cardiovasc Care* 2015;4(2):148-57. doi: 10.1177/2048872614547687

152. Cramer GE, Kievit PC, Brouwer MA, et al. Lack of concordance between a rapid bedside and conventional laboratory method of cardiac troponin testing: impact on risk stratification of patients suspected of acute coronary syndrome. *Clin Chim Acta* 2007;381(2):164-6. doi: 10.1016/j.cca.2007.03.001

153. Cullen L, Parsonage WA, Greenslade J, et al. Delta troponin for the early diagnosis of AMI in emergency patients with chest pain. *Int J Cardiol* 2013;168(3):2602-8. doi: 10.1016/j.ijcard.2013.03.044

154. Cullen L, Greenslade JH, Carlton EW, et al. Sex-specific versus overall cut points for a high sensitivity troponin I assay in predicting 1-year outcomes in emergency patients presenting with chest pain. *Heart* 2016;102(2):120-6. doi: 10.1136/heartjnl-2015-308506

155. Dadkhah S, Almuwaqqat Z, Sulaiman S, et al. Sensitive Troponin I and Stress Testing in the Emergency Department for the Early Management of Chest Pain Using 2-Hour Protocol. *Crit Pathw Cardiol* 2017;16(3):89-92. doi: 10.1097/HPC.0000000000000115

156. Daly MJ, Adgey JA, Harbinson MT. Improved detection of acute myocardial infarction in patients with chest pain and significant left main stem coronary stenosis. *QJM* 2012;105(2):127-35. doi: 10.1093/qjmed/hcr134

157. Davarani H, Afzalimoghadam M, Hosseinnejad H, et al. Increasing serum troponin I and early prognosis in patients with chest pain or angina equivalent symptoms in the emergency department. *Iran J Public Health* 2012;41(2):63-69.

158. Dawson C, Benger JR, Bayly G. Serial high-sensitivity troponin measurements for the rapid exclusion of acute myocardial infarction in low-risk patients. *Emerg Med J* 2013;30(7):593-4. doi: 10.1136/emermed-2012-201574

159. Diercks DB, Peacock WFt, Hollander JE, et al. Diagnostic accuracy of a point-of-care troponin I assay for acute myocardial infarction within 3 hours after presentation in early presenters to the emergency department with chest pain. *Am Heart J* 2012;163(1):74-80 e4. doi: 10.1016/j.ahj.2011.09.028

160. Druey S, Wildi K, Twerenbold R, et al. Early rule-out and rule-in of myocardial infarction using sensitive cardiac Troponin I. *Int J Cardiol* 2015;195:163-70. doi: 10.1016/j.ijcard.2015.05.079

For peer review only

161. Eggers KM, Jaffe AS, Venge P, et al. Clinical implications of the change of cardiac troponin I levels in patients with acute chest pain - an evaluation with respect to the Universal Definition of Myocardial Infarction. *Clin Chim Acta* 2011;412(1-2):91-7. doi: 10.1016/j.cca.2010.09.020

162. Etaher A, Chew DP, Frost S, et al. Prognostic Implications of High-Sensitivity Troponin T Levels Among Patients Attending Emergency Departments and Evaluated for an Acute Coronary Syndrome. *Am J Med* 2021;134(8):1019-28 e1. doi: 10.1016/j.amjmed.2021.03.005

163. Fan LY, Yu P, Yu SS, et al. Age-specific 99th percentile cutoff of high-sensitivity cardiac troponin T for early prediction of non-ST-segment elevation myocardial infarction (NSTEMI) in middle-aged patients. *J Clin Lab Anal* 2014;28(1):10-5. doi: 10.1002/jcla.21636

164. Fanaroff AC, Schulteis RD, Pieper KS, et al. Simplified Predictive Instrument to Rule Out Acute Coronary Syndromes in a High-Risk Population. *J Am Heart Assoc* 2015;4(12) doi: 10.1161/JAHA.115.002351

165. Freund Y, Chenevier-Gobeaux C, Bonnet P, et al. High-sensitivity versus conventional troponin in the emergency department for the diagnosis of acute myocardial infarction. *Crit Care* 2011

166. Frisoli TM, Nowak R, Evans KL, et al. Henry Ford HEART Score Randomized Trial: Rapid Discharge of Patients Evaluated for Possible Myocardial Infarction. *Circ Cardiovasc Qual Outcomes* 2017;10(10) doi: 10.1161/CIRCOUTCOMES.117.003617

167. Gassenmaier T, Buchner S, Birner C, et al. High-sensitive Troponin I in acute cardiac conditions: implications of baseline and sequential measurements for diagnosis of myocardial infarction. *Atherosclerosis* 2012;222(1):116-22. doi: 10.1016/j.atherosclerosis.2012.02.007

168. Giannitsis E, Becker M, Kurz K, et al. High-sensitivity cardiac troponin T for early prediction of evolving non-ST-segment elevation myocardial infarction in patients with suspected acute coronary syndrome and negative troponin results on admission. *Clin Chem* 2010;56(4):642-50. doi: 10.1373/clinchem.2009.134460

169. Giannitsis E, Kurz K, Hallermayer K, et al. Analytical validation of a high-sensitivity cardiac troponin T assay. *Clin Chem* 2010;56(2):254-61. doi: 10.1373/clinchem.2009.132654

170. Rubini Gimenez M, Twerenbold R, Boeddinghaus J, et al. Clinical Effect of Sex-Specific Cutoff Values of High-Sensitivity Cardiac Troponin T in Suspected Myocardial Infarction. *JAMA Cardiol* 2016;1(8):912-20. doi: 10.1001/jamacardio.2016.2882

171. Rubini Gimenez M, Wildi K, Wussler D, et al. Early kinetics of cardiac troponin in suspected acute myocardial infarction. *Rev Esp Cardiol (Engl Ed)* 2020 doi: 10.1016/j.rec.2020.04.008 [published Online First: 2020/05/27]

172. Giner-Caro JA, Caballero L, Casas-Pina T, et al. High sensitive cardiac troponin T in the management of uncertain chest pain. *Int J Cardiol* 2013;168(4):4422-3. doi: 10.1016/j.ijcard.2013.05.015

173. Goodman DA, Kavsak PA, Hill SA, et al. Presenting characteristics of patients undergoing cardiac troponin measurements in the emergency department. *CJEM* 2015;17(1):62-6. doi: 10.2310/8000.2013.131298

174. Gratzel P. Neuer Algorithmus bei Infarktverdacht. *MMW Fortschritte der Medizin* 2015;157(16):9.

175. Graven T, Klykken B, Kleinau O, et al. Measurement of high-sensitivity troponin-I in suspected coronary related chest pain in Emergency Departments. *Tidsskr Nor Laegeforen* 2021;141:2021-12. doi: 10.4045/tidsskr.21.0037

176. Greenslade J, Cho E, Van Hise C, et al. Evaluating Rapid Rule-out of Acute Myocardial Infarction Using a High-Sensitivity Cardiac Troponin I Assay at Presentation. *Clin Chem* 2018;64(5):820-29. doi:

For peer review only

10.1373/clinchem.2017.283887

177. Grinstein J, Bonaca MP, Jarolim P, et al. Prognostic implications of low level cardiac troponin elevation using high-sensitivity cardiac troponin T. *Clin Cardiol* 2015;38(4):230-5. doi: 10.1002/clc.22379

178. Haaf P, Reichlin T, Twerenbold R, et al. Risk stratification in patients with acute chest pain using three high-sensitivity cardiac troponin assays. *Eur Heart J* 2014;35(6):365-75. doi: 10.1093/eurheartj/eht218

179. Haider DG, Klemenz T, Fiedler GM, et al. High sensitive cardiac troponin T: Testing the test. *Int J Cardiol* 2017;228:779-83. doi: 10.1016/j.ijcard.2016.10.043

180. Halim SA, Mulgund J, Chen AY, et al. Use of guidelines-recommended management and outcomes among women and men with low-level troponin elevation: insights from CRUSADE. *Circ Cardiovasc Qual Outcomes* 2009;2(3):199-206. doi: 10.1161/CIRCOUTCOMES.108.810127

181. Hall T, Hallén J, Agewall S, et al. Changes in diagnosing non-ST-segment elevation myocardial infarction after the introduction of a new high-sensitivity cardiac troponin T assay: a single-centre experience. *Clin Lab* 2012;58(9-10):1029-36.

182. Hochholzer W, Reichlin T, Twerenbold R, et al. Incremental value of high-sensitivity cardiac troponin T for risk prediction in patients with suspected acute myocardial infarction. *Clin Chem* 2011;57(9):1318-26. doi: 10.1373/clinchem.2011.162073

183. Hochholzer W, Valina CM, Stratz C, et al. High-sensitivity cardiac troponin for risk prediction in patients with and without coronary heart disease. *Int J Cardiol* 2014;176(2):444-9. doi: 10.1016/j.ijcard.2014.07.094

184. Hochholzer W, Neumann F. The New 2015 ESC Guidelines for the Management of Acute Coronary Syndromes in Patients Presenting Without Persistent ST-segment Elevation. *Dtsch Med Wochenschr* 2016;141(11):782-5. doi: 10.1055/s-0042-107115

185. Hoeller R, Rubini Gimenez M, Reichlin T, et al. Normal presenting levels of high-sensitivity troponin and myocardial infarction. *Heart* 2013;99(21):1567-72. doi: 10.1136/heartjnl-2013-303643

186. Hromádka M, Rajdl D, Trefil L, et al. Diagnostic sensitivity of high-sensitivity troponin T in acute myocardial infarction in patients with chest pain. *Cor et Vasa* 2012;54(7-8):e227-e31. doi: 10.1016/j.crvasa.2012.06.002

187. Huang H, Zhu S, Wang W, et al. Diagnosis of acute myocardial infarction in patients with renal insufficiency using high-sensitivity troponin T. *Clin Chem Lab Med* 2015;53(5):723-30. doi: 10.1515/cclm-2014-0715

188. Huang HL, Zhu S, Wang WQ, et al. Diagnosis of Acute Myocardial Infarction in Hemodialysis Patients With High-Sensitivity Cardiac Troponin T Assay. *Arch Pathol Lab Med* 2016;140(1):75-80. doi: 10.5858/arpa.2014-0580-OA

189. Ilva T, Lund J, Porela P, et al. Early markers of myocardial injury: cTnI is enough. *Clin Chim Acta* 2009;400(1-2):82-5. doi: 10.1016/j.cca.2008.10.005

190. Invernizzi L, Doka M, Cappellini F, et al. Effectiveness of highly sensitive troponin T assay for early diagnosis of acute myocardial infarction (AMI). *Biochimica clinica* 2013;37(1):36-39.

191. Işıksaçan N, Bıyık İ, Ertürk M, et al. Comparison of high sensitive and conventional troponin assays in diagnosis of acute myocardial infarction. *Turk J Biochem* 2017;42(1):77-85. doi: 10.1515/tjb-2016-0270

192. Iwasaki M, Yamazaki K, Ikeda N, et al. Point of care assessment of cardiac troponin T level in CKD patients with chest symptom. *Ren Fail* 2017;39(1):166-72. doi: 10.1080/0886022X.2016.1256311

For peer review only

193. Jairam S, Jones P, Samaraie L, et al. Clinical diagnosis and outcomes for Troponin T 'positive' patients assessed by a high sensitivity compared with a 4th generation assay. *Emerg Med Australas* 2011;23(4):490-501. doi: 10.1111/j.1742-6723.2011.01446.x

194. Johannessen TR, Vallersnes OM, Halvorsen S, et al. Pre-hospital One-Hour Troponin in a Low-Prevalence Population of Acute Coronary Syndrome: OUT-ACS study. *Open Heart* 2020;7(2) doi: 10.1136/openhrt-2020-001296

195. Karady J, Mayrhofer T, Ferencik M, et al. Discordance of High-Sensitivity Troponin Assays in Patients With Suspected Acute Coronary Syndromes. *J Am Coll Cardiol* 2021;77(12):1487-99. doi: 10.1016/j.jacc.2021.01.046

196. Karon BS, Wockenfus AM, Hartung KJ, et al. Comparing analytical outliers and the percent of emergency department patients with results above the 99th percentile upper reference limit for 2 conventional and one high sensitivity troponin assay. *Clin Biochem* 2018;53:104-09. doi: 10.1016/j.clinbiochem.2018.01.001

197. Kavsak PA, MacRae AR, Palomaki GE, et al. Health outcomes categorized by current and previous definitions of acute myocardial infarction in an unselected cohort of troponin-naive emergency department patients. *Clin Chem* 2006;52(11):2028-35. doi: 10.1373/clinchem.2006.073403

198. Kavsak PA, Wang X, Ko DT, et al. Short- and long-term risk stratification using a next-generation, high-sensitivity research cardiac troponin I (hs-cTnI) assay in an emergency department chest pain population. *Clin Chem* 2009;55(10):1809-15. doi: 10.1373/clinchem.2009.127241

199. Kavsak P, Hill S, Bhanich SW, et al. Biomarkers for Predicting Serious Cardiac Outcomes at 72 Hours in Patients Presenting Early after Chest Pain Onset with Symptoms of Acute Coronary Syndromes. *Clin Chem* 2012;58(1):298-302. doi: 10.1373/clinchem.2011.172064

200. Kavsak PA, Shortt C, Ma J, et al. A laboratory score at presentation to rule-out serious cardiac outcomes or death in patients presenting with symptoms suggestive of acute coronary syndrome. *Clin Chim Acta* 2017;469:69-74. doi: 10.1016/j.cca.2017.03.021

201. Kavsak P, Worster A, Shortt C, et al. High-sensitivity Cardiac Troponin Concentrations at Emergency Department Presentation in Females and Males With an Acute Cardiac Outcome. *Ann Clin Biochem* 2018;55(5):604-07. doi: 10.1177/0004563217743997

202. Kavsak PA, Worster A, Shortt C, et al. Performance of high-sensitivity cardiac troponin in the emergency department for myocardial infarction and a composite cardiac outcome across different estimated glomerular filtration rates. *Clin Chim Acta* 2018;479:166-70. doi: 10.1016/j.cca.2018.01.034

203. Kavsak PA, Cerasuolo JO, Ko DT, et al. Using the clinical chemistry score in the emergency department to detect adverse cardiac events: a diagnostic accuracy study. *CMAJ Open* 2020;8(4):E676-E84. doi: 10.9778/cmajo.20200047

204. Kelly AM. Performance of a sensitive troponin assay in the early diagnosis of acute myocardial infarction in the emergency department. *Emerg Med Australas* 2011;23(2):181-5. doi: 10.1111/j.1742-6723.2011.01388.x

205. Kelly A, Klim S. What is the 30-day rate of adverse cardiac events in chest pain patients with ED troponin I assays≤99th centile using a contemporary sensitive assay? An exploratory analysis. *Eur J Emerg Med* 2014;21(4):276-80. doi: 10.1097/MEJ.0000000000000079

206. Kelly AM, Klim S. Prospective external validation of an accelerated (2-h) acute coronary syndrome rule-out process using a contemporary troponin assay. *Int J Emerg Med* 2014;7:42. doi: 10.1186/s12245-014-0042-3

For peer review only

207. Kelly AM, Klim S. Does undetectable troponin I at presentation using a contemporary sensitive assay rule out myocardial infarction? A cohort study. *Emerg Med J* 2015;32(10):760-3. doi: 10.1136/emermed-2014-204442

208. Kemper DW, Semjonow V, de Theije F, et al. Analytical evaluation of a new point of care system for measuring cardiac Troponin I. *Clin Biochem* 2017;50(4-5):174-80. doi: 10.1016/j.clinbiochem.2016.11.011

209. Khan E, Lambrakis K, Blyth A, et al. Classification performance of clinical risk scoring in suspected acute coronary syndrome beyond a rule-out troponin profile. *Eur Heart J Acute Cardiovasc Care* 2021;10(9):1038-47. doi: 10.1093/ehjacc/zuab040

210. Khoshnood A, Erlandsson M, Isma N, et al. Diagnostic accuracy of troponin T measured >/=6h after symptom onset for ruling out myocardial infarction. *Scand Cardiovasc J* 2020;54(3):153-61. doi: 10.1080/14017431.2019.1699248

211. Kienbacher CL, Fuhrmann V, van Tulder R, et al. Impact of more conservative European Society of Cardiology guidelines on the management of patients with acute chest pain. *Int J Clin Pract* 2021;75(6):e14133. doi: 10.1111/ijcp.14133

212. Kim J, Gaddam S, Wu WC, et al. Stratified reporting of high sensitivity troponin I assay is associated with suboptimal management of patients with acute coronary syndrome and intermediate troponin elevation. *J Clin Lab Anal* 2013;27(5):402-6. doi: 10.1002/jcla.21618

213. Kimenai DM, Lindahl B, Jernberg T, et al. Sex-specific effects of implementing a high-sensitivity troponin I assay in patients with suspected acute coronary syndrome: results from SWEDEHEART registry. *Sci Rep* 2020;10(1):15227. doi: 10.1038/s41598-020-72204-2

214. Kitamura M, Hata N, Takayama T, et al. High-sensitivity cardiac troponin T for earlier diagnosis of acute myocardial infarction in patients with initially negative troponin T test--comparison between cardiac markers. *J Cardiol* 2013;62(6):336-42. doi: 10.1016/j.jjcc.2013.06.005

215. Kremneva L, Suplotov S, Shalaev S. Estimation of highly sensitive troponin tests in the diagnosis of acute coronary syndrome. *Ration Pharmacother Cardiol* 2016;12(2) doi: 10.20996/1819-6446-2016-12-2-204-209

216. Krintus M, Kozinski M, Boudry P, et al. European multicenter analytical evaluation of the Abbott ARCHITECT STAT high sensitive troponin I immunoassay. *Clin Chem Lab Med* 2014;52(11):1657-65. doi: 10.1515/cclm-2014-0107

217. Kumar A, Swamy R, Ramaswamy. Study on conventional and novel cardiac biomarkers in acute myocardial infarction. *Asian J Pharm Clin Res* 2016;9(2):327-31.

218. Lambrakis K, Papendick C, French JK, et al. Late Outcomes of the RAPID-TnT Randomized Controlled Trial: 0/1-Hour High-Sensitivity Troponin T Protocol in Suspected ACS. *Circulation* 2021;144(2):113-25. doi: 10.1161/CIRCULATIONAHA.121.055009

219. Lee H, Kerr D, O'H Ici D, et al. Clinical Significance of Initial Troponin I in the Grey Zone in Emergency Department Chest Pain Patients: A Retrospective Pilot Study. *Emerg Med J* 2010;27(4):302-4. doi: 10.1136/emj.2009.077669

220. Lehmacher J, Neumann JT, Sorensen NA, et al. Predictive Value of Serial ECGs in Patients with Suspected Myocardial Infarction. *J Clin Med* 2020;9(7) doi: 10.3390/jcm9072303

221. Li WJ, Chen XM, Nie XY, et al. Early diagnostic and prognostic utility of high-sensitive troponin assays in acute myocardial infarction: a meta-analysis. *Intern Med J* 2015;45(7):748-56. doi: 10.1111/imj.12642

222. Lim KD, Yan AT, Casanova A, et al. Quantitative troponin elevation does not provide incremental prognostic value

For peer review only

beyond comprehensive risk stratification in patients with non-ST-segment elevation acute coronary syndromes. *Am Heart J* 2008;155(4):718-24. doi: 10.1016/j.ahj.2007.11.012

223. Lin S, Yokoyama H, Rac VE, et al. Novel biomarkers in diagnosing cardiac ischemia in the emergency department: a systematic review. *Resuscitation* 2012;83(6):684-91. doi: 10.1016/j.resuscitation.2011.12.015

224. Lindahl B, Venge P, James S. The new high-sensitivity cardiac troponin T assay improves risk assessment in acute coronary syndromes. *Am Heart J* 2010;160(2):224-9. doi: 10.1016/j.ahj.2010.05.023

225. Love SA, Sandoval Y, Smith SW, et al. Incidence of Undetectable, Measurable, and Increased Cardiac Troponin I Concentrations Above the 99th Percentile Using a High-Sensitivity vs a Contemporary Assay in Patients Presenting to the Emergency Department. *Clin Chem* 2016;62(8):1115-9. doi: 10.1373/clinchem.2016.256305

226. Lynn JS, Singh A, Snoey ER. Can We Exclude the Diagnosis of Non-ST Segment Myocardial Infarction on the Basis of a Single Troponin I and a Symptom Duration >/=8 Hours? *ISRN Cardiol* 2011;2011:364728. doi: 10.5402/2011/364728

227. Maag R, Sun S, Hannon M, et al. Positive predictive value of an elevated cardiac troponin for type I myocardial infarction in ED patients based on the chief complaint. *Am J Emerg Med* 2015;33(4):516-20. doi: 10.1016/j.ajem.2015.01.003

228. Macrae AR, Kavsak PA, Lustig V, et al. Assessing the requirement for the 6-hour interval between specimens in the American Heart Association Classification of Myocardial Infarction in Epidemiology and Clinical Research Studies. *Clin Chem* 2006;52(5):812-8. doi: 10.1373/clinchem.2005.059550

229. Madsen T, Stewart M, Smyres C, et al. Significance of an Indeterminate Troponin I in Patients Evaluated for Chest Pain in an Emergency Department Observation Unit. *Crit Pathw Cardiol* 2015;14(4):146-9. doi: 10.1097/HPC.0000000000000054

230. Mahler S, Miller C, Hollander J, et al. Identifying patients for early discharge: performance of decision rules among patients with acute chest pain. *Int J Cardiol* 2013;168(2):795-802. doi: 10.1016/j.ijcard.2012.10.010

231. Marjot J, Kaier TE, Henderson K, et al. A single centre prospective cohort study addressing the effect of a rule-in/rule-out troponin algorithm on routine clinical practice. *Eur Heart J Acute Cardiovasc Care* 2019;8(5):404-11. doi: 10.1177/2048872617746850

232. Markota A, Bernhardt M, Palfy M. Comparison of point-of-care and laboratory troponin I assays. *Zdravniški vestnik2011* 2011;80(12):905-8.

233. Masotti S, Prontera C, Musetti V, et al. Evaluation of analytical performance of a new high-sensitivity immunoassay for cardiac troponin I. *Clin Chem Lab Med* 2018;56(3):492-501. doi: 10.1515/cclm-2017-0387

234. Bohula May EA, Bonaca MP, Jarolim P, et al. Prognostic performance of a high-sensitivity cardiac troponin I assay in patients with non-ST-elevation acute coronary syndrome. *Clin Chem* 2014;60(1):158-64. doi: 10.1373/clinchem.2013.206441

235. McRae A, Andruchow J. Highly-sensitive troponin T algorithm facilitates early discharge of low-risk chest pain patients within 1 h of emergency department arrival. *Evid Based Med* 2015;20(4):144. doi: 10.1136/ebmed-2015-110224

236. Melki D, Lind S, Agewall S, et al. Diagnostic value of high sensitive troponin T in chest pain patients with no persistent ST-elevations. *Scand Cardiovasc J* 2011;45(4):198-204. doi: 10.3109/14017431.2011.565792

For peer review only

237. Melki D, Lind S, Agewall S, et al. Prognostic value of combining high sensitive troponin T and N-terminal pro B-type natriuretic peptide in chest pain patients with no persistent ST-elevation. *Clin Chim Acta* 2012;413(9-10):933-7. doi: 10.1016/j.cca.2012.02.008

238. Melki D, Lugnegard J, Alfredsson J, et al. Implications of Introducing High-Sensitivity Cardiac Troponin T Into Clinical Practice: Data From the SWEDEHEART Registry. *J Am Coll Cardiol* 2015;65(16):1655-64. doi: 10.1016/j.jacc.2015.02.044

239. Meune C, Balmelli C, Twerenbold R, et al. Patients with acute coronary syndrome and normal high-sensitivity troponin. *Am J Med* 2011;124(12):1151-7. doi: 10.1016/j.amjmed.2011.07.032

240. Meune C, Reichlin T, Irfan A, et al. How safe is the outpatient management of patients with acute chest pain and mildly increased cardiac troponin concentrations? *Clin Chem* 2012;58(5):916-24. doi: 10.1373/clinchem.2011.178053

241. Miller C, Fermann G, Lindsell C, et al. Initial risk stratification and presenting characteristics of patients with evolving myocardial infarctions. *Emerg Med J* 2008;25(8):492-7. doi: 10.1136/emj.2007.052183

242. Mills N, Churchhouse A, Lee K, et al. Implementation of a sensitive troponin I assay and risk of recurrent myocardial infarction and death in patients with suspected acute coronary syndrome. *JAMA* 2011;305(12):1210-6. doi: 10.1001/jama.2011.338

243. Mills NL, Lee KK, McAllister DA, et al. Implications of lowering threshold of plasma troponin concentration in diagnosis of myocardial infarction: cohort study. *BMJ* 2012;344:e1533. doi: 10.1136/bmj.e1533

244. Le Moal E, Giuliani I, Bertinchant JP, et al. Earlier detection of myocardial infarction by an improved cardiac TnI assay. *Clin Biochem* 2007;40(13-14):1065-73. doi: 10.1016/j.clinbiochem.2007.05.004

245. Mohsen M, Shawky A. The diagnostic utility of High-Sensitivity Cardiac Troponin T in acute coronary syndrome. *The Egyptian Heart Journal* 2016;68(1):1-9. doi: 10.1016/j.ehj.2014.12.003

246. Mokhtari A, Lindahl B, Smith J, et al. Diagnostic Accuracy of High-Sensitivity Cardiac Troponin T at Presentation Combined With History and ECG for Ruling Out Major Adverse Cardiac Events. *Ann Emerg Med* 2016;68(6):649-58.e3. doi: 10.1016/j.annemergmed.2016.06.008

247. Velilla Moliner J, Gros Bañeres B, Povar Marco J, et al. Diagnostic performance of high sensitivity troponin in non-ST elevation acute coronary syndrome. *Medicina Intensiva (English Edition)* 2020;44(2):88-95. doi: 10.1016/j.medine.2019.11.001

248. Mueller M, Celik S, Biener M, et al. Diagnostic and prognostic performance of a novel high-sensitivity cardiac troponin T assay compared to a contemporary sensitive cardiac troponin I assay in patients with acute coronary syndrome. *Clin Res Cardiol* 2012;101(10):837-45. doi: 10.1007/s00392-012-0469-6

249. Mueller M, Biener M, Vafaie M, et al. Absolute and relative kinetic changes of high-sensitivity cardiac troponin T in acute coronary syndrome and in patients with increased troponin in the absence of acute coronary syndrome. *Clin Chem* 2012;58(1):209-18. doi: 10.1373/clinchem.2011.171827

250. Mueler C. Use of high-sensitivity troponin for the diagnosis of acute myocardial infarction. *Coron Artery Dis* 2013;24(8):710-2. doi: 10.1097/MCA.0000000000000049

251. Mueller M, Biener M, Vafaie M, et al. Prognostic performance of kinetic changes of high-sensitivity troponin T in acute coronary syndrome and in patients with increased troponin without acute coronary syndrome. *Int J Cardiol*

2014;174(3):524-9. doi: 10.1016/j.ijcard.2014.04.110

252. Mueller-Hennessen M, Mueller C, Giannitsis E, et al. Serial Sampling of High-Sensitivity Cardiac Troponin T May Not Be Required for Prediction of Acute Myocardial Infarction Diagnosis in Chest Pain Patients with Highly Abnormal Concentrations at Presentation. *Clin Chem* 2017;63(2):542-51. doi: 10.1373/clinchem.2016.258392

253. Mungai E, Hamilton B, Burns D. Comparison of High-Sensitivity Troponin T Assay to Conventional Troponin T Assay for Rule Out of Acute Coronary Syndrome in the Emergency Department. *Adv Emerg Nurs J* 2020;42(4):304-14.

254. Nacke L, Blanchet J, Reyes G, et al. Effectiveness of different cutoff points of high-sensitivity troponin T to diagnose myocardial infarction. *Revista de la Federacion Argentina de Cardiologia* 2014;43(3):141-45.

255. Nadarajah R, Gale C. The management of acute coronary syndromes in patients presenting without persistent ST-segment elevation: key points from the ESC 2020 Clinical Practice Guidelines for the general and emergency physician. *Clin Med (Lond)* 2021;21(2):e206-e11. doi: 10.7861/clinmed.2020-0879

256. Narain S, Gupta N, Sethi R, et al. Clinical Correlation of Multiple Biomarkers for Risk Assessment in Patients With Acute Coronary Syndrome. *Indian Heart J* 2008;60(6):536-42.

257. Nejatian A, Omstedt A, Hoijer J, et al. Outcomes in Patients With Chest Pain Discharged After Evaluation Using a High-Sensitivity Troponin T Assay. *J Am Coll Cardiol* 2017;69(21):2622-30. doi: 10.1016/j.jacc.2017.03.586

258. Nestelberger T, Wildi K, Boeddinghaus J, et al. Characterization of the observe zone of the ESC 2015 high-sensitivity cardiac troponin 0h/1h-algorithm for the early diagnosis of acute myocardial infarction. *Int J Cardiol* 2016;207:238-45. doi: 10.1016/j.ijcard.2016.01.112

259. Nestelberger T, Boeddinghaus J, Lopez-Ayala P, et al. Cardiovascular Biomarkers in the Early Discrimination of Type 2 Myocardial Infarction. *JAMA Cardiol* 2021;6(7):771-80. doi: 10.1001/jamacardio.2021.0669

260. Nestelberger T, Lopez-Ayala P, Boeddinghaus J, et al. External Validation and Extension of a Clinical Score for the Discrimination of Type 2 Myocardial Infarction. *J Clin Med* 2021;10(6) doi: 10.3390/jcm10061264

261. Neumann JT, Sorensen NA, Ojeda F, et al. Immediate Rule-Out of Acute Myocardial Infarction Using Electrocardiogram and Baseline High-Sensitivity Troponin I. *Clin Chem* 2017;63(1):394-402. doi: 10.1373/clinchem.2016.262659

262. Nilsson T, Johannesson E, Lundager Forberg J, et al. Diagnostic accuracy of the HEART Pathway and EDACS-ADP when combined with a 0-hour/1-hour hs-cTnT protocol for assessment of acute chest pain patients. *Emerg Med J* 2021;38(11):808-13. doi: 10.1136/emermed-2020-210833

263. Normann J, Mueller M, Biener M, et al. Effect of older age on diagnostic and prognostic performance of high-sensitivity troponin T in patients presenting to an emergency department. *Am Heart J* 2012;164(5):698-705 e4. doi: 10.1016/j.ahj.2012.08.003

264. Nowak RM, Jacobsen G, Limkakeng A, Jr., et al. Outpatient versus observation/inpatient management of emergency department patients rapidly ruled-out for acute myocardial infarction: Findings from the HIGH-US study. *Am Heart J* 2021;231:6-17. doi: 10.1016/j.ahj.2020.10.067

265. Olivieri F, Galeazzi R, Giavarina D, et al. Aged-related increase of high sensitive Troponin T and its implication in acute myocardial infarction diagnosis of elderly patients. *Mech Ageing Dev* 2012;133(5):300-5. doi: 10.1016/j.mad.2012.03.005

For peer review only

266. Omland T. New Sensitive Cardiac Troponin Assays for the Early Diagnosis of Myocardial Infarction. *Drugs Today (Barc)* 2011;47(4):303-12. doi: 10.1358/dot.2011.47.4.1533082

267. Osredkar J, Krivic K, Fabjan T, et al. Point-of-care high-sensitivity assay on PATHFAST as the backup in the emergency room. *Medicine Access @ Point of Care* 2021;5 doi: 10.1177/23992026211055095

268. Palamalai V, Murakami MM, Apple FS. Diagnostic performance of four point of care cardiac troponin I assays to rule in and rule out acute myocardial infarction. *Clin Biochem* 2013;46(16-17):1631-5. doi: 10.1016/j.clinbiochem.2013.06.026

269. Paoloni R, Kumar P, Janu M. Pilot study of high-sensitivity troponin T testing to facilitate safe early disposition decisions in patients presenting to the emergency department with chest pain. *Intern Med J* 2010;40(3):188-92. doi: 10.1111/j.1445-5994.2009.01962.x

270. Papendick C, Blyth A, Seshadri A, et al. A randomized trial of a 1-hour troponin T protocol in suspected acute coronary syndromes: Design of the Rapid Assessment of Possible ACS In the emergency Department with high sensitivity Troponin T (RAPID-TnT) study. *Am Heart J* 2017;190:25-33. doi: 10.1016/j.ahj.2017.05.004

271. Parikh RH, Seliger SL, de Lemos J, et al. Prognostic Significance of High-Sensitivity Cardiac Troponin T Concentrations between the Limit of Blank and Limit of Detection in Community-Dwelling Adults: A Metaanalysis. *Clin Chem* 2015;61(12):1524-31. doi: 10.1373/clinchem.2015.244160

272. Patsias I, Swanson B, Hudson M, et al. he (Dis) Utility of a Change in Troponin I for Diagnosis of Non-ST-Segment Elevation Myocardial Infarction in an Observation Unit. *Crit Pathw Cardiol* 2017;16(3):105-08. doi: 10.1097/HPC.0000000000000120

273. Peck D, Knott J, Lefkovits J. Clinical impact of a high-sensitivity troponin assay introduction on patients presenting to the emergency department. *Emerg Med Australas* 2016;28(3):273-8. doi: 10.1111/1742-6723.12566

274. Pickering JW, Flaws D, Smith SW, et al. A Risk Assessment Score and Initial High-sensitivity Troponin Combine to Identify Low Risk of Acute Myocardial Infarction in the Emergency Department. *Acad Emerg Med* 2018;25(4):434-43. doi: 10.1111/acem.13343

275. Pickering JW, Young JM, George PM, et al. Early kinetic profiles of troponin I and T measured by high-sensitivity assays in patients with myocardial infarction. *Clin Chim Acta* 2020;505:15-25. doi: 10.1016/j.cca.2020.02.009

276. Puelacher C, Gugala M, Adamson PD, et al. Incidence and outcomes of unstable angina compared with non-ST-elevation myocardial infarction. *Heart* 2019;105(18):1423-31. doi: 10.1136/heartjnl-2018-314305

277. Rao M, Panduranga P, Al-Mukhaini M, et al. Predictive value of a 4-hour accelerated diagnostic protocol in patients with suspected ischemic chest pain presenting to an emergency department. *Oman Med J* 2012;27(3):207-11. doi: 10.5001/omj.2012.47

278. Reichlin T, Hochholzer W, Bassetti S, et al. Early diagnosis of myocardial infarction with sensitive cardiac troponin assays. *N Engl J Med* 2009;361(9):858-67. doi: 10.1056/NEJMoa0900428

279. Reichlin T, Twerenbold R, Maushart C, et al. Risk stratification in patients with unstable angina using absolute serial changes of 3 high-sensitive troponin assays. *Am Heart J* 2013;165(3):371-8 e3. doi: 10.1016/j.ahj.2012.11.010

280. Reiter M, Reichlin T, Twerenbold R, et al. Diagnosis of acute myocardial infarction using highly sensitive cardiac troponin assays. *Eur Cardiol* 2011;7(1):18-20. doi: 10.15420/ecr.2011.7.1.18

281. Ren D, Huang T, Liu X, et al. High-sensitive cardiac troponin for the diagnosis of acute myocardial infarction in

different chronic kidney disease stages. *BMC Cardiovasc Disord* 2021;21(1):100. doi: 10.1186/s12872-020-01746-0

282. Renaud B, Maison P, Ngako A, et al. Impact of point-of-care testing in the emergency department evaluation and treatment of patients with suspected acute coronary syndromes. *Acad Emerg Med* 2008;15(3):216-24. doi: 10.1111/j.1553-2712.2008.00069.x
283. Roberts A. Biomarkers: hs-cTnT measurement to rule out MI. *Nat Rev Cardiol* 2014;11(6):311. doi: 10.1038/nrcardio.2014.52
284. Roos A, Bandstein N, Lundback M, et al. Stable High-Sensitivity Cardiac Troponin T Levels and Outcomes in Patients With Chest Pain. *J Am Coll Cardiol* 2017;70(18):2226-36. doi: 10.1016/j.jacc.2017.08.064
285. Roos A, Hellgren A, Rafatnia F, et al. Investigations, findings, and follow-up in patients with chest pain and elevated high-sensitivity cardiac troponin T levels but no myocardial infarction. *Int J Cardiol* 2017;232:111-16. doi: 10.1016/j.ijcard.2017.01.044
286. Röttger E, de Vries-Spithoven S, Reitsma J, et al. Safety of a 1-hour Rule-out High-sensitive Troponin T Protocol in Patients With Chest Pain at the Emergency Department. *Crit Pathw Cardiol* 2017;16(4):129-34. doi: 10.1097/HPC.0000000000000135
287. Rubini Gimenez M, Hoeller R, Reichlin T, et al. Rapid rule out of acute myocardial infarction using undetectable levels of high-sensitivity cardiac troponin. *Int J Cardiol* 2013;168(4):3896-901. doi: 10.1016/j.ijcard.2013.06.049
288. Rudolph V, Keller T, Schulz A, et al. Diagnostic and prognostic performance of myeloperoxidase plasma levels compared with sensitive troponins in patients admitted with acute onset chest pain. *Circ Cardiovasc Genet* 2012;5(5):561-8. doi: 10.1161/CIRCGENETICS.111.962290
289. Saad YM, McEwan J, Shugman IM, et al. Use of a high-sensitivity troponin T assay in the assessment and disposition of patients attending a tertiary Australian emergency department: a cross-sectional pilot study. *Emerg Med Australas* 2015;27(5):405-11. doi: 10.1111/1742-6723.12430
290. Saenger A, Korpi-Steiner N, Bryant S, et al. Utilization of a High Sensitive Troponin T Assay Optimizes Serial Sampling in the Diagnosis of Acute Myocardial Infarction Compared to Multiple Contemporary Troponin Assays. *Circ* 2010;122(21):A21588.
291. Saenger AK, Beyrau R, Braun S, et al. Multicenter analytical evaluation of a high-sensitivity troponin T assay. *Clin Chim Acta* 2011;412(9-10):748-54. doi: 10.1016/j.cca.2010.12.034
292. Sanchis J, Bardaji A, Bosch X, et al. Usefulness of high-sensitivity troponin T for the evaluation of patients with acute chest pain and no or minimal myocardial damage. *Am Heart J* 2012;164(2):194-200 e1. doi: 10.1016/j.ahj.2012.05.015
293. Sanchis J, Garcia-Blas S, Mainar L, et al. High-sensitivity versus conventional troponin for management and prognosis assessment of patients with acute chest pain. *Heart* 2014;100(20):1591-6. doi: 10.1136/heartjnl-2013-305440
294. Sanchis J, Abellan L, Garcia-Blas S, et al. Usefulness of delta troponin for diagnosis and prognosis assessment of non-ST-segment elevation acute chest pain. *Eur Heart J Acute Cardiovasc Care* 2016;5(5):399-406. doi: 10.1177/2048872615593534
295. Sandoval Y, Smith SW, Schulz KM, et al. Diagnosis of type 1 and type 2 myocardial infarction using a high-sensitivity cardiac troponin I assay with sex-specific 99th percentiles based on the third universal definition of myocardial

For peer review only

infarction classification system. *Clin Chem* 2015;61(4):657-63. doi: 10.1373/clinchem.2014.236638

296. Sandoval Y, Smith SW, Love SA, et al. Single High-Sensitivity Cardiac Troponin I to Rule Out Acute Myocardial Infarction. *Am J Med* 2017;130(9):1076-83 e1. doi: 10.1016/j.amjmed.2017.02.032
297. Sandoval Y, Smith SW, Shah AS, et al. Rapid Rule-Out of Acute Myocardial Injury Using a Single High-Sensitivity Cardiac Troponin I Measurement. *Clin Chem* 2017;63(1):369-76. doi: 10.1373/clinchem.2016.264523
298. Santalo M, Martin A, Velilla J, et al. Using high-sensitivity troponin T: the importance of the proper gold standard. *Am J Med* 2013;126(8):709-17. doi: 10.1016/j.amjmed.2013.03.003
299. Schonemann-Lund M, Schoos MM, Iversen K, et al. Retrospective Evaluation of Two Fast-track Strategies to Rule Out Acute Coronary Syndrome in a Real-life Chest Pain Population. *J Emerg Med* 2015;49(6):833-42. doi: 10.1016/j.jemermed.2015.06.026
300. Shah ASV, Anand A, Sandoval Y, et al. High-sensitivity cardiac troponin I at presentation in patients with suspected acute coronary syndrome: a cohort study. *The Lancet* 2015;386(10012):2481-88. doi: 10.1016/s0140-6736(15)00391-8
301. Shah AS, Griffiths M, Lee KK, et al. High sensitivity cardiac troponin and the under-diagnosis of myocardial infarction in women: prospective cohort study. *BMJ* 2015;350:g7873. doi: 10.1136/bmj.g7873
302. Shin YS, Ahn S, Kim YJ, et al. External validation of the emergency department assessment of chest pain score accelerated diagnostic pathway (EDACS-ADP). *Am J Emerg Med* 2020;38(11):2264-70. doi: 10.1016/j.ajem.2019.09.019
303. Shortt C, Phan K, Hill SA, et al. An approach to rule-out an acute cardiovascular event or death in emergency department patients using outcome-based cutoffs for high-sensitivity cardiac troponin assays and glucose. *Clin Biochem* 2015;48(4-5):282-7. doi: 10.1016/j.clinbiochem.2014.11.010
304. Shortt C, Ma J, Clayton N, et al. Rule-In and Rule-Out of Myocardial Infarction Using Cardiac Troponin and Glycemic Biomarkers in Patients with Symptoms Suggestive of Acute Coronary Syndrome. *Clin Chem* 2017;63(1):403-14. doi: 10.1373/clinchem.2016.261545
305. Simpson AJ, Potter JM, Koerbin G, et al. Use of observed within-person variation of cardiac troponin in emergency department patients for determination of biological variation and percentage and absolute reference change values. *Clin Chem* 2014;60(6):848-54. doi: 10.1373/clinchem.2013.219410
306. Singer AJ, Than MP, Smith S, et al. Missed myocardial infarctions in ED patients prospectively categorized as low risk by established risk scores. *Am J Emerg Med* 2017;35(5):704-09. doi: 10.1016/j.ajem.2017.01.003
307. Slagman A, von Recum J, Mockel M, et al. Diagnostic performance of a high-sensitive troponin T assay and a troponin T point of care assay in the clinical routine of an Emergency Department: A clinical cohort study. *Int J Cardiol* 2017;230:454-60. doi: 10.1016/j.ijcard.2016.12.085
308. Sorensen NA, Neumann JT, Ojeda F, et al. Challenging the 99th percentile: A lower troponin cutoff leads to low mortality of chest pain patients. *Int J Cardiol* 2017;232:289-93. doi: 10.1016/j.ijcard.2016.12.167
309. Sorensen NA, Ludwig S, Makarova N, et al. Prognostic Value of a Novel and Established High-Sensitivity Troponin I Assay in Patients Presenting with Suspected Myocardial Infarction. *Biomolecules* 2019;9(9) doi: 10.3390/biom9090469
310. Stengaard C, Sorensen JT, Ladefoged SA, et al. Quantitative point-of-care troponin T measurement for diagnosis and

For peer review only

prognosis in patients with a suspected acute myocardial infarction. *Am J Cardiol* 2013;112(9):1361-6. doi: 10.1016/j.amjcard.2013.06.026

311. Stopyra JP, Miller CD, Hiestand BC, et al. Chest Pain Risk Stratification: A Comparison of the 2-Hour Accelerated Diagnostic Protocol (ADAPT) and the HEART Pathway. *Crit Pathw Cardiol* 2016;15(2):46-9. doi: 10.1097/HPC.0000000000000072
312. Stopyra J, Snavely AC, Hiestand B, et al. Comparison of accelerated diagnostic pathways for acute chest pain risk stratification. *Heart* 2020;106(13):977-84. doi: 10.1136/heartjnl-2019-316426
313. Stoyanov KM, Biener M, Hund H, et al. Effects of crowding in the emergency department on the diagnosis and management of suspected acute coronary syndrome using rapid algorithms: an observational study. *BMJ Open* 2020;10(10):e041757. doi: 10.1136/bmjopen-2020-041757
314. Storrow AB, Christenson RH, Nowak RM, et al. Diagnostic performance of cardiac Troponin I for early rule-in and rule-out of acute myocardial infarction: Results of a prospective multicenter trial. *Clin Biochem* 2015;48(4-5):254-9. doi: 10.1016/j.clinbiochem.2014.08.018
315. Storrow AB, Nowak RM, Diercks DB, et al. Absolute and relative changes (delta) in troponin I for early diagnosis of myocardial infarction: Results of a prospective multicenter trial. *Clin Biochem* 2015;48(4-5):260-7. doi: 10.1016/j.clinbiochem.2014.09.012
316. Straface AL, Myers JH, Kirchick HJ, et al. A rapid point-of-care cardiac marker testing strategy facilitates the rapid diagnosis and management of chest pain patients in the emergency department. *Am J Clin Pathol* 2008;129(5):788-95. doi: 10.1309/9GGNMURLJWJD88W3
317. Su Q, Guo Y, Liu H, et al. Diagnostic Role of High-Sensitivity Cardiac Troponin T in Acute Myocardial Infarction and Cardiac Noncoronary Artery Disease. *Arch Med Res* 2015;46(3):193-8. doi: 10.1016/j.arcmed.2015.03.005
318. Suh D, Keller DI, Hof D, et al. Rule-out of non-ST elevation myocardial infarction by five point of care cardiac troponin assays according to the 0 h/3 h algorithm of the European Society of Cardiology. *Clin Chem Lab Med* 2018;56(4):649-57. doi: 10.1515/cclm-2017-0486
319. Suzuki K, Komukai K, Nakata K, et al. The Usefulness and Limitations of Point-of-care Cardiac Troponin Measurement in the Emergency Department. *Intern Med* 2018;57(12):1673-80. doi: 10.2169/internalmedicine.0098-17
320. Takakuwa KM, Ou F-S, Peterson ED, et al. The Usage Patterns of Cardiac Bedside Markers Employing Point-of-Care Testing for Troponin in Non-ST-Segment Elevation Acute Coronary Syndrome: Results from CRUSADE. *Clinical Cardiology* 2009;32(9):498-505. doi: 10.1002/clc.20626
321. Tecson K, Arnold W, Barrett T, et al. Interpretation of positive troponin results among patients with and without myocardial infarction. *Proc (Bayl Univ Med Cent)* 2017;30(1):11-15. doi: 10.1080/08998280.2017.11929513
322. Than M, Cullen L, Reid CM, et al. A 2-h diagnostic protocol to assess patients with chest pain symptoms in the Asia-Pacific region (ASPECT): a prospective observational validation study. *The Lancet* 2011;377(9771):1077-84. doi: 10.1016/s0140-6736(11)60310-3
323. Than M, Cullen L, Aldous S, et al. 2-Hour Accelerated Diagnostic Protocol to Assess Patients With Chest Pain Symptoms Using Contemporary Troponins as the Only Biomarker. *Journal of the American College of Cardiology* 2012;59(23):2091-8. doi: 10.1016/j.jacc.2012.02.035

For peer review only

324. Than M, Aldous S, Lord SJ, et al. A 2-hour diagnostic protocol for possible cardiac chest pain in the emergency department: a randomized clinical trial. *JAMA Intern Med* 2014;174(1):51-8. doi: 10.1001/jamainternmed.2013.11362

325. Thelin J, Melander O, Ohlin B. Early rule-out of acute coronary syndrome using undetectable levels of high sensitivity troponin T. *Eur Heart J Acute Cardiovasc Care* 2015;4(5):403-9. doi: 10.1177/2048872614554107

326. Truong QA, Bayley J, Hoffmann U, et al. Multi-marker strategy of natriuretic peptide with either conventional or high-sensitivity troponin-T for acute coronary syndrome diagnosis in emergency department patients with chest pain: from the "Rule Out Myocardial Infarction using Computer Assisted Tomography" (ROMICAT) trial. *Am Heart J* 2012;163(6):972-79 e1. doi: 10.1016/j.ahj.2012.03.010

327. Twerenbold R, Wildi K, Jaeger C, et al. Optimal Cutoff Levels of More Sensitive Cardiac Troponin Assays for the Early Diagnosis of Myocardial Infarction in Patients With Renal Dysfunction. *Circulation* 2015;131(23):2041-50. doi: 10.1161/CIRCULATIONAHA.114.014245

328. Vafaie M, Biener M, Mueller M, et al. Analytically false or true positive elevations of high sensitivity cardiac troponin: a systematic approach. *Heart* 2014;100(6):508-14. doi: 10.1136/heartjnl-2012-303202

329. van der Laarse A, Cobbaert CM, Gorgels AP, et al. Will future troponin measurement overrule the ECG as the primary diagnostic tool in patients with acute coronary syndrome? *J Electrocardiol* 2013;46(4):312-7. doi: 10.1016/j.jelectrocard.2013.02.007

330. Van Hise CB, Greenslade JH, Parsonage W, et al. External validation of heart-type fatty acid binding protein, high-sensitivity cardiac troponin, and electrocardiography as rule-out for acute myocardial infarction. *Clin Biochem* 2018;52:161-63. doi: 10.1016/j.clinbiochem.2017.10.001

331. Vasile VC, Jaffe AS. High-Sensitivity Cardiac Troponin for the Diagnosis of Patients with Acute Coronary Syndromes. *Curr Cardiol Rep* 2017;19(10):92. doi: 10.1007/s11886-017-0904-4

332. Vasudevan A, Singer AJ, DeFilippi C, et al. Renal Function and Scaled Troponin in Patients Presenting to the Emergency Department with Symptoms of Myocardial Infarction. *Am J Nephrol* 2017;45(4):304-09. doi: 10.1159/000458451

333. Venge P, Johnston N, Lindahl B, et al. Normal plasma levels of cardiac troponin I measured by the high-sensitivity cardiac troponin I access prototype assay and the impact on the diagnosis of myocardial ischemia. *J Am Coll Cardiol* 2009;54(13):1165-72. doi: 10.1016/j.jacc.2009.05.051

334. Venge P, James S, Jansson L, et al. Clinical performance of two highly sensitive cardiac troponin I assays. *Clin Chem* 2009;55(1):109-16. doi: 10.1373/clinchem.2008.106500

335. Wang W, Huang H, Zhu S, et al. High-Sensitivity Cardiac Troponin T in Patients with Acute Myocardial Infarction in Acute Exacerbation of Chronic Obstructive Pulmonary Disease. *Clin lab* 2015;61(8):1083-93.

336. Wang JA, Qin Y, Lv J, et al. Clinical application of high-sensitivity cardiac troponin T test in acute myocardial infarction diagnosis. *Genet Mol Res* 2015;14(4):17959-65. doi: 10.4238/2015.December.22.21

337. Wassef AW, Hiebert B, Saeed MF, et al. Novel high-sensitivity troponin assay requires higher cut-off value to separate acute myocardial infarction from non-acute myocardial infarction in a high-risk population. *Can J Physiol Pharmacol* 2015;93(10):873-7. doi: 10.1139/cjpp-2014-0473

338. Wassie M, Lee MS, Sun BC, et al. Single vs Serial Measurements of Cardiac Troponin Level in the Evaluation of

For peer review only

Patients in the Emergency Department With Suspected Acute Myocardial Infarction. *JAMA Netw Open* 2021;4(2):e2037930. doi: 10.1001/jamanetworkopen.2020.37930

339. Weber M, Bazzino O, Navarro Estrada JL, et al. Improved diagnostic and prognostic performance of a new high-sensitive troponin T assay in patients with acute coronary syndrome. *Am Heart J* 2011;162(1):81-8. doi: 10.1016/j.ahj.2011.04.007
340. Wereski R, Kimenai DM, Taggart C, et al. Cardiac Troponin Thresholds and Kinetics to Differentiate Myocardial Injury and Myocardial Infarction. *Circulation* 2021;144(7):528-38. doi: 10.1161/CIRCULATIONAHA.121.054302
341. Westwood M, van Asselt T, Ramaekers B, et al. High-sensitivity troponin assays for the early rule-out or diagnosis of acute myocardial infarction in people with acute chest pain: a systematic review and cost-effectiveness analysis. *Health Technol Assess* 2015;19(44):1-234. doi: 10.3310/hta19440
342. Wildi K, Reichlin T, Twerenbold R, et al. Serial changes in high-sensitivity cardiac troponin I in the early diagnosis of acute myocardial infarction. *Int J Cardiol* 2013;168(4):4103-10. doi: 10.1016/j.ijcard.2013.07.078
343. Willeit P, Welsh P, Evans JDW, et al. High-Sensitivity Cardiac Troponin Concentration and Risk of First-Ever Cardiovascular Outcomes in 154,052 Participants. *J Am Coll Cardiol* 2017;70(5):558-68. doi: 10.1016/j.jacc.2017.05.062
344. Wolf S, Kaur R, McKeown W, et al. Noise Versus Signal: The Clinical Implications of an Increasingly Sensitive Troponin Assay for Patients With Suspected Acute Coronary Syndrome. *Crit Pathw Cardiol* 2014;13(3):89-95. doi: 10.1097/HPC.0000000000000020
345. Wong P, Rao G, Innasimuthu A, et al. Validation of a prediction score model to distinguish acute coronary syndromes from other conditions causing raised cardiac troponin T levels. *Coron Artery Dis* 2010;21(6):363-8. doi: 10.1097/MCA.0b013e32833d18d8
346. Yang HS, Shemesh A, Li J, et al. No increase in the incidence of cardiac troponin I concentration above the 99th percentile by Siemens Centaur high-sensitivity compared to the contemporary assay. *Clin Biochem* 2021;89:77-80. doi: 10.1016/j.clinbiochem.2020.12.001
347. Yean KS, Abd. Wahab MB, Zakaria MIB. A study on modified accelerated diagnostic protocol to safely discharge low-risk chest pain patients in emergency department. *Hong Kong Journal of Emergency Medicine* 2019;27(3):134-45. doi: 10.1177/1024907918820750
348. Yokoyama H, Higuma T, Endo T, et al. "30-minute-delta" of high-sensitivity troponin I improves diagnostic performance in acute myocardial infarction. *J Cardiol* 2018;71(2):144-48. doi: 10.1016/j.jjcc.2017.08.003
349. Zhao Y, Sivaswamy A, Lee MK, et al. A feasibility study for CODE-MI: High-sensitivity cardiac troponin-Optimizing the diagnosis of acute myocardial infarction/injury in women. *Am Heart J* 2021;234:60-70. doi: 10.1016/j.ahj.2021.01.008
350. Advantageous Predictors of Acute Coronary Syndromes Evaluation (APACE) Study (APACE) [Available from: https://www.clinicaltrials.gov/ct2/show/NCT00470587 accessed 16 May 2020.
351. Ghali W, Knudtson M. Overview of the Alberta Provincial Project for Outcome Assessment in Coronary Heart Disease. On behalf of the APPROACH investigators. *Can J Cardiol* 2000;16(10):1225-30.
352. Biomarkers in Acute Cardiac Care (BACC) [Available from: https://clinicaltrials.gov/ct2/show/NCT02355457

For peer review only

accessed 2nd August 2020.

353. Than MP, Pickering JW, Aldous SJ, et al. Effectiveness of EDACS Versus ADAPT Accelerated Diagnostic Pathways for Chest Pain: A Pragmatic Randomized Controlled Trial Embedded Within Practice. *Ann Emerg Med* 2016;68(1):93-102 e1. doi: 10.1016/j.annemergmed.2016.01.001

354. High-Sensitivity Troponin in the Evaluation of Patients With Acute Coronary Syndrome (High-STEACS) [Available from: https://clinicaltrials.gov/ct2/show/NCT01852123 accessed 2nd August 2020.

355. Cullen L, Greenslade JH, Hawkins T, et al. Improved Assessment of Chest pain Trial (IMPACT): assessing patients with possible acute coronary syndromes. *Med J Aust* 2017;207(5):195-200. doi: 10.5694/mja16.01351

356. Kavsak P, Worster A, You J, et al. Ninety-minute vs 3-h performance of high-sensitivity cardiac troponin assays for predicting hospitalization for acute coronary syndrome. *Clin Chem* 2013;59(9):1407–10. doi: 10.1373/clinchem.2013.208595

357. Use of Abbott High Sensitivity Troponin I Assay In Acute Coronary Syndromes (UTROPIA) [Available from: https://clinicaltrials.gov/ct2/show/NCT02060760 accessed 2nd August 2020.

For peer review only

Preferred Reporting Items for Systematic reviews and Meta-Analyses extension for Scoping Reviews (PRISMA-ScR) Checklist

SECTION	ITEM	PRISMA-ScR CHECKLIST ITEM	REPORTED ON PAGE #
**TITLE**			
Title	1	Identify the report as a scoping review.	P.1
**ABSTRACT**			
Structured summary	2	Provide a structured summary that includes (as applicable): background, objectives, eligibility criteria, sources of evidence, charting methods, results, and conclusions that relate to the review questions and objectives.	P.2
**INTRODUCTION**			
Rationale	3	Describe the rationale for the review in the context of what is already known. Explain why the review questions/objectives lend themselves to a scoping review approach.	P.4-5
Objectives	4	Provide an explicit statement of the questions and objectives being addressed with reference to their key elements (e.g., population or participants, concepts, and context) or other relevant key elements used to conceptualize the review questions and/or objectives.	P.5
**METHODS**			
Protocol and registration	5	Indicate whether a review protocol exists; state if and where it can be accessed (e.g., a Web address); and if available, provide registration information, including the registration number.	P.5
Eligibility criteria	6	Specify characteristics of the sources of evidence used as eligibility criteria (e.g., years considered, language, and publication status), and provide a rationale.	P.6-7
Information sources*	7	Describe all information sources in the search (e.g., databases with dates of coverage and contact with authors to identify additional sources), as well as the date the most recent search was executed.	P.6
Search	8	Present the full electronic search strategy for at least 1 database, including any limits used, such that it could be repeated.	P.6
Selection of sources of evidence†	9	State the process for selecting sources of evidence (i.e., screening and eligibility) included in the scoping review.	P.6-7
Data charting process‡	10	Describe the methods of charting data from the included sources of evidence (e.g., calibrated forms or forms that have been tested by the team before their use, and whether data charting was done independently or in duplicate) and any processes for obtaining and confirming data from investigators.	P7-9
Data items	11	List and define all variables for which data were sought and any assumptions and simplifications made.	P.7-8
Critical appraisal of individual sources of evidence§	12	If done, provide a rationale for conducting a critical appraisal of included sources of evidence; describe the methods used and how this information was used in any data synthesis (if appropriate).	P.7-9
Synthesis of	13	Describe the methods of handling and summarizing	P.7-9

SECTION	ITEM	PRISMA-ScR CHECKLIST ITEM	REPORTED ON PAGE #
results		the data that were charted.	
**RESULTS**			
Selection of sources of evidence	14	Give numbers of sources of evidence screened, assessed for eligibility, and included in the review, with reasons for exclusions at each stage, ideally using a flow diagram.	P.9; Figure 1; Appendix Table 2
Characteristics of sources of evidence	15	For each source of evidence, present characteristics for which data were charted and provide the citations.	Appendix Tables 1 and 2; Appendix Figure 1
Critical appraisal within sources of evidence	16	If done, present data on critical appraisal of included sources of evidence (see item 12).	P.9-13; Table 1; Figure 2; Appendix Figure 1
Results of individual sources of evidence	17	For each included source of evidence, present the relevant data that were charted that relate to the review questions and objectives.	Appendix Tables 1 and 2
Synthesis of results	18	Summarize and/or present the charting results as they relate to the review questions and objectives.	P. 9-13; Table 1; Figure 2; Appendix Figure 1
**DISCUSSION**			
Summary of evidence	19	Summarize the main results (including an overview of concepts, themes, and types of evidence available), link to the review questions and objectives, and consider the relevance to key groups.	P. 14
Limitations	20	Discuss the limitations of the scoping review process.	P. 15-16
Conclusions	21	Provide a general interpretation of the results with respect to the review questions and objectives, as well as potential implications and/or next steps.	P. 17
**FUNDING**			
Funding	22	Describe sources of funding for the included sources of evidence, as well as sources of funding for the scoping review. Describe the role of the funders of the scoping review.	P. 18-19

JBI = Joanna Briggs Institute; PRISMA-ScR = Preferred Reporting Items for Systematic reviews and Meta-Analyses extension for Scoping Reviews.
* Where *sources of evidence* (see second footnote) are compiled from, such as bibliographic databases, social media platforms, and Web sites.
† A more inclusive/heterogeneous term used to account for the different types of evidence or data sources (e.g., quantitative and/or qualitative research, expert opinion, and policy documents) that may be eligible in a scoping review as opposed to only studies. This is not to be confused with *information sources* (see first footnote).
‡ The frameworks by Arksey and O'Malley (6) and Levac and colleagues (7) and the JBI guidance (4, 5) refer to the process of data extraction in a scoping review as data charting.
§ The process of systematically examining research evidence to assess its validity, results, and relevance before using it to inform a decision. This term is used for items 12 and 19 instead of "risk of bias" (which is more applicable to systematic reviews of interventions) to include and acknowledge the various sources of evidence that may be used in a scoping review (e.g., quantitative and/or qualitative research, expert opinion, and policy document).

From: Tricco AC, Lillie E, Zarin W, O'Brien KK, Colquhoun H, Levac D, et al. PRISMA Extension for Scoping Reviews (PRISMAScR): Checklist and Explanation. Ann Intern Med. 2018;169:467–473. doi: 10.7326/M18-0850.

For peer review only

BMJ Open

Serial high-sensitivity cardiac troponin testing for the diagnosis of myocardial infarction: A scoping review

Journal:	*BMJ Open*
Manuscript ID	bmjopen-2022-066429.R1
Article Type:	Original research
Date Submitted by the Author:	21-Oct-2022
Complete List of Authors:	Ohtake, Hirotaka; Fujita Health University, Department of Emergency and General Internal Medicine<br>Terasawa, Teruhiko; Fujita Health University, Department of Emergency and General Internal Medicine<br>Zhelev, Zhivko; University of Exeter, University of Exeter Medical School<br>Iwata, Mitsunaga; Fujita Health University, Department of Emergency and General Internal Medicine<br>Rogers, Morwenna; University of Exeter, NIHR CLAHRC South West Peninsula<br>Peters, Jaime; University of Exeter, Peninsula Technology Assessment Group (PenTAG); University of Exeter, Peninsula Technology Assessment Group (PenTAG)<br>Hyde, Chris; University of Exeter, Exeter Test Group
**Primary Subject Heading**:	Cardiovascular medicine
Secondary Subject Heading:	Cardiovascular medicine, Emergency medicine, Diagnostics, Evidence based practice
Keywords:	Coronary heart disease < CARDIOLOGY, Ischaemic heart disease < CARDIOLOGY, Myocardial infarction < CARDIOLOGY, Cardiology < INTERNAL MEDICINE

SCHOLARONE™
Manuscripts

I, the Submitting Author has the right to grant and does grant on behalf of all authors of the Work (as defined in the below author licence), an exclusive licence and/or a non-exclusive licence for contributions from authors who are: i) UK Crown employees; ii) where BMJ has agreed a CC-BY licence shall apply, and/or iii) in accordance with the terms applicable for US Federal Government officers or employees acting as part of their official duties; on a worldwide, perpetual, irrevocable, royalty-free basis to BMJ Publishing Group Ltd ("BMJ") its licensees and where the relevant Journal is co-owned by BMJ to the co-owners of the Journal, to publish the Work in this journal and any other BMJ products and to exploit all rights, as set out in our licence.

The Submitting Author accepts and understands that any supply made under these terms is made by BMJ to the Submitting Author unless you are acting as an employee on behalf of your employer or a postgraduate student of an affiliated institution which is paying any applicable article publishing charge ("APC") for Open Access articles. Where the Submitting Author wishes to make the Work available on an Open Access basis (and intends to pay the relevant APC), the terms of reuse of such Open Access shall be governed by a Creative Commons licence – details of these licences and which Creative Commons licence will apply to this Work are set out in our licence referred to above.

Other than as permitted in any relevant BMJ Author's Self Archiving Policies, I confirm this Work has not been accepted for publication elsewhere, is not being considered for publication elsewhere and does not duplicate material already published. I confirm all authors consent to publication of this Work and authorise the granting of this licence.

For peer review only

Serial high-sensitivity cardiac troponin testing for the diagnosis of myocardial infarction: A scoping review

Hirotaka Ohtake[1], Teruhiko Terasawa[1], Zhivko Zhelev[2], Mitsunaga Iwata[1], Morwenna Rogers[3], Jaime L Peters[2], Chris Hyde[2]

[1] Department of Emergency and General Internal Medicine, Fujita Health University School of Medicine, Toyoake, Japan

[2] Exeter Test Group, University of Exeter Medical School, University of Exeter, Exeter, Devon, UK

[3] NIHR ARC South West Peninsula, University of Exeter Medical School, Exeter, UK.

Corresponding author: Teruhiko Terasawa, MD, PhD; Department of Emergency and General Internal Medicine, Fujita Health University School of Medicine; 1-98 Dengakugakubo, Kutsukake-cho, Toyoake, Aichi 470-1192, Japan; terasawa@fujita-hu.ac.jp

For peer review only

For peer review only - http://bmjopen.bmj.com/site/about/guidelines.xhtml

Abstract

***Objectives**:* We aimed to assess the diversity and practices of existing studies on several assays and algorithms for serial measurements of high-sensitivity cardiac troponin (hs-cTn) for risk stratification and the diagnosis of myocardial infarction (MI) and 30-day outcomes in patients suspected of having non-ST-segment elevation MI (NSTEMI).

Methods: We searched multiple databases including MEDLINE, EMBASE, Science Citation Index, the Cochrane Database of Systematic Reviews, and the CENTRAL databases for studies published between January 2006 and November 2021. Studies that assessed the diagnostic accuracy of serial hs-cTn testing in patients suspected of having NSTEMI in the emergency department (ED) were eligible. Data were analysed using the scoping review method.

Results: We included 86 publications mainly from research centres in Europe, North America, and Australasia. Two hs-cTn assays, manufactured by Abbott (43/86) and Roche (53/86), dominated the evaluations. The studies most commonly measured the concentrations of hs-cTn at two time points, at presentation and a few hours thereafter, to assess the two- or three-strata algorithm for diagnosing or ruling out MI. Although data from 83 studies (97%) were prospectively collected, 0 to 90% of the eligible patients were excluded from the analysis due to missing blood samples or the lack of a final diagnosis in 53 studies (62%) that reported relevant data. Only 19 studies (22%) reported on head-to-head comparisons of alternative assays.

Conclusion: Evidence on the accuracy of serial hs-cTn testing was largely derived from selected research institutions and relied on two specific assays. The proportions of the eligible patients excluded from the study raise concerns about directly applying the study findings to clinical practice in frontline EDs.

For peer review only - http://bmjopen.bmj.com/site/about/guidelines.xhtml

Strengths and limitations of this study

- This is the first scoping review that explored the diversity of study methodologies adopted in studies on testing with serial high-sensitivity cardiac troponin measurements in patients with suspected non-ST elevation myocardial infarction in the emergency department.
- The review method included a comprehensive literature search, duplicate assessment of eligibility, double extraction of data, and descriptive synthesis through tables and graphs.
- The assessed specifications included the regions and sources of studies, targeted participants and their study flow, troponin assays, sampling algorithms and direct comparisons thereof, and definitions of outcomes.
- The quantitative results about the accuracy and clinical outcomes were not assessed because this was beyond the scope of the review.

For peer review only

For peer review only - http://bmjopen.bmj.com/site/about/guidelines.xhtml

INTRODUCTION

Acute coronary syndrome (ACS) is considered a major cause of death worldwide.[1,2] Acute myocardial infarction (AMI) is a form of ACS, which represents permanent cellular damage in the affected myocardium due to ischemia. AMI is clinically subcategorized into ST-segment elevation myocardial infarction (STEMI) and non-ST-segment elevation myocardial infarction (NSTEMI). Each type of AMI has a unique prognosis, and their managements differ substantially. Since STEMI is an acute life-threatening condition, prompt reperfusion therapy is essential. In contrast, because the prognosis of NSTEMI varies depending on its etiology, accurate diagnosis and risk stratification based on medical history, electrocardiogram (ECG) findings, and cardiac biomarker concentrations are of paramount significance in patients suspected of ACS.[3,4]

For the clinical management of NSTEMI, cardiac troponin (cTn) has been used as the mainstay of clinical diagnosis since 2000.[5-8] To avoid unnecessary hospital admissions and expedite the diagnostic process, high-sensitivity cardiac Tn (hs-cTn), a group of more sensitive cTn assays,[9] has been introduced into clinical practice since 2010. Although several primary studies and meta-analyses on the single measurement of hs-cTn reported their high sensitivity and specificity,[10-12] several challenges persist. First, blood concentrations of hs-cTn troponin take two to three hours to increase, and they may not be detectable within three hours from the onset of AMI.[3,13] Second, despite its high sensitivity, elevated concentrations of hs-cTn are observed in several clinical conditions other than AMI, including acute myocardial injury (e.g. acute heart failure, and tachyarrhythmia) and chronic myocardial injury (e.g.

For peer review only - http://bmjopen.bmj.com/site/about/guidelines.xhtml

structural heart disease and chronic heart failure).[3,8,13] To differentiate these conditions, serial measurements of hs-cTn, i.e., assessing the absolute and/or relative changes of repeated measurements, were proposed to increase the specificity for diagnosing acute MI. Based on several studies on serial hs-cTn testing algorithms with high sensitivity and high negative predictive value, the current clinical guidelines on NSTE-ACS recommend serial measurements of hs-cTn at presentation and after 1-3 hours.[3,4]

However, the comparative effectiveness of management strategies based on serial hs-cTn measurements has not been fully elucidated, because several alternative assays are clinically available and existing reports are from studies with different designs and inconsistent testing algorithms. Thus, this study aimed to explore the diversity of the methodologies used in primary studies on serial hs-cTn measurements in patients suspected of having ACS in the emergency department (ED). We constructed an evidence map of existing studies on serial hs-cTn testing for diagnosing NSTEMI and predicting 30-day clinical outcomes. We critically appraised the currently available evidence and highlighted the issues that need to be addressed in future research.

METHODS

This study is a focused analysis performed in conjunction with a registered systematic review project (PROSPERO registration number CRD42018106379). The protocol for the original systematic review is available at https://bmjopen.bmj.com/content/9/3/e026012.long.[10] Since it was a secondary

For peer review only - http://bmjopen.bmj.com/site/about/guidelines.xhtml

analysis of publicly available data, no ethical review was required. This report followed the PRISMA Extension for Scoping Reviews.[14]

Data search

We searched Ovid MEDLINE, EMBASE, Science Citation Index, and Cochrane Database of Systematic Reviews for studies published between 1 January 2006 and 17 November 2021 with no restrictions of language or publication status. The search terms included “chest pain”, “acute coronary syndrome,” “myocardial infarction,” “cardiac troponin”, “emergency room” and their synonyms.[10] The full search strategy is available in **Appendix**. We excluded editorials, letters, comments, conference abstracts, review articles, and meta-analyses. Also, we excluded studies assessing clinical prediction rules (e.g., Global Registry of Acute Coronary Events (GRACE) Risk Score[15]).

Study eligibility

We included prospective and retrospective studies that evaluated patients aged ≥18 years who were suspected of having NSTEMI in an ED and had two or more serial troponin measurements using an hs-cTn assay. Eligible were studies that reported the diagnostic accuracy of AMI and/or 30-day clinical outcomes. We only included single-gate design studies, i.e., studies that consisted of a single group of subjects based on a single eligibility criteria.[16] Studies that included mixed populations of patients—with suspected STEMI and NSTEMI—were included only when data for the patients with suspected NSTEMI were separately extractable. Studies that exclusively assessed patients with suspected STEMI were excluded. Two investigators double-screened the titles and abstracts and examined the full-text articles to assess eligibility. We defined

For peer review only - http://bmjopen.bmj.com/site/about/guidelines.xhtml

hs-cTn as assays that satisfied the requirements of the International Clinical Federation of Clinical Chemistry and Laboratory Medicine (i.e., <10% coefficient of variation at the 99th percentile, and ≥ 50% measurable concentrations above the limit of detection for both males and females).[9] Discrepancies were resolved by consensus.

Data extraction

The following data were extracted: 1) publication and study characteristics: authors, journal name, publication year, enrolment years, number of eligible and included patients, study design, the name of the study cohort(s), geographical region(s), participant age and use of ECG to exclude patients; 2) test characteristics: assays, the timing of blood sampling, cut-off values, algorithms adopted (binary testing algorithms for ruling out MI *vs.* three-strata testing algorithms for stratifying patients into three different risk groups, i.e., high-, intermediate-, and low-risk groups commonly referred to as “rule-in,” “observational zone,” and “rule-out” for MI diagnosis); 3) reference standard characteristics: specific diagnostic criteria of MI, such as those defined in clinical guidelines and/or versions of the universal definition of MI, and the assessors of the final diagnoses.

Operationalisation

Our target population was a group of patients suspected of having NSTEMI who presented at the ED. We recorded the numbers of patients suspected of having NSTEMI who presented at the ED, patients who completed one or more study-specific serial testing algorithm(s), and patients who were assessed for test accuracy and/or 30-day clinical outcomes. A testing algorithm was specified based on the number and timing of

For peer review only - http://bmjopen.bmj.com/site/about/guidelines.xhtml

hs-cTn measurements. The results for the algorithm were typically reported as a single value measured at presentation, together with an absolute or a relative difference between specific measurement time points (typically at presentation and a few hours later), which was categorised as delta or percent change in the hs-cTn concentrations. We classified the studies that involved two hs-cTn measurements into three groups, namely, 0 and 1h, 0 and 2h, and 0 and 3h, based on the blood sampling timing (in hours) of the first and second samples. Other algorithms involving three or more blood samples were grouped into a separate category, labelled as "others." Assays were classified by specific manufacturers, i.e., Abbott (Abbott Laboratories, Illinois, USA), Roche (Roche Diagnostics, Basel, Switzerland), Siemens (Siemens Healthcare, Erlangen, Germany), and Beckman (Beckman Coulter, California, USA). Assays by other manufacturers were categorised as "miscellaneous."

To assess the evidence, we used the study with the largest sample to avoid double-counting when multiple studies reported (partially) overlapping patient populations. For the study locations, we assumed that each specific research institution involved in the study assessed patients residing within its geographical region only. Comparative studies were defined as studies that adopted a paired design to assess multiple assays on the same study participants and directly compared the diagnostic accuracy for AMI or 30-day clinical outcomes. This review did not standardise the definition of AMI or the 30-day clinical outcomes and adopted the study-reported outcome definitions as specifically reported.

Analyses

For peer review only - http://bmjopen.bmj.com/site/about/guidelines.xhtml

We considered each publication to be the unit of analysis and performed descriptive analyses using percentages or medians and ranges. We combined data as a weighted average only if the pertinent data were available for specific subgroups. The assessed design specifications included the regions and sources of studies, characteristics of targeted participants and their study flow, specific troponin assays used, sampling algorithms with their operational characteristics, direct comparisons of two or more algorithms and/or assays, and definitions of the index MI and 30-day outcomes.

The volume of clinical evidence was assessed with graphs and tables using Stata 17.0 (Stata Corp. College Station, TX, USA). The graphical presentation of the study locations was constructed using Google Maps (Google Inc., Mountain View, CA, USA) and Mapcustomizer (available at https://www.mapcustomizer.com/).

Patient and public involvement

We did not involve patients or the public in the preparation of this scoping review.

RESULTS

Inclusion of primary studies

Figure 1 shows the PRISMA flow diagram for this scoping review. Our search yielded 6838 articles; 6230 of them were excluded after examining the titles and abstracts. After excluding 549 perused full-text articles, we finally included 86 publications, including 72 reports on test accuracy and 52 on 30-day clinical outcomes (**Appendix Table 1**). [17-102] The excluded articles are listed in **Appendix Table 2**.

For peer review only - http://bmjopen.bmj.com/site/about/guidelines.xhtml

Study characteristics

Eighty-three studies (97%) were based on prospectively collected blood samples, and the median sample size was 1391 (range, 160-6403) (**Appendix Table 1**). The most commonly assessed assays were manufactured by Abbot (n=41) and Roche (n=53), followed by those manufactured by Siemens (n=10) and Beckman (n=6). Three studies assessed point-of-care assays that met the definition of hs-cTn.[76,80,102] Serial hs-cTn testing was predominantly assessed in Europe and North America and the participating institutions were limited to specific research centres (**Appendix Table 1**, **Appendix Table 3**, **and Appendix Figure 1**). In contrast, only a few research centres per country from Australasia and Asia participated and provided pertinent data.

Of the 86 cohorts that reported accuracy and/or 30-day outcome data using a specific assay and a sampling algorithm, only 42 (49%) were considered unique, which included 78,606 non-overlapping patients (**Appendix Table 3**). Of the 42 studies, 12 (29%) studies that assessed the assays manufactured by Abbott involved unique cohorts (seven, three, and eight reports on the 0 and 1h, 0 and 2h, and 0 and 3h protocols, respectively). Similarly, data from 25 of 42 studies (60%) that assessed the assays manufactured by Roche involved unique cohorts (16, five, and four reports on the 0 and 1h, 0 and 2h, and 0 and 3h protocols, respectively).

Nineteen studies compared two or more assays using a paired design (**Table 1**). The most commonly reported comparison was between the assays by Abbott and Roche (17 studies on test accuracy and six on 30-day outcomes). Few studies have performed

For peer review only - http://bmjopen.bmj.com/site/about/guidelines.xhtml

head-to-head comparisons involving the same patients. Two studies compared hs-cTn with an earlier generation non-hs cTn assay.[17,51]

Table 1. Direct comparisons of alternative high-sensitivity cardiac troponin assays*

**Hs-cTn assays**	**Abbott**	**Roche**	**Siemens**	**Beckman**
**0 and 1h protocol**				
**Abbott**		6 (10731)	0	0
**Roche**	8 (13779)		0	0
**Siemens**	2 (1235)	1 (418)		0
**Beckman**	1 (278)	1 (278)	1 (278)	
0 and 2h protocol				
Abbott		2 (4063)	0	1 (1118)
Roche	5 (7497)		0	1 (1118)
Siemens	1 (313)	1 (313)		0
Beckman	1 (1118)	1 (1118)	1 (217)	
0 and 3h protocol				
Abbott		1 (2945)	0	0
Roche	4 (6419)		0	0
Siemens	1 (1809)	1 (1809)		0
Beckman	1 (1110)	1 (1110)	1 (1110)	
Miscellaneous				
Abbott		0	0	0
Roche	1 (1735)		0	0
Siemens	0	1 (830)		0
Beckman	0	1 (830)	1 (830)	

* For each algorithm (i.e., 0 and 1h, 0 and 2h, and 0 and 3h protocols and a combined, miscellaneous protocol group described in the left-most column), cells in the lower left and upper right parts (separated by a right-lower diagonal line comprising black closed cells) show the volume of comparative evidence on diagnostic accuracy of acute myocardial infarction and 30-day clinical outcomes, respectively. Each cell represents a specific comparison between assays described in the leftmost column and the top row. The number of comparative studies that compared a specific pair of assays is followed by the number of assessed patients in parentheses.

Hs-cTn = high-sensitivity cardiac troponin

For peer review only

Typically, the studies reported only the number of enrolled patients (i.e., patients suspected of having NSTEMI who were eligible for and agreed to participate in the study), and the complete data on all clinically relevant patients (i.e., the number of all patients suspected of having NSTEMI who presented at the ED) were missing. Fifty-two studies (60%) reported quantitative data on patients who failed to complete a study-specific serial testing algorithm(s) or patients whose diagnosis or 30-day clinical outcomes could not be established (**Appendix Table 1**). Various proportions (median 20%; range, 0%-90%) of enrolled patients were excluded from the analysis, typically due to missing blood samples or the lack of a final diagnosis.

Patient characteristics

The mean or median participant age ranged from 53 to 73 years, and most studies involved patients in their 40s to 70s (**Appendix Table 1**). Only one study[56] specifically focused on 0 and 1h protocol for a subgroup of elderly patients derived from three cohorts, specifically, patients aged 70 years or older. Of 86 studies, 17 (20%) excluded patients with chronic kidney disease (CKD) or those requiring regular haemodialysis. In contrast, only four studies specifically focused on 0 and 1h, 3h or 6-12h protocol for a subgroup of patients with renal dysfunction, which were derived from four cohorts.[61,62,64,65] Only a single study specifically focused on 0 and 3h protocol for a subgroup of female patients only derived from three cohorts. No further studies that focused on these sub populations have been found through the update search and manual search based on the reference lists.[52]

Testing algorithms

For peer review only - http://bmjopen.bmj.com/site/about/guidelines.xhtml

Forty-seven studies (55%) assessed the three-strata algorithms. These studies stratified patients into “rule-out (low-risk),” “observational zone (intermediate-risk),” and “rule-in (high-risk)” groups according to two sets of diagnostic criteria based on the concentrations of hs-cTn at baseline, 1-3 hours, and/or the difference between the hs-cTn concentrations of the two samples. Other studies conventionally categorised the patients into two strata (i.e. “rule-out (low-risk)” and “rule-in (high-risk)” groups) based on a single set of criteria. The majority of data were based on assays manufactured by either Abbott or Roche, and the 0 and 1h algorithm was the most frequently reported (**Figure 2**).

Outcomes

Eighty-four studies (98%) adopted the universal definition of MI (versions 2007, 2012, and/or 2018)[6-8] to establish a diagnosis of AMI. A few of these studies also followed the guidelines proposed by the American College of Cardiology (ACC) (1/86, 1%),[103] the ACC and the American Heart Association (AHA) guidelines (1/86, 1%),[104] ACC and the European Society of Cardiology (ESC)[105], and the ACC/AHA[104,106] and ESC[3,107] guidelines (4/86, 5%) in addition to either version of the universal definition. Two studies[17,21] relied on the ACC guidelines[103] alone. The adjudicators of the clinical diagnosis of AMI were cardiologists in 72 studies (84%). The studies variably reported 30-day outcomes; of the 51 studies that reported one or more 30-day outcomes, 11 (22%) reported all-cause mortality, and 41 (80%) reported cardiac death, whereas 36 (68%) reported major adverse cardiac events, a composite outcome including AMI, as well as cardiac death or death from all causes. Other reported clinical outcomes observed within 30 days included urgent

For peer review only - http://bmjopen.bmj.com/site/about/guidelines.xhtml

revascularization, percutaneous coronary intervention, coronary artery bypass graft (19/51, 37%), and ventricular arrhythmia (11/51, 22%). Twenty-seven studies (53%) also reported long-term clinical outcomes, which included events that developed within up to two years.[34-37,45,57,62,65,75,94,95]

Discussion

To the best of our knowledge, this is the first scoping review to comprehensively assess the reports on testing with serial hs-cTn measurements in patients with suspected NSTEMI in the ED. We summarised how studies measured serial hs-cTn and assessed its diagnostic accuracy for AMI and the 30-day clinical outcomes. Our results showed that most existing data were based on the Abbott cTnI or Roche cTnT assays, and the timing of the blood measurements and the diagnostic algorithms varied. The number of studies assessing these two assays using the 0 and 1h, 0 and 2h, and 0 and 3h protocols has been continuously increasing since 2011 when guidelines[29] recommended serial hs-cTn measurements; fewer studies have assessed other assays and/or alternative algorithms. Limited data on patients with CKD or older adults, as well as data stratified by sex, were reported,[62,64] which were deemed still under evaluation. Most studies followed the universal definition to diagnose the index MI. However, in addition to North America or Europe, only a limited number of research teams involving several specialty institutions in specific countries have contributed to the current evidence. Most importantly, the studies excluded variable proportions of eligible patients from the analysis due to missing blood samples or concrete final diagnoses.

For peer review only

Strengths

We comprehensively explored the existing evidence, focusing on how the studies were designed, analysed, and reported and, the implications for clinical practice of the identified limitations and concerns. Previously reported systematic reviews focused on a two-strata rule-out strategy using unique serial measurements of hs-cTnT assay only[108] or the 0 and 1h three-strata strategy only[109] regardless of the assays assessed, and they did not perform a comprehensive field synopsis covering all relevant information. The objective of our scoping review is to describe the diversity in the adopted study methodologies together with their potential limitations following the standard scoping review methods. Therefore, this review should be an additional view that follows the recently published critical appraisal of the current evidence base,[110][111] both of which will help identify the current evidence gaps as well as help design future studies.

Limitation

This scoping review performed a focused analysis on the reported study methodologies and did not address the primary objectives of the originally planned systematic review. Therefore, several limitations need to be discussed. First, we did not assess the quantitative results on accuracy and other clinical outcomes because this was beyond the scope of the present scoping review. Second, we focused on studies that assessed only test accuracy and 30-day outcomes. Therefore, data on studies that evaluated long-term outcomes have not been addressed. Third, since we excluded conference abstracts, this review may have missed newer relevant publications. Fourth, the recently developed clinical prediction rules (e.g., History, ECG, Age, Risk factors and Troponin

For peer review only - http://bmjopen.bmj.com/site/about/guidelines.xhtml

(HEART) Score,[112] Emergency Department Assessment of Chest Pain (EDACS) Score[113], and GRACE Risk Score[15]) have consistently incorporated hs-cTn concentrations as a component variable.[40] We excluded these studies that used hs-cTn as part of risk prediction models. Finally, several studies have reported that higher hs-cTn concentrations were associated with increased mortality in patients with suspected ACS; however, our review did not address this association.[34,36,37,45,57,62,65]

Clinical implications

Recently, a pooled study conducted by Neumann et al. proposed a high-accurate risk-assessment tool for NSTEMI using a serial hs-cTn measurements based on an international consortium of prospectively registered data from 15 cohorts. Of these, nine cohorts were also assessed in this scoping review, at least some portions of which should have contributed to the dataset deriving the risk-assessment tool. Given the methodological concerns raised in this review, depending on how the study methodologies in the rest of the data have been addressed and/or improved, uncertainties still exist in its individualized application in real-life clinical practice. First, ACS and MI are common in elderly populations as well as patients with CKD,[114,115] while the eligible studies in our review mainly focused on middle-aged populations, and approximately a third of the studies excluded patients with impaired renal function. Additionally, hs-cTnI concentrations are generally higher in men than in women,[116] which bases the use of sex-specific cut-offs recommended by the fourth Universal definition of MI in contrast to the uniform cut-off adopted in the risk-assessment tool.[117] The recent critical appraisal report[111] is also in line with this individualized approach. Moreover, quality specifications of the assays are also

For peer review only - http://bmjopen.bmj.com/site/about/guidelines.xhtml

relevant. For example, critical factors, which are appropriate for collecting and measuring samples and applying the results to clinical practices.[118] Therefore, the optimal sample timings and cut-off values need to be validated on an individualized basis and account for age, sex, and renal function under the appropriate quality control.[119] [120] Second, our review found that the existing data were largely based on two assays by two manufacturers (i.e., assays manufactured by Abbott and Roche); the evidence is sparse for the others. Furthermore, evidence on hs-cTnT and hs-cTnI has limited data concerning direct comparative studies of assays. Therefore, comparative studies are needed since systematic differences between hs-cTnT and hs-cTnI as well as among hs-cTnI methods have been reported.[121] Third, most of the included studies were conducted in specialised centres in Europe, North America, or Australasia. A recent meta-analysis of diagnostic accuracy that focused on only the 0 and 1h algorithm[109] pointed out that sensitivity was not universally high across cohorts, as reported in the primary studies in these specialised centres; reproducibility of the excellent results appeared to be limited for the studies from Asia. Given this observation, validation in these regions is required. Fourth, the studies included in our review missed variable proportions of clinically relevant patients who presented at EDs with suspected NSTEMI. This appears to stem, at least in part, from convenience sampling. The failure to apply gold standard tests to all participants, may also have been responsible for the excluded cases without an established diagnosis of the cause of chest pain, which is inevitable in real-life clinical settings. These methodological weaknesses would have distorted, at least to some extent, the typical disease spectrum of clinically relevant populations. Our review failed to address how this patient loss affected the study results.

For peer review only - http://bmjopen.bmj.com/site/about/guidelines.xhtml

CONCLUSIONS

Data on diagnostic test accuracy and short-term outcomes by serial hs-cTn measurements were largely derived from particular research institutions in Europe, North America, or Australasia and based mainly on two specific assays. The exclusion of variable proportions of eligible patients, which was inevitable even in well-conducted prospective studies, raised concerns regarding the studies' generalizability and direct applications in real-world ED clinical practice.

For peer review only

For peer review only - http://bmjopen.bmj.com/site/about/guidelines.xhtml

Acknowledgments

The authors thank Drs Chihiro Kato and Jun Shinohara for assisting with data extraction.

Contributors

TT and ZZ lead the protocol development. HO, TT, ZZ, MI, MR, JLP, and CH drafted and revised the protocol. MR performed the literature searches. HO, TT, ZZ, JLP determined the eligibility of primary studies and acquired the data. HO and TT analysed the data. HO, TT, ZZ, MI, MR, JLP, and CH interpreted the findings. HO and TT drafted the first version of the report. HO, TT, ZZ, MI, MR, JLP, and CH critically read the manuscript and provided feedback for revision. HO, TT, ZZ, MI, MR, JLP, and CH read and approved the final manuscript.

Competing interests

The authors declare no conflicts of interest associated with this study.

Funding

This study was supported in part by a research grant from the Ministry of Education, Culture, Sports, Science, and Technology (MEXT) of Japan (18K08902) and by the National Institute for Health Research (NIHR) Collaboration for Leadership in Applied Health Research and Care South West Peninsula (NIHR CLAHRC South West Peninsula; grant no, N/A). The funders had no involvement in the study design, data collection and analysis, decision to publish, or preparation of the manuscript. The views expressed in this publication are those of the author(s) and not necessarily those of

For peer review only - http://bmjopen.bmj.com/site/about/guidelines.xhtml

MEXT in Japan, the National Institute for Health Research, or the Department of Health and Social Care.

Ethics statements

Patient consent for publication

Not required.

Ethics approval

Since this scoping review was a secondary analysis of publicly available data, no ethical review was required.

Data Availability Statement

All data relevant to the study are included in the article or uploaded as supplementary information.

ORCID iDs

Hirotaka Ohtake: https://orcid.org/0000-0002-0223-4789
Teruhiko Terasawa: https://orcid.org/0000-0002-0975-391X
Zhivko Zhelev: https://orcid.org/0000-0002-0106-2401
Mitsunaga Iwata: https://orcid.org/0000-0001-8979-4801
Morwenna Rogers: https://orcid.org/0000-0002-6039-238X
Jaime L Peters: https://orcid.org/0000-0003-1778-3518
Chris Hyde: https://orcid.org/0000-0002-7349-0616

For peer review only - http://bmjopen.bmj.com/site/about/guidelines.xhtml

REFERENCES

1. Organization WH. Cardiovascular diseases (CVDs): Key facts 2017 [Available from: https://www.who.int/en/news-room/fact-sheets/detail/cardiovascular-diseases-(cvds) accessed 10 May 2020.

2. Reed GW, Rossi JE, Cannon CP. Acute myocardial infarction. *The Lancet* 2017;389(10065):197-210. doi: 10.1016/s0140-6736(16)30677-8

3. Collet JP, Thiele H, Barbato E, et al. 2020 ESC Guidelines for the management of acute coronary syndromes in patients presenting without persistent ST-segment elevation. *Eur Heart J* 2021;42(14):1289-367. doi: 10.1093/eurheartj/ehaa575

4. Amsterdam EA, Wenger NK, Brindis RG, et al. 2014 AHA/ACC Guideline for the Management of Patients with Non-ST-Elevation Acute Coronary Syndromes: a report of the American College of Cardiology/American Heart Association Task Force on Practice Guidelines. *J Am Coll Cardiol* 2014;64(24):e139-e228. doi: 10.1016/j.jacc.2014.09.017

5. Alpert J, Thygesen K, Antman E, et al. Myocardial infarction redefined—a consensus document of The Joint European Society of Cardiology/American College of Cardiology committee for the redefinition of myocardial infarction. *J Am Coll Cardiol* 2000;36(3):959-69. doi: 10.1016/s0735-1097(00)00804-4

6. Thygesen K, Alpert JS, White HD, et al. Universal definition of myocardial infarction. *Circulation* 2007;116(22):2634-53. doi: 10.1161/CIRCULATIONAHA.107.187397

7. Thygesen K, Alpert JS, Jaffe AS, et al. Third universal definition of myocardial infarction. *Circulation* 2012;126(16):2020-35. doi: 10.1161/CIR.0b013e31826e1058

8. Thygesen K, Alpert JS, Jaffe AS, et al. Fourth universal definition of myocardial infarction (2018). *Eur Heart J* 2019;40(3):237-69. doi: 10.1093/eurheartj/ehy462

9. High-Sensitivity* Cardiac Troponin I and T Assay Analytical Characteristics Designated by Manufacturer IFCC Committee on Clinical Applications of Cardiac Bio-Markers [Available from: https://www.ifcc.org/media/477656/high-sensitivity-cardiac-troponin-i-and-t-assay-analytical-characteristics-designated-by-manufacturer-v012019.pdf.

10. Zhelev Z, Ohtake H, Iwata M, et al. Diagnostic accuracy of contemporary and high-sensitivity cardiac troponin assays used in serial testing, versus single-sample testing as a comparator, to triage patients suspected of acute non-ST-segment

For peer review only - http://bmjopen.bmj.com/site/about/guidelines.xhtml

elevation myocardial infarction: a systematic review protocol. *BMJ Open* 2019;9(3):e026012. doi: 10.1136/bmjopen-2018-026012

11. Pickering JW, Than MP, Cullen L, et al. Rapid Rule-out of Acute Myocardial Infarction With a Single High-Sensitivity Cardiac Troponin T Measurement Below the Limit of Detection: A Collaborative Meta-analysis. *Ann Intern Med* 2017;166(10):715-24. doi: 10.7326/M16-2562
12. Chapman AR, Lee KK, McAllister DA, et al. Association of High-Sensitivity Cardiac Troponin I Concentration With Cardiac Outcomes in Patients With Suspected Acute Coronary Syndrome. *JAMA* 2017;318(19):1913-24. doi: 10.1001/jama.2017.17488
13. Weber M, Bazzino O, Navarro Estrada JL, et al. Improved diagnostic and prognostic performance of a new high-sensitive troponin T assay in patients with acute coronary syndrome. *Am Heart J* 2011;162(1):81-8. doi: 10.1016/j.ahj.2011.04.007
14. Tricco AC, Lillie E, Zarin W, et al. PRISMA Extension for Scoping Reviews (PRISMA-ScR): Checklist and Explanation. *Ann Intern Med* 2018;169(7):467-73. doi: 10.7326/M18-0850
15. Fox KA, Dabbous OH, Goldberg RJ, et al. Prediction of risk of death and myocardial infarction in the six months after presentation with acute coronary syndrome: prospective multinational observational study (GRACE). *BMJ* 2006;333(7578):1091. doi: 10.1136/bmj.38985.646481.55
16. Rutjes AW, Reitsma JB, Vandenbroucke JP, et al. Case-control and two-gate designs in diagnostic accuracy studies. *Clin Chem* 2005;51(8):1335-41. doi: 10.1373/clinchem.2005.048595
17. Aldous SJ, Florkowski CM, Crozier IG, et al. Comparison of high sensitivity and contemporary troponin assays for the early detection of acute myocardial infarction in the emergency department. *Ann Clin Biochem* 2011;48(Pt 3):241-8. doi: 10.1258/acb.2010.010219
18. Aldous SJ, Richards AM, Cullen L, et al. Early dynamic change in high-sensitivity cardiac troponin T in the investigation of acute myocardial infarction. *Clin Chem* 2011;57(8):1154-60. doi: 10.1373/clinchem.2010.161166
19. Keller T, Zeller T, Ojeda F, et al. Serial Changes in Highly Sensitive Troponin I Assay and Early Diagnosis of Myocardial Infarction. *JAMA* 2011;306(24):2684-93. doi: 10.1001/jama.2011.1896
20. Reichlin T, Irfan A, Twerenbold R, et al. Utility of absolute and relative changes in cardiac troponin concentrations in the early diagnosis of acute myocardial

For peer review only - http://bmjopen.bmj.com/site/about/guidelines.xhtml

infarction. *Circulation* 2011;124(2):136-45. doi: 10.1161/CIRCULATIONAHA.111.023937

21. Aldous S, Pemberton C, Richards AM, et al. High-sensitivity troponin T for early rule-out of myocardial infarction in recent onset chest pain. *Emerg Med J* 2012;29(10):805-10. doi: 10.1136/emermed-2011-200222
22. Reichlin T, Schindler C, Drexler B, et al. One-hour rule-out and rule-in of acute myocardial infarction using high-sensitivity cardiac troponin T. *Arch Intern Med* 2012;172(16):1211-8. doi: 10.1001/archinternmed.2012.3698
23. Schreiber DH, Agbo C, Wu AH. Short-term (90 min) diagnostic performance for acute non-ST segment elevation myocardial infarction and 30-day prognostic evaluation of a novel third-generation high sensitivity troponin I assay. *Clin Biochem* 2012;45(16-17):1295-301. doi: 10.1016/j.clinbiochem.2012.06.005
24. Cullen L, Mueller C, Parsonage WA, et al. Validation of high-sensitivity troponin I in a 2-hour diagnostic strategy to assess 30-day outcomes in emergency department patients with possible acute coronary syndrome. *J Am Coll Cardiol* 2013;62(14):1242-49. doi: 10.1016/j.jacc.2013.02.078
25. Irfan A, Reichlin T, Twerenbold R, et al. Early diagnosis of myocardial infarction using absolute and relative changes in cardiac troponin concentrations. *Am J Med* 2013;126(9):781-88 e2. doi: 10.1016/j.amjmed.2013.02.031
26. Aldous S, Mark Richards A, George PM, et al. Comparison of new point-of-care troponin assay with high sensitivity troponin in diagnosing myocardial infarction. *Int J Cardiol* 2014;177(1):182-6. doi: 10.1016/j.ijcard.2014.09.026
27. Cullen L, Aldous S, Than M, et al. Comparison of high sensitivity troponin T and I assays in the diagnosis of non-ST elevation acute myocardial infarction in emergency patients with chest pain. *Clin Biochem* 2014;47(6):321-6. doi: 10.1016/j.clinbiochem.2013.11.019
28. Rubini Gimenez M, Twerenbold R, Reichlin T, et al. Direct comparison of high-sensitivity-cardiac troponin I vs. T for the early diagnosis of acute myocardial infarction. *Eur Heart J* 2014;35(34):2303-11. doi: 10.1093/eurheartj/ehu188
29. Parsonage WA, Greenslade JH, Hammett CJ, et al. Validation of an accelerated high-sensitivity troponin T assay protocol in an Australian cohort with chest pain. *Med J Aust* 2014;200(3):161-5. doi: 10.5694/mja13.10466
30. Biener M, Mueller M, Vafaie M, et al. Impact of leading presenting symptoms on the diagnostic performance of high-sensitivity cardiac troponin T and on outcomes in patients with suspected acute coronary syndrome. *Clin Chem* 2015;61(5):744-51. doi: 10.1373/clinchem.2014.235317

For peer review only - http://bmjopen.bmj.com/site/about/guidelines.xhtml

31. Eggers KM, Aldous S, Greenslade JH, et al. Two-hour diagnostic algorithms for early assessment of patients with acute chest pain--Implications of lowering the cardiac troponin I cut-off to the 97.5th percentile. *Clin Chim Acta* 2015;445:19-24. doi: 10.1016/j.cca.2015.03.002

32. Rubini Gimenez M, Twerenbold R, Jaeger C, et al. One-hour rule-in and rule-out of acute myocardial infarction using high-sensitivity cardiac troponin I. *Am J Med* 2015;128(8):861-70 e4. doi: 10.1016/j.amjmed.2015.01.046

33. Pickering JW, Young JM, George P, et al. The utility of presentation and 4-hour high sensitivity troponin I to rule-out acute myocardial infarction in the emergency department. *Clin Biochem* 2015;48(18):1219-24. doi: 10.1016/j.clinbiochem.2015.07.033

34. Reichlin T, Twerenbold R, Wildi K, et al. Prospective validation of a 1-hour algorithm to rule-out and rule-in acute myocardial infarction using a high-sensitivity cardiac troponin T assay. *CMAJ* 2015;187(8):E243-52. doi: 10.1503/cmaj.141349/-/DC1

35. Reichlin T, Cullen L, Parsonage WA, et al. Two-hour algorithm for triage toward rule-out and rule-in of acute myocardial infarction using high-sensitivity cardiac troponin T. *Am J Med* 2015;128(4):369-79 e4. doi: 10.1016/j.amjmed.2014.10.032

36. Jaeger C, Wildi K, Twerenbold R, et al. One-hour rule-in and rule-out of acute myocardial infarction using high-sensitivity cardiac troponin I. *Am Heart J* 2016;171(1):92-102 e1-5. doi: 10.1016/j.ahj.2015.07.022

37. Boeddinghaus J, Reichlin T, Cullen L, et al. Two-Hour Algorithm for Triage toward Rule-Out and Rule-In of Acute Myocardial Infarction by Use of High-Sensitivity Cardiac Troponin I. *Clin Chem* 2016;62(3):494-504. doi: 10.1373/clinchem.2015.249508

38. Mokhtari A, Borna C, Gilje P, et al. A 1-h Combination Algorithm Allows Fast Rule-Out and Rule-In of Major Adverse Cardiac Events. *J Am Coll Cardiol* 2016;67(13):1531-40. doi: 10.1016/j.jacc.2016.01.059

39. Mueller C, Giannitsis E, Christ M, et al. Multicenter Evaluation of a 0-Hour/1-Hour Algorithm in the Diagnosis of Myocardial Infarction With High-Sensitivity Cardiac Troponin T. *Ann Emerg Med* 2016;68(1):76-87 e4. doi: 10.1016/j.annemergmed.2015.11.013

40. Neumann JT, Sorensen NA, Schwemer T, et al. Diagnosis of Myocardial Infarction Using a High-Sensitivity Troponin I 1-Hour Algorithm. *JAMA Cardiol* 2016;1(4):397-404. doi: 10.1001/jamacardio.2016.0695

For peer review only - http://bmjopen.bmj.com/site/about/guidelines.xhtml

41. Parsonage WA, Mueller C, Greenslade JH, et al. Validation of NICE diagnostic guidance for rule out of myocardial infarction using high-sensitivity troponin tests. *Heart* 2016;102(16):1279-86. doi: 10.1136/heartjnl-2016-309270

42. Pickering JW, Greenslade JH, Cullen L, et al. Assessment of the European Society of Cardiology 0-Hour/1-Hour Algorithm to Rule-Out and Rule-In Acute Myocardial Infarction. *Circulation* 2016;134(20):1532-41. doi: 10.1161/CIRCULATIONAHA.116.022677

43. Pickering JW, Greenslade JH, Cullen L, et al. Validation of presentation and 3 h high-sensitivity troponin to rule-in and rule-out acute myocardial infarction. *Heart* 2016;102(16):1270-8. doi: 10.1136/heartjnl-2015-308505

44. Wildi K, Nelles B, Twerenbold R, et al. Safety and efficacy of the 0 h/3 h protocol for rapid rule out of myocardial infarction. *Am Heart J* 2016;181:16-25. doi: 10.1016/j.ahj.2016.07.013

45. Boeddinghaus J, Nestelberger T, Twerenbold R, et al. Direct Comparison of 4 Very Early Rule-Out Strategies for Acute Myocardial Infarction Using High-Sensitivity Cardiac Troponin I. *Circulation* 2017;135(17):1597-611. doi: 10.1161/CIRCULATIONAHA.116.025661

46. Chapman AR, Anand A, Boeddinghaus J, et al. Comparison of the Efficacy and Safety of Early Rule-Out Pathways for Acute Myocardial Infarction. *Circulation* 2017;135(17):1586-96. doi: 10.1161/CIRCULATIONAHA.116.025021

47. Lindahl B, Jernberg T, Badertscher P, et al. An algorithm for rule-in and rule-out of acute myocardial infarction using a novel troponin I assay. *Heart* 2017;103(2):125-31. doi: 10.1136/heartjnl-2016-309951

48. McRae AD, Innes G, Graham M, et al. Comparative Evaluation of 2-Hour Rapid Diagnostic Algorithms for Acute Myocardial Infarction Using High-Sensitivity Cardiac Troponin T. *Can J Cardiol* 2017;33(8):1006-12. doi: 10.1016/j.cjca.2017.04.010

49. Mokhtari A, Lindahl B, Schiopu A, et al. A 0-Hour/1-Hour Protocol for Safe, Early Discharge of Chest Pain Patients. *Acad Emerg Med* 2017;24(8):983-92. doi: 10.1111/acem.13224

50. Neumann JT, Sorensen NA, Ojeda F, et al. Early diagnosis of acute myocardial infarction using high-sensitivity troponin I. *PLoS One* 2017;12(3):e0174288. doi: 10.1371/journal.pone.0174288

51. Sandoval Y, Smith SW, Thordsen SE, et al. Diagnostic Performance of High Sensitivity Compared with Contemporary Cardiac Troponin I for the Diagnosis

For peer review only - http://bmjopen.bmj.com/site/about/guidelines.xhtml

of Acute Myocardial Infarction. *Clin Chem* 2017;63(10):1594-604. doi: 10.1373/clinchem.2017.272930

52. Schofer N, Brunner F, Schlüter M, et al. Gender-specific diagnostic performance of a new highsensitivity cardiac troponin I assay for detection of acute myocardial infarction. *Eur Heart J* 2017;6(1):60-68. doi: 10.1177/2048872615626660
53. Shiozaki M, Inoue K, Suwa S, et al. Utility of the 0-hour/1-hour high-sensitivity cardiac troponin T algorithm in Asian patients with suspected non-ST elevation myocardial infarction. *Int J Cardiol* 2017;249:32-35. doi: 10.1016/j.ijcard.2017.09.009
54. Wildi K, Cullen L, Twerenbold R, et al. Direct Comparison of 2 Rule-Out Strategies for Acute Myocardial Infarction: 2-h Accelerated Diagnostic Protocol vs 2-h Algorithm. *Clin Chem* 2017;63(7):1227-36. doi: 10.1373/clinchem.2016.268359
55. Peacock WF, Baumann BM, Bruton D, et al. Efficacy of High-Sensitivity Troponin T in Identifying Very-Low-Risk Patients With Possible Acute Coronary Syndrome. *JAMA Cardiol* 2018;3(2):104-11. doi: 10.1001/jamacardio.2017.4625
56. Boeddinghaus J, Nestelberger T, Twerenbold R, et al. Impact of age on the performance of the ESC 0/1h-algorithms for early diagnosis of myocardial infarction. *Eur Heart J* 2018;39(42):3780-94. doi: 10.1093/eurheartj/ehy514
57. Boeddinghaus J, Twerenbold R, Nestelberger T, et al. Clinical Validation of a Novel High-Sensitivity Cardiac Troponin I Assay for Early Diagnosis of Acute Myocardial Infarction. *Clin Chem* 2018;64(9):1347-60. doi: 10.1373/clinchem.2018.286906
58. Borna C, Kollberg K, Larsson D, et al. The objective CORE score allows early rule out in acute chest pain patients. *Scand Cardiovasc J* 2018;52(6):308-14. doi: 10.1080/14017431.2018.1546891
59. Chapman AR, Hesse K, Andrews J, et al. High-Sensitivity Cardiac Troponin I and Clinical Risk Scores in Patients With Suspected Acute Coronary Syndrome. *Circulation* 2018;138(16):1654-65. doi: 10.1161/CIRCULATIONAHA.118.036426
60. Greenslade JH, Carlton EW, Van Hise C, et al. Diagnostic Accuracy of a New High-Sensitivity Troponin I Assay and Five Accelerated Diagnostic Pathways for Ruling Out Acute Myocardial Infarction and Acute Coronary Syndrome. *Ann Emerg Med* 2018;71(4):439-51 e3. doi: 10.1016/j.annemergmed.2017.10.030

For peer review only - http://bmjopen.bmj.com/site/about/guidelines.xhtml

61. Gunsolus I, Sandoval Y, Smith SW, et al. Renal Dysfunction Influences the Diagnostic and Prognostic Performance of High-Sensitivity Cardiac Troponin I. *J Am Soc Nephrol* 2018;29(2):636-43. doi: 10.1681/ASN.2017030341
62. Miller-Hodges E, Anand A, Shah ASV, et al. High-Sensitivity Cardiac Troponin and the Risk Stratification of Patients With Renal Impairment Presenting With Suspected Acute Coronary Syndrome. *Circulation* 2018;137(5):425-35. doi: 10.1161/CIRCULATIONAHA.117.030320
63. Nowak RM, Gandolfo CM, Jacobsen G, et al. Ultrarapid Rule-out for Acute Myocardial Infarction Using the Generation 5 Cardiac Troponin T Assay: Results From the REACTION-US Study. *Ann Emerg Med* 2018;72(6):654-64. doi: 10.1016/j.annemergmed.2018.06.021
64. Twerenbold R, Badertscher P, Boeddinghaus J, et al. 0/1-Hour Triage Algorithm for Myocardial Infarction in Patients With Renal Dysfunction. *Circulation* 2018;137(5):436-51. doi: 10.1161/CIRCULATIONAHA.117.028901
65. Twerenbold R, Neumann JT, Sorensen NA, et al. Prospective Validation of the 0/1-h Algorithm for Early Diagnosis of Myocardial Infarction. *J Am Coll Cardiol* 2018;72(6):620-32. doi: 10.1016/j.jacc.2018.05.040
66. Amann M, Gaiser F, Schwenk SI, et al. Evaluation of a 1-hour troponin algorithm for diagnosing myocardial infarction in high-risk patients admitted to a chest pain unit: the prospective FAST-MI cohort study. *BMJ Open* 2019;9(11):e032124. doi: 10.1136/bmjopen-2019-032124
67. Boeddinghaus J, Twerenbold R, Nestelberger T, et al. Clinical Use of a New High-Sensitivity Cardiac Troponin I Assay in Patients with Suspected Myocardial Infarction. *Clin Chem* 2019;65(11):1426-36. doi: 10.1373/clinchem.2019.304725
68. Cappellini F, Falbo R, Saltafossi D, et al. Development of an algorithm for ruling-out non-ST elevation myocardial infarction in the emergency department using high sensitivity troponin T assay. *Clin Chim Acta* 2019;495:1-7. doi: 10.1016/j.cca.2019.03.1625
69. Chapman AR, Fujisawa T, Lee KK, et al. Novel high-sensitivity cardiac troponin I assay in patients with suspected acute coronary syndrome. *Heart* 2019;105(8):616-22. doi: 10.1136/heartjnl-2018-314093
70. Chew DP, Lambrakis K, Blyth A, et al. A Randomized Trial of a 1-Hour Troponin T Protocol in Suspected Acute Coronary Syndromes: The Rapid Assessment of Possible Acute Coronary Syndrome in the Emergency Department With High-

For peer review only - http://bmjopen.bmj.com/site/about/guidelines.xhtml

Sensitivity Troponin T Study (RAPID-TnT). *Circulation* 2019;140(19):1543-56. doi: 10.1161/CIRCULATIONAHA.119.042891
71. Lin Z, Lim SH, Chua SJT, et al. High-sensitivity troponin T and long-term adverse cardiac events among patients presenting with suspected acute coronary syndrome in Singapore. *Singapore Med J* 2019;60(8):418-26. doi: 10.11622/smedj.2019013
72. Mueller-Hennessen M, Lindahl B, Giannitsis E, et al. Combined testing of copeptin and high-sensitivity cardiac troponin T at presentation in comparison to other algorithms for rapid rule-out of acute myocardial infarction. *Int J Cardiol* 2019;276:261-67. doi: 10.1016/j.ijcard.2018.10.084
73. Nestelberger T, Boeddinghaus J, Wussler D, et al. Predicting Major Adverse Events in Patients With Acute Myocardial Infarction. *J Am Coll Cardiol* 2019;74(7):842-54. doi: 10.1016/j.jacc.2019.06.025
74. Nestelberger T, Boeddinghaus J, Greenslade J, et al. Two-Hour Algorithm for Rapid Triage of Suspected Acute Myocardial Infarction Using a High-Sensitivity Cardiac Troponin I Assay. *Clin Chem* 2019;65(11):1437-47. doi: 10.1373/clinchem.2019.305193
75. Neumann JT, Sorensen NA, Rubsamen N, et al. Evaluation of a new ultra-sensitivity troponin I assay in patients with suspected myocardial infarction. *Int J Cardiol* 2019;283:35-40. doi: 10.1016/j.ijcard.2018.12.001
76. Sorensen NA, Neumann JT, Ojeda F, et al. Diagnostic Evaluation of a High-Sensitivity Troponin I Point-of-Care Assay. *Clin Chem* 2019;65(12):1592-601. doi: 10.1373/clinchem.2019.307405
77. Twerenbold R, Costabel JP, Nestelberger T, et al. Outcome of Applying the ESC 0/1-hour Algorithm in Patients With Suspected Myocardial Infarction. *J Am Coll Cardiol* 2019;74(4):483-94. doi: 10.1016/j.jacc.2019.05.046
78. Wildi K, Boeddinghaus J, Nestelberger T, et al. Comparison of fourteen rule-out strategies for acute myocardial infarction. *Int J Cardiol* 2019;283:41-47. doi: 10.1016/j.ijcard.2018.11.140
79. Andruchow JE, Boyne T, Seiden-Long I, et al. Prospective comparative evaluation of the European Society of Cardiology (ESC) 1-hour and a 2-hour rapid diagnostic algorithm for myocardial infarction using high-sensitivity troponin-T. *CJEM* 2020;22(5):712-20. doi: 10.1017/cem.2020.349
80. Boeddinghaus J, Nestelberger T, Koechlin L, et al. Early Diagnosis of Myocardial Infarction With Point-of-Care High-Sensitivity Cardiac Troponin I. *J Am Coll Cardiol* 2020;75(10):1111-24. doi: 10.1016/j.jacc.2019.12.065

For peer review only - http://bmjopen.bmj.com/site/about/guidelines.xhtml

81. Kavsak PA, Mondoux SE, Ma J, et al. Comparison of two biomarker only algorithms for early risk stratification in patients with suspected acute coronary syndrome. *Int J Cardiol* 2020;319:140-43. doi: 10.1016/j.ijcard.2020.06.066
82. Kavsak PA, Mondoux SE, Sherbino J, et al. Clinical evaluation of Ortho Clinical Diagnostics high-sensitivity cardiac Troponin I assay in patients with symptoms suggestive of acute coronary syndrome. *Clin Biochem* 2020;80:48-51. doi: 10.1016/j.clinbiochem.2020.04.003
83. Kim JW, Kim H, Yun YM, et al. Absolute Change in High-Sensitivity Cardiac Troponin I at Three Hours After Presentation is Useful for Diagnosing Acute Myocardial Infarction in the Emergency Department. *Ann Lab Med* 2020;40(6):474-80. doi: 10.3343/alm.2020.40.6.474
84. Nowak RM, Christenson RH, Jacobsen G, et al. Performance of Novel High-Sensitivity Cardiac Troponin I Assays for 0/1-Hour and 0/2- to 3-Hour Evaluations for Acute Myocardial Infarction: Results From the HIGH-US Study. *Ann Emerg Med* 2020;76(1):1-13. doi: 10.1016/j.annemergmed.2019.12.008
85. Peacock WF, Christenson R, Diercks DB, et al. Myocardial Infarction Can Be Safely Excluded by High-sensitivity Troponin I Testing 3 Hours After Emergency Department Presentation. *Acad Emerg Med* 2020 doi: 10.1111/acem.13922
86. Sandoval Y, Smith SW, Schulz K, et al. Comparison of 0/3-Hour Rapid Rule-Out Strategies Using High-Sensitivity Cardiac Troponin I in a US Emergency Department. *Circ Cardiovasc Qual Outcomes* 2020;13(7):e006565. doi: 10.1161/CIRCOUTCOMES.120.006565
87. Shiozaki M, Inoue K, Suwa S, et al. Implementing the European Society of Cardiology 0-h/1-h algorithm in patients presenting very early after chest pain. *Int J Cardiol* 2020;320:1-6. doi: 10.1016/j.ijcard.2020.07.037
88. Allen BR, Christenson RH, Cohen SA, et al. Diagnostic Performance of High-Sensitivity Cardiac Troponin T Strategies and Clinical Variables in a Multisite US Cohort. *Circulation* 2021;143(17):1659-72. doi: 10.1161/CIRCULATIONAHA.120.049298
89. Andersen CF, Bang C, Lauridsen KG, et al. External validation of a high-sensitive troponin I algorithm for rapid evaluation of acute myocardial infarction in a Danish cohort. *Eur Heart J Acute Cardiovasc Care* 2021;10(9):1056-64. doi: 10.1093/ehjacc/zuab062
90. Dongxu C, Yannan Z, Yilin Y, et al. Evaluation of the 0 h/1 h high-sensitivity cardiac troponin T algorithm in diagnosis of non-ST-segment elevation

For peer review only - http://bmjopen.bmj.com/site/about/guidelines.xhtml

myocardial infarction (NSTEMI) in Han population. *Clin Chem Lab Med* 2021;59(4):757-64. doi: 10.1515/cclm-2020-0367

91. Dupuy AM, Badiou S, Montagnon V, et al. Analytical assessment and performance of the 0/3h algorithm with novel high sensitivity cardiac troponin I. *Clin Chim Acta* 2021;519:111-17. doi: 10.1016/j.cca.2021.04.003

92. Kavsak PA, Hewitt MK, Mondoux SE, et al. Diagnostic Performance of Serial High-Sensitivity Cardiac Troponin Measurements in the Emergency Setting. *J Cardiovasc Dev Dis* 2021;8(8) doi: 10.3390/jcdd8080097

93. Koechlin L, Boeddinghaus J, Nestelberger T, et al. Performance of the ESC 0/2h-algorithm using high-sensitivity cardiac troponin I in the early diagnosis of myocardial infarction. *Am Heart J* 2021;242:132-37. doi: 10.1016/j.ahj.2021.08.008

94. Lopez-Ayala P, Nestelberger T, Boeddinghaus J, et al. Novel Criteria for the Observe-Zone of the ESC 0/1h-hs-cTnT Algorithm. *Circulation* 2021;144(10):773-87. doi: 10.1161/CIRCULATIONAHA.120.052982

95. McCord J, Hana A, Cook B, et al. The role of cardiac testing with the 0/1-hour high-sensitivity cardiac troponin algorithm evaluating for acute myocardial infarction. *Am Heart J* 2021;233:68-77. doi: 10.1016/j.ahj.2020.12.015

96. Olsson P, Khoshnood A, Mokhtari A, et al. Glucose and high-sensitivity troponin T predict a low risk of major adverse cardiac events in emergency department chest pain patients. *Scand Cardiovasc J* 2021;55(6):354-61. doi: 10.1080/14017431.2021.1987512

97. Ruangsomboon O, Thirawattanasoot N, Chakorn T, et al. The utility of the 1-hour high-sensitivity cardiac troponin T algorithm compared with and combined with five early rule-out scores in high-acuity chest pain emergency patients. *Int J Cardiol* 2021;322:23-28. doi: 10.1016/j.ijcard.2020.08.099

98. Sörensen NA, Neumann JT, Ojeda F, et al. Differences in measurement of high-sensitivity troponin in an on-demand and batch-wise setting. *European Heart Journal Acute Cardiovascular Care* 2021;10(3):302-09. doi: 10.1177/2048872620924198

99. Sorensen NA, Gossling A, Neumann JT, et al. Diagnostic Validation of a High-Sensitivity Cardiac Troponin I Assay. *Clin Chem* 2021;67(9):1230-39. doi: 10.1093/clinchem/hvab070

100. Steiro OT, Tjora HL, Langorgen J, et al. Clinical risk scores identify more patients at risk for cardiovascular events within 30 days as compared to standard ACS

For peer review only - http://bmjopen.bmj.com/site/about/guidelines.xhtml

risk criteria: the WESTCOR study. *Eur Heart J Acute Cardiovasc Care* 2021;10(3):287-301. doi: 10.1093/ehjacc/zuaa016

101. Wildi K, Lopez-Ayala P, Koechlin L, et al. Validation of the Novel European Society of Cardiology 0/2-hour Algorithm Using Hs-cTnT in the Early Diagnosis of Myocardial Infarction. *Am J Cardiol* 2021;154:128-30. doi: 10.1016/j.amjcard.2021.06.003

102. Nestelberger T, Boeddinghaus J, Gimenez MR, et al. Direct comparison of high-sensitivity cardiac troponin T and I in the early differentiation of type 1 vs. type 2 myocardial infarction. *Eur Heart J Acute Cardiovasc Care* 2022;11(1):62-74. doi: 10.1093/ehjacc/zuab039

103. Cannon CP, Battler A, Brindis RG, et al. American College of Cardiology key data elements and definitions for measuring the clinical management and outcomes of patients with acute coronary syndromes: A report of the American College of Cardiology Task Force on Clinical Data Standards (Acute Coronary Syndromes Writing Committee). *Journal of the American College of Cardiology* 2001;38(7):2114-30. doi: 10.1016/s0735-1097(01)01702-8

104. Anderson JL, Adams CD, Antman EM, et al. ACC/AHA 2007 guidelines for the management of patients with unstable angina/non-ST-Elevation myocardial infarction: a report of the American College of Cardiology/American Heart Association Task Force on Practice Guidelines (Writing Committee to Revise the 2002 Guidelines for the Management of Patients With Unstable Angina/Non-ST-Elevation Myocardial Infarction) developed in collaboration with the American College of Emergency Physicians, the Society for Cardiovascular Angiography and Interventions, and the Society of Thoracic Surgeons endorsed by the American Association of Cardiovascular and Pulmonary Rehabilitation and the Society for Academic Emergency Medicine. *J Am Coll Cardiol* 2007;50(7):e1-e157. doi: 10.1016/j.jacc.2007.02.013

105. Apple FS, Wu AH, Jaffe AS. European Society of Cardiology and American College of Cardiology guidelines for redefinition of myocardial infarction: how to use existing assays clinically and for clinical trials. *Am Heart J* 2002;144(6):981-6. doi: 10.1067/mhj.2002.124048

106. Anderson JL, Adams CD, Antman EM, et al. 2012 ACCF/AHA focused update incorporated into the ACCF/AHA 2007 guidelines for the management of patients with unstable angina/non-ST-elevation myocardial infarction: a report of the American College of Cardiology Foundation/American Heart Association

For peer review only - http://bmjopen.bmj.com/site/about/guidelines.xhtml

Task Force on Practice Guidelines. *J Am Coll Cardiol* 2013;61(23):e179-347. doi: 10.1016/j.jacc.2013.01.014
107. Hamm CW, Bassand JP, Agewall S, et al. ESC Guidelines for the management of acute coronary syndromes in patients presenting without persistent ST-segment elevation: The Task Force for the management of acute coronary syndromes (ACS) in patients presenting without persistent ST-segment elevation of the European Society of Cardiology (ESC). *Eur Heart J* 2011;32(23):2999-3054. doi: 10.1093/eurheartj/ehr236
108. Arslan M, Dedic A, Boersma E, et al. Serial high-sensitivity cardiac troponin T measurements to rule out acute myocardial infarction and a single high baseline measurement for swift rule-in: A systematic review and meta-analysis. *Eur Heart J Acute Cardiovasc Care* 2020;9(1):14-22. doi: 10.1177/2048872618819421
109. Chiang CH, Chiang CH, Lee GH, et al. Safety and efficacy of the European Society of Cardiology 0/1-hour algorithm for diagnosis of myocardial infarction: systematic review and meta-analysis. *Heart* 2020;106(13):985-91. doi: 10.1136/heartjnl-2019-316343
110. Apple FS, Collinson PO, Kavsak PA, et al. Getting Cardiac Troponin Right: Appraisal of the 2020 European Society of Cardiology Guidelines for the Management of Acute Coronary Syndromes in Patients Presenting without Persistent ST-Segment Elevation by the International Federation of Clinical Chemistry and Laboratory Medicine Committee on Clinical Applications of Cardiac Bio-Markers. *Clin Chem* 2021;67(5):730-35. doi: 10.1093/clinchem/hvaa337
111. Giannitsis E, Blankenberg S, Christenson RH, et al. Critical appraisal of the 2020 ESC guideline recommendations on diagnosis and risk assessment in patients with suspected non-ST-segment elevation acute coronary syndrome. *Clin Res Cardiol* 2021;110(9):1353-68. doi: 10.1007/s00392-021-01821-2
112. Six A, Backus B, Kelder J. Chest pain in the emergency room: value of the HEART score. *Neth Heart J* 2008;16(6):191-6. doi: 10.1007/BF03086144
113. Than M, Flaws D, Sanders S, et al. Development and validation of the Emergency Department Assessment of Chest pain Score and 2 h accelerated diagnostic protocol. *Emerg Med Australas* 2014;26(1):34-44. doi: 10.1111/1742-6723.12164
114. Fox CS, Muntner P, Chen AY, et al. Use of evidence-based therapies in short-term outcomes of ST-segment elevation myocardial infarction and non-ST-segment

For peer review only - http://bmjopen.bmj.com/site/about/guidelines.xhtml

elevation myocardial infarction in patients with chronic kidney disease: a report from the National Cardiovascular Data Acute Coronary Treatment and Intervention Outcomes Network registry. *Circulation* 2010;121(3):357-65. doi: 10.1161/CIRCULATIONAHA.109.865352
115. Bhatnagar P, Wickramasinghe K, Williams J, et al. The epidemiology of cardiovascular disease in the UK 2014. *Heart* 2015;101(15):1182-9. doi: 10.1136/heartjnl-2015-307516
116. Apple FS, Sandoval Y, Jaffe AS, et al. Cardiac Troponin Assays: Guide to Understanding Analytical Characteristics and Their Impact on Clinical Care. *Clin Chem* 2017;63(1):73-81. doi: 10.1373/clinchem.2016.255109
117. Neumann JT, Twerenbold R, Ojeda F, et al. Application of High-Sensitivity Troponin in Suspected Myocardial Infarction. *N Engl J Med* 2019;380(26):2529-40. doi: 10.1056/NEJMoa1803377
118. Wu AHB, Christenson RH, Greene DN, et al. Clinical Laboratory Practice Recommendations for the Use of Cardiac Troponin in Acute Coronary Syndrome: Expert Opinion from the Academy of the American Association for Clinical Chemistry and the Task Force on Clinical Applications of Cardiac Bio-Markers of the International Federation of Clinical Chemistry and Laboratory Medicine. Clin Chem 2018; 64(4):645-655. doi: 10.1373/clinchem.2017.277186
119. Twerenbold R, Wildi K, Jaeger C, et al. Optimal Cutoff Levels of More Sensitive Cardiac Troponin Assays for the Early Diagnosis of Myocardial Infarction in Patients With Renal Dysfunction. *Circulation* 2015;131(23):2041-50. doi: 10.1161/CIRCULATIONAHA.114.014245
120. Dubin R, Li Y, He J, et al. Predictors of high sensitivity cardiac troponin T in chronic kidney disease patients: a cross-sectional study in the chronic renal insufficiency cohort (CRIC). *BMC Nephrology* 2013;14(229). doi: 10.1186/1471-2369-14-229
121. Clerico A, Ripoli A, Zaninotto M, et al. Head-to-head comparison of plasma cTnI concentration values measured with three high-sensitivity methods in a large Italian population of healthy volunteers and patients admitted to emergency department with acute coronary syndrome: A multi-center study. Clin Chim Acta 2019;496:25-34. doi: 10.1016/j.cca.2019.06.012

For peer review only - http://bmjopen.bmj.com/site/about/guidelines.xhtml

Figure legends

Figure 1. PRISMA Flow Diagram

Figure 2. Cumulative number of study participants assessed by specific testing protocols and assays for serial high-sensitivity cardiac troponin measurements*

* The numbers of cumulative participants are based on simple summations of all participants assessed in the relevant studies. These numbers may be overestimated due to overlapping inclusions of patients from same cohorts and/or institutions.

For peer review only

For peer review only - http://bmjopen.bmj.com/site/about/guidelines.xhtml

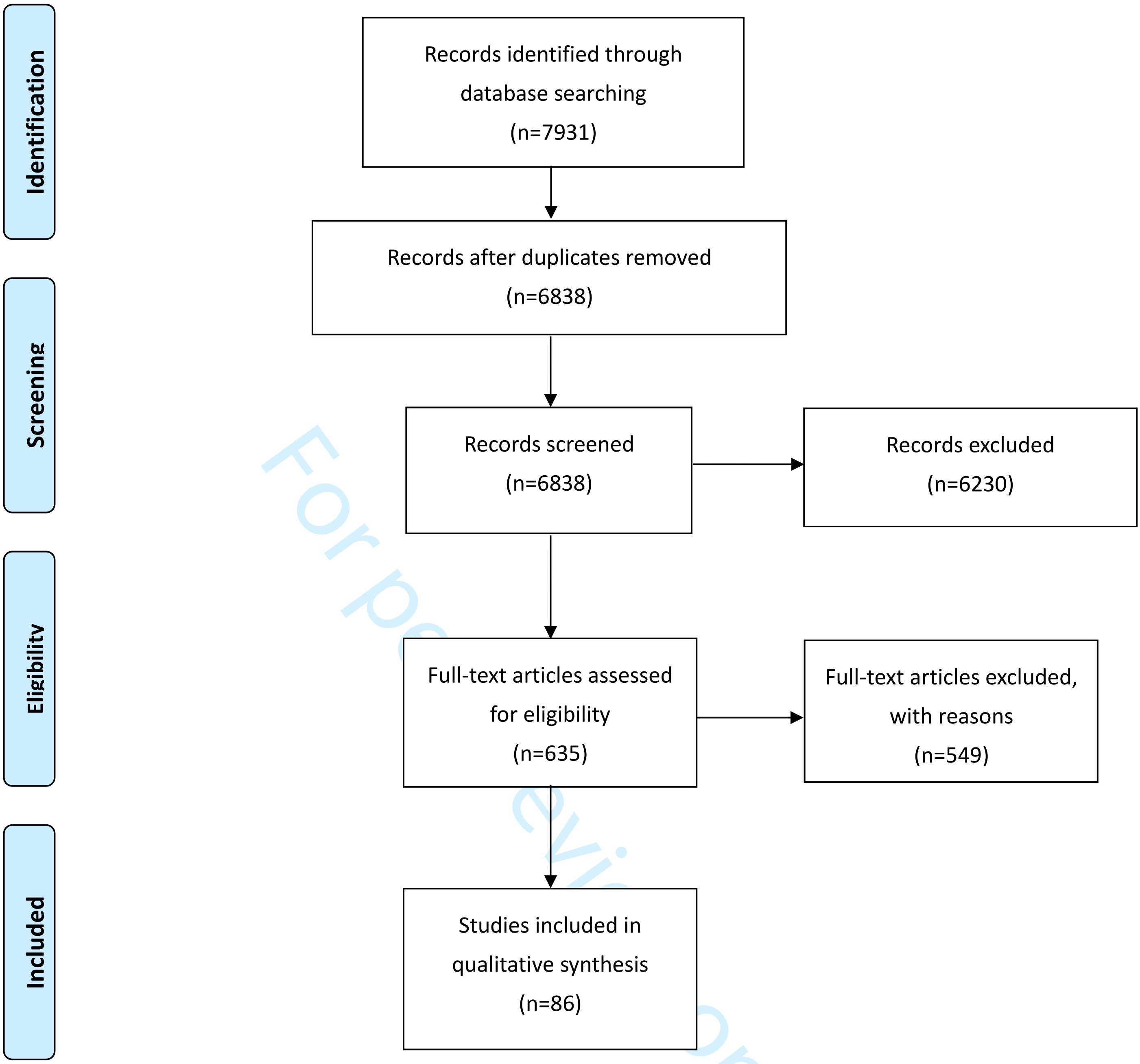

For peer review only

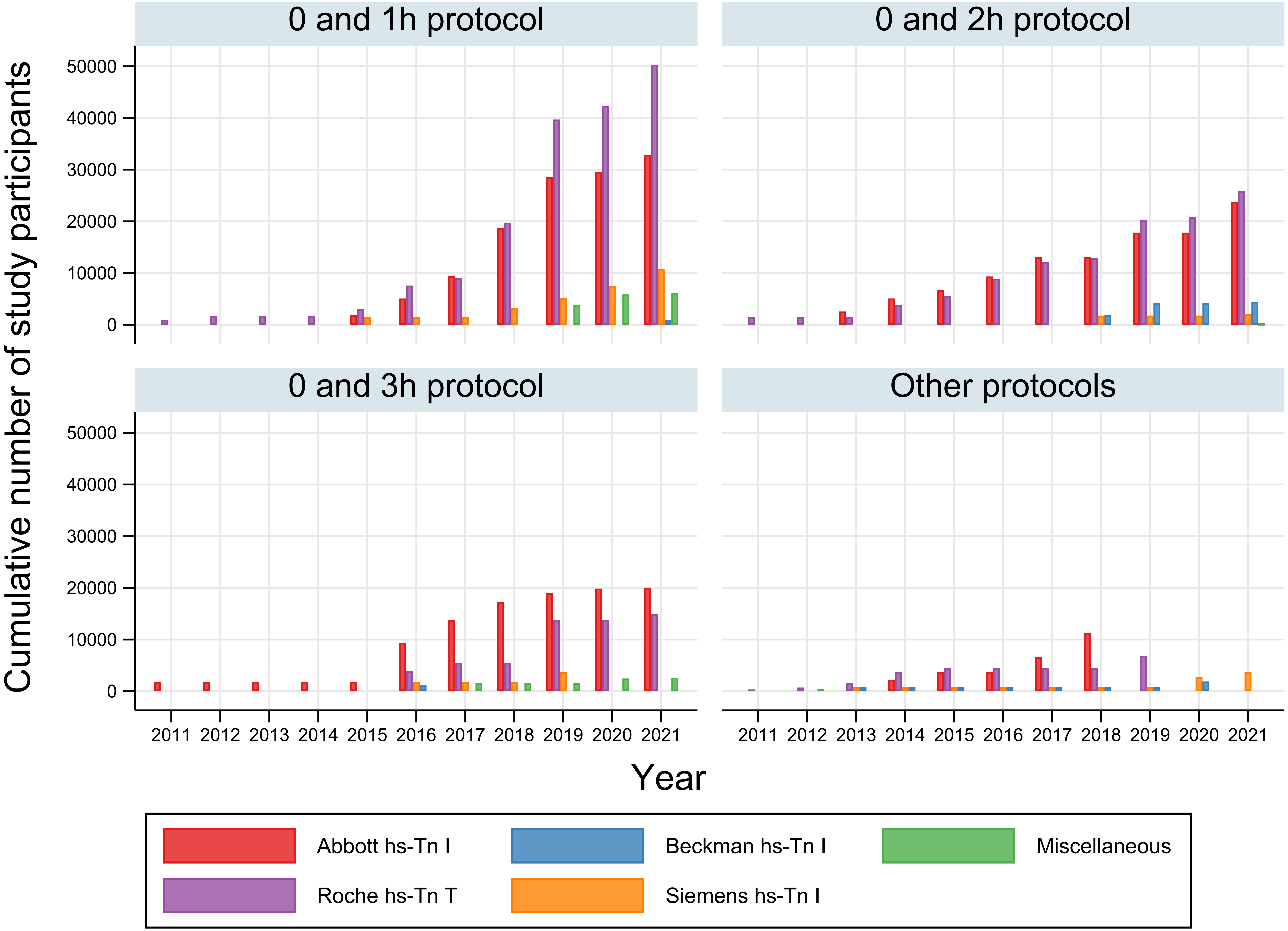
0 and 1h protocol
0 and 2h protocol
0 and 3h protocol
Other protocols
Cumulative number of study participants
50000
40000
30000
20000
10000
0
2011 2012 2013 2014 2015 2016 2017 2018 2019 2020 2021
Year
Abbott hs-Tn I
Beckman hs-Tn I
Miscellaneous
Roche hs-Tn T
Siemens hs-Tn I

Supplementary material

Search Strategy

MEDLINE by Ovid

1 exp Chest Pain/
2 (chest adj2 pain*).ti,ab.
3 chest discomfort.ti,ab.
4 exp Acute Coronary Syndrome/
5 acute coronary syndrome*.ti,ab.
6 ACS.ti,ab.
7 exp Angina, Unstable/
8 unstable angina.ti,ab.
9 Myocardial Infarction/di
10 myocardial infarction.ti,ab.
11 heart attack.ti,ab.
12 or/1-11
13 cardiac troponin*.ti,ab.
14 high* sensitiv* troponin*.ti,ab.
15 hs-ctn*.ti,ab.
16 elecsys.ti,ab.
17 architectstat.ti,ab.
18 architect stat.ti,ab.
19 accutni*.ti,ab.
20 accutnl*.ti,ab.
21 centaur ultra.ti,ab.
22 HISCL.ti,ab.
23 vidas.ti,ab.
24 lumipulse.ti,ab.
25 serial troponin.ti,ab.
26 contemporary troponin.ti,ab.
27 heart adp.ti,ab.
28 (EDACS and troponin).ti,ab.
29 (Adapt adp and troponin).ti,ab.
30 (GRACE and troponin).ti,ab.
31 (TIMI and troponin).ti,ab
32 or/13-31

For peer review only

33 exp Emergency Service, Hospital/
34 (emergency adj (room* or department*)).ti,ab.
35 (ER or ED).ti,ab.
36 (presenting or presented).ti,ab.
37 presentation.ti,ab
38 (admission or admitted).ti,ab.
39 chest pain unit.ti,ab.
40 or/33-39
41 12 and 32 and 40

Embase by Elsevier

1 exp Chest Pain/
2 (chest adj2 pain*).ti,ab.
3 chest discomfort.ti,ab.
4 exp Acute Coronary Syndrome/
5 acute coronary syndrome*.ti,ab.
6 ACS.ti,ab.
7 exp Angina, Unstable/
8 unstable angina.ti,ab.
9 Myocardial Infarction/di
10 myocardial infarction.ti,ab.
11 heart attack.ti,ab.
12 or/1-11
13 cardiac troponin*.ti,ab.
14 high* sensitiv* troponin*.ti,ab.
15 hs-ctn*.ti,ab.
16 elecsys.ti,ab.
17 architectstat.ti,ab.
18 architect stat.ti,ab.
19 accutni*.ti,ab.
20 accutnl*.ti,ab.
21 centaur ultra.ti,ab.
22 HISCL.ti,ab.
23 vidas.ti,ab.
24 lumipulse.ti,ab.
25 serial troponin.ti,ab.

For peer review only

26 or/13-25
27 exp Emergency Service, Hospital/
28 (emergency adj (room* or department*)).ti,ab.
29 (ER or ED).ti,ab.
30 (presenting or presented).ti,ab.
31 presentation.ti,ab.
32 (admission or admitted).ti,ab.
33 chest pain unit.ti,ab.
34 or/27-33
35 12 and 26 and 34
36 limit 35 to yr="2006 -Current"

Cochrane Database of Systematic Reviews and CENTRAL database by the Cochrane Library

#1 MeSH descriptor: [Chest Pain] this term only
#2 (chest next pain):ti,ab
#3 (chest next discomfort):ti,ab
#4 MeSH descriptor: [Acute Coronary Syndrome] explode all trees
#5 ACS:ti,ab
#6 (acute next coronary next syndrome):ti,ab
#7 MeSH descriptor: [Coronary Artery Disease] explode all trees
#8 (coronary next artery next disease):ti,ab
#9 MeSH descriptor: [Angina, Unstable] explode all trees
#10 (unstable next angina):ti,ab
#11 MeSH descriptor: [Myocardial Infarction] explode all trees
#12 (myocardial next infarction):ti,ab
#13 (heart next attack):ti,ab
#14 #1 or #2 or #3 or #4 or #5 or #6 or #7 or #8 or #9 or #10 or #11 or #12 or #13
#15 MeSH descriptor: [Troponin] explode all trees
#16 troponin:ti,ab
#17 hs-ctn*:ti,ab
#18 #15 or #16 or #17
#19 MeSH descriptor: [Emergency Service, Hospital] explode all trees
#20 (emergency next (room* or department*)):ti,ab
#21 (ER or ED):ti,ab
#22 (presenting or presented):ti,ab

For peer review only

#23 presentation:ti,ab

#24 (admission or admitted):ti,ab

#25 "chest pain unit":ti,ab

#26 #19 or #20 or #21 or #22 or #23 or #24 or #25

#27 #14 and #18 and #26

Science Citation Index

#1 TITLE: (high* sensitiv* troponin)

#2 TITLE: ("serial troponin" or "serial cardiac troponin")

#3 TOPIC: ("myocardial infarction*" or "heart attack*" or "heart failure")

#4 TOPIC: (diagnosi* or detect* or identif*)

#5 #2 OR #1

#6 #5 AND #4 AND #3

For peer review only

Appendix Table 1. Included studies reporting the diagnostic accuracy of AMI and 30-day clinical outcomes.

Author/publish year[*]	Number of patients[†]	Age[‡], year	Renal dysfunction[‖]	Study name or region	Enrolment years	Need of ECG[¶]	Clinical outcomes[**]
**Troponin I assays manufactured by Abbott**							
0 and 1h protocol							
Gimenez 2015[1]	1811/2377 (76)	62 (49-62)	Excl	APACE	2006-2012		30-day
Pickering 2016a[2]	2222 (ND)	60 (±14)	Incl	ADAPT, ADAPT-ADP, EDACS-ADP, RING	ADAPT: 2007-2011; ADAPT-ADP: 2010-2012; EDACS: 2013-2014; RING: ND	Excl	Dx MI
Neumann 2016[3]	1040 (ND)	65 (52-75)	Incl	BACC	2013-2014		Dx MI
Boeddinghaus 2017[4]	2828/3642 (78)	62 (49-74)	Excl	APACE	2006-2015		Dx MI, 30-day
Neumann 2017[5]	1516 (ND)	65 (51-75)	Incl	BACC	2013-2016		Dx MI
Twerenbold 2018a[6]	2504<br>445 /4168 (71)	58 (47-70)[‡‡]<br>79 (73-84)[§§]	Excl<br>Incl	APACE<br>APACE	ND<br>ND		Dx MI, 30-day<br>Dx MI, 30-day
Twerenbold 2018b[7]	3500/5631 (62)	62[‖‖]	P-Incl	APACE, BACC	2006-2016		Dx MI, 30-day
Boeddinghaus 2018a[8]	4269/5136 (83)	62 (49-74)	P-Incl	APACE, BACC, TRAPID-AMI	2006-2015		Dx MI, 30-day
Wildi 2019[9]	2945/4169 (71)	62 (49-74)	Excl	APACE	2006-2015	P-need	Dx MI, 30-day
Nestelberger 2019a[10]	2828/3980 (71)	62 (49-74)	Excl	APACE	2006-2015		Dx MI, 30-day
Boeddinghaus 2019[11]	937/1288 (73)	60 (48-74)	Excl	APACE	2011-2015		Dx MI
Neuman 2019[12]	1534 (ND)	65[‖‖]	Incl	BACC	ND		Dx MI
Sörensen 2019[13]	1279 (ND)	65[‖‖]	Incl	BACC	2015-2016		Dx MI
Boeddinghaus 2020[14]	962/1328 (72)	60 (47-73)	Excl	APACE	2011-2014		Dx MI, 30-day
Sörensen 2021a[15]	937 (ND)	65 (52-75)	Incl	BACC	2013-2016		Dx MI
Sörensen 2021b[16]	1800 (ND)	65 (52-75)	Incl	BACC	2013-2014		Dx MI
Nestelberger 2022[17]	862/6021 (14)	61 (49-74)	Excl	APACE	2006-2018		Dx MI
0 and 2h protocol							
Cullen 2013[18]	2544 (ND)	61[‖‖]	P-Incl	APACE, ADAPT	APACE: 2006-2008; ADAPT: 2007-2011	P-need	30-day
Aldous 2014[19]	962 (ND)	66 (56-76)	Incl	ASPECT, ADAPT	2007-2010		Dx MI, 30-day
Cullen 2014[20]	1959/1571 (80)	60[‖‖]	Incl	Australia/New Zealand	2007-2011		Dx MI
Eggers 2015[21]	1624 (ND)	61 (±15)	Incl	ADAPT	2007-2011		Dx MI
Boeddinghaus 2016[22]	2629 (ND)	62[‖‖]	P-Incl	APACE, ADAPT	APACE: 2006-2012; ADAPT: 2007-2011	P-need	Dx MI, 30-day
Wildi 2017[23]	2525/4417 (57)	62[‖‖]	P-Incl	APACE, ADAPT	APACE: 2006-2013; ADAPT: 2007-2011	P-need	Dx MI, 30-day
Lindahl 2017[24]	1197 (ND)	64[‖‖]	P-Incl	APACE, FASTEST	APACE: 2008-2014, FASTEST: 2013-2014		Dx MI, 30-day
Wildi 2019[9]	2357/4169 (57)	62 (50-74)	Excl	APACE	2006-2015	P-need	Dx MI, 30-day
Nestelberger 2019b[25]	2411/2979 (81)	56[‖‖]	P-Incl	APACE, ADAPT, IMPACT	APACE: ND, ADAPT: 2007-2011, IMPACT: 2011-2014		Dx MI, 30-day
Kavsak 2021[26]	935/1366 (68)	69[‖‖]	Incl	ROMI-3	2013		30-day
Koechlin 2021[27]	1863/4587 (41)	61 (50-74)	Excl	APACE	ND		Dx MI
Nestelberger 2022[17]	660/6021 (11)	61 (49-74)	Excl	APACE	2006-2018		Dx MI
0 and 3h protocol							
Keller 2011[††28]	1818 (ND)	61 (±14)	Incl	Germany	2007-2008		Dx MI
Neumann 2016[3]	1040 (ND)	65 (52-75)	Incl	BACC	2013-2014		Dx MI
Wildi 2016[29]	2226/2922 (76)	62 (49-74)	Excl	APACE	2006-2013		Dx MI
Parsonage 2016[30]	3128/3580 (87)	61[‖‖]	P-Incl	APACE, Australia/New Zealand	Australia/New Zealand: 2008-2011; APACE: 2006-2013		30-day
Pickering 2016b[31]	1172 (ND)	60 (±14)	Incl	ADAPT, ADAPT-ADP, EDACS-ADP, RING	ADAPT: 2007-2011; ADAPT-ADP: 2010-2012; EDACS: 2013-2014; RING: ND	Excl	Dx MI
Neumann 2017[5]	1516 (ND)	65 (51-75)	Incl	BACC	2013-2016		Dx MI
Chapman 2017[32]	1218/1292 (94)	62 (±14)	Incl	High-STEACS	2013-2015		Dx MI, 30-day
Sandoval 2017[33]	1068 (ND)	ND	Incl	UTROPIA	2014		Dx MI
Chapman 2018[34]	1935/1951 (99)	62 (±14)	Incl	High-STEACS	2013-2017	P-need	Dx MI, 30-day
Gunsolus 2018[35]	1555 (ND)	58 (±15)	Incl	UTROPIA	2014		Dx MI
Wildi 2019[9]	1742/4169 (42)	63 (50-76)	Excl	APACE	2006-2015	P-need	Dx MI, 30-day
Kim 2020[36]	281/362 (78)	59[‖‖]	ND	Korea, Seoul	2015-2016		Dx MI
Sandoval 2020[37]	582 (ND)	ND	Incl	UTROPIA	2014		30-day
Dupuy 2021[38]	ND	73 (±17)	Incl	Montpellier, France	ND		Dx MI
Miscellaneous							
Gimenez 2014[39]	2226/2956 (75)	62 (50-75)	Excl	APACE	2006-2012		Dx MI
Pickering 2015[40]	368 (ND)	61 (±13)	Incl	Australia/New Zealand	2010-2012	P-need	Dx MI
Chapman 2017[32]	1218/1292 (94)	62 (±14)	Incl	High-STEACS	2013-2015		Dx MI, 30-day

Author/publish year*	Number of patients†	Age‡, year	Renal dysfunction‖	Study name or region	Enrolment years	Need of ECG¶	Clinical outcomes**
Sandoval 2017[33]	1631 (ND)	ND	Incl	UTROPIA	2014		Dx MI, 30-day
Miller-Hodges 2018[41]	2193/4789 (46)	64 (±16)	Incl	UK	2013-2014		Dx MI, 30-day
**Troponin T assays manufactured by Roche**							
0 and 1h protocol							
Reichlin 2011[42]	836/1197 (70)	64 (51-76)	Excl	APACE	2007-2009		Dx MI
Reichlin 2012[43]	872/1197 (73)	64 (51-75)	Excl	APACE	2006-2009		Dx MI, 30-day
Reichlin 2015a[44]	1320/1656 (80)	60 (49-73)	Excl	APACE	2007-2009		Dx MI, 30-day
Mokhtari 2016[45]	1038 (ND)	61 (± 18)	Incl	Sweden	2013-2014		Dx MI, 30-day
Mokhtari 2017[46]	1020/1167 (87)	61 (±17)	Incl	Sweden	2013-2014	Excl	30-day
Mueller 2016[††47]	1282 (ND)	62 (50-74)	Excl	TRAPID-AMI	2011-2013		Dx MI, 30-day
Pickering 2016a[2]	2222 (ND)	60 (± 14)	Incl	ADAPT, ADAPT-ADP, EDACS-ADP, RING	ADAPT: 2007-2011; ADAPT-ADP: 2010-2012; EDACS: 2013-2014; RING: ND	Excl	Dx MI
Shiozaki 2017[48]	413 (ND)	72 (59-81)	Incl	Japan	2014-2017		Dx MI, 30-day
Twerenbold 2018a[6]	2767/4168 (78)<br>487	58 (47-70) ‡‡<br>79 (73-84) §§	Excl<br>Excl	APACE<br>APACE	ND<br>ND		DX MI, 30-day<br>DX MI, 30-day
Twerenbold 2018b[7]	4368/5631 (78)	62 (50-74)	P-Incl	APACE, BACC	2006-2016		DX MI, 30-day
Boeddinghaus 2018a[8]	5890/6744 (87)	61 (49-74)	P-Incl	APACE, BACC, TRAPID-AMI	2006-2015		Dx MI, 30-day
Mueller-Hennessen2019[49]	922/1282 (72)	62 (49-74)	Excl	TRAPID-AMI	ND		Dx MI, 30-day
Wildi 2019[9]	3020/4169 (72)	60 (49-73)	Excl	APACE	2006-2015	P-need	Dx MI, 30-day
Chew 2019[50]	1646/1646 (100)	59 (49-69)	Excl	RAPID-TnT	2015-2019	Excl	30-day
Nestelberger 2019a[10]	3123/3980 (78)	61 (49-74)	Excl	APACE	2006-2015		Dx MI, 30-day
Twerenbold 2019[51]	2296 (ND)	60 (49-71)	Incl	Switzerland, Argentina	2015-2017		30-day
Amann 2019[52]	1317 (ND)	72[‖‖]	Incl	FAST-MI	2015-2016		Dx MI, 30-day
Andruchow 2020[53]	350/608 (58)	60 (52-72)	Excl	Canada, Alberta	2014-2016		Dx MI, 30-day
Cappellini 2019[54]	7176/14170 (51)	73 (59-82)	Incl	Italy, Desio	2011-2016		Dx MI
Shiozaki 2020[55]	1074/1183 (91)	72 (60-80)	Excl	Japan, Taiwan	2014-2019		30-day
Boeddinghaus 2019[11]	937/1288 (73)	60 (48-74)	Excl	APACE	2011-2015		Dx MI
Boeddinghaus 2020[14]	962/1328 (72)	ND	Excl	APACE	2011-2014		Dx MI, 30-day
Andruchow 2020[53]	350/2911 (12)	61[‖‖]	P-Inc	APPROACH	2014-2016		Dx MI, 30-day
Allen 2021[56]	1462/1583 (92)	58 (±13)	Inc	STOP CP	2017-2018		30-day
Dongxu 2021[57]	577/600 (96)	66 (59-75)	P-Inc	China	2017-2018		Dx MI, 30-day
Lopez-Ayala 2021[58]	2076/6684 (31)	63	P-Inc	APACE	APACE: 2006-2018		Dx MI, 30-day
Olsson 2021[59]	1031(ND)	61 (± 18)	Incl	Sweden	2013-2014		30-day
Ruangsomboon 2021[60]	350 (ND)	66 (±15)	P-Incl	Thailand	ND	P-need	Dx MI, 30-day
Sörensen 2021a[98]	565 (ND)	65 (52-75)	Incl	BACC	2013-2016		Dx MI
Nestelberger 2022[15]	892/6021 (15)	61 (49-74)	Excl	APACE	2006-2018		Dx MI
0 and 2h protocol							
Aldous 2011a[61]	939 (ND)	65 (56-76)	Incl	New Zealand	2007-2009		Dx MI
Reichlin 2011[42]	590/1197 (49)	64 (51-76)	Excl	APACE	2007-2009		Dx MI
Cullen 2014[20]	1571/1959 (80)	60[‖‖]	Incl	Australia/New Zealand	2007-2011		Dx MI
Parsonage 2014[††62]	764 (ND)	55 (±15)	Incl	Australia	2008-2011		Dx MI, 30-day
Reichlin 2015b[63]	1665/2788 (60)	59[‖‖]	P-incl	APACE, Australia	APACE: 2006-2011, Australia: 2008-2011		30-day
Parsonage 2016[30]	3374/3580 (94)	61[‖‖]	P-Incl	APACE, Australia/New Zealand	Australia/New Zealand: 2008-2011; APACE: 2006-2013		30-day
Wildi 2017[23]	2525/4417 (57)	62[‖‖]	P-Incl	APACE, ADAPT	APACE: 2006-2013; ADAPT: 2007-2011	P-need	Dx MI, 30-day
McRae 2017[64]	722/7131 (10)	59 (50-69)	Excl	APPROACH	2013		Dx MI, 30-day
Borna 2018[65]	751/1165 (64)	64 (50-74)	Incl	Sweden	2013-2014	P-need	30-day
Wildi 2019[9]	2473/4169 (59)	62 (50-74)	Excl	APACE	2006-2015	P-need	Dx MI, 30-day
Lin 2019[66]	2444/2474 (99)	55 (47-64)	Excl	Singapore	2010-2013		30-day
Nestelberger 2019b[25]	2411/2979 (81)	56[‖‖]	P-Incl	APACE, ADAPT, IMPACT	APACE: ND, ADAPT: 2007-2011, IMPACT: 2011-2014		Dx MI, 30-day
Andruchow 2020[53]	550/608 (90)	60 (52-72)	Excl	Canada, Alberta	2014-2016		Dx MI, 30-day
Koechlin 2021[27]	2227/4587 (49)	61 (50-74)	Excl	APACE	ND		Dx MI
Wildi 2021[67]	2127 (ND)	61 (49-73)	Excl	APACE	ND		Dx MI
Nestelberger 2022[17]	691/6021 (11)	61 (49-74)	Excl	APACE	2006-2018		Dx MI
0 and 3h protocol							
Pickering 2016b[31]	1098 (ND)	60 (±14)	Incl	ADAPT, ADAPT-ADP, EDACS-ADP, RING	ADAPT: 2007-2011; ADAPT-ADP: 2010-2012; EDACS:	Excl	Dx MI

For peer review only

Author/publish year[*]	Number of patients[†]	Age[‡], year	Renal dysfunction[‖]	Study name or region	Enrolment years	Need of ECG[¶]	Clinical outcomes[**]
					2011-2014; RING: ND		
Wildi 2016[29]	2727/2922 (93)	62 (49-74)	Excl	APACE	2006-2013		Dx MI
Peacock 2018[††68]	1679 (ND)	55 (47-64)	Incl	USA	2011-2015		Dx MI
Wildi 2019[9]	1908/4169 (46)	63 (50-76)	Excl	APACE	2006-2015	P-need	Dx MI, 30-day
Cappellini 2019[54]	7176/14170 (51)	73 (59-82)	Incl	Italy, Desio	2011-2016		Dx MI
Dupuy 2021[38]	160 (ND)	73 (±17)	Incl	Montpellier, France	ND		Dx MI
Steiro 2021[69]	932/984 (95)	63 (52-74)	Incl	WESTCOR	2015-2017	P-need	30-day
Miscellaneous							
Aldous 2011b[††70]	332 (ND)	64 (53-74)	Incl	New Zealand	2006-2007		Dx MI
Aldous 2012[††71]	385 (ND)	65 (56-76)	Incl	New Zealand	2007-2010		Dx MI
Irfan 2013[72]	830 (ND)	64 (51-75)	Excl	APACE	2006-2009		Dx MI
Gimenez 2014[39]	2226/2956 (75)	62 (50-75)	Excl	APACE	2006-2012		Dx MI
Biener 2015[73]	658/721 (91)	ND	Incl	Germany	ND		Dx MI
Nowak 2018[74]	569/1699 (33)	55 (49-63)	Incl	REACTION-US	2013-2015		Dx MI, 30-day
Lin 2019[66]	2444/2474 (99)	55 (47-64)	Excl	Singapore	2010-2013		Dx MI
**Troponin I assays manufactured by Siemens**							
0 and 1h protocol							
Jaeger 2016[75]	1500/1883 (80)	62 (49-74)	Excl	APACE	ND		Dx MI, 30-day
Boeddinghaus 2018b[76]	1347/1876 (72)	62 (49-75)	Excl	APACE	2006-2013		30-day
Chapman 2019[77]	1951/406 (21)	62 (±14)	Incl	High-STEACS	2013-2017	P-need	30-day
Nowak 2020[78]	2063/2470 (84)	56 (48-65)	Incl	HIGH-US	2015-2016		Dx MI, 30-day
Andersen 2021[79]	1003/1479 (68)	64 (52-74)	P-Incl	RACING-MI	2016-2019		Dx MI
Sörensen 2021b[16]	1800 (ND)	65 (52-75)	Incl	BACC	2013-2014		Dx MI
Nestelberger 2022[17]	418/6021 (7)	61 (49-74)	Excl	APACE	2006-2018		Dx MI
0 and 2h protocol							
Boeddinghaus 2018b[76]	1033/1876 (55)	62 (49-75)	Excl	APACE	2006-2013		30-day
Nestelberger 2022[17]	313/6021 (5)	61 (49-74)	Excl	APACE	2006-2018		Dx MI
0 and 3h protocol							
Wildi 2016[29]	1809/2922 (62)	62 (49-74)	Excl	APACE	2006-2013		Dx MI
Chapman 2019[77]	1920/1951 (98)	62 (±14)	Incl	High-STEACS	2013-2017	P-need	30-day
Sörensen 2021b[16]	1800 (ND)	65 (52-75)	Incl	BACC	2013-2014		Dx MI
Miscellaneous							
Irfan 2013[72]	830 (ND)	64 (51-75)	Excl	APACE	2006-2009		Dx MI
Nowak 2020[78]	1863/2470 (75)	56 (48-65)	Incl	HIGH-US	2015-2016		Dx MI, 30-day
Andersen 2021[79]	1003/1479 (68)	64 (52-74)	P-Incl	RACING-MI	2016-2019		Dx MI
**Troponin I assays manufactured by Beckman**							
0 and 1h protocol							
McCord 2021[80]	552/569 (97)	56 (±11)	Incl	REACTION-US	2013-2015		Dx MI
Nestelberger 2022[17]	278/6021 (5)	61 (49-74)	Excl	APACE	2006-2018		Dx MI
0 and 2h protocol							
Greenslade 2018[81]	1811/2314 (78)	53 (±14)	Incl	ADAPT, IMPACT	ADAPT: 2008-2011; IMPACT: 2011-2014	P-need	30-day
Nestelbergr 2019b[25]	2411/2979 (81)	56[‖‖]	P-Incl	APACE, ADAPT, IMPACT	APACE: ND, ADAPT: 2007-2011, IMPACT: 2011-2014		Dx MI, 30-day
Nestelberger 2022[17]	217/6021 (4)	61 (49-74)	Excl	APACE	2006-2018		Dx MI
0 and 3h protocol							
Wildi 2016[44]	1110/2922 (38)	62 (49-74)	Excl	APACE	2006-2013		Dx MI
Peacock 2020[29]	1049/1929 (54)	56[‖‖]	P-Incl	USA	2012-2013		Dx MI
Miscellaneous							
Irfan 2013[72]	830 (ND)	64 (51-75)	Excl	APACE	2006-2009		Dx MI
Peacock 2020[82]	1049/1929 (54)	56[‖‖]	Excl	USA	2012-2013		Dx MI
**Other assays**							
0 and 1h protocol							
Boeddinghaus 2019[11] (VITROS)	1039/1288 (81)	60 (48-74)	Excl	APACE	2011-2015		Dx MI, 30-day
Neuman 2019[12] (Singulex Clarity)	1534 (ND)	65[‖‖]	Incl	BACC	ND		Dx MI
Sörensen 2019[13] (PATHFAST)	1279 (ND)	65[‖‖]	Incl	BACC	2015-2016		Dx MI
Boeddinghaus 2020[14] (Triage True)	1084/1328 (82)	60 (47-73)	Excl	APACE	2011-2014		Dx MI, 30-day
Kavsak 2020a[83] (VITROS)	906/1366 (66)	68[‖‖]	Incl	ROMI-3	2013-2017		Dx MI, 30-day
Nestelberger 2022[17] (Triage True)	204/6021 (3)	61 (49-74)	Excl	APACE	2006-2018		Dx MI
Nestelberger 2022[17] (VITROS)	160/6021 (3)	61 (49-74)	Excl	APACE	2006-2018		Dx MI
0 and 2h protocol							

For peer review only

Author/publish year*	Number of patients†	Age‡, year	Renal dysfunction‖	Study name or region	Enrolment years	Need of ECG¶	Clinical outcomes**
Nestelberger 2022[17] (Triage True)	204/6021 (3)	61 (49-74)	Excl	APACE	2006-2018		Dx MI
Nestelberger 2022[17] (VITROS)	160/6021 (3)	61 (49-74)	Excl	APACE	2006-2018		Dx MI
0 and 3h protocol							
Schofer 2017[84] (Singulex)	1560/1699 (92)	62 (49-74)	Incl	Germany	2007-2008		Dx MI
Kavsak 2020b[85] (VITROS)	906/1355 (66)	68‖‖	Incl	ROMI-3	2013-2017		Dx MI, 30-day
Dupuy 2021[38] (VITROS)	160	73 (±17)	Incl	Montpellier, France	ND		Dx MI
Miscellaneous							
Schreiber 2012[86] (Singulex)	465/486 (96)	67 (21-98)¶¶	Incl	USA	2005-2006		Dx MI, 30-day

ADAPT = 2-hour Accelerated Diagnostic Protocol to Assess Patients with Chest Pain Symptoms using Contemporary Troponins as the Only Biomarker; ADAPT-ADP = 2-hour Accelerated Diagnostic Protocol to Assess Patients with Chest Pain Symptoms using Contemporary Troponins as the Only Biomarker - Accelerated Diagnostic Pathway; APACE = Advantageous Predictors of Acute Coronary Syndromes Evaluation study; APPOACH = Alberta Provincial PRoject for Outcome Assessment in Coronary Heart Disease registry; ASPECT = Asia Pacific Evaluation of Chest pain Trial; BACC = Biomarkers in Acute Cardiac Care study; CKD = chronic kidney diseases; 30-day = 30-day clinical outcomes; Dx = diagnosis; EDACS-ADP = Emergency Department Assessment of Chest Pain Score - Accelerated Diagnostic Pathway; Excl = excluded; FASTEST = Fast Assessment of Thoracic pain in the Emergency department using high-Sensitive Troponins; High-STEACS = High-Sensitivity Troponin in the Evaluation of Patients With Acute Coronary Syndrome; IMPACT = Improved Assessment of Chest Pain Trial; Incl = included; IQR = interquartile range; MI = myocardial infarction; ND = no data; P-Incl = partially included; P-need = partially needed; RING = Reducing the Time Interval for Identifying New Guideline Defined Myocardial Infarction in Patients with Suspected Acute Coronary Syndrome; SD = standard deviation; TRAPID-AMI = High-sensitivity cardiac Troponin T assay for RAPID rule-out of AMI; USA = United States of America; UTROPIA = Use of Abbott High Sensitivity Troponin I Assay In Acute Coronary Syndromes, RAPID-TnT = Rapid Assessment of Possible ACS in the Emergency Department with High-Sensitivity Troponin T, ROMI-3 = Rule-out MI 3h.

*Underlined studies are representative reports with the largest sample size of unique study participants among reports based on (partially) overlapping cohorts.

†The number of analysed/enrolled patients; the number of enrolled patients is presented only if the relevant data were reported.

‡Median (interquartile range) or mean ± SD.

‖Renal dysfunction refers to terminal kidney disease that requires regular haemodialysis. “Partially included” indicates a mixed study that consisted of several cohorts including patients with or without renal dysfunction.

¶Need of ECG in the assessment of participant eligibility. “Partially needed” (shown as “P-need”) indicates that at least one of the participating study sites or included cohorts needed ECG findings for assessing the participant eligibility. “Excluded” (shown as “Excl”) indicates patients with ischemia on ECG were excluded.

**Studies that assessed the diagnostic accuracy of myocardial infarction and 30-day clinical outcomes are indicated as Dx MI and 30-day, respectively.

††A study that included patients suspected of having ST-elevation MI in addition to patients suspected of having non-ST elevation MI.

‡‡Normal renal function.

§§Renal dysfunction.

‖‖Weighted average.

For peer review only

¶¶Range.

For peer review only

Appendix Table 2. Excluded articles and reasons for exclusion.

Authors	Reasons
Agewall 2007[87]	Only patients admitted in the coronary care units
Ahmadi 2017[88]	Only patients with positive hs-cTn
Alcalai 2021[89]	Single, baseline hs-cTn only
Aldous 2011[90]	Hs-cTn assessed at baseline only
Aldous 2012a[91]	No accuracy data
Aldous 2012c[92]	No accuracy data
Alghamdi 2020[93]	Use of a non-hs-cTn
Al–Saleh 2014[94]	Review article
Alushi 2021[95]	Irrelevant clinical context
Anand 2021[96]	Irrelevant clinical context
Apple 2006[97]	Use of a non-hs-cTn
Apple 2009[98]	Use of a non-hs-cTn
Astley 2014[99]	Study protocol
Avest 2016[100]	Single, baseline hs-cTn only
Bahrmann 2012[101]	Irrelevant clinical context
Bahrmann 2013[102]	Irrelevant clinical context
Bahrmann 2016[103]	Irrelevant
Bali 2021[104]	Use of a non-hs-cTn
Ballocca 2017[105]	Patients with positive hs-cTn only
Bandstein 2014[106]	Single, baseline hs-cTn only
Bandstein 2016[107]	Irrelevant clinical context
Bandstein 2017[108]	Single, baseline hs-cTn only
Bellini 2021[109]	Irrelevant clinical context
Bhardwaj 2011[110]	Single, baseline hs-cTn only
Biener 2013[111]	Irrelevant clinical context
Birkhahn 2011[112]	No accuracy data
Bjurman 2013[113]	Only patients admitted in the coronary care units
Body 2011[114]	Single, baseline hs-cTn only
Body 2015a[115]	Single, baseline hs-cTn only
Body 2015b[116]	Single, baseline hs-cTn only
Body 2016[117]	Single, baseline hs-cTn only
Body 2020[118]	Single, baseline hs-cTn only
Boeckel 2015[119]	Use of a non-hs-cTn.
Boeddinghaus 2017[120]	Patients with positive hs-cTn only
Bonaca 2013[121]	Single, baseline hs-cTn only
Borna 2014[122]	Only patients admitted in the coronary care units

For peer review only

Borna 2016[123]	Only patients admitted in the hospitals
Bove 2017[124]	Only patients admitted in the hospitals
Braga 2011[125]	No accuracy data
Brichko 2018[126]	Patients with negative hs-cTn only
Bularga 2019 [127]	Single, baseline hs-cTn only
Caglar 2020[128]	Irrelevant clinical context
Carlton 2016[129]	Single, baseline hs-cTn only
Carlton 2016[130]	Irrelevant
Carlton 2018[131]	Irrelevant
Carlton 2020 [132]	Single, baseline hs-cTn only
Cavender 2017[133]	Irrelevant
Celik 2011[134]	Case series of patients with ACS
Chapman 2017[135]	Review article
Charpentier 2016 [136]	Guidelines
Chenevier–Gobeaux 2016[137]	Irrelevant clinical context
Chew 2016[138]	Irrelevant
Chew 2018[139]	Single, baseline hs-cTn only
Christ 2010[140]	Single, baseline hs-cTn only
Christ 2018[141]	Use of a non-hs-cTn
Christenson 2020[142]	No accuracy data
Chuang 2016[143]	Irrelevant clinical context
Clerico 2019[144]	Irrelevant clinical context
Colak 2017[145]	Article not obtainable
Collinson 2006a[146]	Single, baseline hs-cTn only
Collinson 2006b[147]	Single, baseline hs-cTn only
Collinson 2015[148]	No accuracy data
Conde 2013[149]	No accuracy data
Cook 2021[150]	Single, baseline hs-cTn only
Corsini 2015[151]	No accuracy data
Cramer 2007[152]	Use of a non-hs-cTn
Cullen 2013[153]	Use of a non-hs-cTn
Cullen 2016[154]	Irrelevant
Dadkhah 2017[155]	Irrelevant
Daly 2012[156]	Patients submitted to the catheter laboratory only
Davarani 2012[157]	Use of a non-hs-cTn
Dawson 2013[158]	Irrelevant clinical context
Diercks 2012[159]	Use of a non-hs-cTn
Druey 2015[160]	Use of a non-hs-cTn

For peer review only

Eggers 2011[161]	Use of a non-hs-cTn
Etaher 2021[162]	Irrelevant clinical context
Fan 2014[163]	No accuracy data
Fanaroff 2015[164]	Irrelevant
Freund 2011[165]	Single, baseline hs-cTn only
Frisoli 2017[166]	Irrelevant
Gassenmaier 2012[167]	Irrelevant clinical context
Giannitsis 2010[168]	Only patients admitted in the chest pain unit
Giannitsis 2010a[169]	Only patients with confirmed NSTE-ACS
Gimenez 2016[170]	Single, baseline hs-cTn only and no accuracy data
Gimenez 2020[171]	Irrelevant clinical context
Gimenez 2021[171]	Irrelevant clinical context
Giner–Caro 2013[172]	Letter
Goodman 2015[173]	Irrelevant clinical context
Gratzel 2015[174]	No accuracy data
Graven 2021[175]	Irrelevant clinical context
Greenslade 2018[176]	Single, baseline hs-cTn only
Grinstein 2015[177]	Only patients with NSTEMI
Haaf 2014[178]	Single, baseline hs-cTn only
Haider 2017[179]	Single, baseline hs-cTn only
Halim 2009[180]	Single, baseline hs-cTn only
Hall 2012[181]	No accuracy data
Hochholzer 2011[182]	Single, baseline hs-cTn only
Hochholzer 2014[183]	Patients submitted to the catheter laboratory only
Hochholzer 2016[184]	Guidelines
Hoeller 2013[185]	Single, baseline hs-cTn only
Hromadka 2012[186]	Irrelevant clinical context
Huang 2015[187]	Single, baseline hs-cTn only
Huang 2016[188]	Single, baseline hs-cTn only
Ilva 2009[189]	Single, baseline hs-cTn only
Invernizzi 2013[190]	Review article
Isiksacan 2017[191]	Single, baseline hs-cTn only
Iwasaki 2017[192]	Use of a non-hs-cTn
Jairam 2011[193]	Irrelevant clinical context
Johannessen 2020[194]	Irrelevant clinical context
Karady 2021[195]	Use of a non-hs-cTn
Karon 2018[196]	Irrelevant clinical context

For peer review only

Kavsak 2006[197]	Use of a non-hs-cTn
Kavsak 2009[198]	Single, baseline hs-cTn only
Kavsak 2012[199]	Single, baseline hs-cTn only
Kavsak 2017[200]	Single, baseline hs-cTn only
Kavsak 2018a[201]	Single, baseline hs-cTn only
Kavsak 2018b[202]	Single, baseline hs-cTn only
Kavsak 2020[203]	Single, baseline hs-cTn only
Kelly 2011[204]	Single, baseline hs-cTn only
Kelly 2014a[205]	Patients with negative hs-cTn only
Kelly 2014b[206]	Irrelevant
Kelly 2015[207]	Irrelevant
Kemper 2017[208]	Use of a non-hs-cTn
Khan 2021[209]	Irrelevant clinical cotext
Khoshnood 2020[210]	Single, baseline hs-cTn only
Kienbacher 2021[211]	No accuracy data
Kim 2013[212]	Irrelevant
Kimenai 2020[213]	Only patients admitted in the coronary care units
Kitamura 2013[214]	Single, baseline hs-cTn only
Kremneva 2016[215]	Use of a non-hs-cTn.
Krintus 2014[216]	Irrelevant
Kumar 2016[217]	Two-gate design (patients with MI and healthy subjects)
Lambrakis 2021[218]	Irrelevant clinical context
Lee 2010[219]	No accuracy data
Lehmacher 2020[220]	Irrelevant clinical context
Li 2015[221]	Meta-analysis.
Lim 2008[222]	Irrelevant
Lim 2020[194]	Single, baseline hs-cTn only
Lin 2012[223]	Review article.
Lindahl 2010[224]	Patients with ACS only
Love 2016[225]	Irrelevant clinical context
Lynn 2011[226]	Irrelevant clinical context
Maag 2015[227]	Irrelevant clinical context
MacRae 2006[228]	No accuracy data
Madsen 2015[229]	Only patients with positive hs-cTn
Mahler 2013[230]	Irrelevant
Marjot 2017[231]	Single, baseline hs-cTn only
Markota 2011[232]	Use of a non-hs-cTn.
Masotti 2018[233]	Irrelevant

For peer review only

May 2014[234]	Single, baseline hs-cTn only
McRae 2015[235]	Commentary
McRae 2017[64]	Single, baseline hs-cTn only
Melki 2011[236]	Only patients admitted in the coronary care units
Melki 2012[237]	Irrelevant
Melki 2015[238]	Irrelevant
Meune 2011[239]	Single, baseline hs-cTn only
Meune 2012[240]	Irrelevant
Miller 2008[241]	Irrelevant clinical context
Mills 2011[242]	Irrelevant
Mills 2012[243]	Single, baseline hs-cTn only
Moal 2007[244]	Irrelevant
Mohsen 2016[245]	No accuracy data
Mokhtari 2016[246]	Single, baseline hs-cTn only
Moliner 2020[247]	No accuracy data
Mueller 2012a[248]	Single, baseline hs-cTn only
Mueller 2012b[249]	Irrelevant clinical context
Mueller 2013[250]	Review article.
Mueller 2014[251]	Irrelevant clinical context
Mueller–Hennessen 2017[252]	Only patients with positive hs-cTn
Mungai 2020[253]	Irrelevant clinical context
Nacke 2014[254]	Only patients with ACS
Nadarajah 2021[255]	Clinical guidance
Narain 2008[256]	Only patients with ACS
Nejatian 2017[257]	Irrelevant clinical context
Nestelberger 2016[258]	No accuracy data
Nestelberger 2021a[259]	Irrelevant clinical context
Nestelberger 2021b[260]	Irrelevant clinical context
Neumann 2017[261]	Single, baseline hs-cTn only
Nilsson 2021[262]	Irrelevant clinical context
Normann 2012[263]	Irrelevant clinical context
Nowak 2021[264]	No accuracy data
Olivieri 2012[265]	No accuracy data
Omland 2011[266]	Review article
Osredkar 2021[267]	Use of non-hs-cTn
Palamalai 2013[268]	Use of non-hs-cTn
Paoloni 2010[269]	No accuracy data
Papendick 2017[270]	Study protocol

For peer review only

Parikh 2015[271]	Meta-analysis
Patsias 2017[272]	Only patients with positive hs-cTn
Peck 2016[273]	Irrelevant clinical context
Pickering 2018[274]	Irrelevant clinical context
Pickering 2020[275]	Irrelevant clinical context
Puelacher 2019[276]	Irrelevant clinical context
Rao 2012[277]	Irrelevant
Reichlin 2009[278]	Single, baseline hs-cTn only
Reichlin 2013[279]	Case series
Reiter 2011[280]	Review article
Ren 2021[281]	Single, baseline hs-cTn only
Renaud 2008[282]	Use of non-hs-cTn.
Roberts 2014[283]	Not a primary study
Roos 2017a[284]	Irrelevant clinical context
Roos 2017b[285]	Irrelevant clinical context
Rottger 2017[286]	Irrelevant clinical context
Rubini 2013[287]	No accuracy data
Rudolph 2012[288]	Irrelevant
Saad 2015a[289]	Single, baseline hs-cTn only
Saad 2015b[289]	Single, baseline hs-cTn only
Saenger 2010[290]	Conference abstract.
Saenger 2011[291]	Irrelevant
Sanchis 2012[292]	Only patients with positive hs-cTn
Sanchis 2014[293]	Irrelevant
Sanchis 2016[294]	Irrelevant
Sandoval 2015[295]	No accuracy data
Sandoval 2017[296]	Single, baseline hs-cTn only
Sandoval 2017[297]	Single, baseline hs-cTn only
Sandoval 2020[37]	Irrelevant clinical context
Santalo 2013[298]	Multi-gate design of case-mix
Schonemann–Lund 2015[299]	Irrelevant clinical context
Shah 2015[300]	Single, baseline hs-cTn only
Shah 2015 [301]	Single, baseline hs-cTn only
Shin 2020[302]	Irrelevant clinical context
Shortt 2015[303]	Single, baseline hs-cTn only
Shortt 2017[304]	Single, baseline hs-cTn only
Simpson 2014[305]	Irrelevant clinical context
Singer 2017[306]	Use of non-hs-cTn

For peer review only

Slagman 2017[307]	Use of non-hs-cTn
Sorensen 2017[308]	Irrelevant
Sorensen 2019[309]	Single, baseline hs-cTn only
Stengaard 2013[310]	Use of non-hs-cTn
Stopyra 2016[311]	Irrelevant
Stopyra 2020[312]	Irrelevant clinical context
Stoyanov 2020[313]	Irrelevant clinical context
Storrow 2015a[314]	Use of non-hs-cTn
Storrow 2015b[315]	Use of non-hs-cTn
Straface 2008[316]	Use of non-hs-cTn
Su 2015[317]	Irrelevant clinical context
Suh 2018[318]	Use of non-hs-cTn
Suzuki 2018[319]	Use of non-hs-cTn
Takakuwa 2009[320]	Use of non-hs-cTn
Tecson 2017[321]	Use of non-hs-cTn
Than 2011[322]	Use of non-hs-cTn
Than 2012[323]	Use of non-hs-cTn
Than 2014[324]	Irrelevant
Thelin 2015[325]	No accuracy data
Truong 2012[326]	Irrelevant
Twerenbold 2015[327]	Single, baseline hs-cTn only
Vafaie 2014[328]	Educational article
van der Laarse 2013[329]	Use of non-hs-cTn.
Van Hise 2018[330]	Irrelevant
Vasile 2017[331]	Review article
Vasudevan 2017[332]	Use of non-hs-cTn.
Venge 2009a[333]	Two–gate design study
Venge 2009b[334]	Irrelevant clinical context
Wang 2015a[335]	Only patients with COPD
Wang 2015b[336]	Two-gate design study (patients with AMI and healthy subjects)
Wassef 2015a[337]	Only patients admitted in the coronary care units
Wassef 2015b[337]	Only patients admitted in the Cardiology department
Wassie 2020[338]	Use of non-hs-cTn
Weber 2011[339]	Irrelevant clinical context
Wereski 2021[340]	Irrelevant clinical context
Westwood 2015[341]	Review article
Wildi 2013[342]	Patients with positive hs-cTn were excluded.
Willeit 2017[343]	Meta-analysis

For peer review only

Wolf 2014[344]	Patients with ACS only
Wong 2010[345]	Irrelevant clinical context
Yang 2021[346]	Irrelevant clinical context
Yean 2020[347]	Irrelevant clinical context
Yokoyama 2018[348]	Irrelevant clinical context
Zhao 2021[349]	Irrelevant clinical context

AMI = acute myocardial infarction; ACS = acute coronary syndrome; COPD = chronic obstructive pulmonary disease; hs–cTn = high sensitivity cardiac troponin, NSTE-ACS = non-ST-elevation acute coronary syndrome

For peer review only

Appendix Table 3. Institution and geographic characteristics of included study cohorts.

Cohort name		Europe		Australasia		North America		Asia	
(N of participants)	Institutions	City	Country	City	Country	City	Country	City	Country
ADAPT[323] (1635)***	Christchurch Hospital			Christchurch	New Zealand				
	Royal Brisbane and Women's Hospital			Brisbane	Australia				
ADAPT-ADP[324] (406)***	Christchurch Hospital			Christchurch	New Zealand				
APACE[350] (4368) ***	Hospital Clinic Barcelona	Barcelona	Spain						
	Hospital Clinic San Carlos	Madrid	Spain						
	Hospital del Mar	Barcelona	Spain						
	Kantonsspital Baselland, Standort Bruderholz	Bottmingen	Switzerland						
	Kantonsspital Baselland, Standort Liestal	Liestal	Switzerland						
	Kantonsspital Olten	Olten	Switzerland						
	Klinik St. Anna	Luzern	Switzerland						
	Masaryk University	Brno	Czech						
	Medical University of Silesia	Silesia	Poland						
	San Martino Hospital	San Martino	Italy						
	Spital Limmattal	Schlieren	Switzerland						
	University Hospital of Basal	Basel	Switzerland						
	University Hospital of Zurich	Zurich	Switzerland						
APPROACH[351] (722)	University of Calgary					Calgary	Canada		
	University of Alberta					Edomonton	Canada		

For peer review only

Cohort name (N of participants)	Institutions	Europe City	Europe Country	Australasia City	Australasia Country	North America City	North America Country	Asia City	Asia Country
ASPECT[322] (ND)	Alberta Health Services					Calgary	Canada		
	Apollo Hospital							Delhi	India
	Chang Gung Memorial Hospital							Taipei	Taiwan
	China Medl University Hospital							Taipei	Taiwan
	Christchurch Hospital			Christchurch	New Zealand				
	Far Eastern Memorial Hospital							Taipei	Taiwan
	Mackay Memorial Hospital							Taipei	Taiwan
	National Cardiovascular Centre Harapan Kita							Jakarta	Indonesia
	Peoples' Hospital							Beijing	China
	Queen Elizabeth Hospital							Hong Kong	China
	Ramathibodi Hospital							Bangkok	Thailand
	Royal Brisbane and Women's Hospital			Brisbane	Australia				
	Severance Hospital							Seoul	Korea
	Singapore General Hospital							Singapore	Singapore
	Xuanwu Hospital							Beijing	China
BACC[352] (3500)***	University Heart Center Hamburg	Hamburg	Germany						
EDACS-ADP[353] (128)***	Christchurch Hospital			Christchurch	New Zealand				

For peer review only

Cohort name		Europe		Australasia		North America		Asia	
(N of participants)	Institutions	City	Country	City	Country	City	Country	City	Country
FASTEST[24] (605)	Uppsala University	Uppsala	Sweden						
	Karolinska Institute	Huddinge	Sweden						
	Karolinska University Hospital	Stockholm	Sweden						
FAST-MI[52] (1317)	University Heart Center Freiburg	Bad Krozingen	Germany						
Hamburg[28] (1818)	Federal Armed Forces Hospital Koblenz	Hamburg	Germany						
	Johannes Gutenberg-University Mainz	Hamburg	Germany						
	University Hospital Hamburg Eppendorf	Hamburg	Germany						
High STEACS[354] (1886)	Royal Infirmary of Edinburgh	Edinburgh	UK						
HIGH-US [78] (2346)	Baylor College of Medicine					Houston	USA		
	Beth Israel Deaconess					Boston	USA		
	Brigham and Women's					Boston	USA		
	Carolinas Medical Center					Charlotte	USA		
	Case Medical Center					Cleveland	USA		
	Detroit Receiving Wayne State					Detroit	USA		
	Duke University					Durham	USA		
	Hackensack University					Hackensack	USA		
	Hennepin County Medical Center					Minneapolis	USA		
	Henry Ford Hospital					Detroit	USA		
	Indiana University					Indianapolis	USA		
	Mayo Clinic					Rochester	USA		

For peer review only

Cohort name		Europe		Australasia		North America		Asia	
(N of participants)	Institutions	City	Country	City	Country	City	Country	City	Country
	New York Methodist					Brooklyn	USA		
	Research & Development Institute, Van Nuys; Providence Tarzana Medical Center					Tarzana	USA		
	Sinai Grace Wayne State					Detroit	USA		
	St. Vincent Hospital					Worcester	USA		
	Stony Brooks					Stony Brooks	USA		
	Tampa General Hopital					Tampa	USA		
	The Miriam hospital					Providence	USA		
	Thomas Jefferson University					Philadelphia	USA		
	UC Davis					Davis	USA		
	UC San Francisco					San Francisco	USA<br>USA		
	University of Cincinnati					Cincinnati	USA		
	University of Maryland					Baltimore	USA		
	University of New Mexico					Albuquerque	USA		
	University of Texas Medical Branch					Galveston	USA		
	University of Virginia					Charlottesvill e	USA		
	Virginia Common Health University					Richmond	USA		
	Wake Forrest University					Winston-Salem	USA		
IMPACT[355] (ND)	Royal Brisbane and Women's Hospital			Brisbane	Australia				
Italy[54] (6403)	Desio Hospital	Desio	Italy						

For peer review only

Cohort name		Europe		Australasia		North America		Asia	
(N of participants)	Institutions	City	Country	City	Country	City	Country	City	Country
Juntendo[48] (1074)***	Juntendo University Hospital							Tokyo	Japan
RACING-MI[79] (1003)	Randers Regional Hospital	Randers	Denmark						
RAPID-TnT [50] (1646)	Flinders Medical Centre - Bedford Park			Adelaide	Australia				
	The Royal Adelaide Hospital -			Adelaide	Australia				
	Noarlunga Health Service - Noarlunga Centre			Adelaide	Australia				
	Lyell McEwin Hospital - Elizabeth Vale			Adelaide	Australia				
	The Queen Elizabeth Hospital -			Woodville	Australia				
REACTION-US[80] (552)	Henry Ford Hopital					Detroit	USA		
RING[356] (148)***	McMaster University					Hamilton	Canada		
ROMI-3[83] (935)***	McMaster University					Hamilton	Canada		
Shanghai[57] (577)	Zhongshan Hospital							Shanghai	China
Singapore[66] (2444)	Singapore General Hospital								Singapore
Skåne[45 65] (1038)***	Skåne University Hospital	Lund	Sweden						
STOP CP[56] (1642)	UC Davis					Sacramento	USA		

For peer review only

Cohort name		Europe		Australasia		North America		Asia	
(N of participants)	Institutions	City	Country	City	Country	City	Country	City	Country
	University of Florida					Gainesville	USA		
	University of Maryland					Baltimore	USA		
	Henry Ford Health System					Detroit	USA		
	Wake Forest University					Winston-Salem	USA		
	University of Utah					Salt Lake City	USA		
TRAPID-AMI[47] (1282)***	Central Manchester U niversity Hospital	Manchester	UK						
	Cliniques University Saint-Luc (UCLouvain)	Brussel	Belgium						
	Henry Ford Health System					Detroit	USA		
	Institut d'Investigacions Biomèdiques Sant Pau	Barcelona	Spain						
	Karolinska Institute	Huddinge	Sweden						
	Liverpool Hosp ital and University New South Wales			Liverpool	Australia				
	Nuremberg Paracelsus Medical University	Nuremberg	Germany						
	University Hospital of Basal	Basel	Switzerland						
	Heidelberg University Hospital	Heidelberg	Germany						
	University Maryland					Baltimore	USA		
	Uppsala University	Uppsala	Sweden						
US multicenter[68] (1679)	Charleston Area Medical Center					Charleston	USA		
	Cleveland Clinic Foundation					Cleveland	USA		

For peer review only

Cohort name		Europe		Australasia		North America		Asia	
(N of participants)	Institutions	City	Country	City	Country	City	Country	City	Country
	Cooper University Hospital					Camden	USA		
	Duke University					Durham	USA		
	Health Science Center of Houston Medical School					Houston	USA		
	Henry Ford Health System					Detroit	USA		
	Indianapolis University					Indianapolis	USA		
	Mayo Clinic					Rochester	USA		
	Minneapolis Heart Institute Foundation					Minneapolis	USA		
	Newton Wellesley Hospital					Newton	USA		
	Prince George's Hospital					Maryland	USA		
	San Francisco General Hospital					San Francisco	USA		
	South Shore Hospital					Weymouth	USA		
	Thomas Jefferson University Hospital					Philadelphia	USA		
	University North Carolina					Chapel Hill	USA		
UTROPIA[357] (1631)	Hennepin County Medical Center					Minneapolis	USA		
WESTCOR[69] (932)	Haukeland University Hospital	Bergen	Norway						

ADAPT = 2-hour Accelerated Diagnostic Protocol to Assess Patients with Chest Pain Symptoms using Contemporary Troponins as the Only Biomarker; ADAPT–ADP = 2-hour Accelerated Diagnostic Protocol to Assess Patients with Chest Pain Symptoms using Contemporary Troponins as the Only Biomarker - Accelerated Diagnostic Pathway; AMI = acute myocardial infarction; APACE = Advantageous Predictors of Acute Coronary Syndromes Evaluation study; APPOACH = Alberta Provincial PRoject for Outcome Assessment in Coronary Heart Disease registry; ASPECT = Asia Pacific Evaluation of Chest pain Trial; BACC = Biomarkers in Acute Cardiac Care study; EDACS–ADP = Emergency Department Assessment of Chest Pain Score – Accelerated Diagnostic Pathway; FASTEST = Fast ASsessment of Thoracic pain in the Emergency department using high-Sensitive Troponins; High-STEACS = High-Sensitivity Troponin in the Evaluation of Patients

For peer review only

With Acute Coronary Syndrome; IMPACT = Improved Assessment of Chest Pain Trial; IQR = interquartile range; RACING-MI= Rapid use of high-sensitive cardiac troponin I for ruling-in and ruling-out of acute myocardial infarction; REACTION-US= Rapid Evaluation of ACuTe Myocardial InfarctiON in the US; RING = Reducing the Time Interval for Identifying New Guideline Defined Myocardial Infarction in Patients with Suspected Acute Coronary Syndrome; TRAPID–AMI = High-sensitivity cardiac Troponin T assay for RAPID rule-out of AMI; UTROPIA = Use of Abbott High Sensitivity Troponin I Assay In Acute Coronary Syndromes

***The largest number represented when multiple publications were derived from the same study cohort.

For peer review only

Appendix Figure 1. Geographical locations of eligible studies*

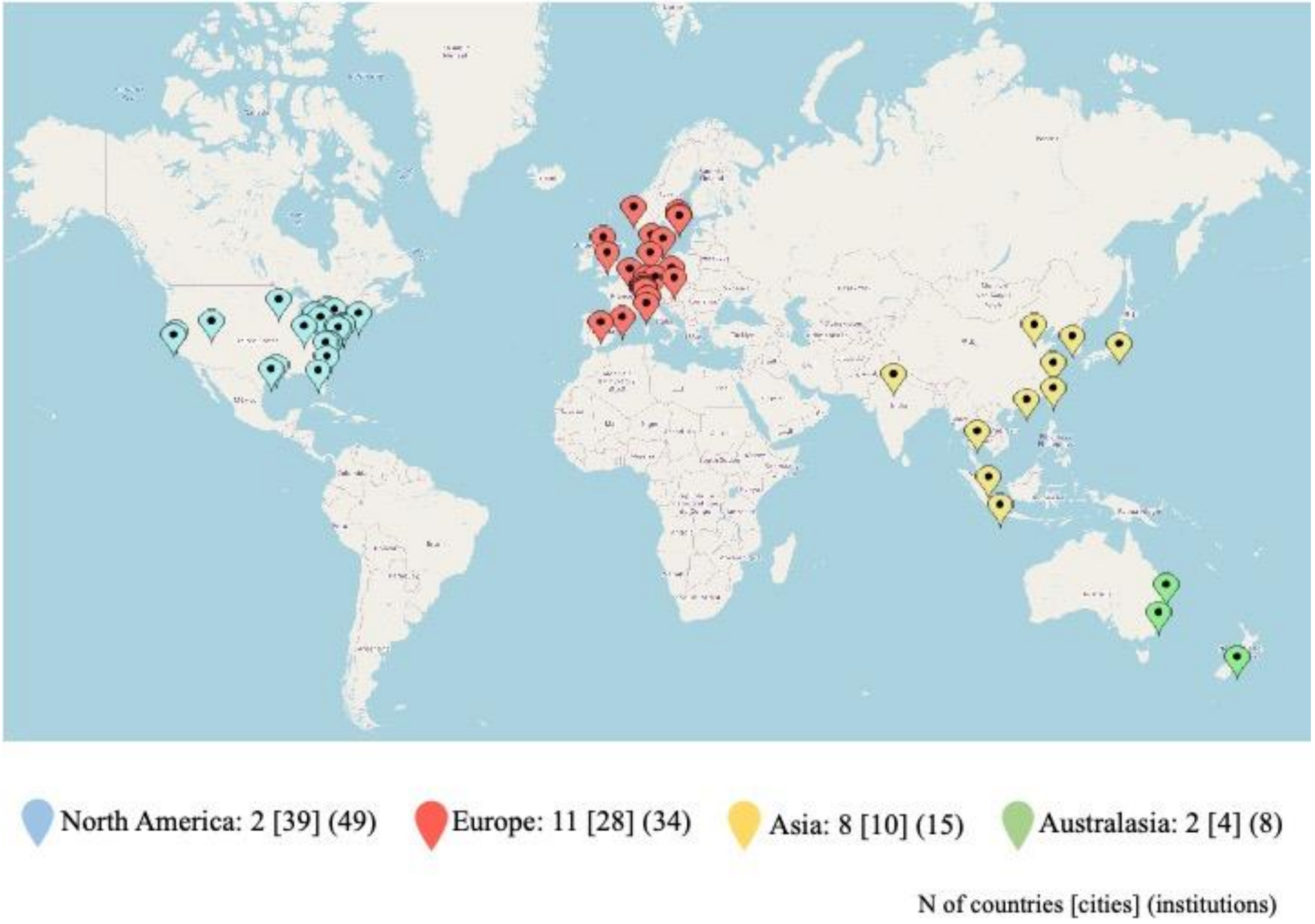

*Non-overlapping geographical regions (city as the unit) covered by the included research institution(s) are plotted. See Appendix Table 2 for details.

References

1. Rubini Gimenez M, Twerenbold R, Jaeger C, et al. One-hour rule-in and rule-out of acute myocardial infarction using high-sensitivity cardiac troponin I. *Am J Med* 2015;128(8):861-70 e4. doi: 10.1016/j.amjmed.2015.01.046

2. Pickering JW, Greenslade JH, Cullen L, et al. Assessment of the European Society of Cardiology 0-Hour/1-Hour Algorithm to Rule-Out and Rule-In Acute Myocardial Infarction. *Circulation* 2016;134(20):1532-41. doi: 10.1161/CIRCULATIONAHA.116.022677

3. Neumann JT, Sorensen NA, Schwemer T, et al. Diagnosis of Myocardial Infarction Using a High-Sensitivity Troponin I 1-Hour Algorithm. *JAMA Cardiol* 2016;1(4):397-404. doi: 10.1001/jamacardio.2016.0695

4. Boeddinghaus J, Nestelberger T, Twerenbold R, et al. Direct Comparison of 4 Very Early Rule-Out Strategies for Acute Myocardial Infarction Using High-Sensitivity Cardiac Troponin I. *Circulation* 2017;135(17):1597-611. doi: 10.1161/CIRCULATIONAHA.116.025661

5. Neumann JT, Sorensen NA, Ojeda F, et al. Early diagnosis of acute myocardial infarction using high-sensitivity troponin I. *PLoS One* 2017;12(3):e0174288. doi: 10.1371/journal.pone.0174288

6. Twerenbold R, Badertscher P, Boeddinghaus J, et al. 0/1-Hour Triage Algorithm for Myocardial Infarction in Patients With Renal Dysfunction. *Circulation* 2018;137(5):436-51. doi: 10.1161/CIRCULATIONAHA.117.028901

7. Twerenbold R, Neumann JT, Sorensen NA, et al. Prospective Validation of the 0/1-h Algorithm for Early Diagnosis of Myocardial Infarction. *J Am Coll Cardiol* 2018;72(6):620-32. doi: 10.1016/j.jacc.2018.05.040

8. Boeddinghaus J, Nestelberger T, Twerenbold R, et al. Impact of age on the performance of the ESC 0/1h-algorithms for early diagnosis of myocardial infarction. *Eur Heart J* 2018;39(42):3780-94. doi: 10.1093/eurheartj/ehy514

9. Wildi K, Boeddinghaus J, Nestelberger T, et al. Comparison of fourteen rule-out strategies for acute myocardial infarction. *Int J Cardiol* 2019;283:41-47. doi: 10.1016/j.ijcard.2018.11.140

10. Nestelberger T, Boeddinghaus J, Wussler D, et al. Predicting Major Adverse Events in Patients With Acute Myocardial Infarction. *J Am Coll Cardiol* 2019;74(7):842-54. doi: 10.1016/j.jacc.2019.06.025

11. Boeddinghaus J, Twerenbold R, Nestelberger T, et al. Clinical Use of a New High-Sensitivity Cardiac Troponin I Assay in Patients with Suspected Myocardial Infarction. *Clin Chem* 2019;65(11):1426-36. doi: 10.1373/clinchem.2019.304725

12. Neumann JT, Sorensen NA, Rubsamen N, et al. Evaluation of a new ultra-sensitivity troponin I assay in patients with suspected myocardial infarction. *Int J Cardiol* 2019;283:35-40. doi: 10.1016/j.ijcard.2018.12.001

13. Sorensen NA, Neumann JT, Ojeda F, et al. Diagnostic Evaluation of a High-Sensitivity Troponin I Point-of-Care Assay. *Clin Chem* 2019;65(12):1592-601. doi: 10.1373/clinchem.2019.307405

14. Boeddinghaus J, Nestelberger T, Koechlin L, et al. Early Diagnosis of Myocardial Infarction With Point-of-Care High-Sensitivity Cardiac Troponin I. *J Am Coll Cardiol* 2020;75(10):1111-24. doi: 10.1016/j.jacc.2019.12.065

15. Sörensen NA, Neumann JT, Ojeda F, et al. Differences in measurement of high-sensitivity troponin in an on-demand and batch-wise setting. *European Heart Journal Acute Cardiovascular Care* 2021;10(3):302-09. doi: 10.1177/2048872620924198

16. Sorensen NA, Gossling A, Neumann JT, et al. Diagnostic Validation of a High-Sensitivity Cardiac Troponin I Assay. *Clin Chem* 2021;67(9):1230-39. doi: 10.1093/clinchem/hvab070

17. Nestelberger T, Boeddinghaus J, Gimenez MR, et al. Direct comparison of high-sensitivity cardiac troponin T and I in

For peer review only

the early differentiation of type 1 vs. type 2 myocardial infarction. *Eur Heart J Acute Cardiovasc Care* 2022;11(1):62-74. doi: 10.1093/ehjacc/zuab039

18. Cullen L, Mueller C, Parsonage WA, et al. Validation of high-sensitivity troponin I in a 2-hour diagnostic strategy to assess 30-day outcomes in emergency department patients with possible acute coronary syndrome. *J Am Coll Cardiol* 2013;62(14):1242-49. doi: 10.1016/j.jacc.2013.02.078
19. Aldous S, Mark Richards A, George PM, et al. Comparison of new point-of-care troponin assay with high sensitivity troponin in diagnosing myocardial infarction. *Int J Cardiol* 2014;177(1):182-6. doi: 10.1016/j.ijcard.2014.09.026
20. Cullen L, Aldous S, Than M, et al. Comparison of high sensitivity troponin T and I assays in the diagnosis of non-ST elevation acute myocardial infarction in emergency patients with chest pain. *Clin Biochem* 2014;47(6):321-6. doi: 10.1016/j.clinbiochem.2013.11.019
21. Eggers KM, Aldous S, Greenslade JH, et al. Two-hour diagnostic algorithms for early assessment of patients with acute chest pain--Implications of lowering the cardiac troponin I cut-off to the 97.5th percentile. *Clin Chim Acta* 2015;445:19-24. doi: 10.1016/j.cca.2015.03.002
22. Boeddinghaus J, Reichlin T, Cullen L, et al. Two-Hour Algorithm for Triage toward Rule-Out and Rule-In of Acute Myocardial Infarction by Use of High-Sensitivity Cardiac Troponin I. *Clin Chem* 2016;62(3):494-504. doi: 10.1373/clinchem.2015.249508
23. Wildi K, Cullen L, Twerenbold R, et al. Direct Comparison of 2 Rule-Out Strategies for Acute Myocardial Infarction: 2-h Accelerated Diagnostic Protocol vs 2-h Algorithm. *Clin Chem* 2017;63(7):1227-36. doi: 10.1373/clinchem.2016.268359
24. Lindahl B, Jernberg T, Badertscher P, et al. An algorithm for rule-in and rule-out of acute myocardial infarction using a novel troponin I assay. *Heart* 2017;103(2):125-31. doi: 10.1136/heartjnl-2016-309951
25. Nestelberger T, Boeddinghaus J, Greenslade J, et al. Two-Hour Algorithm for Rapid Triage of Suspected Acute Myocardial Infarction Using a High-Sensitivity Cardiac Troponin I Assay. *Clin Chem* 2019;65(11):1437-47. doi: 10.1373/clinchem.2019.305193
26. Kavsak PA, Hewitt MK, Mondoux SE, et al. Diagnostic Performance of Serial High-Sensitivity Cardiac Troponin Measurements in the Emergency Setting. *J Cardiovasc Dev Dis* 2021;8(8) doi: 10.3390/jcdd8080097
27. Koechlin L, Boeddinghaus J, Nestelberger T, et al. Performance of the ESC 0/2h-algorithm using high-sensitivity cardiac troponin I in the early diagnosis of myocardial infarction. *Am Heart J* 2021;242:132-37. doi: 10.1016/j.ahj.2021.08.008
28. Keller T, Zeller T, Ojeda F, et al. Serial Changes in Highly Sensitive Troponin I Assay and Early Diagnosis of Myocardial Infarction. *JAMA* 2011;306(24):2684-93. doi: 10.1001/jama.2011.1896
29. Wildi K, Nelles B, Twerenbold R, et al. Safety and efficacy of the 0 h/3 h protocol for rapid rule out of myocardial infarction. *Am Heart J* 2016;181:16-25. doi: 10.1016/j.ahj.2016.07.013
30. Parsonage WA, Mueller C, Greenslade JH, et al. Validation of NICE diagnostic guidance for rule out of myocardial infarction using high-sensitivity troponin tests. *Heart* 2016;102(16):1279-86. doi: 10.1136/heartjnl-2016-309270
31. Pickering JW, Greenslade JH, Cullen L, et al. Validation of presentation and 3 h high-sensitivity troponin to rule-in and rule-out acute myocardial infarction. *Heart* 2016;102(16):1270-8. doi: 10.1136/heartjnl-2015-308505
32. Chapman AR, Anand A, Boeddinghaus J, et al. Comparison of the Efficacy and Safety of Early Rule-Out Pathways for

For peer review only

Acute Myocardial Infarction. *Circulation* 2017;135(17):1586-96. doi: 10.1161/CIRCULATIONAHA.116.025021

33. Sandoval Y, Smith SW, Thordsen SE, et al. Diagnostic Performance of High Sensitivity Compared with Contemporary Cardiac Troponin I for the Diagnosis of Acute Myocardial Infarction. *Clin Chem* 2017;63(10):1594-604. doi: 10.1373/clinchem.2017.272930
34. Chapman AR, Hesse K, Andrews J, et al. High-Sensitivity Cardiac Troponin I and Clinical Risk Scores in Patients With Suspected Acute Coronary Syndrome. *Circulation* 2018;138(16):1654-65. doi: 10.1161/CIRCULATIONAHA.118.036426
35. Gunsolus I, Sandoval Y, Smith SW, et al. Renal Dysfunction Influences the Diagnostic and Prognostic Performance of High-Sensitivity Cardiac Troponin I. *J Am Soc Nephrol* 2018;29(2):636-43. doi: 10.1681/ASN.2017030341
36. Kim JW, Kim H, Yun YM, et al. Absolute Change in High-Sensitivity Cardiac Troponin I at Three Hours After Presentation is Useful for Diagnosing Acute Myocardial Infarction in the Emergency Department. *Ann Lab Med* 2020;40(6):474-80. doi: 10.3343/alm.2020.40.6.474
37. Sandoval Y, Smith SW, Schulz K, et al. Comparison of 0/3-Hour Rapid Rule-Out Strategies Using High-Sensitivity Cardiac Troponin I in a US Emergency Department. *Circ Cardiovasc Qual Outcomes* 2020;13(7):e006565. doi: 10.1161/CIRCOUTCOMES.120.006565
38. Dupuy AM, Badiou S, Montagnon V, et al. Analytical assessment and performance of the 0/3h algorithm with novel high sensitivity cardiac troponin I. *Clin Chim Acta* 2021;519:111-17. doi: 10.1016/j.cca.2021.04.003
39. Rubini Gimenez M, Twerenbold R, Reichlin T, et al. Direct comparison of high-sensitivity-cardiac troponin I vs. T for the early diagnosis of acute myocardial infarction. *Eur Heart J* 2014;35(34):2303-11. doi: 10.1093/eurheartj/ehu188
40. Pickering JW, Young JM, George P, et al. The utility of presentation and 4-hour high sensitivity troponin I to rule-out acute myocardial infarction in the emergency department. *Clin Biochem* 2015;48(18):1219-24. doi: 10.1016/j.clinbiochem.2015.07.033
41. Miller-Hodges E, Anand A, Shah ASV, et al. High-Sensitivity Cardiac Troponin and the Risk Stratification of Patients With Renal Impairment Presenting With Suspected Acute Coronary Syndrome. *Circulation* 2018;137(5):425-35. doi: 10.1161/CIRCULATIONAHA.117.030320
42. Reichlin T, Irfan A, Twerenbold R, et al. Utility of absolute and relative changes in cardiac troponin concentrations in the early diagnosis of acute myocardial infarction. *Circulation* 2011;124(2):136-45. doi: 10.1161/CIRCULATIONAHA.111.023937
43. Reichlin T, Schindler C, Drexler B, et al. One-hour rule-out and rule-in of acute myocardial infarction using high-sensitivity cardiac troponin T. *Arch Intern Med* 2012;172(16):1211-8. doi: 10.1001/archinternmed.2012.3698
44. Reichlin T, Twerenbold R, Wildi K, et al. Prospective validation of a 1-hour algorithm to rule-out and rule-in acute myocardial infarction using a high-sensitivity cardiac troponin T assay. *CMAJ* 2015;187(8):E243-52. doi: 10.1503/cmaj.141349/-/DC1
45. Mokhtari A, Borna C, Gilje P, et al. A 1-h Combination Algorithm Allows Fast Rule-Out and Rule-In of Major Adverse Cardiac Events. *J Am Coll Cardiol* 2016;67(13):1531-40. doi: 10.1016/j.jacc.2016.01.059
46. Mokhtari A, Lindahl B, Schiopu A, et al. A 0-Hour/1-Hour Protocol for Safe, Early Discharge of Chest Pain Patients. *Acad Emerg Med* 2017;24(8):983-92. doi: 10.1111/acem.13224

For peer review only

47. Mueller C, Giannitsis E, Christ M, et al. Multicenter Evaluation of a 0-Hour/1-Hour Algorithm in the Diagnosis of Myocardial Infarction With High-Sensitivity Cardiac Troponin T. *Ann Emerg Med* 2016;68(1):76-87 e4. doi: 10.1016/j.annemergmed.2015.11.013

48. Shiozaki M, Inoue K, Suwa S, et al. Utility of the 0-hour/1-hour high-sensitivity cardiac troponin T algorithm in Asian patients with suspected non-ST elevation myocardial infarction. *Int J Cardiol* 2017;249:32-35. doi: 10.1016/j.ijcard.2017.09.009

49. Mueller-Hennessen M, Lindahl B, Giannitsis E, et al. Combined testing of copeptin and high-sensitivity cardiac troponin T at presentation in comparison to other algorithms for rapid rule-out of acute myocardial infarction. *Int J Cardiol* 2019;276:261-67. doi: 10.1016/j.ijcard.2018.10.084

50. Chew DP, Lambrakis K, Blyth A, et al. A Randomized Trial of a 1-Hour Troponin T Protocol in Suspected Acute Coronary Syndromes: The Rapid Assessment of Possible Acute Coronary Syndrome in the Emergency Department With High-Sensitivity Troponin T Study (RAPID-TnT). *Circulation* 2019;140(19):1543-56. doi: 10.1161/CIRCULATIONAHA.119.042891

51. Twerenbold R, Costabel JP, Nestelberger T, et al. Outcome of Applying the ESC 0/1-hour Algorithm in Patients With Suspected Myocardial Infarction. *J Am Coll Cardiol* 2019;74(4):483-94. doi: 10.1016/j.jacc.2019.05.046

52. Amann M, Gaiser F, Schwenk SI, et al. Evaluation of a 1-hour troponin algorithm for diagnosing myocardial infarction in high-risk patients admitted to a chest pain unit: the prospective FAST-MI cohort study. *BMJ Open* 2019;9(11):e032124. doi: 10.1136/bmjopen-2019-032124

53. Andruchow JE, Boyne T, Seiden-Long I, et al. Prospective comparative evaluation of the European Society of Cardiology (ESC) 1-hour and a 2-hour rapid diagnostic algorithm for myocardial infarction using high-sensitivity troponin-T. *CJEM* 2020;22(5):712-20. doi: 10.1017/cem.2020.349

54. Cappellini F, Falbo R, Saltafossi D, et al. Development of an algorithm for ruling-out non-ST elevation myocardial infarction in the emergency department using high sensitivity troponin T assay. *Clin Chim Acta* 2019;495:1-7. doi: 10.1016/j.cca.2019.03.1625

55. Shiozaki M, Inoue K, Suwa S, et al. Implementing the European Society of Cardiology 0-h/1-h algorithm in patients presenting very early after chest pain. *Int J Cardiol* 2020;320:1-6. doi: 10.1016/j.ijcard.2020.07.037

56. Allen BR, Christenson RH, Cohen SA, et al. Diagnostic Performance of High-Sensitivity Cardiac Troponin T Strategies and Clinical Variables in a Multisite US Cohort. *Circulation* 2021;143(17):1659-72. doi: 10.1161/CIRCULATIONAHA.120.049298

57. Dongxu C, Yannan Z, Yilin Y, et al. Evaluation of the 0 h/1 h high-sensitivity cardiac troponin T algorithm in diagnosis of non-ST-segment elevation myocardial infarction (NSTEMI) in Han population. *Clin Chem Lab Med* 2021;59(4):757-64. doi: 10.1515/cclm-2020-0367

58. Lopez-Ayala P, Nestelberger T, Boeddinghaus J, et al. Novel Criteria for the Observe-Zone of the ESC 0/1h-hs-cTnT Algorithm. *Circulation* 2021;144(10):773-87. doi: 10.1161/CIRCULATIONAHA.120.052982

59. Olsson P, Khoshnood A, Mokhtari A, et al. Glucose and high-sensitivity troponin T predict a low risk of major adverse cardiac events in emergency department chest pain patients. *Scand Cardiovasc J* 2021;55(6):354-61. doi: 10.1080/14017431.2021.1987512

60. Ruangsomboon O, Thirawattanasoot N, Chakorn T, et al. The utility of the 1-hour high-sensitivity cardiac troponin T

For peer review only

algorithm compared with and combined with five early rule-out scores in high-acuity chest pain emergency patients. *Int J Cardiol* 2021;322:23-28. doi: 10.1016/j.ijcard.2020.08.099

61. Aldous SJ, Richards AM, Cullen L, et al. Early dynamic change in high-sensitivity cardiac troponin T in the investigation of acute myocardial infarction. *Clin Chem* 2011;57(8):1154-60. doi: 10.1373/clinchem.2010.161166
62. Parsonage WA, Greenslade JH, Hammett CJ, et al. Validation of an accelerated high-sensitivity troponin T assay protocol in an Australian cohort with chest pain. *Med J Aust* 2014;200(3):161-5. doi: 10.5694/mja13.10466
63. Reichlin T, Cullen L, Parsonage WA, et al. Two-hour algorithm for triage toward rule-out and rule-in of acute myocardial infarction using high-sensitivity cardiac troponin T. *Am J Med* 2015;128(4):369-79 e4. doi: 10.1016/j.amjmed.2014.10.032
64. McRae AD, Innes G, Graham M, et al. Undetectable Concentrations of a Food and Drug Administration-approved High-sensitivity Cardiac Troponin T Assay to Rule Out Acute Myocardial Infarction at Emergency Department Arrival. *Acad Emerg Med* 2017;24(10):1267-77. doi: 10.1111/acem.13229
65. Borna C, Kollberg K, Larsson D, et al. The objective CORE score allows early rule out in acute chest pain patients. *Scand Cardiovasc J* 2018;52(6):308-14. doi: 10.1080/14017431.2018.1546891
66. Lin Z, Lim SH, Chua SJT, et al. High-sensitivity troponin T and long-term adverse cardiac events among patients presenting with suspected acute coronary syndrome in Singapore. *Singapore Med J* 2019;60(8):418-26. doi: 10.11622/smedj.2019013
67. Wildi K, Lopez-Ayala P, Koechlin L, et al. Validation of the Novel European Society of Cardiology 0/2-hour Algorithm Using Hs-cTnT in the Early Diagnosis of Myocardial Infarction. *Am J Cardiol* 2021;154:128-30. doi: 10.1016/j.amjcard.2021.06.003
68. Peacock WF, Baumann BM, Bruton D, et al. Efficacy of High-Sensitivity Troponin T in Identifying Very-Low-Risk Patients With Possible Acute Coronary Syndrome. *JAMA Cardiol* 2018;3(2):104-11. doi: 10.1001/jamacardio.2017.4625
69. Steiro OT, Tjora HL, Langorgen J, et al. Clinical risk scores identify more patients at risk for cardiovascular events within 30 days as compared to standard ACS risk criteria: the WESTCOR study. *Eur Heart J Acute Cardiovasc Care* 2021;10(3):287-301. doi: 10.1093/ehjacc/zuaa016
70. Aldous SJ, Florkowski CM, Crozier IG, et al. Comparison of high sensitivity and contemporary troponin assays for the early detection of acute myocardial infarction in the emergency department. *Ann Clin Biochem* 2011;48(Pt 3):241-8. doi: 10.1258/acb.2010.010219
71. Aldous S, Pemberton C, Richards AM, et al. High-sensitivity troponin T for early rule-out of myocardial infarction in recent onset chest pain. *Emerg Med J* 2012;29(10):805-10. doi: 10.1136/emermed-2011-200222
72. Irfan A, Reichlin T, Twerenbold R, et al. Early diagnosis of myocardial infarction using absolute and relative changes in cardiac troponin concentrations. *Am J Med* 2013;126(9):781-88 e2. doi: 10.1016/j.amjmed.2013.02.031
73. Biener M, Mueller M, Vafaie M, et al. Impact of leading presenting symptoms on the diagnostic performance of high-sensitivity cardiac troponin T and on outcomes in patients with suspected acute coronary syndrome. *Clin Chem* 2015;61(5):744-51. doi: 10.1373/clinchem.2014.235317
74. Nowak RM, Gandolfo CM, Jacobsen G, et al. Ultrarapid Rule-out for Acute Myocardial Infarction Using the Generation 5 Cardiac Troponin T Assay: Results From the REACTION-US Study. *Ann Emerg Med*

For peer review only

2018;72(6):654-64. doi: 10.1016/j.annemergmed.2018.06.021

75. Jaeger C, Wildi K, Twerenbold R, et al. One-hour rule-in and rule-out of acute myocardial infarction using high-sensitivity cardiac troponin I. *Am Heart J* 2016;171(1):92-102 e1-5. doi: 10.1016/j.ahj.2015.07.022

76. Boeddinghaus J, Twerenbold R, Nestelberger T, et al. Clinical Validation of a Novel High-Sensitivity Cardiac Troponin I Assay for Early Diagnosis of Acute Myocardial Infarction. *Clin Chem* 2018;64(9):1347-60. doi: 10.1373/clinchem.2018.286906

77. Chapman AR, Fujisawa T, Lee KK, et al. Novel high-sensitivity cardiac troponin I assay in patients with suspected acute coronary syndrome. *Heart* 2019;105(8):616-22. doi: 10.1136/heartjnl-2018-314093

78. Nowak RM, Christenson RH, Jacobsen G, et al. Performance of Novel High-Sensitivity Cardiac Troponin I Assays for 0/1-Hour and 0/2- to 3-Hour Evaluations for Acute Myocardial Infarction: Results From the HIGH-US Study. *Ann Emerg Med* 2020;76(1):1-13. doi: 10.1016/j.annemergmed.2019.12.008

79. Andersen CF, Bang C, Lauridsen KG, et al. External validation of a high-sensitive troponin I algorithm for rapid evaluation of acute myocardial infarction in a Danish cohort. *Eur Heart J Acute Cardiovasc Care* 2021;10(9):1056-64. doi: 10.1093/ehjacc/zuab062

80. McCord J, Hana A, Cook B, et al. The role of cardiac testing with the 0/1-hour high-sensitivity cardiac troponin algorithm evaluating for acute myocardial infarction. *Am Heart J* 2021;233:68-77. doi: 10.1016/j.ahj.2020.12.015

81. Greenslade JH, Carlton EW, Van Hise C, et al. Diagnostic Accuracy of a New High-Sensitivity Troponin I Assay and Five Accelerated Diagnostic Pathways for Ruling Out Acute Myocardial Infarction and Acute Coronary Syndrome. *Ann Emerg Med* 2018;71(4):439-51 e3. doi: 10.1016/j.annemergmed.2017.10.030

82. Peacock WF, Christenson R, Diercks DB, et al. Myocardial Infarction Can Be Safely Excluded by High-sensitivity Troponin I Testing 3 Hours After Emergency Department Presentation. *Acad Emerg Med* 2020 doi: 10.1111/acem.13922

83. Kavsak PA, Mondoux SE, Ma J, et al. Comparison of two biomarker only algorithms for early risk stratification in patients with suspected acute coronary syndrome. *Int J Cardiol* 2020;319:140-43. doi: 10.1016/j.ijcard.2020.06.066

84. Schofer N, Brunner F, Schlüter M, et al. Gender-specific diagnostic performance of a new highsensitivity cardiac troponin I assay for detection of acute myocardial infarction. *Eur Heart J* 2017;6(1):60-68. doi: 10.1177/2048872615626660

85. Kavsak PA, Mondoux SE, Sherbino J, et al. Clinical evaluation of Ortho Clinical Diagnostics high-sensitivity cardiac Troponin I assay in patients with symptoms suggestive of acute coronary syndrome. *Clin Biochem* 2020;80:48-51. doi: 10.1016/j.clinbiochem.2020.04.003

86. Schreiber DH, Agbo C, Wu AH. Short-term (90 min) diagnostic performance for acute non-ST segment elevation myocardial infarction and 30-day prognostic evaluation of a novel third-generation high sensitivity troponin I assay. *Clin Biochem* 2012;45(16-17):1295-301. doi: 10.1016/j.clinbiochem.2012.06.005

87. Agewall S, Olsson T, Lowbeer C. Usefulness of troponin levels below the diagnostic cut-off level for acute myocardial infarction in predicting prognosis in unselected patients admitted to the coronary care unit. *Am J Cardiol* 2007;99(10):1357-9. doi: 10.1016/j.amjcard.2006.12.059

88. Ahmadi AA, Alemzadeh-Ansari MJ, Jenab Y, et al. Comparison of High-Sensitive Cardiac Troponin-T Changes

For peer review only

between Non-ST-Segment Elevation Myocardial Infarction and Non-Coronary Artery Patients. *Iranian Red Crescent Medical Journal* 2017;19(10) doi: 10.5812/ircmj.55663

89. Alcalai R, Varshisky B, Marhig A, et al. Assessing the Performance of a Novel Point-of-Care Qualitative Assay for Early Diagnosis of Acute Coronary Syndrome. *Cardiology* 2021;146(1):34-41. doi: 10.1159/000511435

90. Aldous SJ, Florkowski CM, Crozier IG, et al. The performance of high sensitivity troponin for the diagnosis of acute myocardial infarction is underestimated. *Clin Chem Lab Med* 2011;50(4):727-9. doi: 10.1515/cclm.2011.830

91. Aldous SJ, Richards MA, Cullen L, et al. A new improved accelerated diagnostic protocol safely identifies low-risk patients with chest pain in the emergency department. *Acad Emerg Med* 2012;19(5):510-6. doi: 10.1111/j.1553-2712.2012.01352.x

92. Aldous S, Richards M, Cullen L, et al. Diagnostic and prognostic utility of early measurement with high-sensitivity troponin T assay in patients presenting with chest pain. *CMAJ* 2012;184(5):E260-8. doi: 10.1503/cmaj.110773

93. Alghamdi A, Reynard C, Morris N, et al. Diagnostic accuracy of the Troponin-only Manchester Acute Coronary Syndromes (T-MACS) decision aid with a point-of-care cardiac troponin assay. *Emerg Med J* 2020;37(4):223-28. doi: 10.1136/emermed-2019-208882

94. Al-Saleh A, Alazzoni A, Al Shalash S, et al. Performance of the high-sensitivity troponin assay in diagnosing acute myocardial infarction: systematic review and meta-analysis. *CMAJ Open* 2014;2(3):E199-207. doi: 10.9778/cmajo.20130074

95. Alushi B, Jost-Brinkmann F, Kastrati A, et al. High-Sensitivity Cardiac Troponin T in Patients with Severe Chronic Kidney Disease and Suspected Acute Coronary Syndrome. *J Clin Med* 2021;10(18) doi: 10.3390/jcm10184216

96. Anand A, Lee KK, Chapman AR, et al. High-Sensitivity Cardiac Troponin on Presentation to Rule Out Myocardial Infarction: A Stepped-Wedge Cluster Randomized Controlled Trial. *Circulation* 2021;143(23):2214-24. doi: 10.1161/CIRCULATIONAHA.120.052380

97. Apple F, Ler R, Chung A, et al. Point-of-care i-STAT cardiac troponin I for assessment of patients with symptoms suggestive of acute coronary syndrome. *Clin Chem* 2006;52(2):322-5

98. Apple FS, Pearce LA, Smith SW, et al. Role of monitoring changes in sensitive cardiac troponin I assay results for early diagnosis of myocardial infarction and prediction of risk of adverse events. *Clin Chem* 2009;55(5):930-7. doi: 10.1373/clinchem.2008.114728

99. Astley CM, Beltrame JF, Zeitz C, et al. Study design of embracing high-sensitivity troponin effectively: the value of more information: a randomized comparison. *Contemp Clin Trials* 2014;39(2):183-90. doi: 10.1016/j.cct.2014.08.012

100. Ter Avest E, Visser A, Reitsma B, et al. Point-of-care troponin T is inferior to high-sensitivity troponin T for ruling out acute myocardial infarction in the emergency department. *Eur J Emerg Med* 2016;23(2):95-101. doi: 10.1186/cc10270

101. Bahrmann P, Heppner HJ, Christ M, et al. Early detection of non-ST-elevation myocardial infarction in geriatric patients by a new high-sensitive cardiac troponin T assay. *Aging Clin Exp Res* 2012;24(3):290-4. doi: 10.3275/7927

102. Bahrmann P, Christ M, Bahrmann A, et al. A 3-hour diagnostic algorithm for non-ST-elevation myocardial infarction using high-sensitivity cardiac troponin T in unselected older patients presenting to the emergency department. *J*

For peer review only

Am Med Dir Assoc 2013;14(6):409-16. doi: 10.1016/j.jamda.2012.12.005

103. Bahrmann P, Bertsch T, Sieber CC, et al. Management of patients with chest pain presenting to the emergency department: in need for the implementation of the 1 h rapid rule-out algorithm using high-sensitivity troponin I assays in clinical practice. *Ann Transl Med* 2016;4(1):18. doi: 10.3978/j.issn.2305-5839.2015.12.31
104. Bali T, Boda S, Reynard C, et al. The association between cardiac risk factors and the probability of acute myocardial infarction in the emergency department: analysis from a multicentre prospective observational study in the high sensitivity troponin era. *Eur J Emerg Med* 2021;28(3):233-7. doi: 10.1097/MEJ.0000000000000809.
105. Ballocca F, D'Ascenzo F, Moretti C, et al. High sensitive TROponin levels In Patients with Chest pain and kidney disease: A multicenter registry - The TROPIC study. *Cardiol J* 2017;24(2):139-50. doi: 10.5603/CJ.a2017.0025
106. Bandstein N, Ljung R, Johansson M, et al. Undetectable high-sensitivity cardiac troponin T level in the emergency department and risk of myocardial infarction. *J Am Coll Cardiol* 2014;63(23):2569-78. doi: 10.1016/j.jacc.2014.03.017
107. Bandstein N, Ljung R, Holzmann MJ. Risk of revisits to the emergency department in admitted versus discharged patients with chest pain but without myocardial infarction in relation to high-sensitivity cardiac troponin T levels. *Int J Cardiol* 2016;203:341-6. doi: 10.1016/j.ijcard.2015.10.170
108. Bandstein N, Wikman A, Ljung R, et al. Survival and resource utilization in patients with chest pain evaluated with cardiac troponin T compared with high-sensitivity cardiac troponin T. *Int J Cardiol* 2017;245:43-48. doi: 10.1016/j.ijcard.2017.05.111
109. Bellini C, Cinci F, Bova G, et al. Methodology to Evaluate Clinical Impact of 0/3 Hour High-Sensitivity Cardiac Troponin T Protocol on Managing Acute Coronary Syndrome in Daily Emergency Department Practice. *Lab Med* 2021;52(5):452-59. doi: 10.1093/labmed/lmaa118
110. Bhardwaj A, Truong QA, Peacock WF, et al. A multicenter comparison of established and emerging cardiac biomarkers for the diagnostic evaluation of chest pain in the emergency department. *Am Heart J* 2011;162(2):276-82 e1. doi: 10.1016/j.ahj.2011.05.022
111. Biener M, Mueller M, Vafaie M, et al. Comparison of a 3-hour versus a 6-hour sampling-protocol using high-sensitivity cardiac troponin T for rule-out and rule-in of non-STEMI in an unselected emergency department population. *Int J Cardiol* 2013;167(4):1134-40. doi: 10.1016/j.ijcard.2012.09.122
112. Birkhahn RH, Haines E, Wen W, et al. Estimating the clinical impact of bringing a multimarker cardiac panel to the bedside in the ED. *Am J Emerg Med* 2011;29(3):304-8. doi: 10.1016/j.ajem.2009.12.007
113. Bjurman C, Larsson M, Johanson P, et al. Small changes in troponin T levels are common in patients with non-ST-segment elevation myocardial infarction and are linked to higher mortality. *J Am Coll Cardiol* 2013;62(14):1231-38. doi: 10.1016/j.jacc.2013.06.050
114. Body R, Carley S, McDowell G, et al. Rapid exclusion of acute myocardial infarction in patients with undetectable troponin using a high-sensitivity assay. *J Am Coll Cardiol* 2011;58(13):1332-9. doi: 10.1016/j.jacc.2011.06.026
115. Body R, Burrows G, Carley S, et al. High-sensitivity cardiac troponin t concentrations below the limit of detection to exclude acute myocardial infarction: a prospective evaluation. *Clin Chem* 2015;61(7):983-9. doi: 10.1373/clinchem.2014.231530
116. Body R, Burrows G, Carley S, et al. Rapid exclusion of acute myocardial infarction in patients with undetectable

troponin using a sensitive troponin I assay. *Ann Clin Biochem* 2015;52(Pt 5):543-9. doi: 10.1177/0004563215576976

117. Body R, Mueller C, Giannitsis E, et al. The Use of Very Low Concentrations of High-sensitivity Troponin T to Rule Out Acute Myocardial Infarction Using a Single Blood Test. *Acad Emerg Med* 2016;23(9):1004-13. doi: 10.1111/acem.13012

118. Body R, Morris N, Reynard C, et al. Comparison of four decision aids for the early diagnosis of acute coronary syndromes in the emergency department. *Emerg Med J* 2020;37(1):8-13. doi: 10.1136/emermed-2019-208898

119. Boeckel JN, Palapies L, Zeller T, et al. Estimation of Values below the Limit of Detection of a Contemporary Sensitive Troponin I Assay Improves Diagnosis of Acute Myocardial Infarction. *Clin Chem* 2015;61(9):1197-206. doi: 10.1373/clinchem.2015.238949

120. Boeddinghaus J, Reichlin T, Nestelberger T, et al. Early diagnosis of acute myocardial infarction in patients with mild elevations of cardiac troponin. *Clin Res Cardiol* 2017;106(6):457-67. doi: 10.1007/s00392-016-1075-9

121. Bonaca MP, Ruff CT, Kosowsky J, et al. Evaluation of the diagnostic performance of current and next-generation assays for cardiac troponin I in the BWH-TIMI ED Chest Pain Study. *Eur Heart J Acute Cardiovasc Care* 2013;2(3):195-202. doi: 10.1177/2048872613486249

122. Borna C, Thelin J, Ohlin B, et al. High-sensitivity troponin T as a diagnostic tool for acute coronary syndrome in the real world: an observational study. *Eur J Emerg Med* 2014;21(3):181-8. doi: 10.1097/MEJ.0b013e328362a71b

123. Borna C, Frostred KL, Ekelund U. Predictive role of high sensitivity troponin T within four hours from presentation of acute coronary syndrome in elderly patients. *BMC Emerg Med* 2016;16:1. doi: 10.1186/s12873-015-0064-z

124. Bove J, Hochman S, Miller J, et al. Effectiveness of 2-hour Troponin in High-risk Patients With Suspected Acute Coronary Syndrome. *Crit Pathw Cardiol* 2017;16(2):53-57. doi: 10.1097/HPC.0000000000000111

125. Braga F, Dolci A, Cavallero A, et al. Evaluation of the sensitivity of two highly sensitive troponin assays for early detection of non ST-elevation myocardial infarction (NSTEMI). *Biochimica Clinica* 2011;35(3):186-89.

126. Brichko L, Schneider HG, Chan W, et al. Rapid and safe discharge from the emergency department: A single troponin to exclude acute myocardial infarction. *Emerg Med Australas* 2018;30(4):486-93. doi: 10.1111/1742-6723.12919

127. Bularga A, Lee KK, Stewart S, et al. High-Sensitivity Troponin and the Application of Risk Stratification Thresholds in Patients With Suspected Acute Coronary Syndrome. *Circulation* 2019;140(19):1557-68. doi: 10.1161/CIRCULATIONAHA.119.042866

128. Caglar B, Serin S. Diagnostic Performance of One-hour Delta High Sensitive Troponin in Acute Coronary Syndrome. *J Coll Physicians Surg Pak* 2020;30(9):906-11. doi: 10.29271/jcpsp.2020.09.906

129. Carlton E, Greenslade J, Cullen L, et al. Evaluation of High-Sensitivity Cardiac Troponin I Levels in Patients With Suspected Acute Coronary Syndrome. *JAMA Cardiol* 2016;1(4):405-12. doi: 10.1001/jamacardio.2016.1309

130. Carlton E, Khattab A, Greaves K. Beyond triage: the diagnostic accuracy of emergency department nursing staff risk assessment in patients with suspected acute coronary syndromes. *Emerg Med j* 2016;33(2):99-104. doi: 10.1136/emermed-2015-20478

131. Carlton EW, Pickering JW, Greenslade J, et al. Assessment of the 2016 National Institute for Health and Care Excellence high-sensitivity troponin rule-out strategy. *Heart* 2018;104(8):665-72. doi: 10.1136/heartjnl-2017-311983

For peer review only

132. Carlton EW, Ingram J, Taylor H, et al. Limit of detection of troponin discharge strategy versus usual care: randomised controlled trial. *Heart* 2020;106(20):1586-94. doi: 10.1136/heartjnl-2020-316692

133. Cavender MA, White WB, Jarolim P, et al. Serial Measurement of High-Sensitivity Troponin I and Cardiovascular Outcomes in Patients With Type 2 Diabetes Mellitus in the EXAMINE Trial (Examination of Cardiovascular Outcomes With Alogliptin Versus Standard of Care). *Circulation* 2017;135(20):1911-21. doi: 10.1161/CIRCULATIONAHA.116.024632

134. Celik S, Giannitsis E, Wollert KC, et al. Cardiac troponin T concentrations above the 99th percentile value as measured by a new high-sensitivity assay predict long-term prognosis in patients with acute coronary syndromes undergoing routine early invasive strategy. *Clin Res Cardiol* 2011;100(12):1077-85. doi: 10.1007/s00392-011-0344-x

135. Chapman AR, Lee KK, McAllister DA, et al. Association of High-Sensitivity Cardiac Troponin I Concentration With Cardiac Outcomes in Patients With Suspected Acute Coronary Syndrome. *JAMA* 2017;318(19):1913-24. doi: 10.1001/jama.2017.17488

136. Charpentier S, Chenevier-Gobeaux C. [2015 ESC guidelines: 1-hour rule-out and rule-in of acute myocardial infarction with high-sensitive troponin T]. *Presse Med* 2016;45(10):859-64. doi: 10.1016/j.lpm.2016.05.023

137. Chenevier-Gobeaux C, Meune C, Lefevre G, et al. A single value of high-sensitive troponin T below the limit of detection is not enough for ruling out non ST elevation myocardial infarction in the emergency department. *Clin Biochem* 2016;49(15):1113-17. doi: 10.1016/j.clinbiochem.2016.05.021

138. Chew DP, Zeitz C, Worthley M, et al. Randomized Comparison of High-Sensitivity Troponin Reporting in Undifferentiated Chest Pain Assessment. *Circ Cardiovasc Qual Outcomes* 2016;9(5):542-53. doi: 10.1161/CIRCOUTCOMES.115.002488

139. Chew G, Frost F, Mullen L, et al. A direct comparison of decision rules for early discharge of suspected acute coronary syndromes in the era of high sensitivity troponin. *Eur Heart J Acute Cardiovasc Care* 2018;8(5):421-31. doi: 10.1177/2048872618755369

140. Christ M, Popp S, Pohlmann H, et al. Implementation of high sensitivity cardiac troponin T measurement in the emergency department. *Am J Med* 2010;123(12):1134-42. doi: 10.1016/j.amjmed.2010.07.015

141. Christ M, Geier F, Blaschke S, et al. Clinical performance of a new point-of-care cardiac troponin I test. *Clin Chem Lab Med* 2018;56(8):1336-44. doi: 10.1515/cclm-2017-0693

142. Christenson RH, Duh SH, Apple FA, et al. Pivotal findings for a high-sensitivity cardiac troponin assay: Results of the HIGH-US study. *Clin Biochem* 2020;78:32-39. doi: 10.1016/j.clinbiochem.2019.10.013

143. Chuang A, Hancock DG, Horsfall M, et al. The predictive value of high sensitivity-troponin velocity within the first 6h of presentation for cardiac outcomes regardless of acute coronary syndrome diagnosis. *Int J Cardiol* 2016;204:106-11. doi: 10.1016/j.ijcard.2015.11.132

144. Clerico A, Ripoli A, Zaninotto M, et al. Head-to-head comparison of plasma cTnI concentration values measured with three high-sensitivity methods in a large Italian population of healthy volunteers and patients admitted to emergency department with acute coronary syndrome: A multi-center study. *Clin Chim Acta* 2019;496:25-34. doi: 10.1016/j.cca.2019.06.012

145. Čolak T, Mikulić I, Landeka K, et al. Predictive Value of High Sensitive Troponin I Assay in Acute Coronary Syndrome Compared to Classic Biochemical Markers. *Psychiatr Danub* 2017;29:823-29.

For peer review only

146. Collinson P, Gaze D, Morris B, et al. Comparison of biomarker strategies for rapid rule out of myocardial infarction in the emergency department using ACC/ESC diagnostic criteria. *Ann Clin Biochem* 2006;43(4):273-80.

147. Collinson PO, Gaze DC, Bainbridge K, et al. Utility of admission cardiac troponin and "Ischemia Modified Albumin" measurements for rapid evaluation and rule out of suspected acute myocardial infarction in the emergency department. *Emerg Med J* 2006;23(4):256-61. doi: 10.1136/emj.2005.028241

148. Collinson PO, Gaze D, Goodacre S. The clinical and diagnostic performance characteristics of the high sensitivity Abbott cardiac troponin I assay. *Clin Biochem* 2015;48(4-5):275-81. doi: 10.1016/j.clinbiochem.2014.12.017

149. Unger P, Dedobbeleer C, Vanden Eynden F, et al. Mitral regurgitation after transcatheter aortic valve replacement: does the prosthesis matter? *Int J Cardiol* 2013;168(2):1706-9. doi: 10.1016/j.ijcard.2013.03.055

150. Cook B, McCord J, Hudson M, et al. Baseline High Sensitivity Cardiac Troponin I Level Below Limit of Quantitation Rules Out Acute Myocardial Infarction in the Emergency Department. *Crit Pathw Cardiol* 2021;20(1):4-9. doi: 10.1097/HPC.0000000000000230

151. Corsini A, Vagnarelli F, Bugani G, et al. Impact of high-sensitivity Troponin T on hospital admission, resources utilization, and outcomes. *Eur Heart J Acute Cardiovasc Care* 2015;4(2):148-57. doi: 10.1177/2048872614547687

152. Cramer GE, Kievit PC, Brouwer MA, et al. Lack of concordance between a rapid bedside and conventional laboratory method of cardiac troponin testing: impact on risk stratification of patients suspected of acute coronary syndrome. *Clin Chim Acta* 2007;381(2):164-6. doi: 10.1016/j.cca.2007.03.001

153. Cullen L, Parsonage WA, Greenslade J, et al. Delta troponin for the early diagnosis of AMI in emergency patients with chest pain. *Int J Cardiol* 2013;168(3):2602-8. doi: 10.1016/j.ijcard.2013.03.044

154. Cullen L, Greenslade JH, Carlton EW, et al. Sex-specific versus overall cut points for a high sensitivity troponin I assay in predicting 1-year outcomes in emergency patients presenting with chest pain. *Heart* 2016;102(2):120-6. doi: 10.1136/heartjnl-2015-308506

155. Dadkhah S, Almuwaqqat Z, Sulaiman S, et al. Sensitive Troponin I and Stress Testing in the Emergency Department for the Early Management of Chest Pain Using 2-Hour Protocol. *Crit Pathw Cardiol* 2017;16(3):89-92. doi: 10.1097/HPC.0000000000000115

156. Daly MJ, Adgey JA, Harbinson MT. Improved detection of acute myocardial infarction in patients with chest pain and significant left main stem coronary stenosis. *QJM* 2012;105(2):127-35. doi: 10.1093/qjmed/hcr134

157. Davarani H, Afzalimoghadam M, Hosseinnejad H, et al. Increasing serum troponin I and early prognosis in patients with chest pain or angina equivalent symptoms in the emergency department. *Iran J Public Health* 2012;41(2):63-69.

158. Dawson C, Benger JR, Bayly G. Serial high-sensitivity troponin measurements for the rapid exclusion of acute myocardial infarction in low-risk patients. *Emerg Med J* 2013;30(7):593-4. doi: 10.1136/emermed-2012-201574

159. Diercks DB, Peacock WFt, Hollander JE, et al. Diagnostic accuracy of a point-of-care troponin I assay for acute myocardial infarction within 3 hours after presentation in early presenters to the emergency department with chest pain. *Am Heart J* 2012;163(1):74-80 e4. doi: 10.1016/j.ahj.2011.09.028

160. Druey S, Wildi K, Twerenbold R, et al. Early rule-out and rule-in of myocardial infarction using sensitive cardiac Troponin I. *Int J Cardiol* 2015;195:163-70. doi: 10.1016/j.ijcard.2015.05.079

For peer review only

161. Eggers KM, Jaffe AS, Venge P, et al. Clinical implications of the change of cardiac troponin I levels in patients with acute chest pain - an evaluation with respect to the Universal Definition of Myocardial Infarction. *Clin Chim Acta* 2011;412(1-2):91-7. doi: 10.1016/j.cca.2010.09.020

162. Etaher A, Chew DP, Frost S, et al. Prognostic Implications of High-Sensitivity Troponin T Levels Among Patients Attending Emergency Departments and Evaluated for an Acute Coronary Syndrome. *Am J Med* 2021;134(8):1019-28 e1. doi: 10.1016/j.amjmed.2021.03.005

163. Fan LY, Yu P, Yu SS, et al. Age-specific 99th percentile cutoff of high-sensitivity cardiac troponin T for early prediction of non-ST-segment elevation myocardial infarction (NSTEMI) in middle-aged patients. *J Clin Lab Anal* 2014;28(1):10-5. doi: 10.1002/jcla.21636

164. Fanaroff AC, Schulteis RD, Pieper KS, et al. Simplified Predictive Instrument to Rule Out Acute Coronary Syndromes in a High-Risk Population. *J Am Heart Assoc* 2015;4(12) doi: 10.1161/JAHA.115.002351

165. Freund Y, Chenevier-Gobeaux C, Bonnet P, et al. High-sensitivity versus conventional troponin in the emergency department for the diagnosis of acute myocardial infarction. *Crit Care* 2011

166. Frisoli TM, Nowak R, Evans KL, et al. Henry Ford HEART Score Randomized Trial: Rapid Discharge of Patients Evaluated for Possible Myocardial Infarction. *Circ Cardiovasc Qual Outcomes* 2017;10(10) doi: 10.1161/CIRCOUTCOMES.117.003617

167. Gassenmaier T, Buchner S, Birner C, et al. High-sensitive Troponin I in acute cardiac conditions: implications of baseline and sequential measurements for diagnosis of myocardial infarction. *Atherosclerosis* 2012;222(1):116-22. doi: 10.1016/j.atherosclerosis.2012.02.007

168. Giannitsis E, Becker M, Kurz K, et al. High-sensitivity cardiac troponin T for early prediction of evolving non-ST-segment elevation myocardial infarction in patients with suspected acute coronary syndrome and negative troponin results on admission. *Clin Chem* 2010;56(4):642-50. doi: 10.1373/clinchem.2009.134460

169. Giannitsis E, Kurz K, Hallermayer K, et al. Analytical validation of a high-sensitivity cardiac troponin T assay. *Clin Chem* 2010;56(2):254-61. doi: 10.1373/clinchem.2009.132654

170. Rubini Gimenez M, Twerenbold R, Boeddinghaus J, et al. Clinical Effect of Sex-Specific Cutoff Values of High-Sensitivity Cardiac Troponin T in Suspected Myocardial Infarction. *JAMA Cardiol* 2016;1(8):912-20. doi: 10.1001/jamacardio.2016.2882

171. Rubini Gimenez M, Wildi K, Wussler D, et al. Early kinetics of cardiac troponin in suspected acute myocardial infarction. *Rev Esp Cardiol (Engl Ed)* 2020 doi: 10.1016/j.rec.2020.04.008 [published Online First: 2020/05/27]

172. Giner-Caro JA, Caballero L, Casas-Pina T, et al. High sensitive cardiac troponin T in the management of uncertain chest pain. *Int J Cardiol* 2013;168(4):4422-3. doi: 10.1016/j.ijcard.2013.05.015

173. Goodman DA, Kavsak PA, Hill SA, et al. Presenting characteristics of patients undergoing cardiac troponin measurements in the emergency department. *CJEM* 2015;17(1):62-6. doi: 10.2310/8000.2013.131298

174. Gratzel P. Neuer Algorithmus bei Infarktverdacht. *MMW Fortschritte der Medizin* 2015;157(16):9.

175. Graven T, Klykken B, Kleinau O, et al. Measurement of high-sensitivity troponin-I in suspected coronary related chest pain in Emergency Departments. *Tidsskr Nor Laegeforen* 2021;141:2021-12. doi: 10.4045/tidsskr.21.0037

176. Greenslade J, Cho E, Van Hise C, et al. Evaluating Rapid Rule-out of Acute Myocardial Infarction Using a High-Sensitivity Cardiac Troponin I Assay at Presentation. *Clin Chem* 2018;64(5):820-29. doi:

For peer review only

10.1373/clinchem.2017.283887

177. Grinstein J, Bonaca MP, Jarolim P, et al. Prognostic implications of low level cardiac troponin elevation using high-sensitivity cardiac troponin T. *Clin Cardiol* 2015;38(4):230-5. doi: 10.1002/clc.22379

178. Haaf P, Reichlin T, Twerenbold R, et al. Risk stratification in patients with acute chest pain using three high-sensitivity cardiac troponin assays. *Eur Heart J* 2014;35(6):365-75. doi: 10.1093/eurheartj/eht218

179. Haider DG, Klemenz T, Fiedler GM, et al. High sensitive cardiac troponin T: Testing the test. *Int J Cardiol* 2017;228:779-83. doi: 10.1016/j.ijcard.2016.10.043

180. Halim SA, Mulgund J, Chen AY, et al. Use of guidelines-recommended management and outcomes among women and men with low-level troponin elevation: insights from CRUSADE. *Circ Cardiovasc Qual Outcomes* 2009;2(3):199-206. doi: 10.1161/CIRCOUTCOMES.108.810127

181. Hall T, Hallén J, Agewall S, et al. Changes in diagnosing non-ST-segment elevation myocardial infarction after the introduction of a new high-sensitivity cardiac troponin T assay: a single-centre experience. *Clin Lab* 2012;58(9-10):1029-36.

182. Hochholzer W, Reichlin T, Twerenbold R, et al. Incremental value of high-sensitivity cardiac troponin T for risk prediction in patients with suspected acute myocardial infarction. *Clin Chem* 2011;57(9):1318-26. doi: 10.1373/clinchem.2011.162073

183. Hochholzer W, Valina CM, Stratz C, et al. High-sensitivity cardiac troponin for risk prediction in patients with and without coronary heart disease. *Int J Cardiol* 2014;176(2):444-9. doi: 10.1016/j.ijcard.2014.07.094

184. Hochholzer W, Neumann F. The New 2015 ESC Guidelines for the Management of Acute Coronary Syndromes in Patients Presenting Without Persistent ST-segment Elevation. *Dtsch Med Wochenschr* 2016;141(11):782-5. doi: 10.1055/s-0042-107115

185. Hoeller R, Rubini Gimenez M, Reichlin T, et al. Normal presenting levels of high-sensitivity troponin and myocardial infarction. *Heart* 2013;99(21):1567-72. doi: 10.1136/heartjnl-2013-303643

186. Hromádka M, Rajdl D, Trefil L, et al. Diagnostic sensitivity of high-sensitivity troponin T in acute myocardial infarction in patients with chest pain. *Cor et Vasa* 2012;54(7-8):e227-e31. doi: 10.1016/j.crvasa.2012.06.002

187. Huang H, Zhu S, Wang W, et al. Diagnosis of acute myocardial infarction in patients with renal insufficiency using high-sensitivity troponin T. *Clin Chem Lab Med* 2015;53(5):723-30. doi: 10.1515/cclm-2014-0715

188. Huang HL, Zhu S, Wang WQ, et al. Diagnosis of Acute Myocardial Infarction in Hemodialysis Patients With High-Sensitivity Cardiac Troponin T Assay. *Arch Pathol Lab Med* 2016;140(1):75-80. doi: 10.5858/arpa.2014-0580-OA

189. Ilva T, Lund J, Porela P, et al. Early markers of myocardial injury: cTnI is enough. *Clin Chim Acta* 2009;400(1-2):82-5. doi: 10.1016/j.cca.2008.10.005

190. Invernizzi L, Doka M, Cappellini F, et al. Effectiveness of highly sensitive troponin T assay for early diagnosis of acute myocardial infarction (AMI). *Biochimica clinica* 2013;37(1):36-39.

191. Işıksaçan N, Bıyık İ, Ertürk M, et al. Comparison of high sensitive and conventional troponin assays in diagnosis of acute myocardial infarction. *Turk J Biochem* 2017;42(1):77-85. doi: 10.1515/tjb-2016-0270

192. Iwasaki M, Yamazaki K, Ikeda N, et al. Point of care assessment of cardiac troponin T level in CKD patients with chest symptom. *Ren Fail* 2017;39(1):166-72. doi: 10.1080/0886022X.2016.1256311

For peer review only

193. Jairam S, Jones P, Samaraie L, et al. Clinical diagnosis and outcomes for Troponin T 'positive' patients assessed by a high sensitivity compared with a 4th generation assay. *Emerg Med Australas* 2011;23(4):490-501. doi: 10.1111/j.1742-6723.2011.01446.x

194. Johannessen TR, Vallersnes OM, Halvorsen S, et al. Pre-hospital One-Hour Troponin in a Low-Prevalence Population of Acute Coronary Syndrome: OUT-ACS study. *Open Heart* 2020;7(2) doi: 10.1136/openhrt-2020-001296

195. Karady J, Mayrhofer T, Ferencik M, et al. Discordance of High-Sensitivity Troponin Assays in Patients With Suspected Acute Coronary Syndromes. *J Am Coll Cardiol* 2021;77(12):1487-99. doi: 10.1016/j.jacc.2021.01.046

196. Karon BS, Wockenfus AM, Hartung KJ, et al. Comparing analytical outliers and the percent of emergency department patients with results above the 99th percentile upper reference limit for 2 conventional and one high sensitivity troponin assay. *Clin Biochem* 2018;53:104-09. doi: 10.1016/j.clinbiochem.2018.01.001

197. Kavsak PA, MacRae AR, Palomaki GE, et al. Health outcomes categorized by current and previous definitions of acute myocardial infarction in an unselected cohort of troponin-naive emergency department patients. *Clin Chem* 2006;52(11):2028-35. doi: 10.1373/clinchem.2006.073403

198. Kavsak PA, Wang X, Ko DT, et al. Short- and long-term risk stratification using a next-generation, high-sensitivity research cardiac troponin I (hs-cTnI) assay in an emergency department chest pain population. *Clin Chem* 2009;55(10):1809-15. doi: 10.1373/clinchem.2009.127241

199. Kavsak P, Hill S, Bhanich SW, et al. Biomarkers for Predicting Serious Cardiac Outcomes at 72 Hours in Patients Presenting Early after Chest Pain Onset with Symptoms of Acute Coronary Syndromes. *Clin Chem* 2012;58(1):298-302. doi: 10.1373/clinchem.2011.172064

200. Kavsak PA, Shortt C, Ma J, et al. A laboratory score at presentation to rule-out serious cardiac outcomes or death in patients presenting with symptoms suggestive of acute coronary syndrome. *Clin Chim Acta* 2017;469:69-74. doi: 10.1016/j.cca.2017.03.021

201. Kavsak P, Worster A, Shortt C, et al. High-sensitivity Cardiac Troponin Concentrations at Emergency Department Presentation in Females and Males With an Acute Cardiac Outcome. *Ann Clin Biochem* 2018;55(5):604-07. doi: 10.1177/0004563217743997

202. Kavsak PA, Worster A, Shortt C, et al. Performance of high-sensitivity cardiac troponin in the emergency department for myocardial infarction and a composite cardiac outcome across different estimated glomerular filtration rates. *Clin Chim Acta* 2018;479:166-70. doi: 10.1016/j.cca.2018.01.034

203. Kavsak PA, Cerasuolo JO, Ko DT, et al. Using the clinical chemistry score in the emergency department to detect adverse cardiac events: a diagnostic accuracy study. *CMAJ Open* 2020;8(4):E676-E84. doi: 10.9778/cmajo.20200047

204. Kelly AM. Performance of a sensitive troponin assay in the early diagnosis of acute myocardial infarction in the emergency department. *Emerg Med Australas* 2011;23(2):181-5. doi: 10.1111/j.1742-6723.2011.01388.x

205. Kelly A, Klim S. What is the 30-day rate of adverse cardiac events in chest pain patients with ED troponin I assays≤99th centile using a contemporary sensitive assay? An exploratory analysis. *Eur J Emerg Med* 2014;21(4):276-80. doi: 10.1097/MEJ.0000000000000079

206. Kelly AM, Klim S. Prospective external validation of an accelerated (2-h) acute coronary syndrome rule-out process using a contemporary troponin assay. *Int J Emerg Med* 2014;7:42. doi: 10.1186/s12245-014-0042-3

For peer review only

207. Kelly AM, Klim S. Does undetectable troponin I at presentation using a contemporary sensitive assay rule out myocardial infarction? A cohort study. *Emerg Med J* 2015;32(10):760-3. doi: 10.1136/emermed-2014-204442
208. Kemper DW, Semjonow V, de Theije F, et al. Analytical evaluation of a new point of care system for measuring cardiac Troponin I. *Clin Biochem* 2017;50(4-5):174-80. doi: 10.1016/j.clinbiochem.2016.11.011
209. Khan E, Lambrakis K, Blyth A, et al. Classification performance of clinical risk scoring in suspected acute coronary syndrome beyond a rule-out troponin profile. *Eur Heart J Acute Cardiovasc Care* 2021;10(9):1038-47. doi: 10.1093/ehjacc/zuab040
210. Khoshnood A, Erlandsson M, Isma N, et al. Diagnostic accuracy of troponin T measured >/=6h after symptom onset for ruling out myocardial infarction. *Scand Cardiovasc J* 2020;54(3):153-61. doi: 10.1080/14017431.2019.1699248
211. Kienbacher CL, Fuhrmann V, van Tulder R, et al. Impact of more conservative European Society of Cardiology guidelines on the management of patients with acute chest pain. *Int J Clin Pract* 2021;75(6):e14133. doi: 10.1111/ijcp.14133
212. Kim J, Gaddam S, Wu WC, et al. Stratified reporting of high sensitivity troponin I assay is associated with suboptimal management of patients with acute coronary syndrome and intermediate troponin elevation. *J Clin Lab Anal* 2013;27(5):402-6. doi: 10.1002/jcla.21618
213. Kimenai DM, Lindahl B, Jernberg T, et al. Sex-specific effects of implementing a high-sensitivity troponin I assay in patients with suspected acute coronary syndrome: results from SWEDEHEART registry. *Sci Rep* 2020;10(1):15227. doi: 10.1038/s41598-020-72204-2
214. Kitamura M, Hata N, Takayama T, et al. High-sensitivity cardiac troponin T for earlier diagnosis of acute myocardial infarction in patients with initially negative troponin T test--comparison between cardiac markers. *J Cardiol* 2013;62(6):336-42. doi: 10.1016/j.jjcc.2013.06.005
215. Kremneva L, Suplotov S, Shalaev S. Estimation of highly sensitive troponin tests in the diagnosis of acute coronary syndrome. *Ration Pharmacother Cardiol* 2016;12(2) doi: 10.20996/1819-6446-2016-12-2-204-209
216. Krintus M, Kozinski M, Boudry P, et al. European multicenter analytical evaluation of the Abbott ARCHITECT STAT high sensitive troponin I immunoassay. *Clin Chem Lab Med* 2014;52(11):1657-65. doi: 10.1515/cclm-2014-0107
217. Kumar A, Swamy R, Ramaswamy. Study on conventional and novel cardiac biomarkers in acute myocardial infarction. *Asian J Pharm Clin Res* 2016;9(2):327-31.
218. Lambrakis K, Papendick C, French JK, et al. Late Outcomes of the RAPID-TnT Randomized Controlled Trial: 0/1-Hour High-Sensitivity Troponin T Protocol in Suspected ACS. *Circulation* 2021;144(2):113-25. doi: 10.1161/CIRCULATIONAHA.121.055009
219. Lee H, Kerr D, O'H Ici D, et al. Clinical Significance of Initial Troponin I in the Grey Zone in Emergency Department Chest Pain Patients: A Retrospective Pilot Study. *Emerg Med J* 2010;27(4):302-4. doi: 10.1136/emj.2009.077669
220. Lehmacher J, Neumann JT, Sorensen NA, et al. Predictive Value of Serial ECGs in Patients with Suspected Myocardial Infarction. *J Clin Med* 2020;9(7) doi: 10.3390/jcm9072303
221. Li WJ, Chen XM, Nie XY, et al. Early diagnostic and prognostic utility of high-sensitive troponin assays in acute myocardial infarction: a meta-analysis. *Intern Med J* 2015;45(7):748-56. doi: 10.1111/imj.12642
222. Lim KD, Yan AT, Casanova A, et al. Quantitative troponin elevation does not provide incremental prognostic value

For peer review only

beyond comprehensive risk stratification in patients with non-ST-segment elevation acute coronary syndromes. *Am Heart J* 2008;155(4):718-24. doi: 10.1016/j.ahj.2007.11.012

223. Lin S, Yokoyama H, Rac VE, et al. Novel biomarkers in diagnosing cardiac ischemia in the emergency department: a systematic review. *Resuscitation* 2012;83(6):684-91. doi: 10.1016/j.resuscitation.2011.12.015

224. Lindahl B, Venge P, James S. The new high-sensitivity cardiac troponin T assay improves risk assessment in acute coronary syndromes. *Am Heart J* 2010;160(2):224-9. doi: 10.1016/j.ahj.2010.05.023

225. Love SA, Sandoval Y, Smith SW, et al. Incidence of Undetectable, Measurable, and Increased Cardiac Troponin I Concentrations Above the 99th Percentile Using a High-Sensitivity vs a Contemporary Assay in Patients Presenting to the Emergency Department. *Clin Chem* 2016;62(8):1115-9. doi: 10.1373/clinchem.2016.256305

226. Lynn JS, Singh A, Snoey ER. Can We Exclude the Diagnosis of Non-ST Segment Myocardial Infarction on the Basis of a Single Troponin I and a Symptom Duration >/=8 Hours? *ISRN Cardiol* 2011;2011:364728. doi: 10.5402/2011/364728

227. Maag R, Sun S, Hannon M, et al. Positive predictive value of an elevated cardiac troponin for type I myocardial infarction in ED patients based on the chief complaint. *Am J Emerg Med* 2015;33(4):516-20. doi: 10.1016/j.ajem.2015.01.003

228. Macrae AR, Kavsak PA, Lustig V, et al. Assessing the requirement for the 6-hour interval between specimens in the American Heart Association Classification of Myocardial Infarction in Epidemiology and Clinical Research Studies. *Clin Chem* 2006;52(5):812-8. doi: 10.1373/clinchem.2005.059550

229. Madsen T, Stewart M, Smyres C, et al. Significance of an Indeterminate Troponin I in Patients Evaluated for Chest Pain in an Emergency Department Observation Unit. *Crit Pathw Cardiol* 2015;14(4):146-9. doi: 10.1097/HPC.0000000000000054

230. Mahler S, Miller C, Hollander J, et al. Identifying patients for early discharge: performance of decision rules among patients with acute chest pain. *Int J Cardiol* 2013;168(2):795-802. doi: 10.1016/j.ijcard.2012.10.010

231. Marjot J, Kaier TE, Henderson K, et al. A single centre prospective cohort study addressing the effect of a rule-in/rule-out troponin algorithm on routine clinical practice. *Eur Heart J Acute Cardiovasc Care* 2019;8(5):404-11. doi: 10.1177/2048872617746850

232. Markota A, Bernhardt M, Palfy M. Comparison of point-of-care and laboratory troponin I assays. *Zdravniški vestnik2011* 2011;80(12):905-8.

233. Masotti S, Prontera C, Musetti V, et al. Evaluation of analytical performance of a new high-sensitivity immunoassay for cardiac troponin I. *Clin Chem Lab Med* 2018;56(3):492-501. doi: 10.1515/cclm-2017-0387

234. Bohula May EA, Bonaca MP, Jarolim P, et al. Prognostic performance of a high-sensitivity cardiac troponin I assay in patients with non-ST-elevation acute coronary syndrome. *Clin Chem* 2014;60(1):158-64. doi: 10.1373/clinchem.2013.206441

235. McRae A, Andruchow J. Highly-sensitive troponin T algorithm facilitates early discharge of low-risk chest pain patients within 1 h of emergency department arrival. *Evid Based Med* 2015;20(4):144. doi: 10.1136/ebmed-2015-110224

236. Melki D, Lind S, Agewall S, et al. Diagnostic value of high sensitive troponin T in chest pain patients with no persistent ST-elevations. *Scand Cardiovasc J* 2011;45(4):198-204. doi: 10.3109/14017431.2011.565792

For peer review only

237. Melki D, Lind S, Agewall S, et al. Prognostic value of combining high sensitive troponin T and N-terminal pro B-type natriuretic peptide in chest pain patients with no persistent ST-elevation. *Clin Chim Acta* 2012;413(9-10):933-7. doi: 10.1016/j.cca.2012.02.008

238. Melki D, Lugnegard J, Alfredsson J, et al. Implications of Introducing High-Sensitivity Cardiac Troponin T Into Clinical Practice: Data From the SWEDEHEART Registry. *J Am Coll Cardiol* 2015;65(16):1655-64. doi: 10.1016/j.jacc.2015.02.044

239. Meune C, Balmelli C, Twerenbold R, et al. Patients with acute coronary syndrome and normal high-sensitivity troponin. *Am J Med* 2011;124(12):1151-7. doi: 10.1016/j.amjmed.2011.07.032

240. Meune C, Reichlin T, Irfan A, et al. How safe is the outpatient management of patients with acute chest pain and mildly increased cardiac troponin concentrations? *Clin Chem* 2012;58(5):916-24. doi: 10.1373/clinchem.2011.178053

241. Miller C, Fermann G, Lindsell C, et al. Initial risk stratification and presenting characteristics of patients with evolving myocardial infarctions. *Emerg Med J* 2008;25(8):492-7. doi: 10.1136/emj.2007.052183

242. Mills N, Churchhouse A, Lee K, et al. Implementation of a sensitive troponin I assay and risk of recurrent myocardial infarction and death in patients with suspected acute coronary syndrome. *JAMA* 2011;305(12):1210-6. doi: 10.1001/jama.2011.338

243. Mills NL, Lee KK, McAllister DA, et al. Implications of lowering threshold of plasma troponin concentration in diagnosis of myocardial infarction: cohort study. *BMJ* 2012;344:e1533. doi: 10.1136/bmj.e1533

244. Le Moal E, Giuliani I, Bertinchant JP, et al. Earlier detection of myocardial infarction by an improved cardiac TnI assay. *Clin Biochem* 2007;40(13-14):1065-73. doi: 10.1016/j.clinbiochem.2007.05.004

245. Mohsen M, Shawky A. The diagnostic utility of High-Sensitivity Cardiac Troponin T in acute coronary syndrome. *The Egyptian Heart Journal* 2016;68(1):1-9. doi: 10.1016/j.ehj.2014.12.003

246. Mokhtari A, Lindahl B, Smith J, et al. Diagnostic Accuracy of High-Sensitivity Cardiac Troponin T at Presentation Combined With History and ECG for Ruling Out Major Adverse Cardiac Events. *Ann Emerg Med* 2016;68(6):649-58.e3. doi: 10.1016/j.annemergmed.2016.06.008

247. Velilla Moliner J, Gros Bañeres B, Povar Marco J, et al. Diagnostic performance of high sensitivity troponin in non-ST elevation acute coronary syndrome. *Medicina Intensiva (English Edition)* 2020;44(2):88-95. doi: 10.1016/j.medine.2019.11.001

248. Mueller M, Celik S, Biener M, et al. Diagnostic and prognostic performance of a novel high-sensitivity cardiac troponin T assay compared to a contemporary sensitive cardiac troponin I assay in patients with acute coronary syndrome. *Clin Res Cardiol* 2012;101(10):837-45. doi: 10.1007/s00392-012-0469-6

249. Mueller M, Biener M, Vafaie M, et al. Absolute and relative kinetic changes of high-sensitivity cardiac troponin T in acute coronary syndrome and in patients with increased troponin in the absence of acute coronary syndrome. *Clin Chem* 2012;58(1):209-18. doi: 10.1373/clinchem.2011.171827

250. Mueler C. Use of high-sensitivity troponin for the diagnosis of acute myocardial infarction. *Coron Artery Dis* 2013;24(8):710-2. doi: 10.1097/MCA.0000000000000049

251. Mueller M, Biener M, Vafaie M, et al. Prognostic performance of kinetic changes of high-sensitivity troponin T in acute coronary syndrome and in patients with increased troponin without acute coronary syndrome. *Int J Cardiol*

For peer review only

2014;174(3):524-9. doi: 10.1016/j.ijcard.2014.04.110

252. Mueller-Hennessen M, Mueller C, Giannitsis E, et al. Serial Sampling of High-Sensitivity Cardiac Troponin T May Not Be Required for Prediction of Acute Myocardial Infarction Diagnosis in Chest Pain Patients with Highly Abnormal Concentrations at Presentation. *Clin Chem* 2017;63(2):542-51. doi: 10.1373/clinchem.2016.258392

253. Mungai E, Hamilton B, Burns D. Comparison of High-Sensitivity Troponin T Assay to Conventional Troponin T Assay for Rule Out of Acute Coronary Syndrome in the Emergency Department. *Adv Emerg Nurs J* 2020;42(4):304-14.

254. Nacke L, Blanchet J, Reyes G, et al. Effectiveness of different cutoff points of high-sensitivity troponin T to diagnose myocardial infarction. *Revista de la Federacion Argentina de Cardiologia* 2014;43(3):141-45.

255. Nadarajah R, Gale C. The management of acute coronary syndromes in patients presenting without persistent ST-segment elevation: key points from the ESC 2020 Clinical Practice Guidelines for the general and emergency physician. *Clin Med (Lond)* 2021;21(2):e206-e11. doi: 10.7861/clinmed.2020-0879

256. Narain S, Gupta N, Sethi R, et al. Clinical Correlation of Multiple Biomarkers for Risk Assessment in Patients With Acute Coronary Syndrome. *Indian Heart J* 2008;60(6):536-42.

257. Nejatian A, Omstedt A, Hoijer J, et al. Outcomes in Patients With Chest Pain Discharged After Evaluation Using a High-Sensitivity Troponin T Assay. *J Am Coll Cardiol* 2017;69(21):2622-30. doi: 10.1016/j.jacc.2017.03.586

258. Nestelberger T, Wildi K, Boeddinghaus J, et al. Characterization of the observe zone of the ESC 2015 high-sensitivity cardiac troponin 0h/1h-algorithm for the early diagnosis of acute myocardial infarction. *Int J Cardiol* 2016;207:238-45. doi: 10.1016/j.ijcard.2016.01.112

259. Nestelberger T, Boeddinghaus J, Lopez-Ayala P, et al. Cardiovascular Biomarkers in the Early Discrimination of Type 2 Myocardial Infarction. *JAMA Cardiol* 2021;6(7):771-80. doi: 10.1001/jamacardio.2021.0669

260. Nestelberger T, Lopez-Ayala P, Boeddinghaus J, et al. External Validation and Extension of a Clinical Score for the Discrimination of Type 2 Myocardial Infarction. *J Clin Med* 2021;10(6) doi: 10.3390/jcm10061264

261. Neumann JT, Sorensen NA, Ojeda F, et al. Immediate Rule-Out of Acute Myocardial Infarction Using Electrocardiogram and Baseline High-Sensitivity Troponin I. *Clin Chem* 2017;63(1):394-402. doi: 10.1373/clinchem.2016.262659

262. Nilsson T, Johannesson E, Lundager Forberg J, et al. Diagnostic accuracy of the HEART Pathway and EDACS-ADP when combined with a 0-hour/1-hour hs-cTnT protocol for assessment of acute chest pain patients. *Emerg Med J* 2021;38(11):808-13. doi: 10.1136/emermed-2020-210833

263. Normann J, Mueller M, Biener M, et al. Effect of older age on diagnostic and prognostic performance of high-sensitivity troponin T in patients presenting to an emergency department. *Am Heart J* 2012;164(5):698-705 e4. doi: 10.1016/j.ahj.2012.08.003

264. Nowak RM, Jacobsen G, Limkakeng A, Jr., et al. Outpatient versus observation/inpatient management of emergency department patients rapidly ruled-out for acute myocardial infarction: Findings from the HIGH-US study. *Am Heart J* 2021;231:6-17. doi: 10.1016/j.ahj.2020.10.067

265. Olivieri F, Galeazzi R, Giavarina D, et al. Aged-related increase of high sensitive Troponin T and its implication in acute myocardial infarction diagnosis of elderly patients. *Mech Ageing Dev* 2012;133(5):300-5. doi: 10.1016/j.mad.2012.03.005

For peer review only

266. Omland T. New Sensitive Cardiac Troponin Assays for the Early Diagnosis of Myocardial Infarction. *Drugs Today (Barc)* 2011;47(4):303-12. doi: 10.1358/dot.2011.47.4.1533082

267. Osredkar J, Krivic K, Fabjan T, et al. Point-of-care high-sensitivity assay on PATHFAST as the backup in the emergency room. *Medicine Access @ Point of Care* 2021;5 doi: 10.1177/23992026211055095

268. Palamalai V, Murakami MM, Apple FS. Diagnostic performance of four point of care cardiac troponin I assays to rule in and rule out acute myocardial infarction. *Clin Biochem* 2013;46(16-17):1631-5. doi: 10.1016/j.clinbiochem.2013.06.026

269. Paoloni R, Kumar P, Janu M. Pilot study of high-sensitivity troponin T testing to facilitate safe early disposition decisions in patients presenting to the emergency department with chest pain. *Intern Med J* 2010;40(3):188-92. doi: 10.1111/j.1445-5994.2009.01962.x

270. Papendick C, Blyth A, Seshadri A, et al. A randomized trial of a 1-hour troponin T protocol in suspected acute coronary syndromes: Design of the Rapid Assessment of Possible ACS In the emergency Department with high sensitivity Troponin T (RAPID-TnT) study. *Am Heart J* 2017;190:25-33. doi: 10.1016/j.ahj.2017.05.004

271. Parikh RH, Seliger SL, de Lemos J, et al. Prognostic Significance of High-Sensitivity Cardiac Troponin T Concentrations between the Limit of Blank and Limit of Detection in Community-Dwelling Adults: A Metaanalysis. *Clin Chem* 2015;61(12):1524-31. doi: 10.1373/clinchem.2015.244160

272. Patsias I, Swanson B, Hudson M, et al. he (Dis) Utility of a Change in Troponin I for Diagnosis of Non-ST-Segment Elevation Myocardial Infarction in an Observation Unit. *Crit Pathw Cardiol* 2017;16(3):105-08. doi: 10.1097/HPC.0000000000000120

273. Peck D, Knott J, Lefkovits J. Clinical impact of a high-sensitivity troponin assay introduction on patients presenting to the emergency department. *Emerg Med Australas* 2016;28(3):273-8. doi: 10.1111/1742-6723.12566

274. Pickering JW, Flaws D, Smith SW, et al. A Risk Assessment Score and Initial High-sensitivity Troponin Combine to Identify Low Risk of Acute Myocardial Infarction in the Emergency Department. *Acad Emerg Med* 2018;25(4):434-43. doi: 10.1111/acem.13343

275. Pickering JW, Young JM, George PM, et al. Early kinetic profiles of troponin I and T measured by high-sensitivity assays in patients with myocardial infarction. *Clin Chim Acta* 2020;505:15-25. doi: 10.1016/j.cca.2020.02.009

276. Puelacher C, Gugala M, Adamson PD, et al. Incidence and outcomes of unstable angina compared with non-ST-elevation myocardial infarction. *Heart* 2019;105(18):1423-31. doi: 10.1136/heartjnl-2018-314305

277. Rao M, Panduranga P, Al-Mukhaini M, et al. Predictive value of a 4-hour accelerated diagnostic protocol in patients with suspected ischemic chest pain presenting to an emergency department. *Oman Med J* 2012;27(3):207-11. doi: 10.5001/omj.2012.47

278. Reichlin T, Hochholzer W, Bassetti S, et al. Early diagnosis of myocardial infarction with sensitive cardiac troponin assays. *N Engl J Med* 2009;361(9):858-67. doi: 10.1056/NEJMoa0900428

279. Reichlin T, Twerenbold R, Maushart C, et al. Risk stratification in patients with unstable angina using absolute serial changes of 3 high-sensitive troponin assays. *Am Heart J* 2013;165(3):371-8 e3. doi: 10.1016/j.ahj.2012.11.010

280. Reiter M, Reichlin T, Twerenbold R, et al. Diagnosis of acute myocardial infarction using highly sensitive cardiac troponin assays. *Eur Cardiol* 2011;7(1):18-20. doi: 10.15420/ecr.2011.7.1.18

281. Ren D, Huang T, Liu X, et al. High-sensitive cardiac troponin for the diagnosis of acute myocardial infarction in

For peer review only

different chronic kidney disease stages. *BMC Cardiovasc Disord* 2021;21(1):100. doi: 10.1186/s12872-020-01746-0

282. Renaud B, Maison P, Ngako A, et al. Impact of point-of-care testing in the emergency department evaluation and treatment of patients with suspected acute coronary syndromes. *Acad Emerg Med* 2008;15(3):216-24. doi: 10.1111/j.1553-2712.2008.00069.x

283. Roberts A. Biomarkers: hs-cTnT measurement to rule out MI. *Nat Rev Cardiol* 2014;11(6):311. doi: 10.1038/nrcardio.2014.52

284. Roos A, Bandstein N, Lundback M, et al. Stable High-Sensitivity Cardiac Troponin T Levels and Outcomes in Patients With Chest Pain. *J Am Coll Cardiol* 2017;70(18):2226-36. doi: 10.1016/j.jacc.2017.08.064

285. Roos A, Hellgren A, Rafatnia F, et al. Investigations, findings, and follow-up in patients with chest pain and elevated high-sensitivity cardiac troponin T levels but no myocardial infarction. *Int J Cardiol* 2017;232:111-16. doi: 10.1016/j.ijcard.2017.01.044

286. Röttger E, de Vries-Spithoven S, Reitsma J, et al. Safety of a 1-hour Rule-out High-sensitive Troponin T Protocol in Patients With Chest Pain at the Emergency Department. *Crit Pathw Cardiol* 2017;16(4):129-34. doi: 10.1097/HPC.0000000000000135

287. Rubini Gimenez M, Hoeller R, Reichlin T, et al. Rapid rule out of acute myocardial infarction using undetectable levels of high-sensitivity cardiac troponin. *Int J Cardiol* 2013;168(4):3896-901. doi: 10.1016/j.ijcard.2013.06.049

288. Rudolph V, Keller T, Schulz A, et al. Diagnostic and prognostic performance of myeloperoxidase plasma levels compared with sensitive troponins in patients admitted with acute onset chest pain. *Circ Cardiovasc Genet* 2012;5(5):561-8. doi: 10.1161/CIRCGENETICS.111.962290

289. Saad YM, McEwan J, Shugman IM, et al. Use of a high-sensitivity troponin T assay in the assessment and disposition of patients attending a tertiary Australian emergency department: a cross-sectional pilot study. *Emerg Med Australas* 2015;27(5):405-11. doi: 10.1111/1742-6723.12430

290. Saenger A, Korpi-Steiner N, Bryant S, et al. Utilization of a High Sensitive Troponin T Assay Optimizes Serial Sampling in the Diagnosis of Acute Myocardial Infarction Compared to Multiple Contemporary Troponin Assays. *Circ* 2010;122(21):A21588.

291. Saenger AK, Beyrau R, Braun S, et al. Multicenter analytical evaluation of a high-sensitivity troponin T assay. *Clin Chim Acta* 2011;412(9-10):748-54. doi: 10.1016/j.cca.2010.12.034

292. Sanchis J, Bardaji A, Bosch X, et al. Usefulness of high-sensitivity troponin T for the evaluation of patients with acute chest pain and no or minimal myocardial damage. *Am Heart J* 2012;164(2):194-200 e1. doi: 10.1016/j.ahj.2012.05.015

293. Sanchis J, Garcia-Blas S, Mainar L, et al. High-sensitivity versus conventional troponin for management and prognosis assessment of patients with acute chest pain. *Heart* 2014;100(20):1591-6. doi: 10.1136/heartjnl-2013-305440

294. Sanchis J, Abellan L, Garcia-Blas S, et al. Usefulness of delta troponin for diagnosis and prognosis assessment of non-ST-segment elevation acute chest pain. *Eur Heart J Acute Cardiovasc Care* 2016;5(5):399-406. doi: 10.1177/2048872615593534

295. Sandoval Y, Smith SW, Schulz KM, et al. Diagnosis of type 1 and type 2 myocardial infarction using a high-sensitivity cardiac troponin I assay with sex-specific 99th percentiles based on the third universal definition of myocardial

For peer review only

infarction classification system. *Clin Chem* 2015;61(4):657-63. doi: 10.1373/clinchem.2014.236638

296. Sandoval Y, Smith SW, Love SA, et al. Single High-Sensitivity Cardiac Troponin I to Rule Out Acute Myocardial Infarction. *Am J Med* 2017;130(9):1076-83 e1. doi: 10.1016/j.amjmed.2017.02.032

297. Sandoval Y, Smith SW, Shah AS, et al. Rapid Rule-Out of Acute Myocardial Injury Using a Single High-Sensitivity Cardiac Troponin I Measurement. *Clin Chem* 2017;63(1):369-76. doi: 10.1373/clinchem.2016.264523

298. Santalo M, Martin A, Velilla J, et al. Using high-sensitivity troponin T: the importance of the proper gold standard. *Am J Med* 2013;126(8):709-17. doi: 10.1016/j.amjmed.2013.03.003

299. Schonemann-Lund M, Schoos MM, Iversen K, et al. Retrospective Evaluation of Two Fast-track Strategies to Rule Out Acute Coronary Syndrome in a Real-life Chest Pain Population. *J Emerg Med* 2015;49(6):833-42. doi: 10.1016/j.jemermed.2015.06.026

300. Shah ASV, Anand A, Sandoval Y, et al. High-sensitivity cardiac troponin I at presentation in patients with suspected acute coronary syndrome: a cohort study. *The Lancet* 2015;386(10012):2481-88. doi: 10.1016/s0140-6736(15)00391-8

301. Shah AS, Griffiths M, Lee KK, et al. High sensitivity cardiac troponin and the under-diagnosis of myocardial infarction in women: prospective cohort study. *BMJ* 2015;350:g7873. doi: 10.1136/bmj.g7873

302. Shin YS, Ahn S, Kim YJ, et al. External validation of the emergency department assessment of chest pain score accelerated diagnostic pathway (EDACS-ADP). *Am J Emerg Med* 2020;38(11):2264-70. doi: 10.1016/j.ajem.2019.09.019

303. Shortt C, Phan K, Hill SA, et al. An approach to rule-out an acute cardiovascular event or death in emergency department patients using outcome-based cutoffs for high-sensitivity cardiac troponin assays and glucose. *Clin Biochem* 2015;48(4-5):282-7. doi: 10.1016/j.clinbiochem.2014.11.010

304. Shortt C, Ma J, Clayton N, et al. Rule-In and Rule-Out of Myocardial Infarction Using Cardiac Troponin and Glycemic Biomarkers in Patients with Symptoms Suggestive of Acute Coronary Syndrome. *Clin Chem* 2017;63(1):403-14. doi: 10.1373/clinchem.2016.261545

305. Simpson AJ, Potter JM, Koerbin G, et al. Use of observed within-person variation of cardiac troponin in emergency department patients for determination of biological variation and percentage and absolute reference change values. *Clin Chem* 2014;60(6):848-54. doi: 10.1373/clinchem.2013.219410

306. Singer AJ, Than MP, Smith S, et al. Missed myocardial infarctions in ED patients prospectively categorized as low risk by established risk scores. *Am J Emerg Med* 2017;35(5):704-09. doi: 10.1016/j.ajem.2017.01.003

307. Slagman A, von Recum J, Mockel M, et al. Diagnostic performance of a high-sensitive troponin T assay and a troponin T point of care assay in the clinical routine of an Emergency Department: A clinical cohort study. *Int J Cardiol* 2017;230:454-60. doi: 10.1016/j.ijcard.2016.12.085

308. Sorensen NA, Neumann JT, Ojeda F, et al. Challenging the 99th percentile: A lower troponin cutoff leads to low mortality of chest pain patients. *Int J Cardiol* 2017;232:289-93. doi: 10.1016/j.ijcard.2016.12.167

309. Sorensen NA, Ludwig S, Makarova N, et al. Prognostic Value of a Novel and Established High-Sensitivity Troponin I Assay in Patients Presenting with Suspected Myocardial Infarction. *Biomolecules* 2019;9(9) doi: 10.3390/biom9090469

310. Stengaard C, Sorensen JT, Ladefoged SA, et al. Quantitative point-of-care troponin T measurement for diagnosis and

For peer review only

prognosis in patients with a suspected acute myocardial infarction. *Am J Cardiol* 2013;112(9):1361-6. doi: 10.1016/j.amjcard.2013.06.026

311. Stopyra JP, Miller CD, Hiestand BC, et al. Chest Pain Risk Stratification: A Comparison of the 2-Hour Accelerated Diagnostic Protocol (ADAPT) and the HEART Pathway. *Crit Pathw Cardiol* 2016;15(2):46-9. doi: 10.1097/HPC.0000000000000072

312. Stopyra J, Snavely AC, Hiestand B, et al. Comparison of accelerated diagnostic pathways for acute chest pain risk stratification. *Heart* 2020;106(13):977-84. doi: 10.1136/heartjnl-2019-316426

313. Stoyanov KM, Biener M, Hund H, et al. Effects of crowding in the emergency department on the diagnosis and management of suspected acute coronary syndrome using rapid algorithms: an observational study. *BMJ Open* 2020;10(10):e041757. doi: 10.1136/bmjopen-2020-041757

314. Storrow AB, Christenson RH, Nowak RM, et al. Diagnostic performance of cardiac Troponin I for early rule-in and rule-out of acute myocardial infarction: Results of a prospective multicenter trial. *Clin Biochem* 2015;48(4-5):254-9. doi: 10.1016/j.clinbiochem.2014.08.018

315. Storrow AB, Nowak RM, Diercks DB, et al. Absolute and relative changes (delta) in troponin I for early diagnosis of myocardial infarction: Results of a prospective multicenter trial. *Clin Biochem* 2015;48(4-5):260-7. doi: 10.1016/j.clinbiochem.2014.09.012

316. Straface AL, Myers JH, Kirchick HJ, et al. A rapid point-of-care cardiac marker testing strategy facilitates the rapid diagnosis and management of chest pain patients in the emergency department. *Am J Clin Pathol* 2008;129(5):788-95. doi: 10.1309/9GGNMURLJWJD88W3

317. Su Q, Guo Y, Liu H, et al. Diagnostic Role of High-Sensitivity Cardiac Troponin T in Acute Myocardial Infarction and Cardiac Noncoronary Artery Disease. *Arch Med Res* 2015;46(3):193-8. doi: 10.1016/j.arcmed.2015.03.005

318. Suh D, Keller DI, Hof D, et al. Rule-out of non-ST elevation myocardial infarction by five point of care cardiac troponin assays according to the 0 h/3 h algorithm of the European Society of Cardiology. *Clin Chem Lab Med* 2018;56(4):649-57. doi: 10.1515/cclm-2017-0486

319. Suzuki K, Komukai K, Nakata K, et al. The Usefulness and Limitations of Point-of-care Cardiac Troponin Measurement in the Emergency Department. *Intern Med* 2018;57(12):1673-80. doi: 10.2169/internalmedicine.0098-17

320. Takakuwa KM, Ou F-S, Peterson ED, et al. The Usage Patterns of Cardiac Bedside Markers Employing Point-of-Care Testing for Troponin in Non-ST-Segment Elevation Acute Coronary Syndrome: Results from CRUSADE. *Clinical Cardiology* 2009;32(9):498-505. doi: 10.1002/clc.20626

321. Tecson K, Arnold W, Barrett T, et al. Interpretation of positive troponin results among patients with and without myocardial infarction. *Proc (Bayl Univ Med Cent)* 2017;30(1):11-15. doi: 10.1080/08998280.2017.11929513

322. Than M, Cullen L, Reid CM, et al. A 2-h diagnostic protocol to assess patients with chest pain symptoms in the Asia-Pacific region (ASPECT): a prospective observational validation study. *The Lancet* 2011;377(9771):1077-84. doi: 10.1016/s0140-6736(11)60310-3

323. Than M, Cullen L, Aldous S, et al. 2-Hour Accelerated Diagnostic Protocol to Assess Patients With Chest Pain Symptoms Using Contemporary Troponins as the Only Biomarker. *Journal of the American College of Cardiology* 2012;59(23):2091-8. doi: 10.1016/j.jacc.2012.02.035

For peer review only

324. Than M, Aldous S, Lord SJ, et al. A 2-hour diagnostic protocol for possible cardiac chest pain in the emergency department: a randomized clinical trial. *JAMA Intern Med* 2014;174(1):51-8. doi: 10.1001/jamainternmed.2013.11362

325. Thelin J, Melander O, Ohlin B. Early rule-out of acute coronary syndrome using undetectable levels of high sensitivity troponin T. *Eur Heart J Acute Cardiovasc Care* 2015;4(5):403-9. doi: 10.1177/2048872614554107

326. Truong QA, Bayley J, Hoffmann U, et al. Multi-marker strategy of natriuretic peptide with either conventional or high-sensitivity troponin-T for acute coronary syndrome diagnosis in emergency department patients with chest pain: from the "Rule Out Myocardial Infarction using Computer Assisted Tomography" (ROMICAT) trial. *Am Heart J* 2012;163(6):972-79 e1. doi: 10.1016/j.ahj.2012.03.010

327. Twerenbold R, Wildi K, Jaeger C, et al. Optimal Cutoff Levels of More Sensitive Cardiac Troponin Assays for the Early Diagnosis of Myocardial Infarction in Patients With Renal Dysfunction. *Circulation* 2015;131(23):2041-50. doi: 10.1161/CIRCULATIONAHA.114.014245

328. Vafaie M, Biener M, Mueller M, et al. Analytically false or true positive elevations of high sensitivity cardiac troponin: a systematic approach. *Heart* 2014;100(6):508-14. doi: 10.1136/heartjnl-2012-303202

329. van der Laarse A, Cobbaert CM, Gorgels AP, et al. Will future troponin measurement overrule the ECG as the primary diagnostic tool in patients with acute coronary syndrome? *J Electrocardiol* 2013;46(4):312-7. doi: 10.1016/j.jelectrocard.2013.02.007

330. Van Hise CB, Greenslade JH, Parsonage W, et al. External validation of heart-type fatty acid binding protein, high-sensitivity cardiac troponin, and electrocardiography as rule-out for acute myocardial infarction. *Clin Biochem* 2018;52:161-63. doi: 10.1016/j.clinbiochem.2017.10.001

331. Vasile VC, Jaffe AS. High-Sensitivity Cardiac Troponin for the Diagnosis of Patients with Acute Coronary Syndromes. *Curr Cardiol Rep* 2017;19(10):92. doi: 10.1007/s11886-017-0904-4

332. Vasudevan A, Singer AJ, DeFilippi C, et al. Renal Function and Scaled Troponin in Patients Presenting to the Emergency Department with Symptoms of Myocardial Infarction. *Am J Nephrol* 2017;45(4):304-09. doi: 10.1159/000458451

333. Venge P, Johnston N, Lindahl B, et al. Normal plasma levels of cardiac troponin I measured by the high-sensitivity cardiac troponin I access prototype assay and the impact on the diagnosis of myocardial ischemia. *J Am Coll Cardiol* 2009;54(13):1165-72. doi: 10.1016/j.jacc.2009.05.051

334. Venge P, James S, Jansson L, et al. Clinical performance of two highly sensitive cardiac troponin I assays. *Clin Chem* 2009;55(1):109-16. doi: 10.1373/clinchem.2008.106500

335. Wang W, Huang H, Zhu S, et al. High-Sensitivity Cardiac Troponin T in Patients with Acute Myocardial Infarction in Acute Exacerbation of Chronic Obstructive Pulmonary Disease. *Clin lab* 2015;61(8):1083-93.

336. Wang JA, Qin Y, Lv J, et al. Clinical application of high-sensitivity cardiac troponin T test in acute myocardial infarction diagnosis. *Genet Mol Res* 2015;14(4):17959-65. doi: 10.4238/2015.December.22.21

337. Wassef AW, Hiebert B, Saeed MF, et al. Novel high-sensitivity troponin assay requires higher cut-off value to separate acute myocardial infarction from non-acute myocardial infarction in a high-risk population. *Can J Physiol Pharmacol* 2015;93(10):873-7. doi: 10.1139/cjpp-2014-0473

338. Wassie M, Lee MS, Sun BC, et al. Single vs Serial Measurements of Cardiac Troponin Level in the Evaluation of

For peer review only

Patients in the Emergency Department With Suspected Acute Myocardial Infarction. *JAMA Netw Open* 2021;4(2):e2037930. doi: 10.1001/jamanetworkopen.2020.37930

339. Weber M, Bazzino O, Navarro Estrada JL, et al. Improved diagnostic and prognostic performance of a new high-sensitive troponin T assay in patients with acute coronary syndrome. *Am Heart J* 2011;162(1):81-8. doi: 10.1016/j.ahj.2011.04.007

340. Wereski R, Kimenai DM, Taggart C, et al. Cardiac Troponin Thresholds and Kinetics to Differentiate Myocardial Injury and Myocardial Infarction. *Circulation* 2021;144(7):528-38. doi: 10.1161/CIRCULATIONAHA.121.054302

341. Westwood M, van Asselt T, Ramaekers B, et al. High-sensitivity troponin assays for the early rule-out or diagnosis of acute myocardial infarction in people with acute chest pain: a systematic review and cost-effectiveness analysis. *Health Technol Assess* 2015;19(44):1-234. doi: 10.3310/hta19440

342. Wildi K, Reichlin T, Twerenbold R, et al. Serial changes in high-sensitivity cardiac troponin I in the early diagnosis of acute myocardial infarction. *Int J Cardiol* 2013;168(4):4103-10. doi: 10.1016/j.ijcard.2013.07.078

343. Willeit P, Welsh P, Evans JDW, et al. High-Sensitivity Cardiac Troponin Concentration and Risk of First-Ever Cardiovascular Outcomes in 154,052 Participants. *J Am Coll Cardiol* 2017;70(5):558-68. doi: 10.1016/j.jacc.2017.05.062

344. Wolf S, Kaur R, McKeown W, et al. Noise Versus Signal: The Clinical Implications of an Increasingly Sensitive Troponin Assay for Patients With Suspected Acute Coronary Syndrome. *Crit Pathw Cardiol* 2014;13(3):89-95. doi: 10.1097/HPC.0000000000000020

345. Wong P, Rao G, Innasimuthu A, et al. Validation of a prediction score model to distinguish acute coronary syndromes from other conditions causing raised cardiac troponin T levels. *Coron Artery Dis* 2010;21(6):363-8. doi: 10.1097/MCA.0b013e32833d18d8

346. Yang HS, Shemesh A, Li J, et al. No increase in the incidence of cardiac troponin I concentration above the 99th percentile by Siemens Centaur high-sensitivity compared to the contemporary assay. *Clin Biochem* 2021;89:77-80. doi: 10.1016/j.clinbiochem.2020.12.001

347. Yean KS, Abd. Wahab MB, Zakaria MIB. A study on modified accelerated diagnostic protocol to safely discharge low-risk chest pain patients in emergency department. *Hong Kong Journal of Emergency Medicine* 2019;27(3):134-45. doi: 10.1177/1024907918820750

348. Yokoyama H, Higuma T, Endo T, et al. "30-minute-delta" of high-sensitivity troponin I improves diagnostic performance in acute myocardial infarction. *J Cardiol* 2018;71(2):144-48. doi: 10.1016/j.jjcc.2017.08.003

349. Zhao Y, Sivaswamy A, Lee MK, et al. A feasibility study for CODE-MI: High-sensitivity cardiac troponin-Optimizing the diagnosis of acute myocardial infarction/injury in women. *Am Heart J* 2021;234:60-70. doi: 10.1016/j.ahj.2021.01.008

350. Advantageous Predictors of Acute Coronary Syndromes Evaluation (APACE) Study (APACE) [Available from: https://www.clinicaltrials.gov/ct2/show/NCT00470587 accessed 16 May 2020.

351. Ghali W, Knudtson M. Overview of the Alberta Provincial Project for Outcome Assessment in Coronary Heart Disease. On behalf of the APPROACH investigators. *Can J Cardiol* 2000;16(10):1225-30.

352. Biomarkers in Acute Cardiac Care (BACC) [Available from: https://clinicaltrials.gov/ct2/show/NCT02355457

For peer review only

accessed 2nd August 2020.

353. Than MP, Pickering JW, Aldous SJ, et al. Effectiveness of EDACS Versus ADAPT Accelerated Diagnostic Pathways for Chest Pain: A Pragmatic Randomized Controlled Trial Embedded Within Practice. *Ann Emerg Med* 2016;68(1):93-102 e1. doi: 10.1016/j.annemergmed.2016.01.001

354. High-Sensitivity Troponin in the Evaluation of Patients With Acute Coronary Syndrome (High-STEACS) [Available from: https://clinicaltrials.gov/ct2/show/NCT01852123 accessed 2nd August 2020.

355. Cullen L, Greenslade JH, Hawkins T, et al. Improved Assessment of Chest pain Trial (IMPACT): assessing patients with possible acute coronary syndromes. *Med J Aust* 2017;207(5):195-200. doi: 10.5694/mja16.01351

356. Kavsak P, Worster A, You J, et al. Ninety-minute vs 3-h performance of high-sensitivity cardiac troponin assays for predicting hospitalization for acute coronary syndrome. *Clin Chem* 2013;59(9):1407–10. doi: 10.1373/clinchem.2013.208595

357. Use of Abbott High Sensitivity Troponin I Assay In Acute Coronary Syndromes (UTROPIA) [Available from: https://clinicaltrials.gov/ct2/show/NCT02060760 accessed 2nd August 2020.

For peer review only

Preferred Reporting Items for Systematic reviews and Meta-Analyses extension for Scoping Reviews (PRISMA-ScR) Checklist

SECTION	ITEM	PRISMA-ScR CHECKLIST ITEM	REPORTED ON PAGE #
**TITLE**			
Title	1	Identify the report as a scoping review.	P. 1
**ABSTRACT**			
Structured summary	2	Provide a structured summary that includes (as applicable): background, objectives, eligibility criteria, sources of evidence, charting methods, results, and conclusions that relate to the review questions and objectives.	P. 2
**INTRODUCTION**			
Rationale	3	Describe the rationale for the review in the context of what is already known. Explain why the review questions/objectives lend themselves to a scoping review approach.	P. 4-5
Objectives	4	Provide an explicit statement of the questions and objectives being addressed with reference to their key elements (e.g., population or participants, concepts, and context) or other relevant key elements used to conceptualize the review questions and/or objectives.	P. 5
**METHODS**			
Protocol and registration	5	Indicate whether a review protocol exists; state if and where it can be accessed (e.g., a Web address); and if available, provide registration information, including the registration number.	P. 5
Eligibility criteria	6	Specify characteristics of the sources of evidence used as eligibility criteria (e.g., years considered, language, and publication status), and provide a rationale.	P. 6-7
Information sources*	7	Describe all information sources in the search (e.g., databases with dates of coverage and contact with authors to identify additional sources), as well as the date the most recent search was executed.	P. 6
Search	8	Present the full electronic search strategy for at least 1 database, including any limits used, such that it could be repeated.	P. 6
Selection of sources of evidence†	9	State the process for selecting sources of evidence (i.e., screening and eligibility) included in the scoping review.	P. 6-7
Data charting process‡	10	Describe the methods of charting data from the included sources of evidence (e.g., calibrated forms or forms that have been tested by the team before their use, and whether data charting was done independently or in duplicate) and any processes for obtaining and confirming data from investigators.	P. 7-9
Data items	11	List and define all variables for which data were sought and any assumptions and simplifications made.	P. 7-8
Critical appraisal of individual sources of evidence§	12	If done, provide a rationale for conducting a critical appraisal of included sources of evidence; describe the methods used and how this information was used in any data synthesis (if appropriate).	P. 7-9
Synthesis of	13	Describe the methods of handling and summarizing	P. 7-9

St. Michael's
Inspired Care.
Inspiring Science.

For peer review only - http://bmjopen.bmj.com/site/about/guidelines.xhtml

SECTION	ITEM	PRISMA-ScR CHECKLIST ITEM	REPORTED ON PAGE #
results		the data that were charted.	
**RESULTS**			
Selection of sources of evidence	14	Give numbers of sources of evidence screened, assessed for eligibility, and included in the review, with reasons for exclusions at each stage, ideally using a flow diagram.	P. 9; Figure 1; Appendix Table 2
Characteristics of sources of evidence	15	For each source of evidence, present characteristics for which data were charted and provide the citations.	Appendix Tables 1 and 2; Appendix Figure 1
Critical appraisal within sources of evidence	16	If done, present data on critical appraisal of included sources of evidence (see item 12).	P. 9-14; Table 1; Figure 2; Appendix Figure 1
Results of individual sources of evidence	17	For each included source of evidence, present the relevant data that were charted that relate to the review questions and objectives.	Appendix Tables 1 and 2
Synthesis of results	18	Summarize and/or present the charting results as they relate to the review questions and objectives.	P. 9-14; Table 1; Figure 2; Appendix Figure 1
**DISCUSSION**			
Summary of evidence	19	Summarize the main results (including an overview of concepts, themes, and types of evidence available), link to the review questions and objectives, and consider the relevance to key groups.	P. 14
Limitations	20	Discuss the limitations of the scoping review process.	P. 15-16
Conclusions	21	Provide a general interpretation of the results with respect to the review questions and objectives, as well as potential implications and/or next steps.	P. 18
**FUNDING**			
Funding	22	Describe sources of funding for the included sources of evidence, as well as sources of funding for the scoping review. Describe the role of the funders of the scoping review.	P. 19-20

JBI = Joanna Briggs Institute; PRISMA-ScR = Preferred Reporting Items for Systematic reviews and Meta-Analyses extension for Scoping Reviews.

* Where *sources of evidence* (see second footnote) are compiled from, such as bibliographic databases, social media platforms, and Web sites.

† A more inclusive/heterogeneous term used to account for the different types of evidence or data sources (e.g., quantitative and/or qualitative research, expert opinion, and policy documents) that may be eligible in a scoping review as opposed to only studies. This is not to be confused with *information sources* (see first footnote).

‡ The frameworks by Arksey and O'Malley (6) and Levac and colleagues (7) and the JBI guidance (4, 5) refer to the process of data extraction in a scoping review as data charting.

§ The process of systematically examining research evidence to assess its validity, results, and relevance before using it to inform a decision. This term is used for items 12 and 19 instead of "risk of bias" (which is more applicable to systematic reviews of interventions) to include and acknowledge the various sources of evidence that may be used in a scoping review (e.g., quantitative and/or qualitative research, expert opinion, and policy document).

From: Tricco AC, Lillie E, Zarin W, O'Brien KK, Colquhoun H, Levac D, et al. PRISMA Extension for Scoping Reviews (PRISMAScR): Checklist and Explanation. Ann Intern Med. 2018;169:467–473. doi: 10.7326/M18-0850.

St. Michael's
Inspired Care.
Inspiring Science.

For peer review only - http://bmjopen.bmj.com/site/about/guidelines.xhtml
